# Discovery of Novel Biphenyl Carboxylic Acid Derivatives as Potent URAT1 Inhibitors

**DOI:** 10.3390/molecules28217415

**Published:** 2023-11-03

**Authors:** Xianxin Hou, Yongcheng Wang, Yajun Yang, Zhiyan Xiao

**Affiliations:** Beijing Key Laboratory of Active Substance Discovery and Druggability Evaluation, Institute of Materia Medica, Chinese Academy of Medical Sciences and Peking Union Medical College, Beijing 100050, China

**Keywords:** URAT1, biphenyl carboxylic acid, hyperuricemia, gout

## Abstract

Urate transporter 1 (URAT1) is a clinically validated target for the treatment of hyperuricemia and gout. Due to the absence of protein structures, the molecular design of new URAT1 inhibitors generally resorts to ligand-based approaches. Two series of biphenyl carboxylic acids were designed based on the structures of URAT1 inhibitors Epaminurad and Telmisartan via a strategy of pharmacophore fusion. Fifty-one novel compounds were synthesized and most of them showed obvious inhibition against human URAT1. **A1** and **B21** were identified as the most potent URAT1 inhibitors in series A and B, respectively. They exhibited IC_50_ values of 0.93 μM and 0.17 μM, which were comparable or superior to the clinical uricosuric drug benzbromarone. The results confirmed the effectiveness of ligand-based approaches in identifying novel and potent URAT1 inhibitors.

## 1. Introduction

High serum uric acid concentration is an important risk factor for the development of gout. Chronically elevated uric acid levels, which is termed hyperuricemia, can lead to deposition of monosodium urate crystals in articular and non-articular structures, and hyperuricemia is the major cause of gout flares [1,2]. Besides anti-inflammatory treatment during gout attacks, effective control of the uric acid level is a key strategy for the prevention and treatment of gout. Urate lowering therapies (ULTs) aim to reverse hyperuricemia, which thereby dissolve monosodium urate crystals and prevent gout attacks in the long term.

Insufficient urate excretion is the primary reason for high serum urate (SU) levels, and urate excretion is predominantly regulated by urate transporters in the kidneys, specifically URAT1 (SLC22A12) [3,4,5,6], GLUT9 (SLC2A9) [5,7], and ABCG2 [8]. Therefore, uricosuric agents represented by URAT1 inhibitors are principal components of currently available small-molecule ULTs [9,10,11].

**Figure 1:**
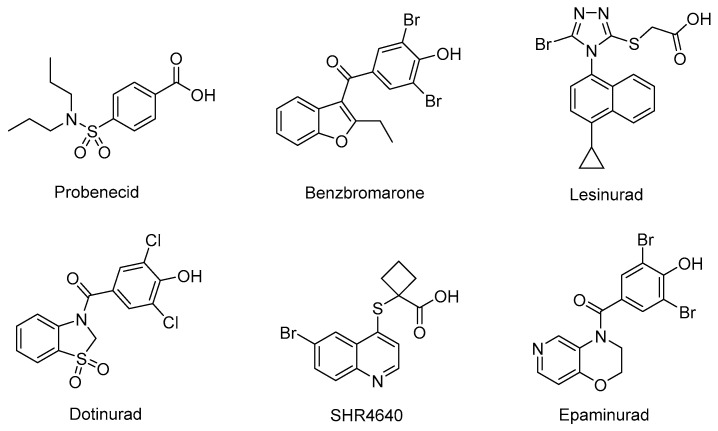
The structures of representative URAT1 Inhibitors.

URAT1 inhibitors can inhibit urate reabsorption, promote urate excretion, and thus lower the serum urate level. Hence, URAT1 inhibitors have drawn extensive attention in recent years as an effective urate-lowering therapy [11,12]. Currently, four URAT1 inhibitors, namely Probenecid, Benzbromarone, Lesinurad, and Dotinurad (Figure 1), are used in clinic for the management of hyperuricemia. Probenecid exerts its effects by inhibiting both URAT1 and GLUT9. Despite its poor efficacy, Probenecid is still an option for patients who cannot tolerate allopurinol or fail to achieve target SU in monotherapy with xanthine oxidase (XO) inhibitors [9]. Benzbromarone is a highly active URAT1 inhibitor with an IC_50_ at submicromolar level. Although it is much more effective than Probenecid, occurrence of liver toxicity has been occasionally reported. Therefore, liver function monitoring is required when prescribing this drug [9,13,14]. Lesinurad is a relatively weak URAT1 inhibitor with an IC_50_ at micromolar level. Due to its poor efficacy, Lesinurad is usually used in clinic in combination with XO inhibitors to achieve reasonable urate lowering effects [15,16]. Dotinurad is a selective urate reabsorption inhibitor (SURI). It inhibits URAT1 selectively, yet only shows minimal effects on ABCG2, OAT1, and OAT3. Dotinurad was approved in Japan for the treatment of hyperuricemia and gout in 2020 [17,18,19]. The clinic application of the marketed URAT1 inhibitors is more or less limited due to poor efficacy and potential toxicity associated with these drugs. Thus, safe and effective management of hyperuricemia is still an unmet clinic need. Most URAT1 inhibitors currently in clinical investigations are structurally derived from the marketed drugs, which might inherently share similar pharmacological and safety profiles with the previous drugs. Therefore, it is imperative to discover new chemical prototypes of URAT1 inhibitors [20,21].

Due to the transmembrane nature of URAT1, its structure has not been resolved, and the design of new URAT1 inhibitors mainly relies on ligand-based approaches [22,23,24,25]. Among the diverse URAT1 inhibitors reported, SHR4640 and Epaminurad (Figure 1) are highlighted as representative inhibitors progressed to clinical investigations. SHR4640 is a potent URAT1 inhibitor exhibiting good pharmacokinetic properties [26]. In a Phase II clinical trial in China, SHR4640 exhibited obvious urate lowering effects and good tolerability with a regimen of 5 mg or 10 mg once daily by oral administration for five weeks. SHR4640 is currently undergoing a phase III clinical trial [27,28]. Epaminurad (UR-1102) is a highly selective URAT1 inhibitor derived from benzbromarone, yet it has selectivity and safety profiles superior to benzbromarone [13,29,30].

**Figure 2:**
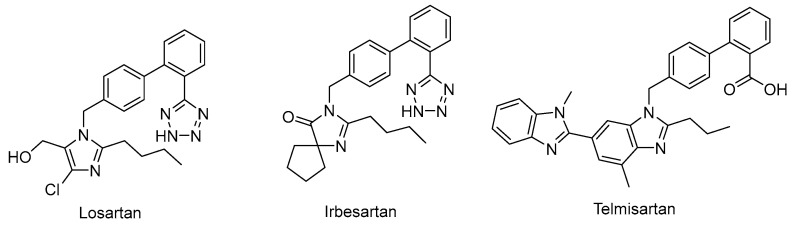
Representative angiotensin II receptor blockers with URAT1 inhibitory activity.

Drug repurposing is not only a promising field to identify new therapeutic uses for existing drugs, but also an effective approach to recognize potential leads in drug discovery [31]. For example, the angiotensin II–receptor blockers (ARBs) Losartan, Irbesartan, and Telmisartan (Figure 2) are therapeutic drugs to treat hypertension. However, urate-lowering effects were observed during their clinical application, and they were later recognized as weak URAT1 inhibitors [32], which provided a new chemical prototype for URAT1 inhibitors.

**Figure 3:**
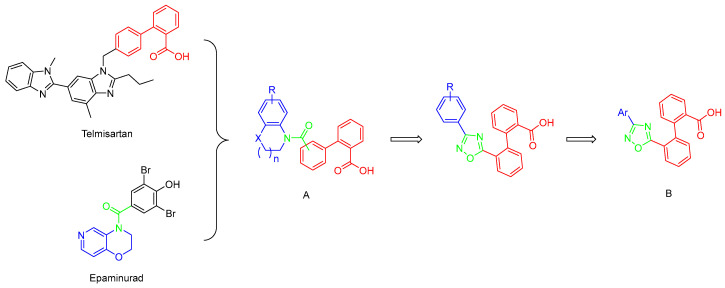
Novel biphenyl carboxylic acid-based URAT1 inhibitors designed by pharmacophore fusion. The anionic (presented in red) and hydrophobic (presented in blue) components were derived from Telmisartan and Epaminurad, respectively. The linker between the two parts was presented in green. A and B represent the general formula for compounds in Series A and B, respectively.

Careful examination of the structures of representative URAT1 inhibitors revealed the pharmacophore of URAT1 inhibitors, which includes anionic and hydrophobic components. To identify novel URAT1 inhibitors with better therapeutic profiles, we exploited a pharmacophore fusion strategy based on the structures of Telmisartan and Epaminurad (Figure 3). It was reported that the 3,5-dibromo-4-hydroxyphenyl fragment might be responsible for metabolic activation and resultant hepatic toxicity of Benzbromarone derivatives [14]. The biphenyl carboxylic acid fragment from Telmisartan was thus taken as the anionic component, while fragments similar to the 3,4-dihydro-2H-pyrido [4,3-b][1,4]oxazine part in Epaminurad were introduced as the hydrophobic component. The two parts are fused together through amide bonds to provide target compounds of series A (Figure 3), which were designed to reduce toxicity (compared to Epaminurad) and improve chemical accessibility (compared to Telmisartan) simultaneously. The amide bond in series A compounds was replaced with 1,2,4-oxadiazol via a bioisosteric strategy, and a variety of aromatic moieties were incorporated as the hydrophobic part to obtain target compounds of series B (Figure 3). These two series of compounds were both featured by the presence of the biphenyl carboxylic acid moiety, and we conducted preliminary structure–activity relationship (SAR) exploration on these compound classes to discover new chemical prototypes as potent URAT1 inhibitors.

## 2. Results and Discussion

### 2.1. Chemistry

**Scheme 1:**
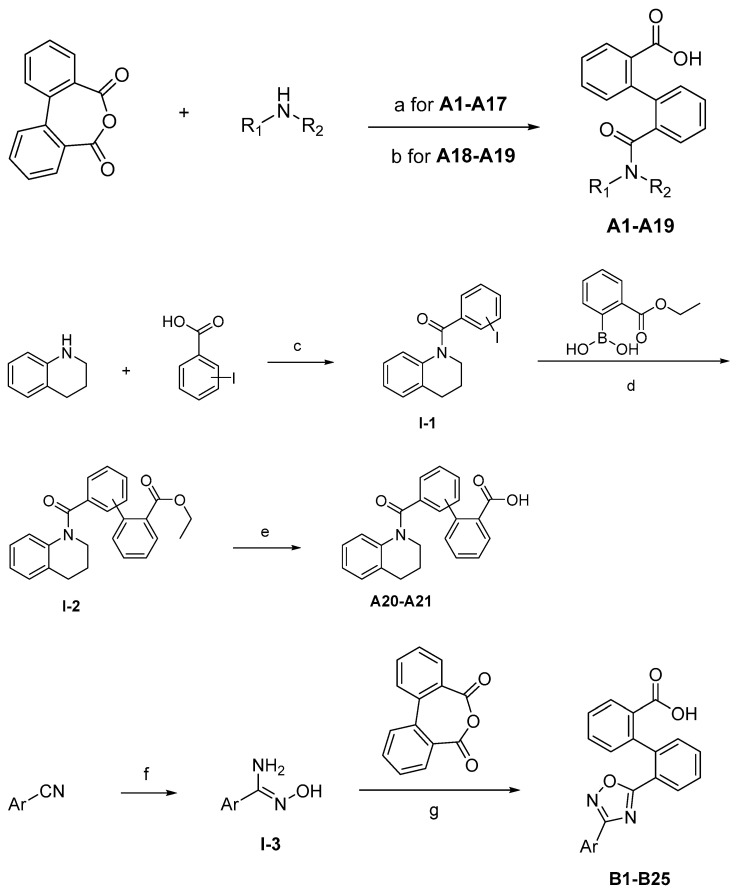

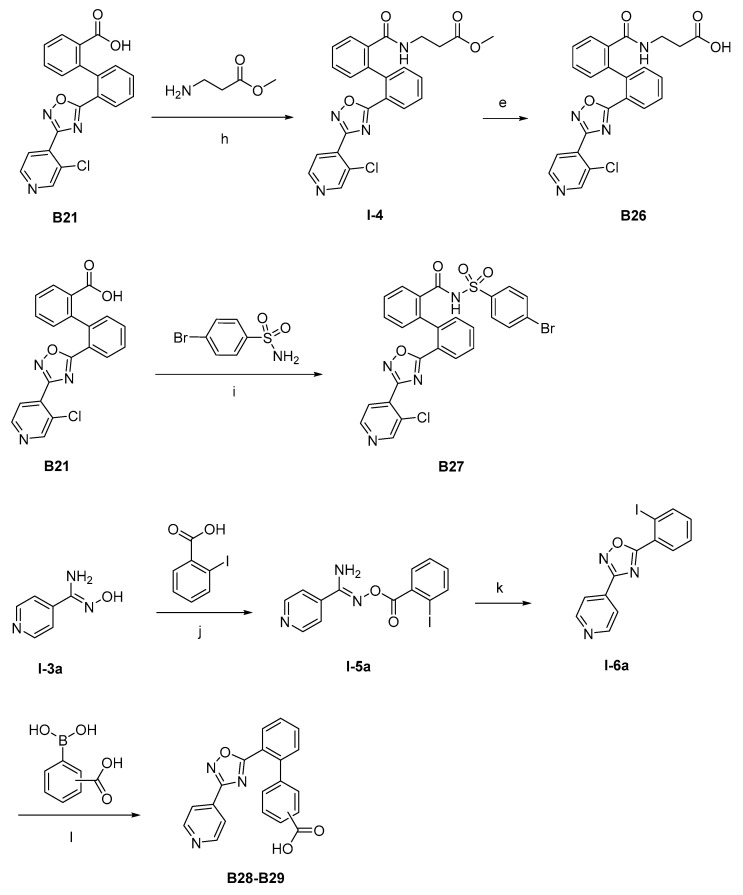

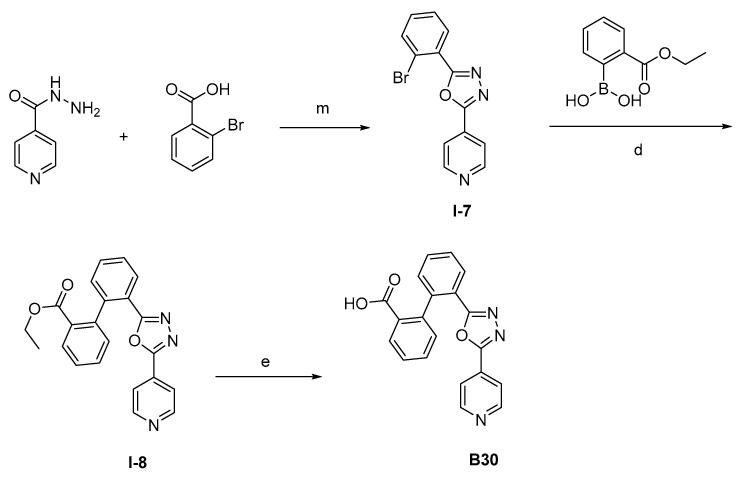
Synthetic routes of target compounds **A1**–**A21**, **B1**–**B30**. Reagents and conditions: (a) Toluene, 120 °C; (b) Triethylamine, DMAP, CH_2_Cl_2_, r.t., then 1N aq. HCl; (c) HATU, Triethylamine, Acetonitrile, r.t.; (d) K_2_CO_3_, Pd(PPh_3_)_4_, Toluene:95%Ethanol (1:1), 110 °C, N_2_; (e) LiOH.H_2_O, Ethanol:H_2_O (4:1), r.t., then 1N aq. HCl; (f) NH_2_OH.HCl, Na_2_CO_3_, Ethanol:H_2_O (4:1), 60 °C; (g) DMSO, r.t., then NaOH, r.t., then 1N aq. HCl; (h) Triethylamine, HATU, DMF, 60 °C; (i) EDCI, DMAP, CH_2_Cl_2_, r.t.; (j) Triethylamine, HATU, CH_2_Cl_2_, r.t.; (k) Cs_2_CO_3_, DMSO, r.t.; (l) K_2_CO_3_, Pd(PPh_3_)_4_, Toluene:95%Ethanol (1:1), 110 °C, N_2_, then 1N aq. HCl; (m) POCl_3_, 110 °C. DMAP: 4-dimethylaminopyridine; HATU: 2-(7-azabenzotriazol-1-yl)-*N*,*N*,*N*′,*N′*-tetramethyluronium hexafluorophosphate; EDCI: 1-ethyl-(3-dimethylaminopropyl)carbodiimide hydrochloride.

The synthesis of the target compounds were demonstrated in Scheme 1. Briefly, diphenic anhydride underwent amide condensation with substituted amines to form compounds **A1**–**A19**. Intermediate **I-1** was obtained through condensation of iodine substituted benzoic acid with 1,2,3,4-tetrahydroquinoline. **I-1** underwent a coupling reaction with carboxylic ester substituted phenylboronic acid to give intermediate **I-2**, which is then hydrolyzed to provide compounds **A20**–**A21**. Cyanides reacted with hydroxylamine hydrochloride to afford intermediate **I-3**, which reacted with diphenic anhydride to furnish the oxadiazole ring and provide compounds **B1**–**B25**. Compound **B21** was subjected to successive amidation and hydrolysis to obtain compound **B26**. Compound **B27** was also obtained from **B21** by reacting with 4-bromobenzenesulfonamide. Intermediate **I-3a** condensed with iodine substituted benzoic acid to get intermediate **I-5a**, which was then cyclized to give the oxadiazole intermediate **I-6a**. **I-6a** underwent Suzuki reaction with substituted phenylboronic acid to produce compounds **B28** and **B29**. Intermediate **I-7** furnished the 1,3,4-oxadiazole ring through the hydrazine and carboxylic acid condensation. **I-7** then underwent Suzuki reaction with substituted phenylboronic acid to form intermediate **I-8**, and subsequently hydrolyzed to obtain compound **B30**.

### 2.2. In Vitro Evaluation and Structure–Activity Relationships

All the target compounds were evaluated for their URAT1 inhibitory activities in HEK293-URAT1 cells using ^14^C-labeled uric acid as the substrate. The activity data are listed in Table 1 and Table 2. Currently available data revealed some SAR clues. The SAR exploration on series A compounds mainly focused on the hydrophobic component. Regioisomers **A1**, **A20** and **A21** were first investigated to determine the regioselectivity for URAT1 inhibition, and the ortho-substituted pattern (**A1**) emerged to be optimal. With the ortho-substituted biphenyl carboxylic acid fixed, a variety of hydrophobic components were introduced as R. This molecular area seemed sensitive to steric hindrance. Moieties with sizes similar to tetrahydroquinoline in **A1** are prone to maintain certain URAT1 inhibitory activities (**A2**–**A11**), though the activity was more or less decreased. However, moieties with sizes much larger (**A12**, **A14**) or smaller (**A16**–**A19**) than tetrahydroquinoline generally led to significantly decreased inhibitory activity. **A13** and **A15** were exceptions. The introduction of an electron-deficient pyridyl retained the URAT1 inhibitory activity (**A13**), while tetrahydroisoquinoline as R caused significant loss of activity (**A15**). These results implied the involvement of factors other than steric hindrance in affecting the inhibitory activity. For series B compounds, the ortho-substituted biphenyl carboxylic acid was initially kept, and various aromatic components were incorporated (**B1**–**B25**). Substituted phenyl (**B1**–**B7**), furanyl (**B8**), thienyl (**B9** and **B10**), pyrimidyl (**B11**), and pyridyl (**B12**–**B14**) were initially attempted, and only compound **B14** with 4-pyridyl showed significant inhibitory activity. Different substituents were introduced onto the 4-pyridyl (**B15**–**B21**). Except dimethyl substitution (**B20**), all the other substitution patterns led to increased inhibitory activities. In contrast, quinolyl (**B22**–**B24**) or methoxyl substituted naphthyl (**B25**) resulted in decreased URAT1 inhibition. Extending the carboxyl acid with β-alanine (**B26**) caused dramatic loss of activity, yet amidation of the carboxyl acid with 4-bromobenzenesulfonamide (**B27**) retained considerable inhibition. Regioisomers **B28**–**B30** were also explored, and ortho-substituted biphenyl carboxylic acid and 1,2,4-oxadiazole were preferred.

## 3. Materials and Methods

### 3.1. Chemistry

High-resolution mass spectra (HR-MS) were measured with an Agilent 6530 Accurate-Mass Quadrupole Time-of-Flight (Q-TOF) LC/MS system equipped with electrospray ionization (ESI). ^1^H NMR and ^13^C NMR spectra were recorded on a Quantum-I 400 or AVANCE NEO 700 spectrometer with TMS as the internal standard. All reagents and solvents were commercially obtained from local suppliers and were used directly without further purification.

General preparation of compounds **A1**–**A17** .The diphenic anhydride (1.0 eq) and substituted amines (1.0 eq) were mixed in 20 mL toluene. The mixture was stirred at 110 °C for 12 h, and the solvent was evaporated in a vacuum. The residue was purified by column chromatography to give **A1**–**A17**.

Compound **A1**: Yield 61%; white solid; mp: 147–149 °C; ^1^H NMR (400 MHz, DMSO-d_6_) δ7.84–7.77 (m, 1H), 7.50–7.37 (m, 5H), 7.21–7.15 (m, 1H), 7.14–7.09 (m, 1H), 7.03–6.96 (m, 2H), 6.92 (ddd, *J* = 14.6, 7.1, 1.8 Hz, 2H), 3.45 (t, *J* = 6.0 Hz, 2H), 2.43 (t, *J* = 6.9 Hz, 2H), 1.59 (*p*, *J* = 6.6 Hz, 2H); ^13^C NMR (176 MHz, DMSO-d_6_) δ168.07, 167.40, 139.05, 137.64, 137.03, 135.38, 130.89, 130.08, 129.13, 128.06, 126.97, 126.73, 124.36, 123.47, 25.22, 22.05; HR-ESI-MS: *m*/*z* = 358.1455 [M + H]^+^, calculated for C_23_H_20_NO_3_: 358.1438.

Compound **A2**: Yield 63%; white solid; mp: 174–176 °C; ^1^H NMR (400 MHz, DMSO-d_6_) δ12.36 (s, 1H), 7.87–7.74 (m, 1H), 7.55–7.37 (m, 6H), 7.24 (dd, *J* = 6.3, 2.3 Hz, 1H), 7.08 (s, 1H), 6.95 (td, *J* = 7.8, 1.6 Hz, 1H), 6.81–6.62 (m, 2H), 3.83 (s, 2H), 3.70–3.42 (m, 2H); HR-ESI-MS: *m*/*z* = 360.1252 [M + H]^+^, calculated for C_22_H_18_NO_4_: 360.1230.

Compound **A3**: Yield 51%; white solid; mp: 193–195 °C; ^1^H NMR (400 MHz, DMSO-d_6_) δ12.22 (s, 1H), 8.01–7.78 (m, 1H), 7.55–7.36 (m, 5H), 7.20 (d, *J* = 7.3 Hz, 1H), 7.09–7.02 (m, 2H), 7.00–6.94 (m, 1H), 6.92–6.77 (m, 2H), 3.71 (s, 2H), 2.93 (t, *J* = 5.5 Hz, 2H); HR-ESI-MS: *m*/*z* = 376.1001 [M + H]^+^, calculated for C_22_H_18_NO_3_S: 376.1002.

Compound **A4**: Yield 66%; whsite solid; mp: 195–196 °C; ^1^H NMR (400 MHz, DMSO-d_6_) δ12.72 (s, 1H), 7.93 (d, *J* = 8.0 Hz, 1H), 7.77 (dd, *J* = 7.7, 1.4 Hz, 1H), 7.57–7.43 (m, 4H), 7.37 (dt, *J* = 8.9, 7.2 Hz, 2H), 7.28 (d, *J* = 6.9 Hz, 1H), 7.21–7.08 (m, 2H), 6.99 (d, *J* = 7.7 Hz, 1H), 3.79 (s, 2H), 2.87 (s, 2H); HR-ESI-MS: *m*/*z* = 344.1277 [M + H]^+^, calculated for C_22_H_18_NO_3_: 344.1281.

Compound **A5**: Yield 64%; white solid; mp: 215–217 °C; ^1^H NMR (400 MHz, DMSO-d_6_) δ7.86 (d, *J* = 7.6 Hz, 1H), 7.63–6.93 (m, 10H), 6.75–6.59 (m, 1H), 3.25 (s, 2H), 2.56 (s, 1H), 1.57 (m, 4H); HR-ESI-MS: *m*/*z* = 372.1594 [M + H]^+^, calculated for C_24_H_22_NO_3_: 372.1594.

Compound **A6**: Yield 66%; white solid; mp: 231–233 °C; ^1^H NMR (400 MHz, DMSO-d_6_) δ12.29 (s, 1H), 7.86–7.77 (m, 1H), 7.52–7.36 (m, 6H), 7.25–7.20 (m, 1H), 7.12 (dd, *J* = 8.2, 2.0 Hz, 1H), 7.05–6.99 (m, 1H), 6.96 (d, *J* = 8.2 Hz, 1H), 3.43 (t, *J* = 6.0 Hz, 2H), 2.39 (d, *J* = 6.9 Hz, 2H), 1.56 (*p*, *J* = 6.5 Hz, 2H); HR-ESI-MS: *m*/*z* = 436.0553 [M + H]^+^, calculated for C_23_H_19_BrNO_3_: 436.0543.

Compound **A7**: Yield 65%; white solid; mp: 261–262 °C; ^1^H NMR (400 MHz, DMSO-d_6_) δ12.27 (s, 1H), 7.81 (dd, *J* = 6.7, 2.3 Hz, 1H), 7.51–7.37 (m, 5H), 7.20 (q, *J* = 2.8 Hz, 2H), 7.13–7.01 (m, 3H), 3.43 (t, *J* = 6.0 Hz, 2H), 2.44 (d, *J* = 6.9 Hz, 2H), 1.58 (*p*, *J* = 6.5 Hz, 2H); HR-ESI-MS: *m*/*z* = 436.0544 [M + H]^+^, calculated for C_23_H_19_BrNO_3_: 436.0543.

Compound **A8**: Yield 61%; white solid; mp: 201–202 °C; ^1^H NMR (400 MHz, DMSO-d_6_) δ12.26 (s, 1H), 7.82 (dt, *J* = 6.7, 2.1 Hz, 1H), 7.52–7.37 (m, 5H), 7.27 (d, *J* = 7.9 Hz, 1H), 7.17 (dt, *J* = 6.5, 2.0 Hz, 1H), 7.04 (d, *J* = 8.3 Hz, 1H), 6.88 (tt, *J* = 9.9, 8.1, 3.5 Hz, 2H), 3.45 (t, *J* = 5.8 Hz, 2H), 2.40 (t, *J* = 7.2 Hz, 2H), 1.59 (t, *J* = 6.5 Hz, 2H); HR-ESI-MS: *m*/*z* = 436.0552 [M + H]^+^, calculated for C_23_H_19_BrNO_3_: 436.0543.

Compound **A9**: Yield 65%; white solid; mp: 233–234 °C; ^1^H NMR (400 MHz, DMSO-d_6_) δ12.27 (s, 1H), 7.83 (dt, *J* = 7.4, 1.7 Hz, 1H), 7.53–7.38 (m, 5H), 7.31–7.19 (m, 2H), 7.08–6.93 (m, 3H), 3.43 (t, *J* = 6.1 Hz, 2H), 2.43 (t, *J* = 6.9 Hz, 2H), 1.57 (t, *J* = 6.4 Hz, 2H); HR-ESI-MS: *m*/*z* = 392.1064 [M + H]^+^, calculated for C_23_H_19_ClNO_3_: 392.1048.

Compound **A10**: Yield 65%; white solid; mp: 198–200 °C; ^1^H NMR (400 MHz, DMSO-d_6_) δ12.27 (s, 1H), 7.87–7.74 (m, 1H), 7.48–7.35 (m, 5H), 7.18 (dq, *J* = 4.2, 2.4 Hz, 1H), 7.05 (s, 2H), 6.58 (d, *J* = 2.9 Hz, 1H), 6.52 (dd, *J* = 8.9, 2.9 Hz, 1H), 3.69 (s, 3H), 3.41 (t, *J* = 6.1 Hz, 2H), 2.41 (d, *J* = 8.5 Hz, 2H), 1.57 (*p*, *J* = 6.4 Hz, 2H); HR-ESI-MS: *m*/*z* = 388.1536 [M + H]^+^, calculated for C_24_H_22_NO_4_: 388.1543.

Compound **A11**: Yield 67%; white solid; mp: 220–222 °C; ^1^H NMR (400 MHz, DMSO-d_6_) δ7.89–7.72 (m, 1H), 7.41 (dq, *J* = 5.3, 2.5, 1.9 Hz, 5H), 7.26–7.15 (m, 1H), 7.01 (s, 2H), 6.81 (d, *J* = 2.0 Hz, 1H), 6.73 (dd, *J* = 8.3, 2.1 Hz, 1H), 3.42 (t, *J* = 6.1 Hz, 2H), 2.40 (t, *J* = 6.9 Hz, 2H), 2.19 (s, 3H), 1.57 (*p*, *J* = 6.5 Hz, 2H); HR-ESI-MS: *m*/*z* = 372.1588 [M + H]^+^, calculated for C_24_H_22_NO_3_: 372.1594.

Compound **A12**: Yield 55%; white solid; mp: 245–246 °C; ^1^H NMR (400 MHz, DMSO-d_6_) δ12.07 (s, 1H), 7.82 (dd, *J* = 7.0, 2.2 Hz, 1H), 7.59–7.12 (m, 12H), 7.05 (d, *J* = 7.9 Hz, 1H), 6.85 (d, *J* = 6.9 Hz, 1H), 3.50 (t, *J* = 6.0 Hz, 2H), 2.40 (d, *J* = 6.8 Hz, 2H), 1.59 (*p*, *J* = 6.4 Hz, 2H); HR-ESI-MS: *m*/*z* = 452.1649 [M + H]^+^, calculated for C_29_H_23_FNO_3_: 452.1656.

Compound **A13**: Yield 55%; white solid; mp: 266–268 °C; ^1^H NMR (400 MHz, DMSO-d_6_) δ12.22 (s, 1H), 8.62–8.40 (m, 2H), 7.96–7.76 (m, 1H), 7.58 (dd, *J* = 7.0, 2.0 Hz, 1H), 7.49 (dtd, *J* = 14.1, 7.4, 1.7 Hz, 2H), 7.44–7.31 (m, 4H), 7.28 (d, *J* = 5.1 Hz, 2H), 7.19 (dd, *J* = 7.0, 1.9 Hz, 1H), 7.11 (d, *J* = 7.9 Hz, 1H), 6.85 (s, 1H), 3.49 (d, *J* = 6.1 Hz, 2H), 2.40 (s, 2H), 1.58 (*p*, *J* = 6.4 Hz, 2H); HR-ESI-MS: *m*/*z* = 435.1696 [M + H]^+^, calculated for C_28_H_23_N_2_O_3_: 435.1703.

Compound **A14**: Yield 54%; white solid; mp: 177–179 °C; ^1^H NMR (400 MHz, DMSO-d_6_) δ12.19 (s, 1H), 7.81 (dd, *J* = 7.4, 1.7 Hz, 1H), 7.60–7.53 (m, 1H), 7.51–7.44 (m, 2H), 7.43–7.34 (m, 2H), 7.28 (t, *J* = 7.9 Hz, 1H), 7.20 (ddd, *J* = 13.1, 7.5, 2.2 Hz, 3H), 7.04 (d, *J* = 7.9 Hz, 1H), 6.93–6.75 (m, 4H), 3.80 (s, 3H), 3.50 (s, 2H), 2.38 (s, 2H), 1.58 (t, *J* = 6.4 Hz, 2H); HR-ESI-MS: *m*/*z* = 464.1849 [M + H]^+^, calculated for C_30_H_26_NO_4_: 464.1856.

Compound **A15**: Yield 62%; white solid; mp: 171–173 °C; ^1^H NMR (400 MHz, DMSO-d_6_) δ12.74 (s, 1H), 7.76 (dd, *J* = 7.7, 1.5 Hz, 1H), 7.50–6.48 (m, 11H), 4.29 (s, 2H), 3.33 (s, 2H), 2.73–2.17 (m, 2H); HR-ESI-MS: *m*/*z* = 358.1448 [M + H]^+^, calculated for C_23_H_20_NO_3_: 358.1438.

Compound **A16**: Yield 53%; white solid; mp: 169–172 °C; ^1^H NMR (400 MHz, DMSO-d_6_) δ12.64 (s, 1H), 7.85 (d, *J* = 7.7 Hz, 1H), 7.69–6.66 (m, 12H), 3.04 (s, 3H); HR-ESI-MS: *m*/*z* = 332.1284 [M + H]^+^, calculated for C_21_H_18_NO_3_: 332.1281.

Compound **A17**: Yield 58%; white solid; mp: 118–120 °C; ^1^H NMR (400 MHz, DMSO-d_6_) δ12.74 (s, 1H), 8.42 (t, *J* = 6.1 Hz, 1H), 8.39–8.31 (m, 2H), 7.81 (dd, *J* = 7.5, 1.7 Hz, 1H), 7.59–7.41 (m, 5H), 7.16 (td, *J* = 6.4, 2.4 Hz, 2H), 6.90–6.83 (m, 2H), 4.25 (d, *J* = 5.9 Hz, 2H); HR-ESI-MS: *m*/*z* = 333.1246 [M + H]^+^, calculated for C_20_H_17_N_2_O_3_: 333.1234.

General preparation of compounds **A18** and **A19** . The diphenic anhydride (1.0 eq), aromatic amines (1.0 eq), triethylamine (3.0 eq), and 4-dimethylaminopyridine (0.2 eq) were mixed in 20 mL dichloromethane. The mixture was stirred at room temperature for 10 h, and the solvent was evaporated in a vacuum. The reaction mixture was poured into water (50 mL), 1 M HCl solution was added dropwise to separated water phase to adjust the pH to 6 and the aqueous residue was extracted with dichloromethane. The combined organic solution was washed with saturated aqueous sodium chloride solution (30 mL × 3) and dried over anhydrous Na_2_SO_4_. The residue was purified by column chromatography to **A18** and **A19**.

Compound **A18**: Yield 55%; white solid; mp: 190–191 °C; ^1^H NMR (400 MHz, DMSO-d_6_) δ12.63 (s, 1H), 8.33–8.25 (m, 2H), 7.68–7.56 (m, 2H), 7.52–7.43 (m, 2H), 7.39–7.35 (m, 2H), 7.27 (tt, *J* = 7.4, 5.7 Hz, 2H), 7.16–7.05 (m, 1H), 6.99–6.91 (m, 1H); HR-ESI-MS: *m*/*z* = 319.1092 [M + H]^+^, calculated for C_19_H_14_N_2_O_3_: 319.1077.

Compound **A19**: Yield 53%; white solid; mp: 176–178 °C; ^1^H NMR (400 MHz, DMSO-d_6_) δ8.68 (d, *J* = 5.0 Hz, 1H), 7.88 (dd, *J* = 12.7, 8.5 Hz, 2H), 7.82 (d, *J* = 5.0 Hz, 1H), 7.70–7.61 (m, 2H), 7.52–7.45 (m, 3H), 7.39 (t, *J* = 7.7 Hz, 1H), 7.24 (dd, *J* = 6.2, 2.8 Hz, 2H), 7.10 (dd, *J* = 5.9, 3.0 Hz, 1H), 7.05 (d, *J* = 4.0 Hz, 1H); HR-ESI-MS: *m*/*z* = 369.1253 [M + H]^+^, calculated for C_23_H_17_N_2_O_3_: 369.1234.

General preparation of intermediate **I-1**. Iodine substituted benzoic acid (1.2 eq) and 1,2,3,4-tetrahydroquinoline (1.0 eq) were dissolved in 20 mL acetonitrile, then HATU (1.5 eq) and triethylamine (2.0 eq) were added. The mixture was stirred at room temperature for 10 h, and the solvent was evaporated in a vacuum. The residue was purified by column chromatography to give intermediate **I-1**.

General preparation of intermediate **I-2**. The intermediate **I-1** (1.0 eq), substituted phenylboronic acids (1.0 eq), potassium carbonate (3.0 eq), and tetrakis(triphenylphosphine)palladium (0.2 eq) were added into 20 mL of a mixed solvent of toluene: 95%ethanol (1:1). The mixture was stirred at 110 °C under N_2_ for 12 h. The solvent was then evaporated in a vacuum and the residue was purified by column chromatography to get intermediate **I-2**.

General preparation of compounds **A20** and **A21**. The intermediate **I-2** (1.0 eq) and lithium hydroxide monohydrate (2.0 eq) were dissolved in 20 mL of a mixed solvent of ethanol: H_2_O (4:1). The mixture was stirred at room temperature for 8 h, and the solvent was evaporated in a vacuum. The reaction mixture was poured into water (50 mL). 1 M HCl solution was added dropwise to adjust the pH to 2. The mixture was extracted with ethyl acetate (50 mL × 3). The combined organic solution was washed with saturated aqueous sodium chloride solution (30 mL × 3) and dried over anhydrous Na_2_SO_4_. The residue was purified by column chromatography to **A20** and **A21**.

Compound **A20**: Yield 59%; white solid; mp: 165–167 °C; ^1^H NMR (400 MHz, DMSO-d_6_) δ7.74 (dd, *J* = 7.7, 1.4 Hz, 1H), 7.66–7.53 (m, 2H), 7.47 (td, *J* = 7.5, 1.3 Hz, 1H), 7.42–7.34 (m, 3H), 7.33–7.28 (m, 2H), 7.23–7.15 (m, 1H), 7.01 (td, *J* = 7.3, 1.6 Hz, 1H), 6.93 (dd, *J* = 14.9, 7.8 Hz, 2H), 3.77 (t, *J* = 6.6 Hz, 2H), 2.83 (t, *J* = 6.6 Hz, 2H), 1.96 (*p*, *J* = 6.6 Hz, 2H); ^13^C NMR (176 MHz, DMSO-d_6_) δ168.71, 168.60, 142.00, 139.68, 138.20, 134.56, 130.49, 130.35, 129.79, 128.67, 128.12, 127.94, 127.49(2C), 127.27(2C), 127.04, 124.83, 124.32, 123.68, 44.09, 25.62, 22.96; HR-ESI-MS: *m*/*z* = 358.1445 [M + H]^+^, calculated for C_23_H_20_NO_3_: 358.1438.

Compound **A21**: Yield 62%; white solid; mp: 100–102 °C; ^1^H NMR (400 MHz, DMSO-d_6_) δ12.85 (s, 1H), 7.74 (dd, *J* = 7.7, 1.4 Hz, 1H), 7.53 (td, *J* = 7.5, 1.5 Hz, 1H), 7.45 (td, *J* = 7.6, 1.3 Hz, 1H), 7.39–7.34 (m, 2H), 7.33–7.25 (m, 2H), 7.18 (ddd, *J* = 9.5, 7.5, 1.5 Hz, 2H), 7.02 (td, *J* = 7.4, 1.4 Hz, 1H), 6.94 (td, *J* = 7.7, 7.2, 1.6 Hz, 1H), 6.86 (d, *J* = 8.2 Hz, 1H), 3.77 (t, *J* = 6.6 Hz, 2H), 2.81 (t, *J* = 6.6 Hz, 2H), 1.95 (*p*, *J* = 6.6 Hz, 2H); ^13^C NMR (176 MHz, DMSO-d_6_) δ168.78, 168.51, 140.16, 139.60, 138.24, 135.55, 130.69, 130.24, 129.71, 129.29, 128.67, 127.87, 127.36 (d, J = 3.3 Hz), 126.96, 126.17, 124.84, 124.50, 123.68, 43.96, 25.62, 22.99; HR-ESI-MS: *m*/*z* = 358.1443 [M + H]^+^, calculated for C_23_H_20_NO_3_: 358.1438

General preparation of intermediate **I-3**. The aromatic cyanides (1.0 eq), hydroxylamine hydrochloride (1.5 eq), and sodium carbonate (2.0 eq) were added into 20 mL of a mixed solvent of ethanol: H_2_O (4:1). The mixture was stirred at 60 °C for 6 h. The solvent was then evaporated in a vacuum and the residue was purified by column chromatography to provide intermediate **I-3**.

General preparation of compounds **B1**–**B25**. The diphenic anhydride (1.0 eq) and intermediate **I-3** (1.0 eq) were mixed in 20 mL dimethyl sulfoxide. The mixture was stirred at room temperature for 2 h, then sodium hydroxide (2.0 eq) was added to the reaction solution, the mixture was stirred at room temperature for 1 h. The reaction mixture was poured into water (50 mL), 1 M HCl solution was added dropwise to separated water phase to adjust the pH to 5, and the solid precipitates were filtered. The residue was purified by column chromatography to afford **B1**–**B25**.

Compound **B1**: Yield 55%; white solid; mp: 150–153 °C; ^1^H NMR (400 MHz, DMSO-d_6_) δ12.54 (s, 1H), 8.18 (d, *J* = 7.8 Hz, 1H), 7.96 (d, *J* = 7.7 Hz, 1H), 7.94–7.86 (m, 2H), 7.76–7.67 (m, 1H), 7.68–7.59 (m, 2H), 7.55 (t, *J* = 7.6 Hz, 1H), 7.38 (t, *J* = 8.9 Hz, 3H), 7.28 (d, *J* = 7.5 Hz, 1H); ^13^C NMR (101 MHz, DMSO-d_6_) δ 175.95, 167.61, 166.67, 163.92 (d, *J* = 249.1 Hz), 142.53, 141.07, 132.28, 131.58, 130.77 (2C), 130.71, 129.69 (2C), 129.44, 129.35, 129.25, 127.85 (d, *J* = 13.9 Hz, 2C), 122.66 (d, *J* = 2.4 Hz), 122.33, 116.39 (d, *J* = 22.2 Hz, 2C); HR-MS(ESI) *m*/*z* = 361.0983 [M + H]^+^, calculated for C_21_H_14_FN_2_O_3_: 361.0983.

Compound **B2**: Yield 59%; white solid; mp: 173–175 °C; ^1^H NMR (400 MHz, DMSO-d_6_) δ12.56 (s, 1H), 8.20 (d, *J* = 7.3 Hz, 1H), 8.06 (d, *J* = 8.1 Hz, 2H), 7.97 (d, *J* = 7.5 Hz, 1H), 7.90 (d, *J* = 8.3 Hz, 2H), 7.72 (t, *J* = 7.5 Hz, 1H), 7.63 (t, *J* = 7.5 Hz, 2H), 7.55 (t, *J* = 7.5 Hz, 1H), 7.40 (d, *J* = 7.4 Hz, 1H), 7.29 (d, *J* = 7.1 Hz, 1H); ^13^C NMR (101MHz, DMSO-d_6_) δ 176.37, 167.64, 166.54, 142.59, 141.00, 132.43, 131.61, 131.39 (d, *J* = 32.1 Hz), 130.77, 130.75, 129.94, 129.73, 129.34, 127.90 (d, *J* = 15.6 Hz, 2C), 127.72 (3C), 126.21 (d, *J* = 3.5 Hz, 2C), 123.80 (d, *J* = 272.6 Hz), 122.22; HR-ESI-MS: *m*/*z* = 411.0946 [M + H]^+^, calculated for C_22_H_14_F_3_N_2_O_3_: 411.0951.

Compound **B3**: Yield 59%; white solid; mp: 154–156 °C; ^1^H NMR (400 MHz, DMSO-d_6_) δ12.55 (s, 1H), 8.18 (d, *J* = 7.8 Hz, 1H), 7.96 (d, *J* = 7.7 Hz, 1H), 7.86 (d, *J* = 8.7 Hz, 2H), 7.72 (t, *J* = 7.3 Hz, 1H), 7.66–7.59 (m, 4H), 7.55 (t, *J* = 7.4 Hz, 1H), 7.39 (d, *J* = 7.6 Hz, 1H), 7.28 (d, *J* = 7.5 Hz, 1H); ^13^C NMR (101 MHz, DMSO-d_6_) δ 176.09, 167.61, 166.68, 142.53, 141.03, 136.30, 132.32, 131.59, 130.77, 130.72 (2C), 129.71, 129.39 (2C), 129.29, 128.65 (2C), 127.94, 127.79, 124.94, 122.30; HR-ESI-MS: *m*/*z* = 377.0684 [M + H]^+^, calculated for C_21_H_14_ClN_2_O_3_: 377.0687.

Compound **B4**: Yield 51%; white solid; mp: 148–150 °C; ^1^H NMR (400 MHz, DMSO-d_6_) δ12.54 (s, 1H), 8.18 (d, *J* = 7.7 Hz, 1H), 7.95 (d, *J* = 7.6 Hz, 1H), 7.79 (d, *J* = 8.5 Hz, 2H), 7.74 (d, *J* = 8.5 Hz, 2H), 7.70 (d, *J* = 7.5 Hz, 1H), 7.62 (t, *J* = 7.2 Hz, 2H), 7.55 (t, *J* = 7.5 Hz, 1H), 7.39 (d, *J* = 7.5 Hz, 1H), 7.28 (d, *J* = 7.4 Hz, 1H); ^13^C NMR (101 MHz, DMSO-d_6_) δ176.10, 167.61, 166.79, 142.53, 141.02, 132.31 (3C), 131.59, 130.77, 130.72, 129.71, 129.30, 128.81 (3C), 127.94, 127.79, 125.28, 125.16, 122.29; HR-ESI-MS: *m*/*z* = 421.0187 [M + H]^+^, calculated for C_21_H_14_BrN_2_O_3_: 421.0182.

Compound **B5**: Yield 51%; white solid; mp: 154–157 °C; ^1^H NMR (400 MHz, DMSO-d_6_) δ12.53 (s, 1H), 8.17 (dd, *J* = 1.2, 7.8 Hz, 1H), 7.97 (dd, *J* = 1.3, 7.7 Hz, 1H), 7.83–7.75 (m, 2H), 7.70 (td, *J* = 1.3, 7.6 Hz, 1H), 7.65–7.58 (m, 2H), 7.55 (td, *J* = 1.3, 7.6 Hz, 1H), 7.38 (dd, *J* = 1.1, 7.6 Hz, 1H), 7.27 (dd, *J* = 1.2, 7.5 Hz, 1H), 7.10–7.02 (m, 2H), 3.81(s, 3H); ^13^C NMR (101 MHz, DMSO-d_6_) δ 175.50, 167.61, 167.18, 161.68, 142.52, 141.21, 132.10, 131.55, 130.76 (2C), 130.66, 129.71, 129.18, 128.57 (2C), 127.87, 127.73, 122.49, 118.38, 114.58 (2C), 55.38; HR-ESI-MS: *m*/*z* = 373.1179 [M + H]^+^, calculated for C_22_H_17_N_2_O_4_: 373.1183.

Compound **B6**: Yield 60%; white solid; mp: 151–153 °C; ^1^H NMR (400 MHz, DMSO-d_6_) δ12.52 (s, 1H), 8.18 (d, *J* = 7.7 Hz, 1H), 7.96 (d, *J* = 7.6 Hz, 1H), 7.75 (d, *J* = 8.0 Hz, 2H), 7.71 (t, *J* = 7.5 Hz, 1H), 7.67–7.59 (m, 2H), 7.55 (t, *J* = 7.5 Hz, 1H), 7.38 (d, *J* = 7.6 Hz, 1H), 7.33 (d, *J* = 8.0 Hz, 2H), 7.28 (d, *J* = 7.5 Hz, 1H), 2.36 (s, 3H); ^13^C NMR (101 MHz, DMSO-d_6_) δ 175.69, 167.60, 167.43, 142.52, 141.48, 141.17, 132.15, 131.56, 130.77, 130.74, 130.67, 129.71 (3C), 129.21, 127.88, 127.75, 126.83 (2C), 123.33, 122.47, 21.06; HR-ESI-MS: *m*/*z* = 357.1229 [M + H]^+^, calculated for C_22_H_17_N_2_O_3_: 357.1234.

Compound **B7**: Yield 55%; white solid; mp: 119–121 °C; ^1^H NMR (400 MHz, DMSO-d_6_) δ12.55 (s, 1H), 8.19 (d, *J* = 7.8 Hz, 1H), 8.00–7.93 (m, 2H), 7.86 (d, *J* = 7.8 Hz, 1H), 7.81–7.75 (m, 1H), 7.72 (td, *J* = 1.2, 7.6 Hz, 1H), 7.67–7.59 (m, 2H), 7.56 (td, *J* = 1.1, 7.6 Hz, 1H), 7.49 (t, *J* = 7.9 Hz, 1H), 7.40 (d, *J* = 7.6 Hz, 1H), 7.29 (d, *J* = 7.5 Hz, 1H); ^13^C NMR(101 MHz, DMSO-d_6_) δ 176.05, 167.61, 166.31, 142.61, 141.06, 134.30, 132.40, 131.60, 131.47, 130.78 (2C), 130.69, 129.65, 129.48, 129.25, 128.23, 127.90, 127.81, 125.75, 122.27, 122.17; HR-ESI-MS: *m*/*z* = 421.0177 [M + H]^+^, calculated for C_21_H_14_BrN_2_O_3_: 421.0182.

Compound **B8**: Yield 58%; white solid; mp: 171–175 °C; ^1^H NMR (400 MHz, DMSO-d_6_) δ12.56 (s, 1H), 8.15 (d, *J* = 7.7 Hz, 1H), 7.94 (d, *J* = 9.2 Hz, 2H), 7.75–7.67 (m, 1H), 7.65 –7.58 (m, 2H), 7.58–7.50 (m, 1H), 7.38 (d, *J* = 7.5 Hz, 1H), 7.27 (d, *J* = 7.5 Hz, 1H), 7.06 (d, *J* = 3.4 Hz, 1H), 6.77–6.67 (m, 1H); ^13^C NMR (101 MHz, DMSO-d_6_) δ175.83, 167.60, 160.49, 146.43, 142.48, 141.39, 140.93, 132.33, 131.59, 130.78, 130.72, 130.67, 129.76, 129.38, 127.96, 127.79, 122.25, 114.34, 112.22; HR-ESI-MS: *m*/*z* = 333.0869 [M + H]^+^, calculated for C_19_H_13_N_2_O_4_: 333.0870.

Compound **B9**: Yield 58%; white solid; mp: 140–142 °C; ^1^H NMR (400 MHz, DMSO-d_6_) δ12.54 (s, 1H), 8.16 (d, *J* = 7.5 Hz, 1H), 7.96 (d, *J* = 7.7 Hz, 1H), 7.84 (d, *J* = 4.9 Hz, 1H), 7.71 (t, *J* = 7.6 Hz, 1H), 7.67–7.58 (m, 3H), 7.54 (t, *J* = 7.4 Hz, 1H), 7.38 (d, *J* = 7.5 Hz, 1H), 7.28 (d, *J* = 7.4 Hz, 1H), 7.23 (t, *J* = 4.4 Hz, 1H); ^13^C NMR (101 MHz, DMSO-d_6_) δ175.77, 167.59, 163.64, 142.56, 141.02, 132.32, 131.58, 130.76 (2C), 130.69, 130.67, 129.79, 129.75, 129.29, 128.45, 127.93, 127.78, 127.34, 122.21; HR-ESI-MS: *m*/*z* = 349.0635 [M + H]^+^, calculated for C_19_H_13_N_2_O_3_S: 349.0641.

Compound **B10**: Yield 50%; white solid; mp: 146–148 °C; ^1^H NMR (400 MHz, DMSO-d_6_) δ12.53 (s, 1H), 8.16 (d, *J* = 7.7 Hz, 1H), 8.10 (d, *J* = 2.8 Hz, 1H), 7.95 (d, *J* = 7.5 Hz, 1H), 7.74 (dd, *J* = 3.0, 5.0 Hz, 1H), 7.70 (t, *J* = 7.6 Hz, 1H), 7.62 (t, *J* = 7.4 Hz, 2H), 7.54 (t, *J* =7.6 Hz, 1H), 7.45 (d, *J* = 5.0 Hz, 1H), 7.38 (d, *J* = 7.5 Hz, 1H), 7.27 (d, *J* = 7.3 Hz, 1H);^13^C NMR (101 MHz, DMSO-d_6_) δ 175.61, 167.61, 164.11, 142.51, 141.09, 132.18, 131.55, 130.77 (2C), 130.67, 129.72, 129.25, 128.63, 128.37, 127.92, 127.74, 127.45, 125.55, 122.41; HR-ESI-MS: *m*/*z* = 349.0636 [M + H]^+^, calculated for C_19_H_13_N_2_O_3_S: 349.0641.

Compound **B11**: Yield 58%; white solid; mp: 167–169 °C; ^1^H NMR (400 MHz, DMSO-d_6_) δ12.59 (s, 1H), 9.38 (d, *J* = 1.4 Hz, 1H), 9.07 (d, *J* = 5.1 Hz, 1H), 8.21 (dd, *J* = 7.8, 1.4 Hz, 1H), 7.96 (dd, *J* = 7.7, 1.5 Hz, 1H), 7.90 (dd, *J* = 5.1, 1.4 Hz, 1H), 7.74 (td, *J* = 7.6, 1.4 Hz, 1H), 7.64 (tdd, *J* = 7.5, 2.5, 1.5 Hz, 2H), 7.56 (td, *J* = 7.6, 1.4 Hz, 1H), 7.40 (dd, *J* = 7.7, 1.3 Hz, 1H), 7.30 (dd, *J* = 7.6, 1.4 Hz, 1H); ^13^C NMR (176 MHz, DMSO-d_6_) δ177.47, 168.14, 166.91, 159.90, 159.60, 153.03, 142.99, 141.29, 133.09, 132.16, 131.28, 131.16, 130.24, 129.98, 128.56, 128.39, 122.60, 120.13; HR-ESI-MS: *m*/*z* = 345.0996 [M + H]^+^, calculated for C_19_H_13_N_4_O_3_: 345.0982.

Compound **B12**: Yield 50%; white solid; mp: 190–193 °C; ^1^H NMR (400 MHz, DMSO-d_6_) δ12.56 (s, 1H), 8.76–8.70 (m, 1H), 8.20 (d, *J* = 7.8 Hz, 1H), 8.03–7.93 (m, 2H), 7.86 (dd, *J* = 0.8, 8.0 Hz, 1H), 7.72 (t, *J* = 7.5 Hz, 1H), 7.63 (t, *J* = 7.4 Hz, 2H), 7.60–7.52 (m, 2H), 7.39 (d, *J* = 7.6 Hz, 1H), 7.30 (d, *J* = 7.5 Hz, 1H); ^13^C NMR (101 MHz, DMSO-d_6_) δ176.24, 167.64, 167.53, 150.27 (2C), 145.69, 142.47, 141.03, 137.62, 132.28, 131.61, 130.80, 130.74 (2C), 129.76, 129.37, 127.96, 127.81, 126.07, 123.18, 122.45; HR-ESI-MS: *m*/*z* = 344.1026 [M + H]^+^, cal calculated cd for C_20_H_14_N_3_O_3_: 344.1030.

Compound **B13**: Yield 52%; white solid; mp: 210–212 °C; ^1^H NMR (400 MHz, DMSO-d_6_) δ12.56 (s, 1H), 9.00(s, 1H), 8.75 (s, 1H), 8.20 (d, *J* = 7.5 Hz, 2H), 7.97 (d, *J* = 7.7 Hz, 1H), 7.72 (t, *J* = 7.5Hz, 1H), 7.63 (t, *J* = 7.3 Hz, 2H), 7.60–7.52 (m, 2H), 7.40 (d, *J* = 7.5 Hz, 1H), 7.29 (d, *J* = 7.4 Hz, 1H); ^13^C NMR (176 MHz, DMSO-d_6_) δ175.58, 167.04, 165.16, 151.74, 147.08, 142.02, 140.42, 133.78, 131.86, 131.03, 130.19, 130.18, 130.14, 129.09, 128.70, 127.37, 127.24, 123.69, 121.74, 121.53; HR-ESI-MS: *m*/*z* = 344.1028 [M + H]^+^, calculated for C_20_H_14_N_3_O_3_: 344.1030.

Compound **B14**: Yield 65%; white solid; mp: 188–191 °C; ^1^H NMR (400 MHz, DMSO-d_6_) δ12.56 (s, 1H), 8.80 –8.75 (m, 2H), 8.20 (dd, *J* = 1.3, 7.8 Hz, 1H), 7.96 (dd, *J* = 1.4, 7.7 Hz, 1H), 7.81–7.76(m, 2H), 7.73 (td, *J* = 1.4, 7.6 Hz, 1H), 7.68–7.61 (m, 2H), 7.56 (td, *J* = 1.3, 7.6 Hz, 1H), 7.41 (dd, *J* = 1.2, 7.6 Hz, 1H), 7.30 (dd, *J* = 1.2, 7.5 Hz, 1H); ^13^C NMR (176 MHz, DMSO-d_6_) δ176.00, 167.03, 165.58, 150.25, 141.99, 140.31, 132.72, 131.93, 131.04, 130.19, 130.16, 130.14, 129.11, 128.76, 127.41, 127.26, 121.50, 120.18; HR-ESI-MS: *m*/*z* = 344.1027 [M + H]^+^, calculated for C_20_H_14_N_3_O_3_: 344.1030.

Compound **B15**: Yield 39%; white solid; mp: 165–168 °C; ^1^H NMR (400 MHz, DMSO-d_6_) δ12.57 (s, 1H), 8.59 (d, *J* = 5.0 Hz, 1H), 8.21 (d, *J* = 7.7 Hz, 1H), 7.96 (d, *J* = 7.7 Hz, 1H), 7.93 (s, 1H), 7.84 (d, *J* = 5.1 Hz, 1H), 7.74 (t, *J* = 7.5 Hz, 1H), 7.64 (t, *J* = 7.4 Hz, 2H), 7.56 (t, *J* = 7.1 Hz, 1H), 7.41 (d, *J* = 7.6 Hz, 1H), 7.29 (d, *J* = 7.5 Hz, 1H); ^13^C NMR (101 MHz, DMSO-d_6_) δ 176.72, 167.63, 165.05, 151.80 (2C), 142.67, 142.21, 140.88, 136.32, 132.68, 131.65, 130.79 (2C), 129.65, 129.37, 127.98, 127.88, 124.98, 121.88, 120.36; HR-ESI-MS: *m*/*z* = 422.0133 [M + H]^+^, calculated for C_20_H_13_BrN_3_O_3_: 422.0135.

Compound **B16**: Yield 61%; white solid; mp: 145–147 °C; ^1^H NMR (400 MHz, DMSO-d_6_) δ12.57 (s, 1H), 8.69–8.59 (m, 1H), 8.22 (dd, *J* = 7.8, 1.3 Hz, 1H), 7.96 (dd, *J* = 7.8, 1.4 Hz, 1H), 7.84–7.79 (m, 2H), 7.74 (td, *J* = 7.6, 1.4 Hz, 1H), 7.64 (tt, *J* = 7.5, 1.4 Hz, 2H), 7.56 (td, *J* = 7.6, 1.4 Hz, 1H), 7.41 (dd, *J* = 7.7, 1.2 Hz, 1H), 7.30 (dd, *J* = 7.6, 1.3 Hz, 1H).; ^13^C NMR (176 MHz, DMSO-d_6_) δ 176.16, 167.04, 164.61, 150.80, 150.71, 142.07, 140.27, 136.13, 132.09, 131.07, 130.19, 130.18, 129.07, 128.79, 127.41, 127.29, 121.28, 120.75, 119.57; HR-ESI-MS: *m*/*z* = 378.0712 [M + H]^+^, calculated for C_20_H_13_ClN_3_O_3_: 378.0640.

Compound **B17**: Yield 61%; white solid; mp: 98–100 °C; ^1^H NMR (400 MHz, DMSO-d_6_) δ12.57 (s, 1H), 8.46 (d, *J* = 5.1 Hz, 1H), 8.21 (dd, *J* = 7.7, 1.3 Hz, 1H), 7.96 (dd, *J* = 7.6, 1.4 Hz, 1H), 7.78–7.70 (m, 2H), 7.68–7.60 (m, 2H), 7.56 (td, *J* = 7.6, 1.4 Hz, 1H), 7.51 (s, 1H), 7.41 (dd, *J* = 7.6, 1.4 Hz, 1H), 7.30 (dd, *J* = 7.6, 1.3 Hz, 1H); ^13^C NMR (176 MHz, DMSO-d_6_) δ 176.18, 167.04, 164.75 (d, *J* = 3.9 Hz), 162.89 (d, *J* = 236.0 Hz), 148.80, 148.72, 142.06, 140.25, 138.39 (d, *J* = 8.7 Hz), 132.08, 131.06, 130.19, 129.08, 128.79, 127.43, 127.29, 121.31, 118.71 (d, *J* = 4.2 Hz), 106.65, 106.42; HR-ESI-MS: *m*/*z* =362.0945 [M + H]^+^, calculated for C_20_H_13_FN_3_O_3_: 362.0935.

Compound **B18**: Yield 61%; white solid; mp: 228–230 °C; ^1^H NMR (400 MHz, DMSO-d_6_) δ12.56 (s, 1H), 8.63 (dd, *J* = 5.2, 0.9 Hz, 1H), 8.20 (dd, *J* = 7.8, 1.4 Hz, 1H), 7.96 (dd, *J* = 7.8, 1.5 Hz, 1H), 7.73 (td, *J* = 7.6, 1.4 Hz, 1H), 7.68–7.60 (m, 3H), 7.60–7.52 (m, 2H), 7.40 (dd, *J* = 7.6, 1.3 Hz, 1H), 7.29 (dd, *J* = 7.5, 1.4 Hz, 1H), 2.55 (s, 3H); ^13^C NMR (176 MHz, DMSO-d6) δ175.90, 167.07, 165.69, 158.77, 149.57, 142.03, 140.33, 132.95, 131.89, 130.99, 130.26, 130.17, 130.13, 129.11, 128.73, 127.36, 127.23, 121.55, 119.49, 117.31, 23.45; HR-ESI-MS: *m*/*z* = 358.1198 [M + H]^+^, calculated for C_21_H_16_N_3_O_3_: 358.1186.

Compound **B19**: Yield 57%; white solid; mp: 154–156 °C; ^1^H NMR (400 MHz, DMSO-d_6_) δ12.54 (s, 1H), 8.34 (d, *J* = 5.3 Hz, 1H), 8.19 (dd, *J* = 7.8, 1.4 Hz, 1H), 7.96 (dd, *J* = 7.7, 1.5 Hz, 1H), 7.73 (td, *J* = 7.6, 1.4 Hz, 1H), 7.63 (td, *J* = 7.5, 1.5 Hz, 2H), 7.56 (td, *J* = 7.6, 1.4 Hz, 1H), 7.42–7.37 (m, 2H), 7.29 (dd, *J* = 7.6, 1.3 Hz, 1H), 7.12 (t, *J* = 1.1 Hz, 1H), 3.90 (s, 3H); ^13^C NMR (176 MHz, DMSO-d6) δ 175.81, 167.03, 165.42, 163.61, 147.84 (d, *J* = 2.4 Hz), 142.05, 140.37, 135.70, 131.93, 131.03, 130.19, 130.18, 130.15, 129.07, 128.69, 127.38, 127.25, 121.42, 113.42, 107.36, 53.01; HR-ESI-MS: *m*/*z* = 374.1152 [M + H]^+^, calculated for C_21_H_16_N_3_O_4_: 374.1135.

Compound **B20**: Yield 57%; white solid; mp: 214–216 °C; ^1^H NMR (400 MHz, DMSO-d_6_) δ12.55 (s, 1H), 8.19 (dd, *J* = 7.8, 1.4 Hz, 1H), 7.96 (dd, *J* = 7.7, 1.4 Hz, 1H), 7.73 (td, *J* = 7.6, 1.4 Hz, 1H), 7.63 (tdd, *J* = 7.6, 2.7, 1.4 Hz, 2H), 7.56 (td, *J* = 7.6, 1.4 Hz, 1H), 7.45 (s, 2H), 7.40 (dd, *J* = 7.7, 1.3 Hz, 1H), 7.29 (dd, *J* = 7.5, 1.3 Hz, 1H), 2.49 (s, 6H); ^13^C NMR (176 MHz, DMSO-d_6_) δ176.88, 168.19, 166.81, 159.16, 142.96, 141.41, 134.47, 133.05, 132.23, 131.23, 131.10, 130.15, 129.77, 128.49, 128.37, 122.56, 117.81, 24.24(2C); HR-ESI-MS: *m*/*z* = 372.1361 [M + H]^+^, calculated for C_22_H_18_N_3_O_3_: 372.1343.

Compound **B21**: Yield 59%; white solid; mp: 233–235 °C; ^1^H NMR (400 MHz, DMSO-d_6_) δ12.57 (s, 1H), 8.85 (d, *J* = 0.6 Hz, 1H), 8.70 (d, *J* = 5.0 Hz, 1H), 8.20 (dd, *J* = 7.9, 1.3 Hz, 1H), 7.96 (dd, *J* = 7.8, 1.4 Hz, 1H), 7.77 (dd, *J* = 5.0, 0.6 Hz, 1H), 7.74 (td, *J* = 7.6, 1.5 Hz, 1H), 7.64 (tdd, *J* = 7.5, 3.8, 1.4 Hz, 2H), 7.55 (td, *J* = 7.6, 1.4 Hz, 1H), 7.40 (dd, *J* = 7.7, 1.3 Hz, 1H), 7.30 (dd, *J* = 7.6, 1.3 Hz, 1H).; ^13^C NMR (176 MHz, DMSO-d_6_) δ175.33, 166.99, 164.27, 150.18, 148.02, 142.05, 140.35, 131.98, 131.69, 131.08, 130.20, 130.18, 130.01, 129.23, 128.79, 128.41, 127.38, 127.28, 123.89, 121.36; HR-ESI-MS: *m*/*z* = 378.0698 [M + H]^+^, calculated for C_20_H_13_ClN_3_O_3_: 378.0640.

Compound **B22**: Yield 58%; white solid; mp: 258–259 °C; ^1^H NMR (400 MHz, DMSO-d_6_) δ12.56 (s, 1H), 9.06 (d, *J* = 4.4 Hz, 1H), 8.42 (dd, *J* = 8.6, 1.4 Hz, 1H), 8.25 (dd, *J* = 7.8, 1.4 Hz, 1H), 8.14 (dd, *J* = 8.5, 1.3 Hz, 1H), 8.00 (dd, *J* = 7.8, 1.5 Hz, 1H), 7.96 (d, *J* = 4.4 Hz, 1H), 7.87 (ddd, *J* = 8.4, 6.8, 1.4 Hz, 1H), 7.76 (td, *J* = 7.5, 1.4 Hz, 1H), 7.72–7.64 (m, 3H), 7.61 (td, *J* = 7.5, 1.4 Hz, 1H), 7.43 (dd, *J* = 7.7, 1.3 Hz, 1H), 7.36 (dd, *J* = 7.5, 1.4 Hz, 1H); ^13^C NMR (176 MHz, DMSO-d_6_) δ176.23, 168.19, 167.29, 150.82, 148.72, 143.09, 141.47, 133.05, 132.26, 131.33, 131.30, 131.15, 131.12, 130.65, 130.32, 130.18, 129.82, 128.61, 128.51, 128.44, 126.14, 124.37, 122.57, 122.28; HR-ESI-MS: *m*/*z* = 394.1208 [M + H]^+^, calculated for C_24_H_16_N_3_O_3_: 394.1186.

Compound **B23**: Yield 52%; white solid; mp: 236–238 °C; ^1^H NMR (400 MHz, DMSO-d_6_) δ12.57 (s, 1H), 9.27 (d, *J* = 2.1 Hz, 1H), 8.90 (dd, *J* = 2.2, 0.8 Hz, 1H), 8.26 (dd, *J* = 7.8, 1.4 Hz, 1H), 8.17 (dd, *J* = 8.3, 1.5 Hz, 1H), 8.11 (dd, *J* = 8.4, 1.1 Hz, 1H), 8.00 (dd, *J* = 7.7, 1.5 Hz, 1H), 7.90 (ddd, *J* = 8.4, 6.9, 1.5 Hz, 1H), 7.74 (qd, *J* = 7.2, 1.3 Hz, 2H), 7.66 (td, *J* = 7.7, 1.4 Hz, 2H), 7.59 (td, *J* = 7.6, 1.4 Hz, 1H), 7.43 (dd, *J* = 7.6, 1.3 Hz, 1H), 7.32 (dd, *J* = 7.5, 1.4 Hz, 1H); ^13^C NMR (176 MHz, DMSO-d_6_) δ175.64, 165.27, 147.91, 147.12, 134.47, 131.86, 131.05, 130.90, 130.19, 130.16, 129.12, 128.75, 128.47, 128.33, 127.38, 127.20, 126.26, 121.59, 118.88; HR-ESI-MS: *m*/*z* = 394.1180 [M + H]^+^, calculated for C_24_H_16_N_3_O_3_: 394.1186.

Compound **B24**: Yield 51%; white solid; mp: 264–266 °C; ^1^H NMR (400 MHz, DMSO-d_6_) δ12.57 (s, 1H), 9.00 (dd, *J* = 4.2, 1.7 Hz, 1H), 8.57 (d, *J* = 1.8 Hz, 1H), 8.53 (dd, *J* = 8.3, 1.7 Hz, 1H), 8.25 (dd, *J* = 7.9, 1.4 Hz, 1H), 8.20–8.11 (m, 2H), 8.00 (dd, *J* = 7.8, 1.5 Hz, 1H), 7.73 (dd, *J* = 7.6, 1.4 Hz, 1H), 7.69–7.62 (m, 3H), 7.59 (td, *J* = 7.6, 1.4 Hz, 1H), 7.42 (dd, *J* = 7.7, 1.3 Hz, 1H), 7.32 (dd, *J* = 7.5, 1.4 Hz, 1H); ^13^C NMR (176 MHz, DMSO-d_6_) δ176.67, 168.16, 167.59, 152.73, 149.09, 143.04, 141.55, 137.48, 132.87, 132.17, 131.29, 131.23, 131.19, 130.57, 130.25, 129.83, 128.50, 128.32, 128.27, 128.16, 127.38, 124.48, 123.02, 122.83; HR-ESI-MS: *m*/*z* = 394.1205 [M + H]^+^, calculated for C_24_H_16_N_3_O_3_: 394.1186.

Compound **B25**: Yield 51%; white solid; mp: 169–171 °C; ^1^H NMR (400 MHz, DMSO-d_6_) δ12.55 (s, 1H), 8.60–8.45 (m, 1H), 8.29–8.24 (m, 1H), 8.22 (dd, *J* = 7.8, 1.4 Hz, 1H), 8.07–7.96 (m, 2H), 7.73 (td, *J* = 7.5, 1.4 Hz, 1H), 7.69–7.54 (m, 5H), 7.41 (dd, *J* = 7.6, 1.3 Hz, 1H), 7.34 (dd, *J* = 7.5, 1.4 Hz, 1H), 7.10 (d, *J* = 8.3 Hz, 1H), 4.05 (s, 3H); ^13^C NMR (176 MHz, DMSO-d_6_) δ174.95, 168.55, 168.16, 157.97, 143.00, 141.72, 132.65, 132.18, 131.29, 131.20, 131.19, 131.11, 130.84, 130.29, 129.65, 128.53, 128.39, 128.32, 126.39, 125.97, 125.39, 122.94, 122.50, 115.42, 104.52, 56.49; HR-ESI-MS: *m*/*z* = 421.1184 [M-H]^-^, calculated for C_26_H_17_N_2_O_4_: 421.1183.

The procedure for the synthesis of intermediate **I-4**. Compound **B21** (1.0 eq) and methyl 3-aminopropionate hydrochloride (1.2 eq) were dissolved in 20 mL *N*,*N*-dimethylformamide, then HATU (1.5 eq) and triethylamine (2 eq) were added. The mixture was stirred at 60 °C for 10 h, and the solvent was evaporated in a vacuum. The reaction mixture was poured into water (50 mL). The mixture was extracted with ethyl acetate (50 mL × 3). The combined organic solution was washed with saturated aqueous sodium chloride solution (30 mL × 3) and dried over anhydrous Na_2_SO_4_. The residue was purified by column chromatography to **I-4**.

The procedure for the synthesis of compound **B26**. The intermediate **I-4** (1.0 eq) and lithium hydroxide monohydrate (2.0 eq) were dissolved in 20 mL of a mixed solvent of ethanol: H_2_O (4:1). The mixture was stirred at room temperature for 8 h, and the solvent was evaporated in a vacuum. The reaction mixture was poured into water (50 mL). 1 M HCl solution was added dropwise to adjust the pH to 5. The mixture was extracted with ethyl acetate (50 mL × 3). The combined organic solution was washed with saturated aqueous sodium chloride solution (30 mL × 3) and dried over anhydrous Na_2_SO_4_. The residue was purified by column chromatography to **B26**.

Compound **B26**: Yield 53%; white solid; mp: 240–242 °C; ^1^H NMR (400 MHz, DMSO-d_6_) δ8.84 (s, 1H), 8.70 (d, *J* = 5.0 Hz, 1H), 8.14 (dd, *J* = 7.8, 1.4 Hz, 1H), 8.00 (s, 1H), 7.82 (d, *J* = 5.0 Hz, 1H), 7.70 (td, *J* = 7.6, 1.4 Hz, 1H), 7.61 (td, *J* = 7.6, 1.4 Hz, 1H), 7.54–7.44 (m, 3H), 7.39 (dd, *J* = 7.7, 1.3 Hz, 1H), 7.29 (dd, *J* = 7.8, 1.7 Hz, 1H), 3.03 (d, *J* = 7.0 Hz, 2H), 1.83 (t, *J* = 7.4 Hz, 2H); ^13^C NMR (176 MHz, DMSO-d6) δ176.54, 167.71, 165.22, 151.16, 149.12, 141.97, 138.92, 136.97, 132.92, 131.63, 130.70, 130.19, 129.86, 129.47, 128.47, 128.23, 127.46, 125.18, 122.89, 37.00, 36.91; HR-ESI-MS: *m*/*z* = 449.1032 [M + H]^+^, calculated for C_23_H_18_ClN_4_O_4_: 449.1011.

The procedure for the synthesis of compound **B27**. The compound **B21** (1.0 eq) and 4-bromobenzenesulfonamide (1.2 eq) were dissolved in 20 mL *N*, *N*-dimethylformamide in an ice bath, then 4-dimethylaminopyridine (1.5 eq) and EDCI (1.2 eq) were added. The mixture was stirred at room temperature for 6 h. The solvent was evaporated in a vacuum. The residue was purified by column chromatography to give **B27**.

Compound **B27**: Yield 49%; white solid; mp: 188–190 °C; ^1^H NMR (400 MHz, DMSO-d_6_) δ8.84 (d, *J* = 0.5 Hz, 1H), 8.70 (d, *J* = 5.0 Hz, 1H), 8.11–8.04 (m, 1H), 7.86 (dd, *J* = 4.9, 0.6 Hz, 1H), 7.82–7.77 (m, 1H), 7.60–7.46 (m, 2H), 7.42–7.30 (m, 6H), 7.21–7.14 (m, 1H), 7.07–7.00 (m, 1H); ^13^C NMR (176 MHz, DMSO-d_6_) δ175.62, 170.76, 163.81, 150.05, 147.93, 144.64, 143.32, 139.34, 138.44, 131.99, 131.26, 130.27, 129.59, 129.25, 128.62, 128.34, 128.17, 127.88, 127.51, 126.30, 126.15, 124.19, 122.23, 121.45; HR-ESI-MS: *m*/*z* = 594.9864 [M + H]^+^, calculated for C_26_H_17_BrClN_4_O_4_S: 594.9837.

The procedure for the synthesis of Intermediate **I-5a**. Iodine substituted benzoic acid (1.2 eq) and intermediate **I-3a** (1.0 eq) were dissolved in 20 mL dichloromethane, then HATU (1.5 eq) and triethylamine (2 eq) were added. The mixture was stirred at room temperature for 10 h, and the solvent was evaporated in a vacuum. The residue was purified by column chromatography to give intermediate **I-5a**.

The procedure for the synthesis of intermediate **I-6a**. Intermediate **I-5a** (1.0 eq) and cesium carbonate (1.0 eq) were dissolved in 20 mL dimethyl sulfoxide. The mixture was stirred at room temperature for 10 h, the reaction mixture was poured into water (50 mL), the mixture was extracted with ethyl acetate (50 mL × 3). The combined organic solution was washed with saturated aqueous sodium chloride solution (30 mL × 3) and dried over anhydrous Na_2_SO_4_. The residue was purified by column chromatography to intermediate **I-6a**.

General preparation of compounds **B28** and **B29**. The intermediate **I-6a** (1.0 eq), substituted phenylboronic acids (1.0 eq), potassium carbonate (3.0 eq), and tetrakis(triphenylphosphine)palladium (0.2 eq) were added into 20 mL of a mixed solvent of toluene: 95%ethanol (1:1). The mixture was stirred at 110 °C under N_2_ for 12 h. The solvent was then evaporated in a vacuum, the reaction mixture was poured into water (50 mL). 1 M HCl solution was added dropwise to adjust the pH to 5. The mixture was extracted with ethyl acetate (50 mL × 3). The combined organic solution was washed with saturated aqueous sodium chloride solution (30 mL × 3) and dried over anhydrous Na_2_SO_4_. The residue was purified by column chromatography to **B28** and **B29**.

Compound **B28**: Yield 55%; white solid; mp: 288–289 °C; ^1^H NMR (400 MHz, DMSO-d_6_) δ13.07 (s, 1H), 8.82–8.73 (m, 2H), 8.17 (dd, *J* = 7.8, 1.4 Hz, 1H), 7.98 (dt, *J* = 6.8, 1.9 Hz, 1H), 7.88–7.78 (m, 4H), 7.71 (td, *J* = 7.6, 1.3 Hz, 1H), 7.64 (dd, *J* = 7.7, 1.3 Hz, 1H), 7.60–7.52 (m, 2H); ^13^C NMR (176 MHz, DMSO-d_6_) δ176.20, 166.46, 165.81, 150.32(2C), 140.64, 139.18, 132.65, 132.52, 130.74, 130.23, 128.81, 128.09, 128.07, 128.02, 121.42, 120.26(2C); HR-ESI-MS: *m*/*z* = 344.1041 [M + H]^+^, calculated for C_20_H_14_N_3_O_3_: 344.1030.

Compound **B29**: Yield 54%; white solid; mp: 258–260 °C; ^1^H NMR (400 MHz, DMSO-d_6_) δ8.85–8.75 (m, 2H), 8.08 (dd, *J* = 7.8, 1.4 Hz, 1H), 7.94–7.89 (m, 2H), 7.86–7.82 (m, 2H), 7.78 (td, *J* = 7.6, 1.4 Hz, 1H), 7.65 (td, *J* = 7.6, 1.3 Hz, 1H), 7.60 (dd, *J* = 7.8, 1.2 Hz, 1H), 7.19–7.14 (m, 2H); ^13^C NMR (176 MHz, DMSO-d_6_) δ176.78, 167.92, 165.81, 150.30(2C), 141.97, 140.00, 138.65, 132.74, 132.23, 130.56, 130.22, 128.38(2C), 127.33, 126.77(2C), 121.56, 120.34(2C); HR-ESI-MS: *m*/*z* = 344.1043 [M + H]^+^, calculated for C_20_H_14_N_3_O_3_: 344.1030.

The procedure for the synthesis of intermediate **I-7**. The isoniazid (1.0 eq) and 2-bromobenzoic acid (1.05 eq) were dissolved in 20 mL phosphorus oxychloride. The mixture was stirred at 110 °C for 10 h. Saturated sodium bicarbonate solution was added dropwise until there were no bubbles in the reaction solution. The mixture was extracted with ethyl acetate (50 mL × 3). The combined organic solution was washed with saturated aqueous sodium chloride solution (30 mL × 3) and dried over anhydrous Na_2_SO_4_. The residue was purified by column chromatography to obtain **I-7**.

The procedure for the synthesis of intermediate **I-8**. The intermediate **I-7** (1.0 eq), substituted phenylboronic acid (1.0 eq), potassium carbonate (3.0 eq), and tetrakis(triphenylphosphine)palladium (0.2 eq) were added into 20 mL of a mixed solvent of toluene: 95%ethanol (1:1). The mixture was stirred at 110 °C under N_2_ for 12 h. The solvent was then evaporated in a vacuum and the residue was purified by column chromatography to intermediate **I-8**.

The procedure for the synthesis of compound **B30**. The intermediate **I-8** (1.0 eq) and lithium hydroxide monohydrate (2.0 eq) were dissolved in 20 mL of a mixed solvent of ethanol: H_2_O (4:1). The mixture was stirred at room temperature for 8 h, and the solvent was evaporated in a vacuum. The reaction mixture was poured into water (50 mL). 1 M HCl solution was added dropwise to adjust the pH to 5. The mixture was extracted with ethyl acetate (50 mL × 3). The combined organic solution was washed with saturated aqueous sodium chloride solution (30 mL × 3) and dried over anhydrous Na_2_SO_4_. The residue was purified by column chromatography to **B30**.

Compound **B30**: Yield 58%; white solid; mp: 237–239 °C; ^1^H NMR (400 MHz, DMSO-d_6_) δ12.60 (s, 1H), 8.80–8.67 (m, 2H), 8.19 (dt, *J* = 7.7, 1.8 Hz, 1H), 7.95 (dt, *J* = 7.7, 1.8 Hz, 1H), 7.72–7.56 (m, 4H), 7.51–7.46 (m, 2H), 7.40 (dd, *J* = 7.6, 1.4 Hz, 1H), 7.33 (dd, *J* = 7.6, 1.5 Hz, 1H); ^13^C NMR (176 MHz, DMSO-d_6_) δ168.24, 165.58, 162.46, 151.35, 142.00, 141.48, 132.12, 132.10, 131.36, 131.22, 131.10, 130.54, 130.11, 128.96, 128.45, 128.33, 122.26, 120.16; HR-ESI-MS: *m*/*z* = 344.1026 [M + H]^+^, calculated for C_20_H_14_N_3_O_3_: 344.1030.

Spectral data, including HR-ESI-MS, ^1^H-NMR and ^13^C-NMR, for compounds **A1**–**A21** and **B1**–**B30** are provided in Supplementary Materials.

### 3.2. Biology

In Vitro URAT1 Inhibitory Assay. A transient expression system of human URAT1 in HEK293T cells was constructed to measure ^14^C-uric acid transport. The HEK293T cells were obtained from ATCC (catalogue No.: CRL-11268). The cell cryovials were removed from liquid nitrogen storage, and immediately placed on dry ice prior to thawing. The frozen cell vials were placed briefly (30 s to 1 min) in a 37 °C water bath, until only small ice crystals left and the cell pellet was almost completely thawed. The cell suspension was transferred into a 15 mL centrifuge tube, and 10 mL complete medium (DMEM +10% FBS +500 μg/mL G418 +1% P/S) was added. After centrifugation at 1000 rpm for 5 min, the supernatant was discarded, and cell precipitation was resuspended in 5 mL complete medium and transferred to a T75 culture flask. 15 mL of the assay complete medium was added into a T75 flask and incubated at 37 °C and 5% CO_2_. The uptake assay test could be carried out after cell culture adherent to the well in the 96-well plate. Then, the cells were washed with 200 µL/well of pre-warmed Cl^-^ free HBSS buffer, and all buffer was removed at the last washing. 50 µL/well of Cl^-^ free HBSS Buffer containing Uric acid [8-^14^C] (2.5 μCi/mL) and samples were added to the cells, and incubated for 8 min. Then, the cells were washed three times with Cl^-^ free HBSS Buffer and all buffer was removed at the last washing. 50 µL/well of lysis buffer (100 mM NaOH) was added to the lysate cells and stirred at 900 rpm for 5 min. 150 µL/well MicroScint™-40 cocktail was added and agitated at 900 rpm for 5 min. Finally, the 96-plate was counted in a MicroBeta2 (PerkinElmer). The data were analyzed and IC_50_ values was calculated with GraphPad Prism 6 software.

The IC_50_ curves for the compounds are provided in Supplementary Materials.

## 4. Conclusions

Based on the known URAT1 inhibitors Epaminurad and Telmisartan, two series of biphenyl carboxylic acid-based URAT1 inhibitors were recognized, and SAR clues were provided. Both series afforded potent URAT1 inhibitors, and **A1** and **B21** exhibited IC_50_ values of 0.93 μM and 0.17 μM, respectively. These new inhibitors not only represent a new chemical prototype of potent URAT1 inhibitors, but are expected to have reduced toxicity and improved chemical accessibility compared to Epaminurad and Telmisartan. The results confirmed that ligand-based approaches could be an effective way to identify novel and potent URAT1 inhibitors.

## 5. Patents

A Chinese patent (202211251941.3) derived from results presented herein was applied in 2022.

## Figures and Tables

**Table 1:** Structures and URAT1 inhibitory activities of compounds **A1**–**A21**.

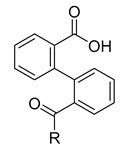							
Cmpd.	R	Inhibition% *	IC_50_ (μM)	Cmpd.	R	Inhibition% *	IC_50_ (μM)
**A1**	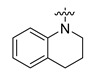	89	0.93	**A11**	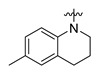	91	2.1
**A2**	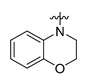	75	3.9	**A12**	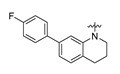	29	ND
**A3**	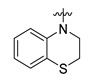	78	ND	**A13**	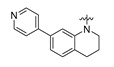	86	1.5
**A4**	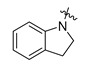	77	4.7	**A14**	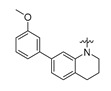	58	ND
**A5**	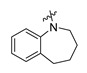	72	ND	**A15**	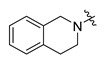	25	ND
**A6**	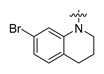	84	1.1	**A16**	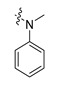	13	ND
**A7**	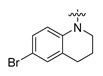	94	1.1	**A17**	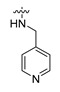	<10	ND
**A8**	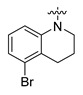	90	1.6	**A18**	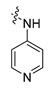	11	ND
**A9**	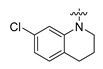	85	2.2	**A19**	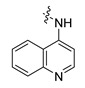	24	ND
**A10**	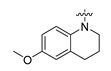	88	1.8				
**A20**	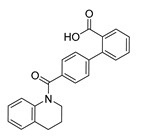					<10	ND
**A21**	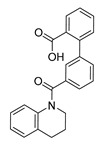					<10	ND
Benz	-					-	0.36

* The percentage inhibition was measured at the concentration of 10 μM and was represented as the mean of three measurements. ND: not determined. Benz: benzbromarone.

**Table 2:** Structures and URAT1 inhibitory activities of compounds **B1**–**B30**.

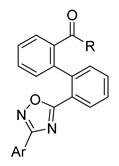				
Cmpd.	Ar	R	Inhibition% *	IC_50_ (μM)
**B1**	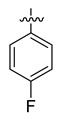	OH	56	ND
**B2**	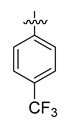	OH	39	ND
**B3**	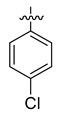	OH	55	ND
**B4**	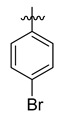	OH	61	ND
**B5**	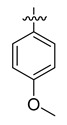	OH	68	ND
**B6**	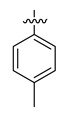	OH	45	ND
**B7**	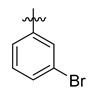	OH	44	ND
**B8**	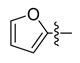	OH	56	ND
**B9**	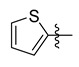	OH	68	ND
**B10**	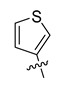	OH	69	ND
**B11**	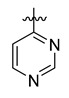	OH	30	ND
**B12**	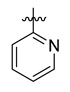	OH	27	ND
**B13**	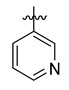	OH	64	ND
**B14**	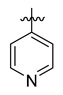	OH	88	1.8
**B15**	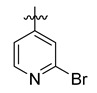	OH	97	0.25
**B16**	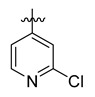	OH	97	0.27
**B17**	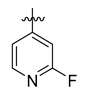	OH	90	0.98
**B18**	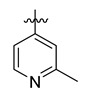	OH	97	0.28
**B19**	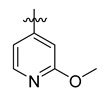	OH	96	0.42
**B20**	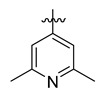	OH	76	3.1
**B21**	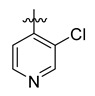	OH	98	0.17
**B22**	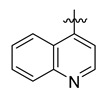	OH	89	1.1
**B23**	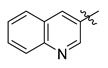	OH	74	6.1
**B24**	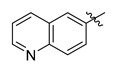	OH	67	ND
**B25**	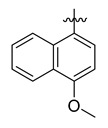	OH	42	ND
**B26**	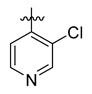	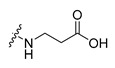	35	ND
**B27**	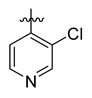	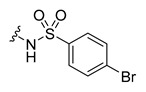	96	1.3
**B28**	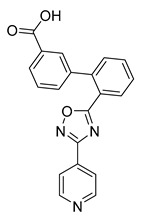		29	ND
**B29**	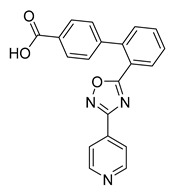		18	ND
**B30**	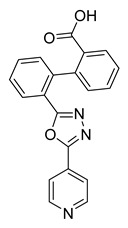		33	ND
Benz	-		-	0.36

* The percentage inhibition was measured at the concentration of 10 μM and was represented as the mean of three measurements. ND: not determined. Benz: benzbromarone.

## Data Availability

Not applicable.

## Supplementary Materials

The following supporting information can be downloaded at: https://www.mdpi.com/article/10.3390/molecules28217415/s1.

## References

[1] Dalbeth N., Gosling A.L., Gaffo A., Abhishek A. (2021). Gout. Lancet.

[2] Campion E.W., Glynn R.J., DeLabry L.O. (1987). Asymptomatic hyperuricemia. Risks and consequences in the normative aging study. Am. J. Med..

[3] Enomoto A., Kimura H., Chairoungdua A., Shigeta Y., Jutabha P., Cha S.H., Hosoyamada M., Takeda M., Sekine T., Igarashi T. (2002). Molecular identification of a renal urate-anion exchanger that regulates blood urate levels. Nature.

[4] Eraly S.A., Vallon V., Rieg T., Gangoiti J.A., Wikoff W.R., Siuzdak G., Barshop B.A., Nigam S.K. (2008). Multiple organic anion transporters contribute to net renal excretion of uric acid. Physiol. Genom..

[5] Nakanishi T., Ohya K., Shimada S., Anzai N., Tamai I. (2013). Functional cooperation of URAT1 (SLC22A12) and URATv1 (SLC2A9) in renal reabsorption of urate. Nephrol. Dial. Transpl..

[6] Nigam S.K. (2018). The SLC22 transporter family: A paradigm for the impact of drug transporters on metabolic pathways, signaling, and disease. Annu. Rev. Pharmacol. Toxicol..

[7] Vitart V., Rudan I., Hayward C., Gray N.K., Floyd J., Palmer C.N., Knott S.A., Kolcic I., Polasek O., Graessler J. (2008). SLC2A9 is a newly identified urate transporter influencing serum urate concentration, urate excretion and gout. Nat. Genet..

[8] Woodward O.M., Kottgen A., Coresh J., Boerwinkle E., Guggino W.B., Kottgen M. (2009). Identification of a urate transporter, ABCG2, with a common functional polymorphism causing gout. Proc. Natl. Acad. Sci. USA.

[9] Sattui S.E., Gaffo A.L. (2016). Treatment of hyperuricemia in gout: Current therapeutic options, latest developments and clinical implications. Ther. Adv. Musculoskelet. Dis..

[10] Song D., Zhao X., Wang F., Wang G. (2021). A brief review of urate transporter 1 (URAT1) inhibitors for the treatment of hyperuricemia and gout: Current therapeutic options and potential applications. Eur. J. Pharmacol..

[11] Stamp L.K., Merriman T.R., Singh J.A. (2018). Expert opinion on emerging urate-lowering therapies. Expert Opin. Emerg. Drugs.

[12] Shi X., Zhao T., da Silva-Junior E.F., Zhang J., Xu S., Gao S., Liu X., Zhan P. (2022). Novel urate transporter 1 (URAT1) inhibitors: A review of recent patent literature (2020-present). Expert. Opin. Ther. Pat..

[13] Ahn S.O., Ohtomo S., Kiyokawa J., Nakagawa T., Yamane M., Lee K.J., Kim K.H., Kim B.H., Tanaka J., Kawabe Y. (2016). Stronger uricosuric effects of the novel selective URAT1 inhibitor UR-1102 lowered plasma urate in tufted capuchin monkeys to a greater extent than benzbromarone. J. Pharmacol. Exp. Ther..

[14] Ohe T., Umezawa R., Kitagawara Y., Yasuda D., Takahashi K., Nakamura S., Abe A., Sekine S., Ito K., Okunushi K. (2018). Synthesis of novel benzbromarone derivatives designed to avoid metabolic activation. Bioorg. Med. Chem. Lett..

[15] Hoy S.M. (2016). Lesinurad: First Global Approval. Drugs.

[16] Yang C., Zhou D., Shen Z., Wilson D.M., Renner M., Miner J.N., Girardet J.L., Lee C.A. (2019). Characterization of stereoselective metabolism, inhibitory effect on uric acid uptake transporters, and pharmacokinetics of Lesinurad atropisomers. Drug Metab. Dispos..

[17] Kuriyama S. (2020). Dotinurad: A novel selective urate reabsorption inhibitor as a future therapeutic option for hyperuricemia. Clin. Exp. Nephrol..

[18] Uda J., Kobashi S., Miyata S., Ashizawa N., Matsumoto K., Iwanaga T. (2020). Discovery of Dotinurad (FYU-981), a new phenol derivative with highly potent uric acid lowering activity. ACS Med. Chem. Lett..

[19] Hosoya T., Sano T., Sasaki T., Fushimi M., Ohashi T. (2020). Dotinurad versus benzbromarone in Japanese hyperuricemic patient with or without gout: A randomized, double-blind, parallel-group, phase 3 study. Clin. Exp. Nephrol..

[20] Felser A., Lindinger P.W., Schnell D., Kratschmar D.V., Odermatt A., Mies S., Jeno P., Krahenbuhl S. (2014). Hepatocellular toxicity of benzbromarone: Effects on mitochondrial function and structure. Toxicology.

[21] Lee M.H., Graham G.G., Williams K.M., Day R.O. (2008). A benefit-risk assessment of benzbromarone in the treatment of gout. Was its withdrawal from the market in the best interest of patients?. Drug Saf..

[22] Zhao Z.A., Jiang Y., Li L., Chen Y.Y., Li Y.M., Lan Q.S., Wu T., Lin C.T., Cao Y., Nandakumar K.S. (2020). Structural insights into the atomistic mechanisms of uric acid recognition and translocation of human urate anion transporter 1. ACS Omega.

[23] Pan Y., Kong L.D. (2016). Urate transporter URAT1 inhibitors: A patent review (2012–2015). Expert. Opin. Ther. Pat..

[24] Otani N., Ouchi M., Kudo H., Tsuruoka S., Hisatome I., Anzai N. (2020). Recent approaches to gout drug discovery: An update. Expert Opin. Drug Discov..

[25] Dong Y., Zhao T., Ai W., Zalloum W.A., Kang D., Wu T., Liu X., Zhan P. (2019). Novel urate transporter 1 (URAT1) inhibitors: A review of recent patent literature (2016–2019). Expert Opin. Ther. Pat..

[26] Peng J., Hu Q., Gu C., Liu B., Jin F., Yuan J., Feng J., Zhang L., Lan J., Dong Q. (2016). Discovery of potent and orally bioavailable inhibitors of human uric acid transporter 1 (hURAT1) and binding mode prediction using homology model. Bioorg Med. Chem. Lett..

[27] Lin Y., Chen X., Ding H., Ye P., Gu J., Wang X., Jiang Z., Li D., Wang Z., Long W. (2021). Efficacy and safety of a selective URAT1 inhibitor SHR4640 in Chinese subjects with hyperuricaemia: A randomized controlled phase II study. Rheumatology.

[28] Tang H., Cui B., Chen Y., Chen L., Wang Z., Zhang N., Yang Y., Wang X., Xie X., Sun L. (2022). Safety and efficacy of SHR4640 combined with febuxostat for primary hyperuricemia: A multicenter, randomized, double-blind, phase II study. Ther. Adv. Musculoskelet. Dis..

[29] Ahn S.O., Horiba N., Ohtomo S., Lee K.J., Kim K.H., Kim B.H., Kiyokawa J., Yamane M., Ikegami H., Nakagawa T. (2013). The therapeutic efficacy of the novel uricosuric agent UR-1102 for hyperuricemia and gout. Ann. Rheum. Dis..

[30] Pascart T., Richette P. (2018). Investigational drugs for hyperuricemia, an update on recent developments. Expert Opin. Investig. Drugs.

[31] Pushpakom S., Iorio F., Eyers P.A., Escott K.J., Hopper S., Wells A., Doig A., Guilliams T., Latimer J., McNamee C. (2019). Drug repurposing: Progress, challenges and recommendations. Nat. Rev. Drug Discov..

[32] Nakamura M., Anzai N., Jutabha P., Sato H., Sakurai H., Ichida K. (2010). Concentration-dependent inhibitory effect of irbesartan on renal uric acid transporters. J. Pharmacol. Sci..

