# Supplementary material for: Discovery of Novel Biphenyl Carboxylic Acid Derivatives as Potent URAT1 Inhibitors
Source: Molecules. 2023 Nov 3;28(21):7415. doi: 10.3390/molecules28217415 (PMC10648204; doi:10.3390/molecules28217415)

Supplementary Material

Discovery of novel biphenyl carboxylic acid derivatives as potent URAT1 inhibitors

Xianxin Hou, Yongcheng Wang, Yajun Yang and Zhiyan Xiao*

Beijing Key Laboratory of Active Substance Discovery and Druggability Evaluation, Institute of Materia Medica, Chinese Academy of Medical Sciences and Peking Union Medical College, Beijing 100050, China

*Correspondence: xiaoz@imm.ac.cn

Spectral data, including HR-ESI-MS, ^{1}H-NMR and ^{13}C-NMR, for the target compounds are provided herein.

Compound **A1**:

HR-ESI-MS:

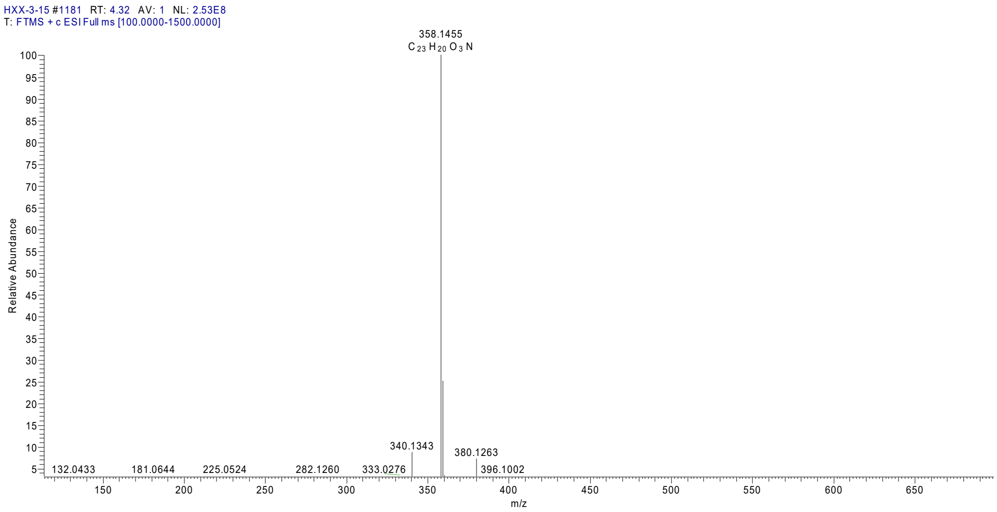

^{1}H-NMR:

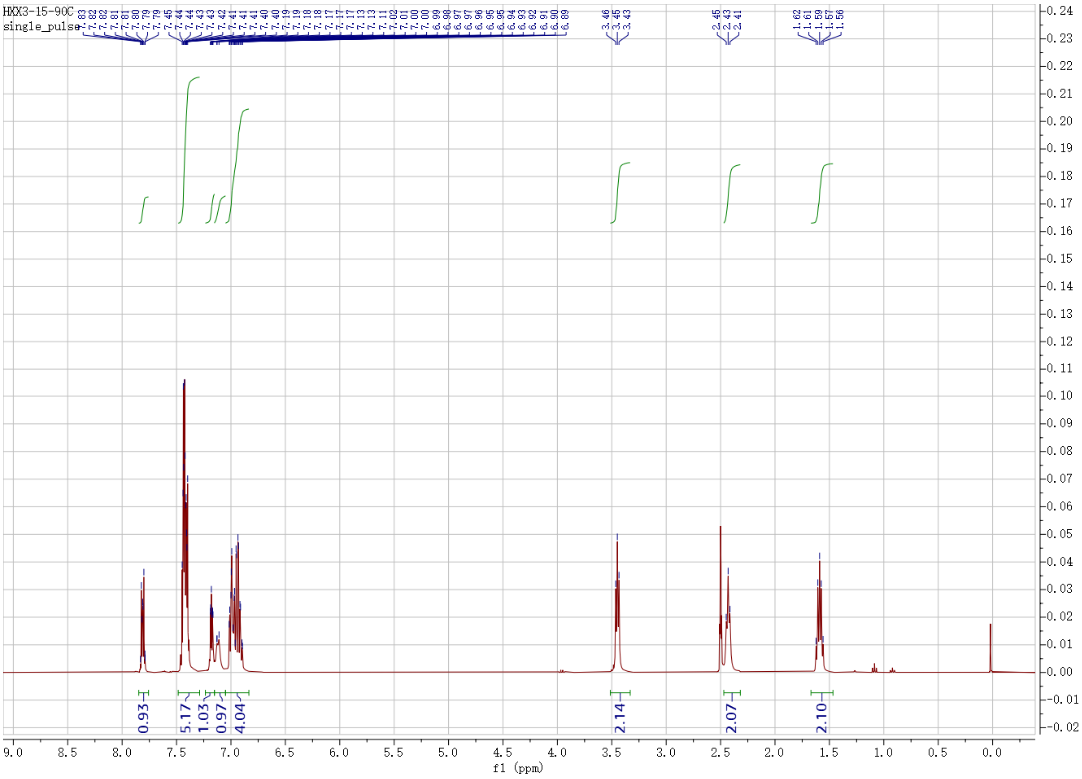

^{13}C-NMR:

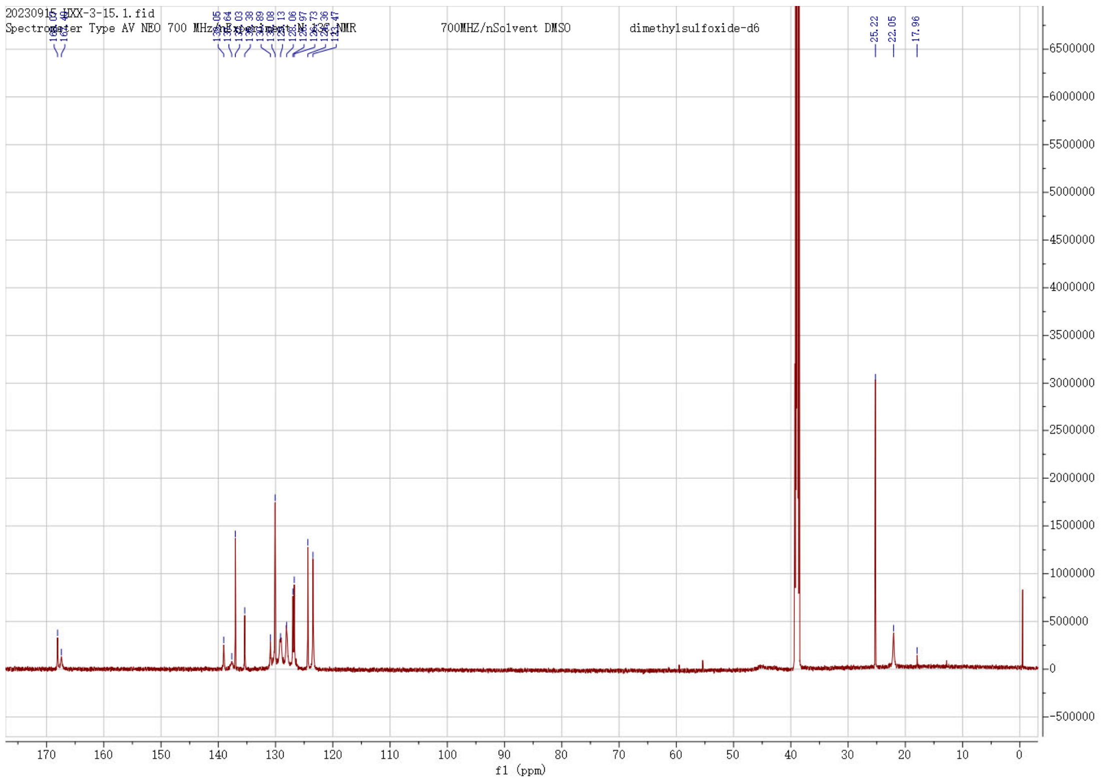

Compound **A2**:

HR-ESI-MS:

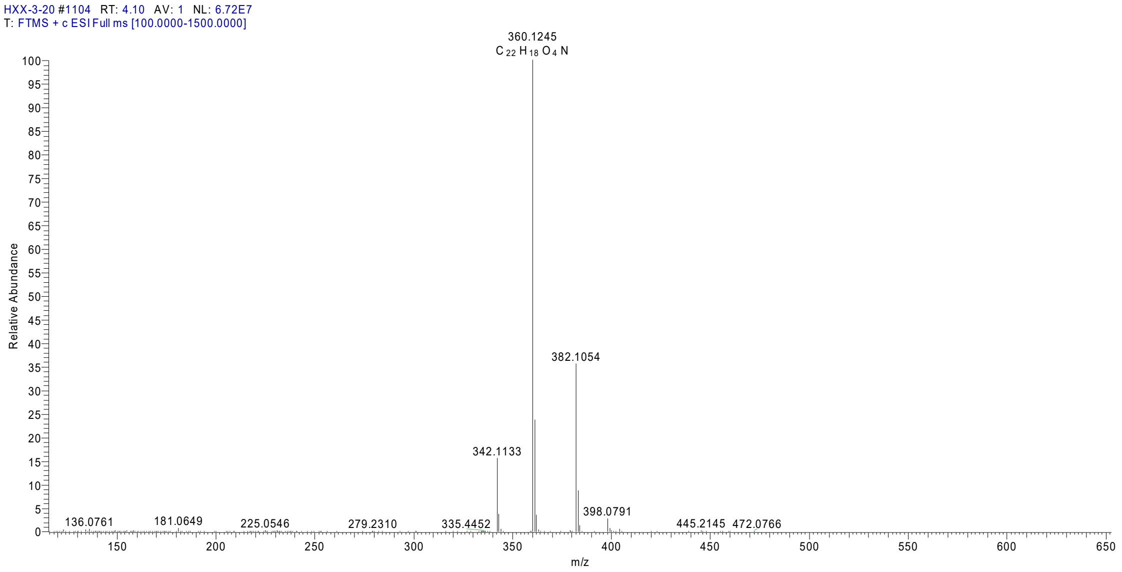

^{1}H-NMR:

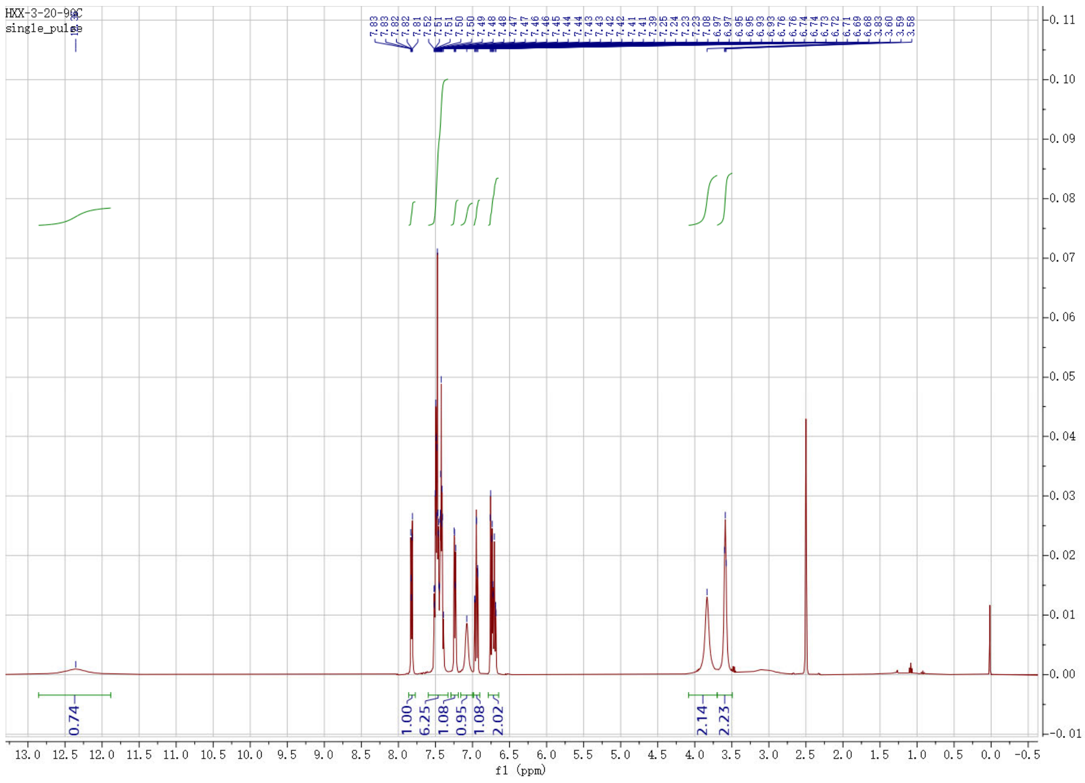

Compound **A3**:

HR-ESI-MS:

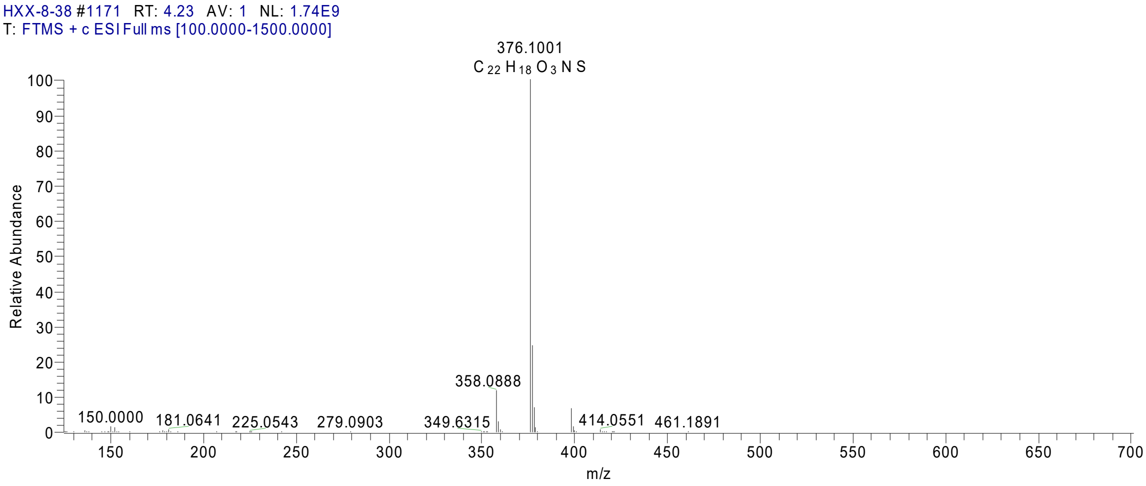

^{1}H-NMR:

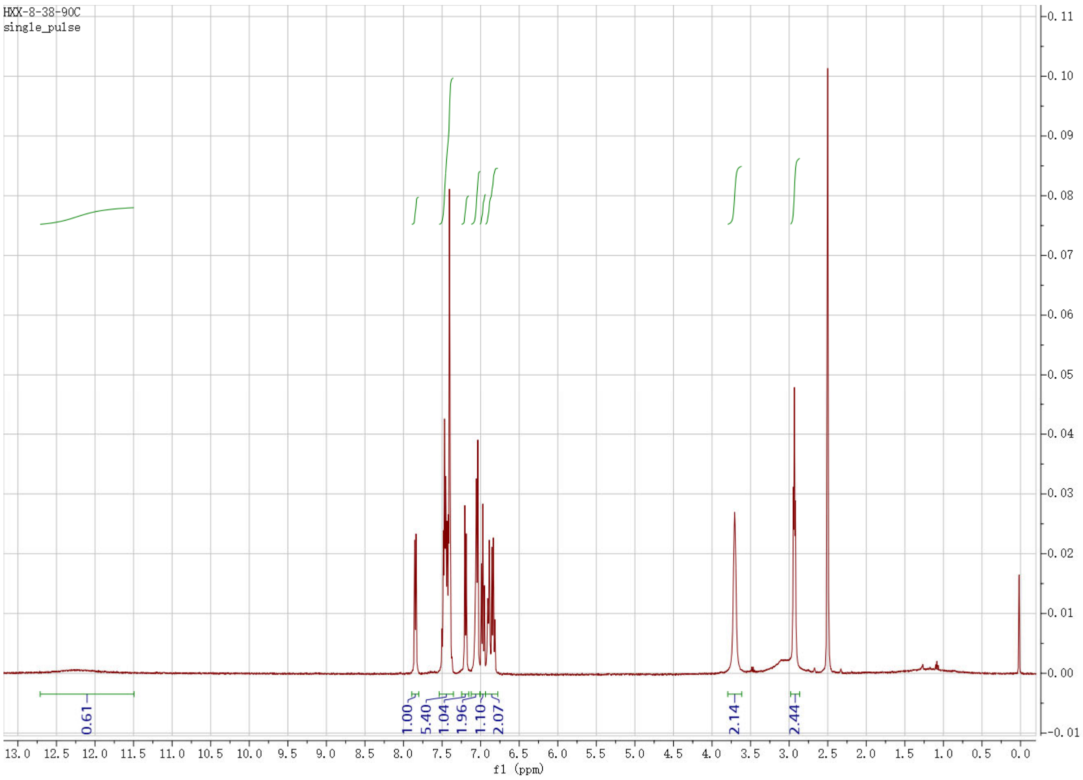

Compound **A4**:

HR-ESI-MS:

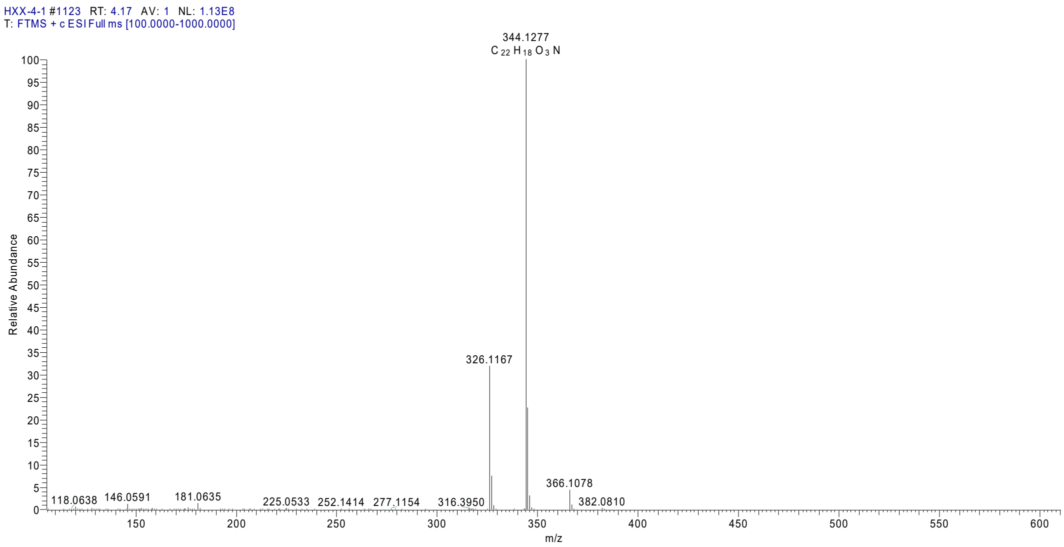

^{1}H-NMR:

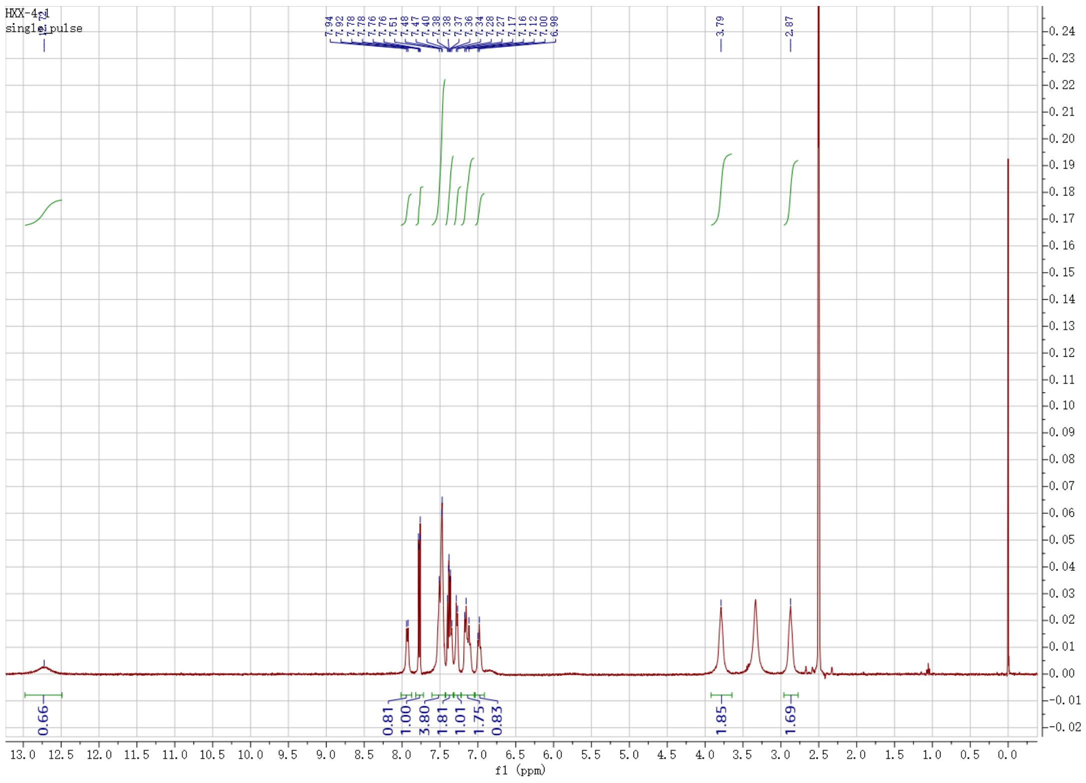

Compound **A5**:

HR-ESI-MS:

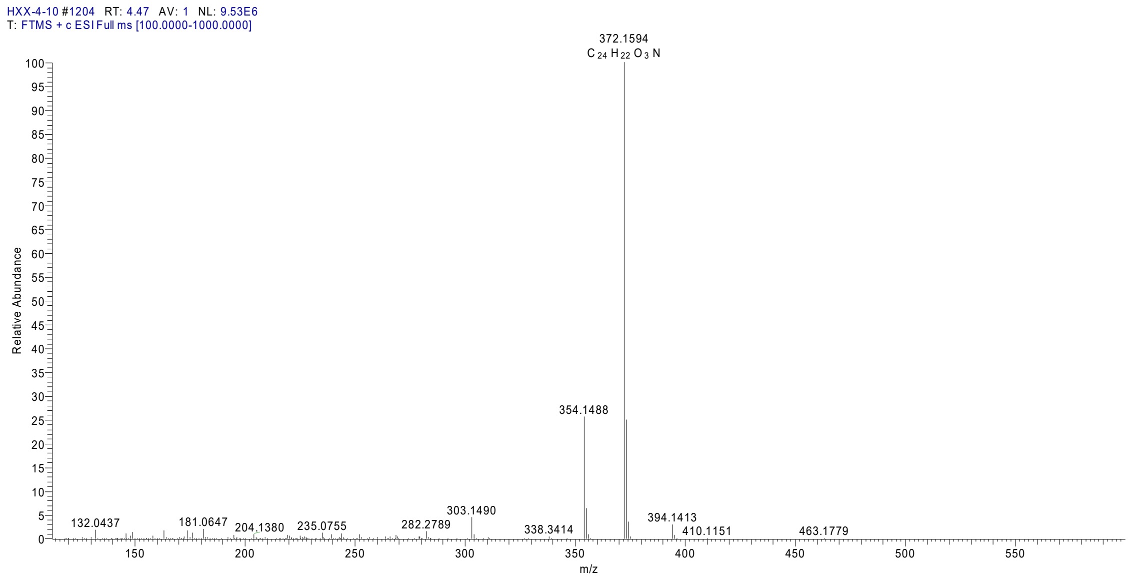

^{1}H-NMR:

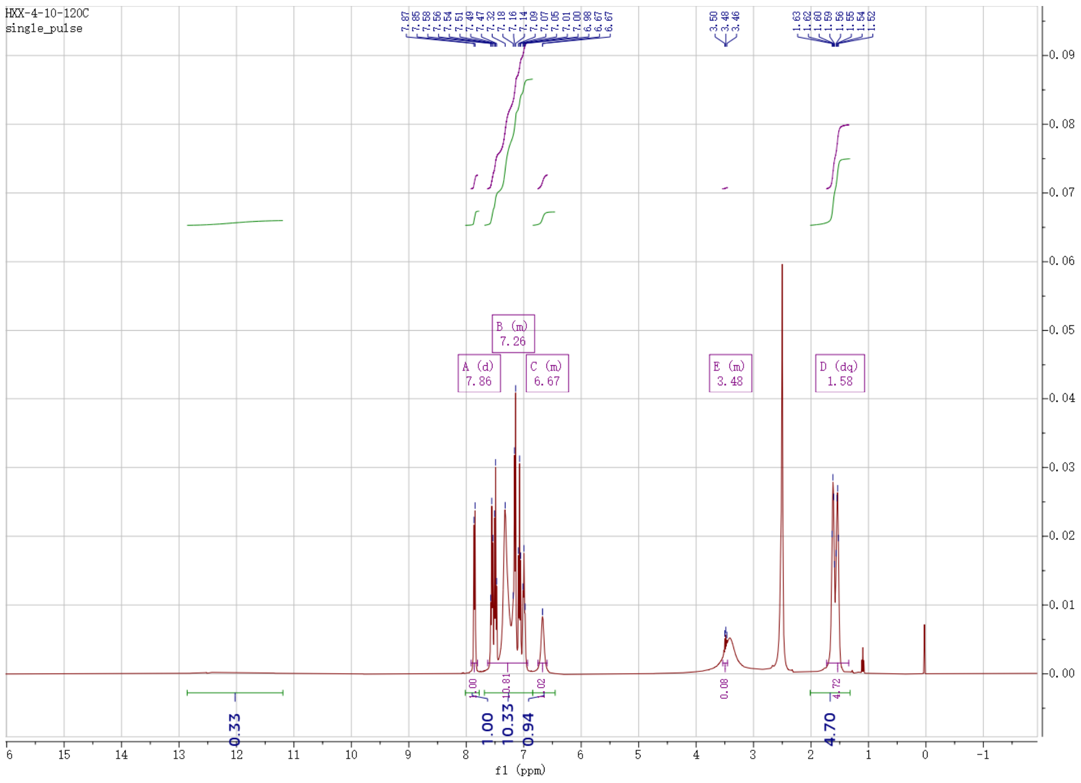

Compound **A6**:

HR-ESI-MS:

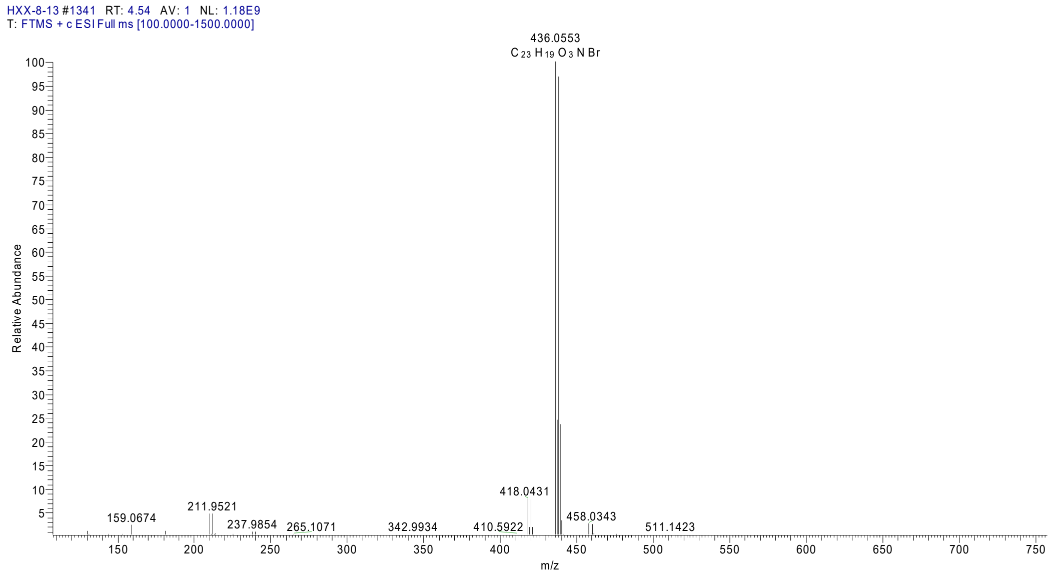

^{1}H-NMR:

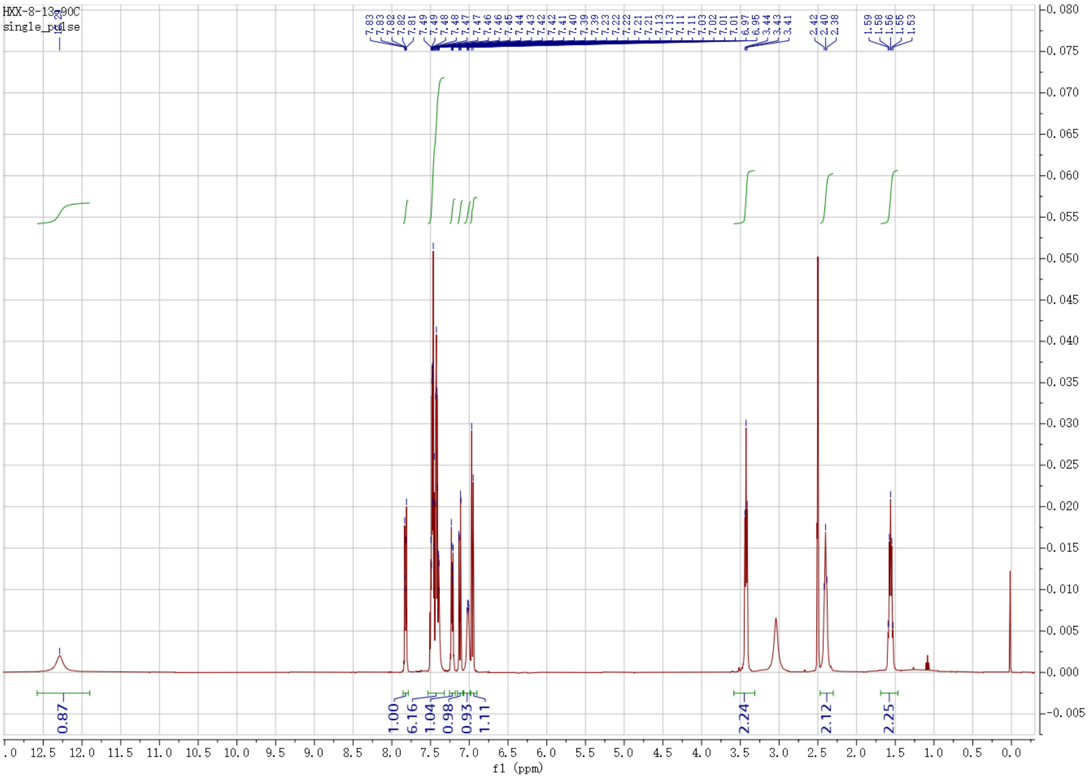

Compound **A7**:

HR-ESI-MS:

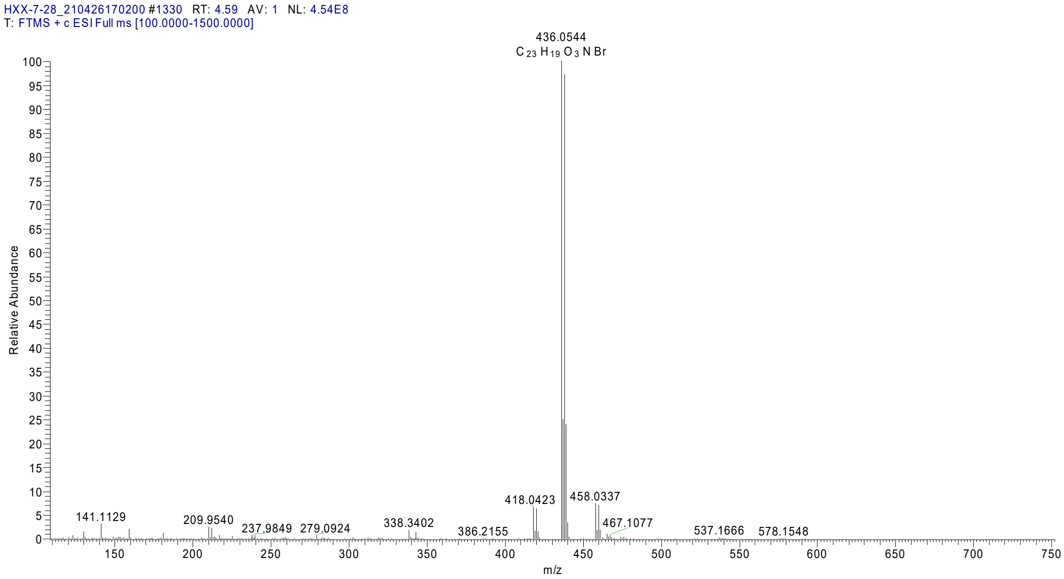

^{1}H-NMR:

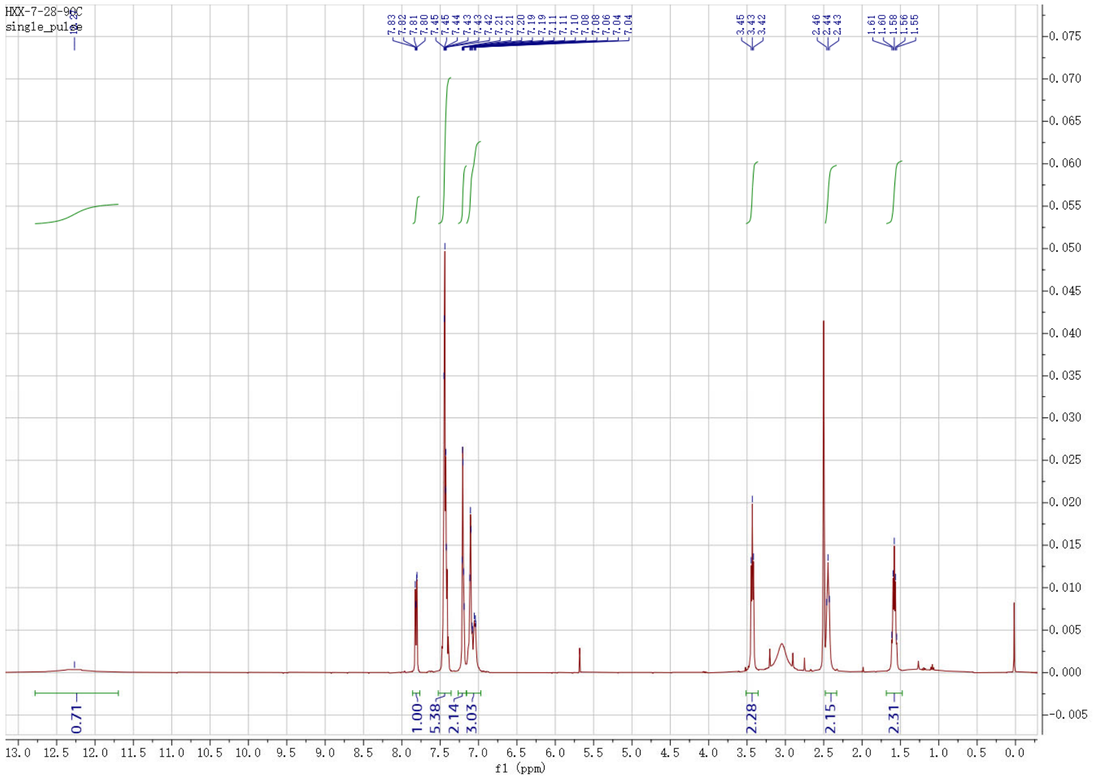

Compound **A8**:

HR-ESI-MS:

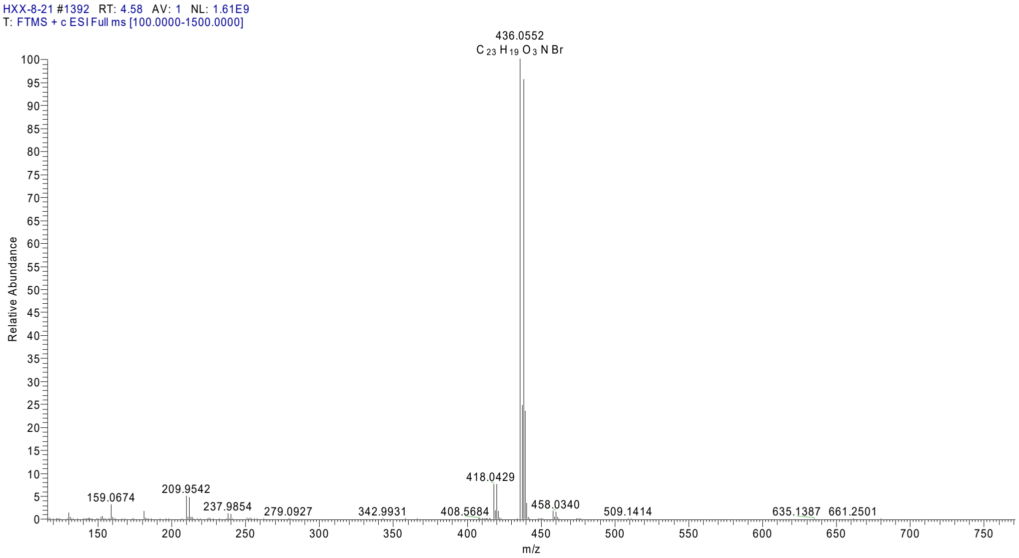

^{1}H-NMR:

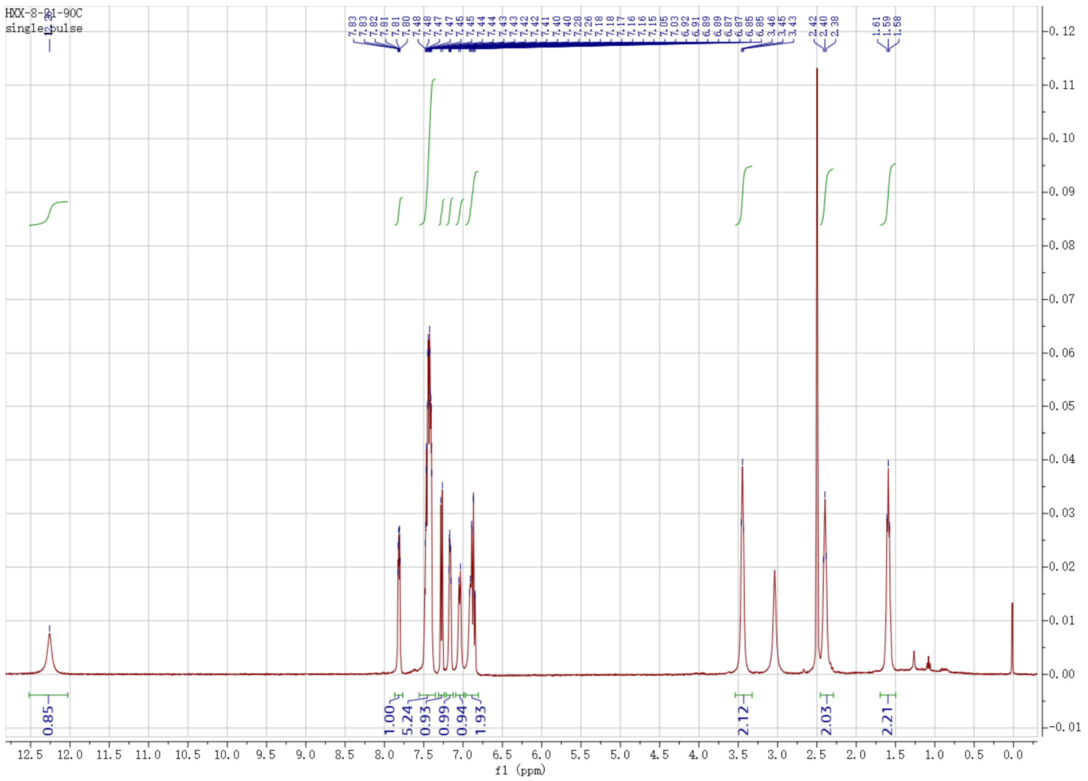

Compound **A9**:

HR-ESI-MS:

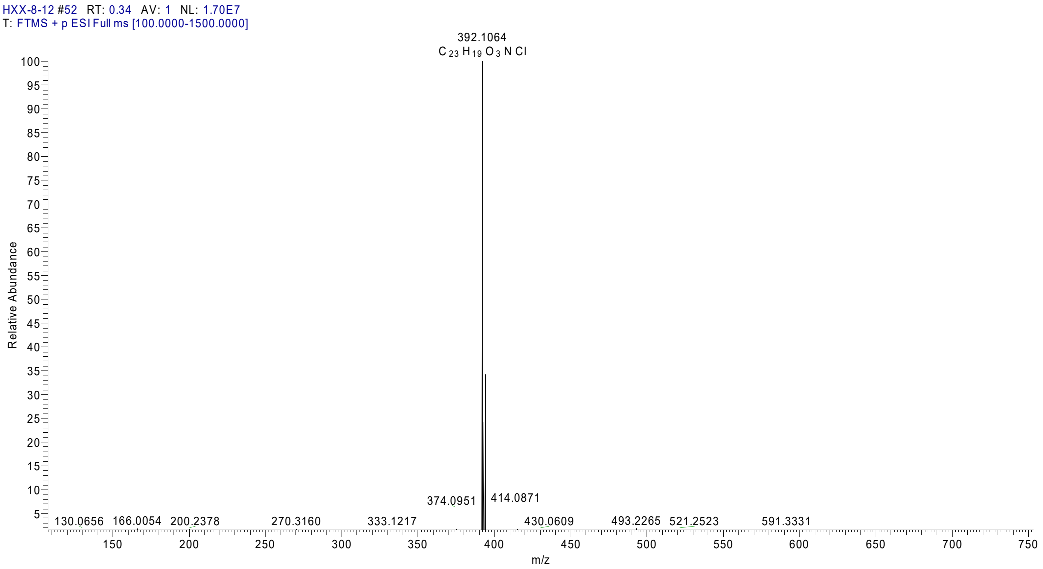

^{1}H-NMR:

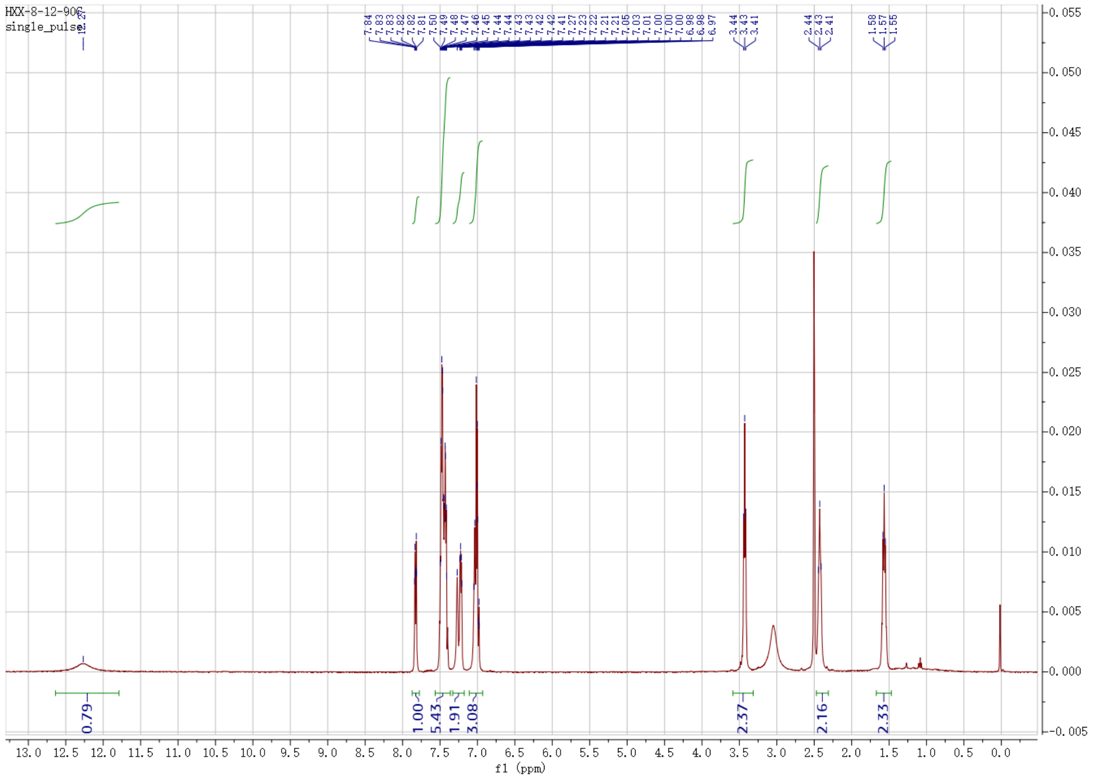

Compound **A10**:

HR-ESI-MS:

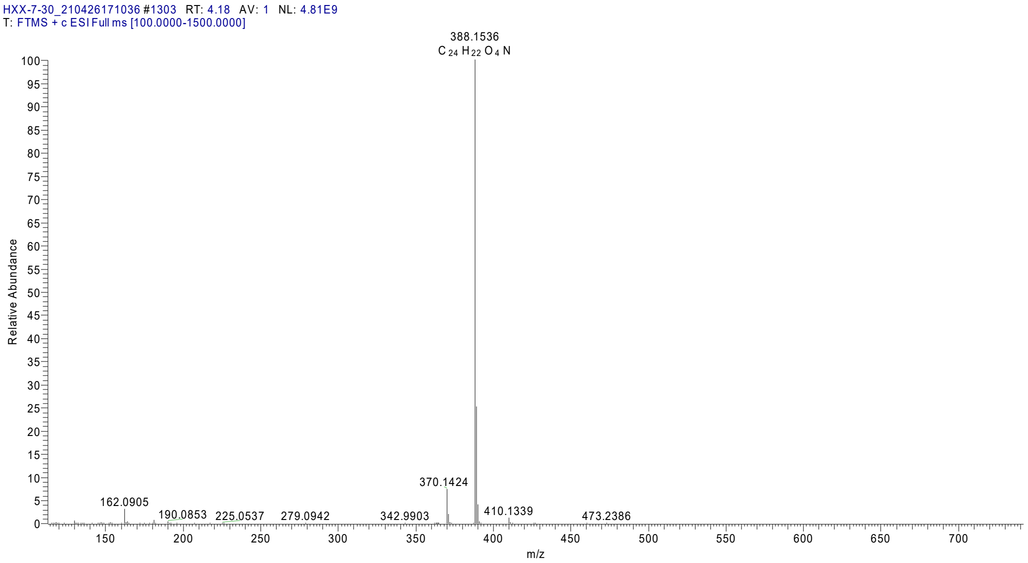

^{1}H-NMR:

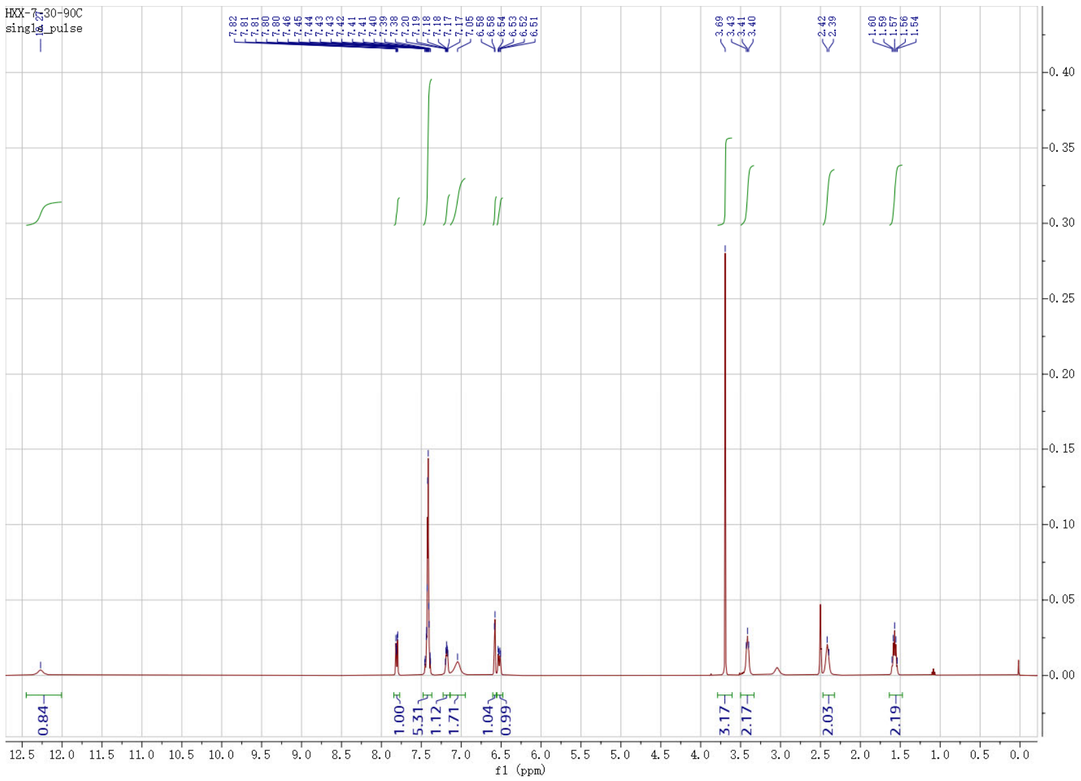

Compound **A11**:

HR-ESI-MS:

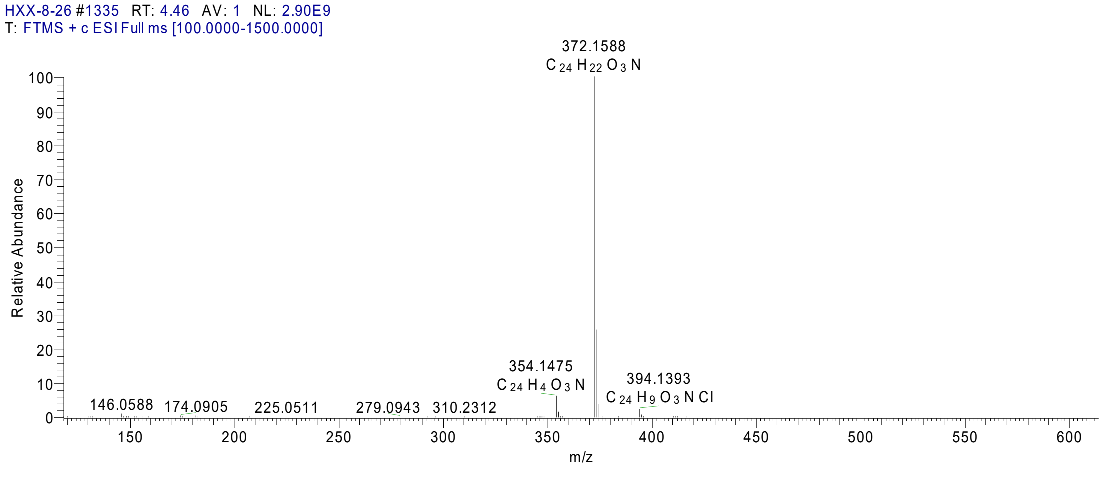

^{1}H-NMR:

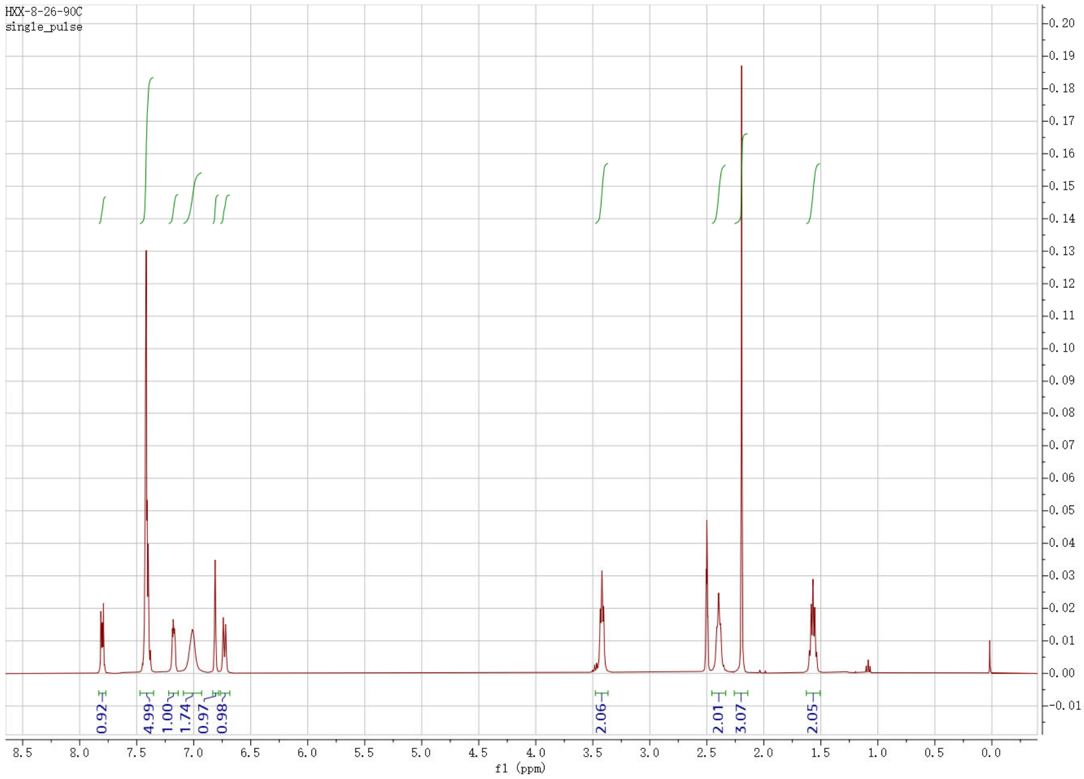

Compound **A12**:

HR-ESI-MS:

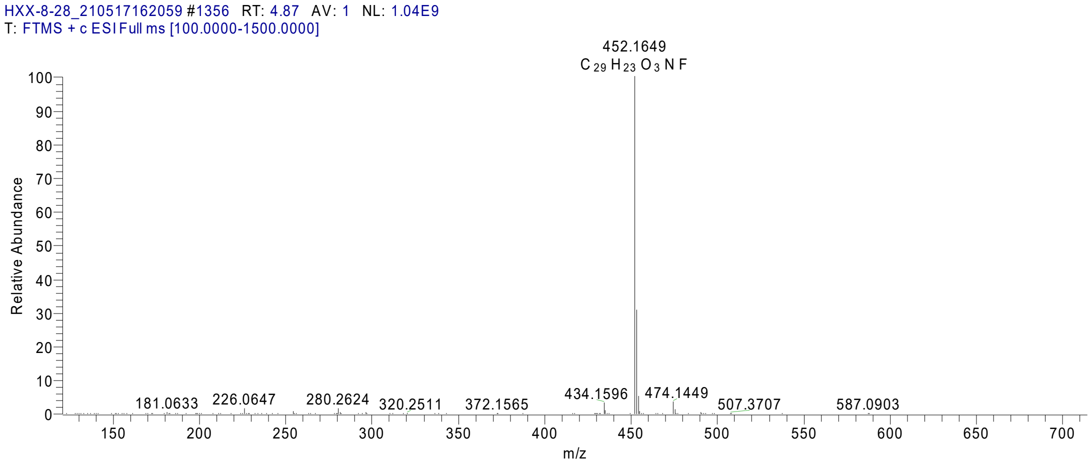

^{1}H-NMR:

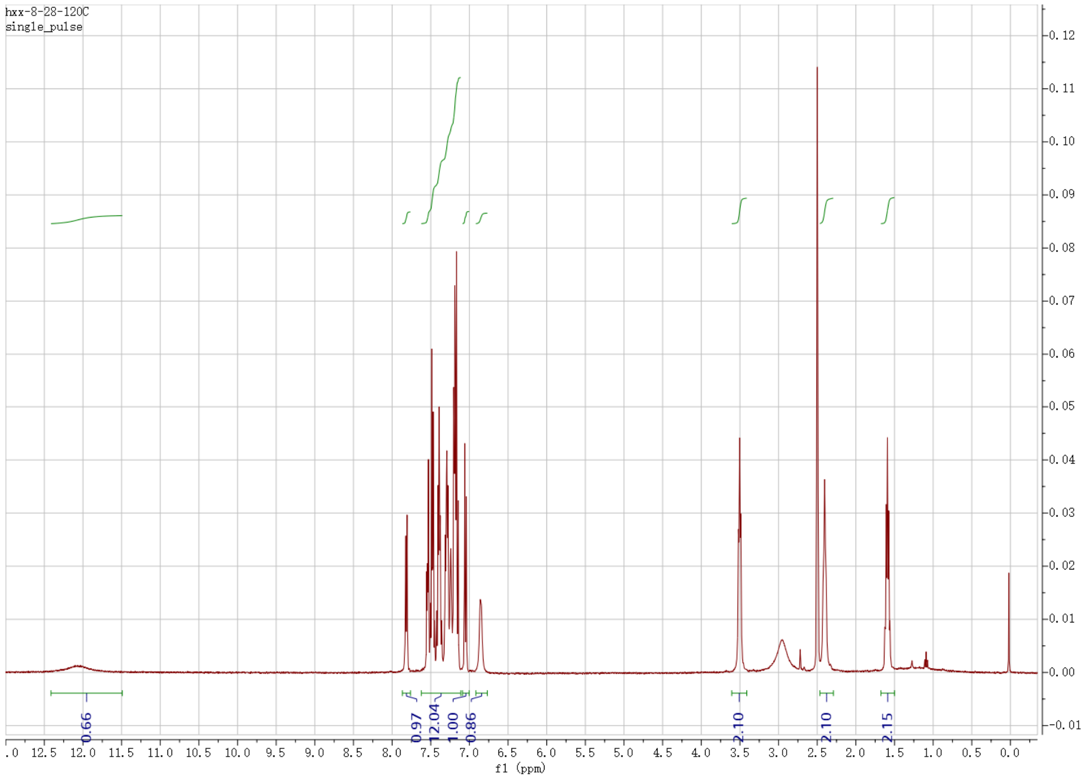

Compound **A13**:

HR-ESI-MS:

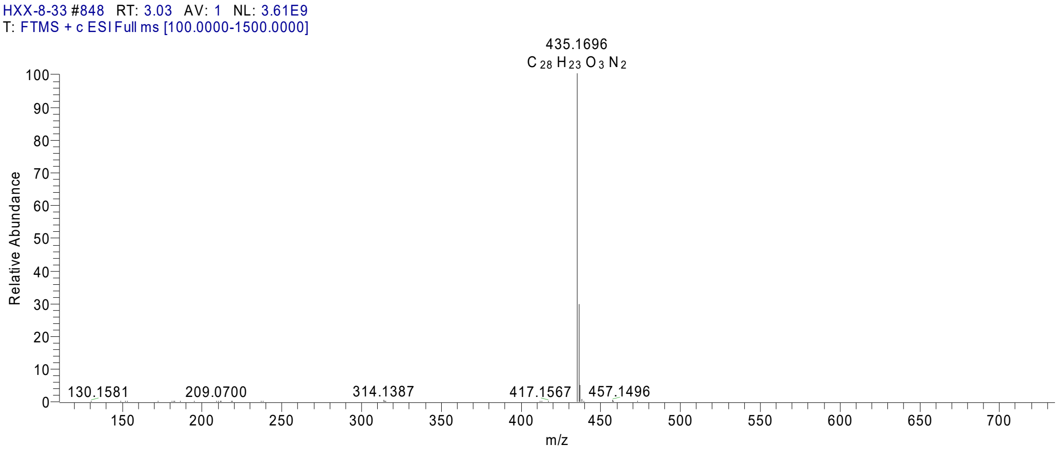

^{1}H-NMR:

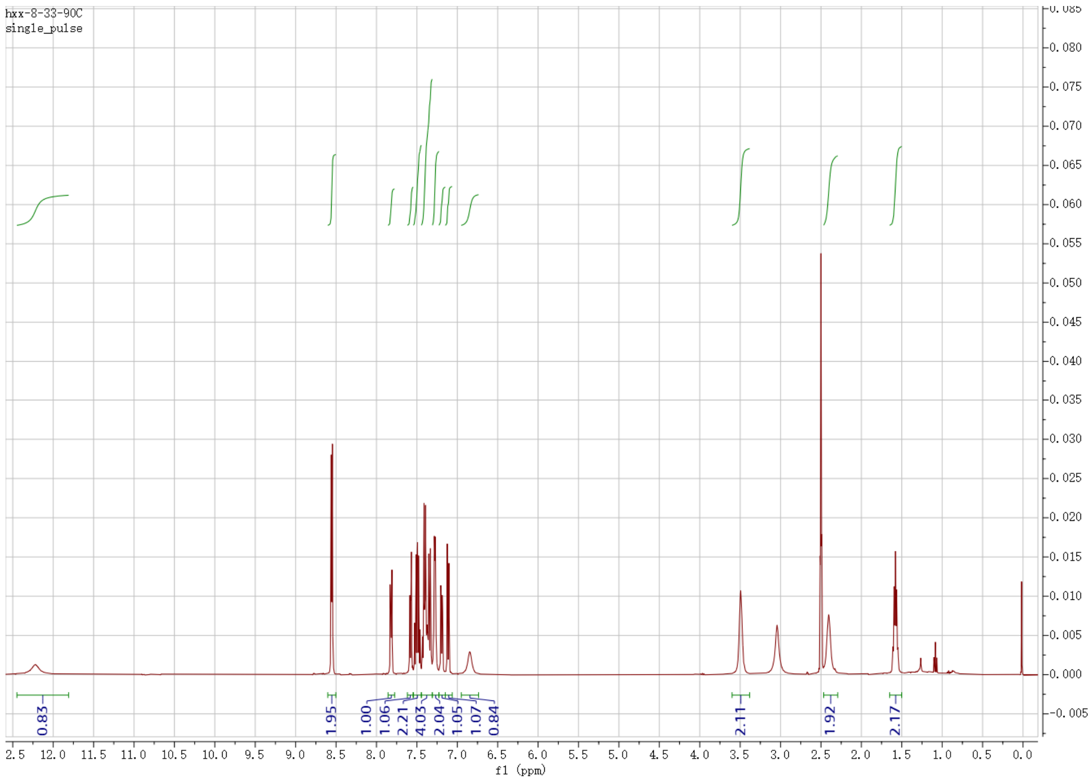

Compound **A14**:

HR-ESI-MS:

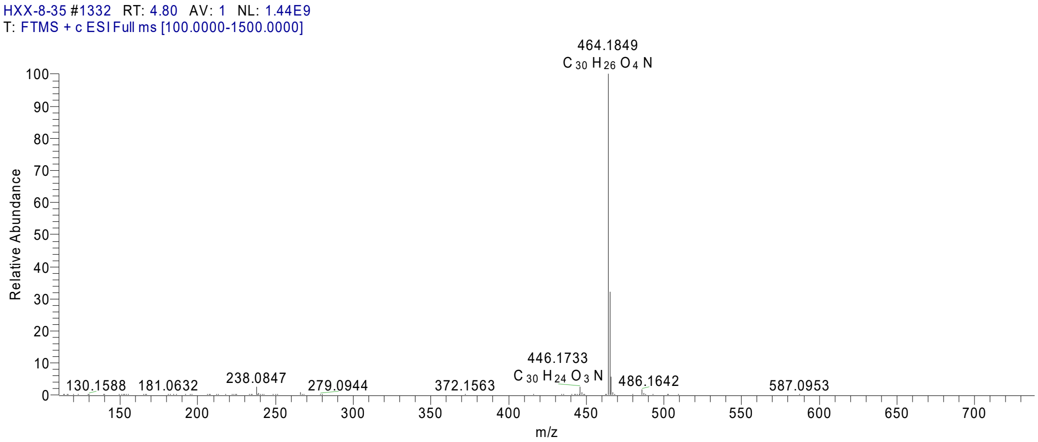

^{1}H-NMR:

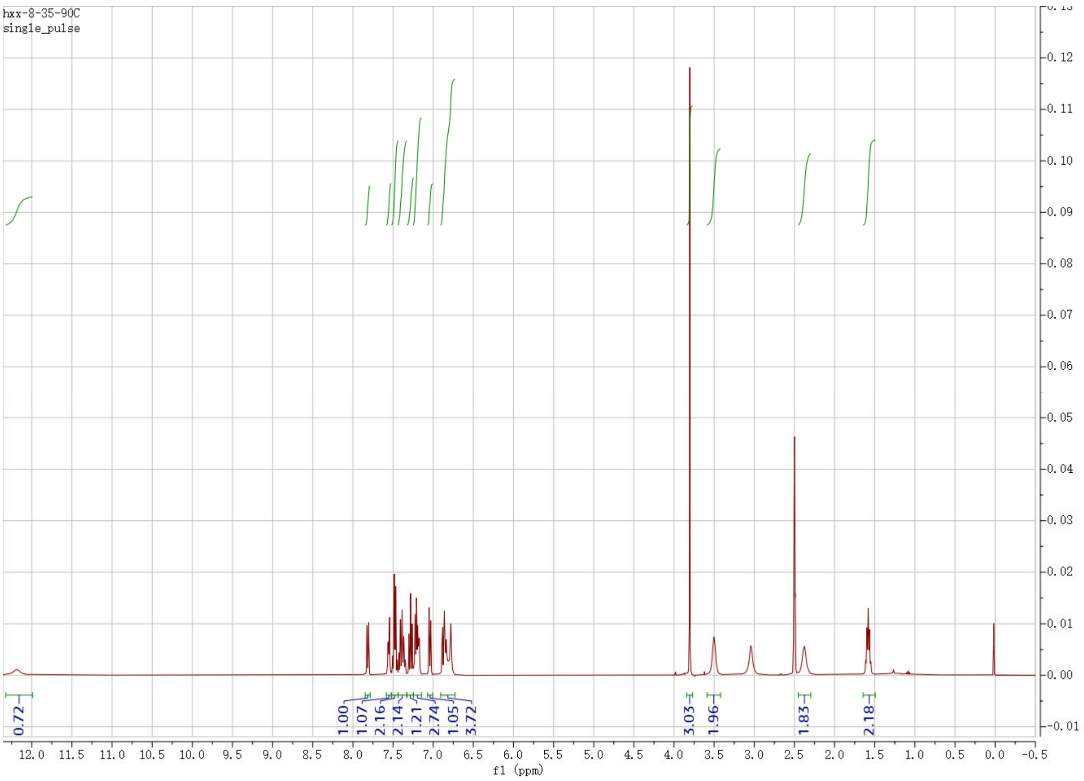

Compound **A15**:

HR-ESI-MS:

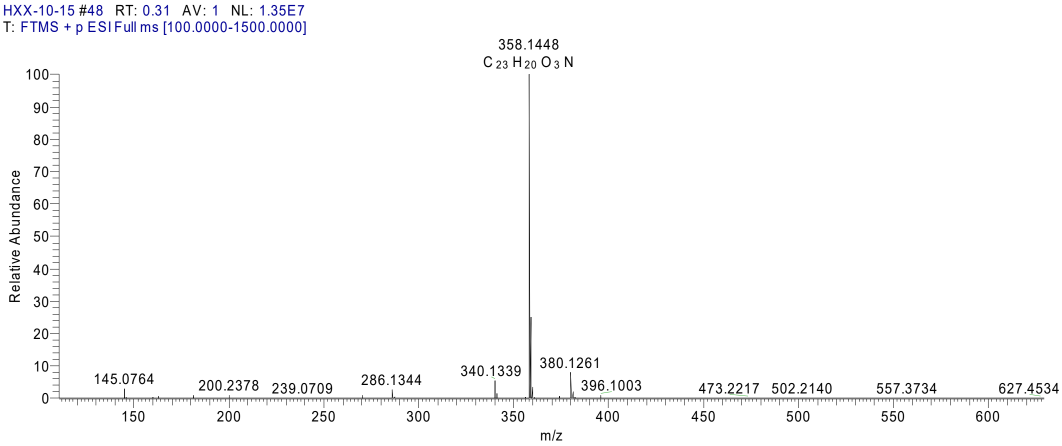

^{1}H-NMR:

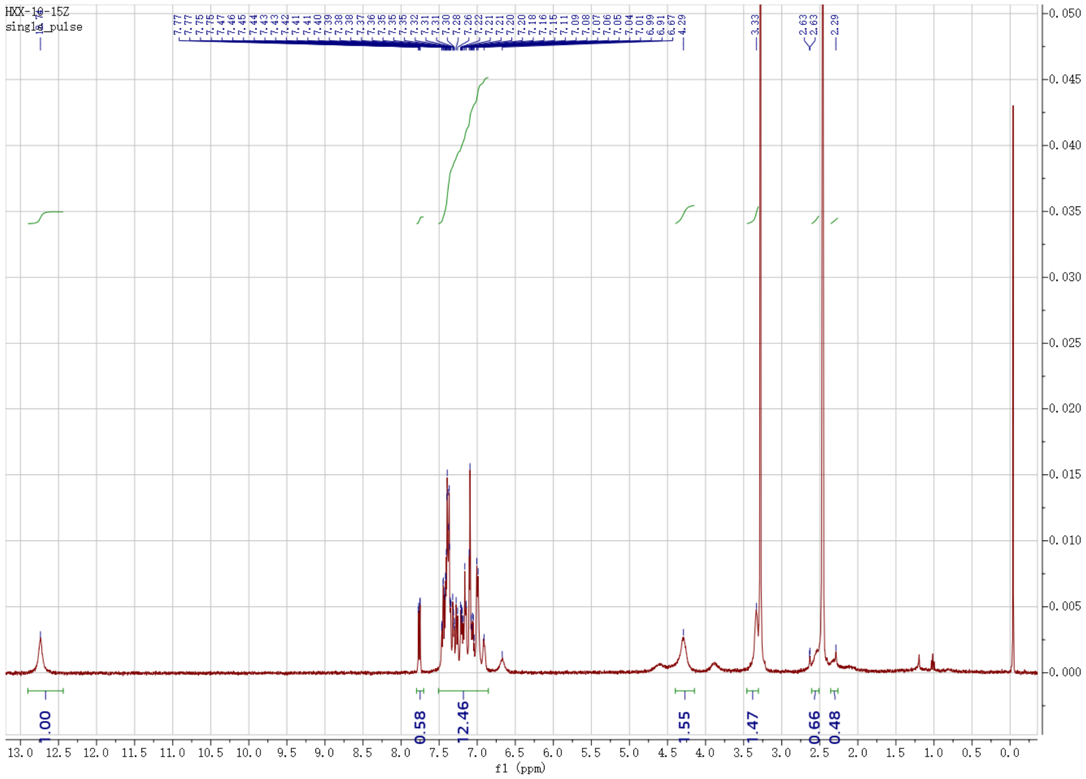

Compound **A16**:

HR-ESI-MS:

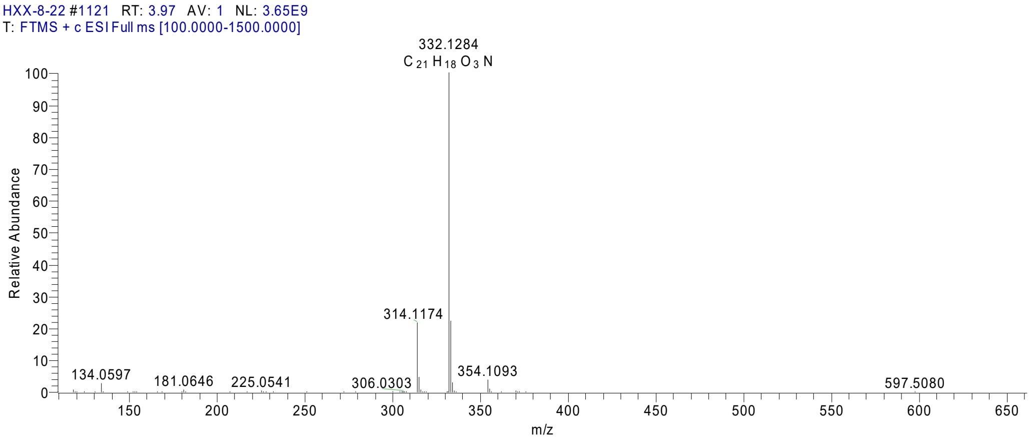

^{1}H-NMR:

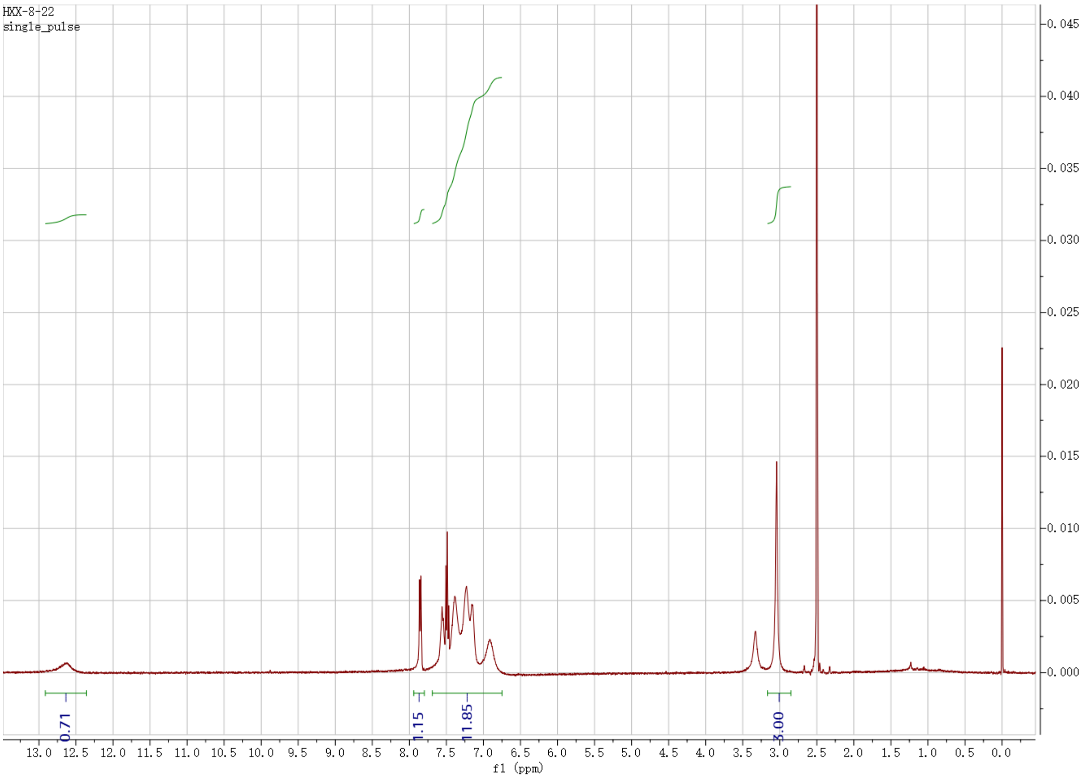

Compound **A17**:

HR-ESI-MS:

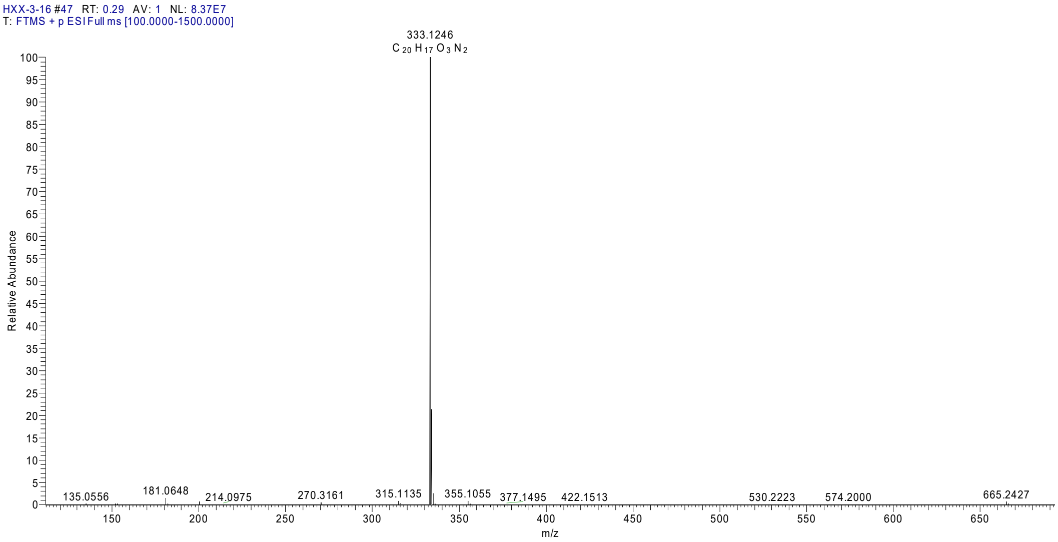

^{1}H-NMR:

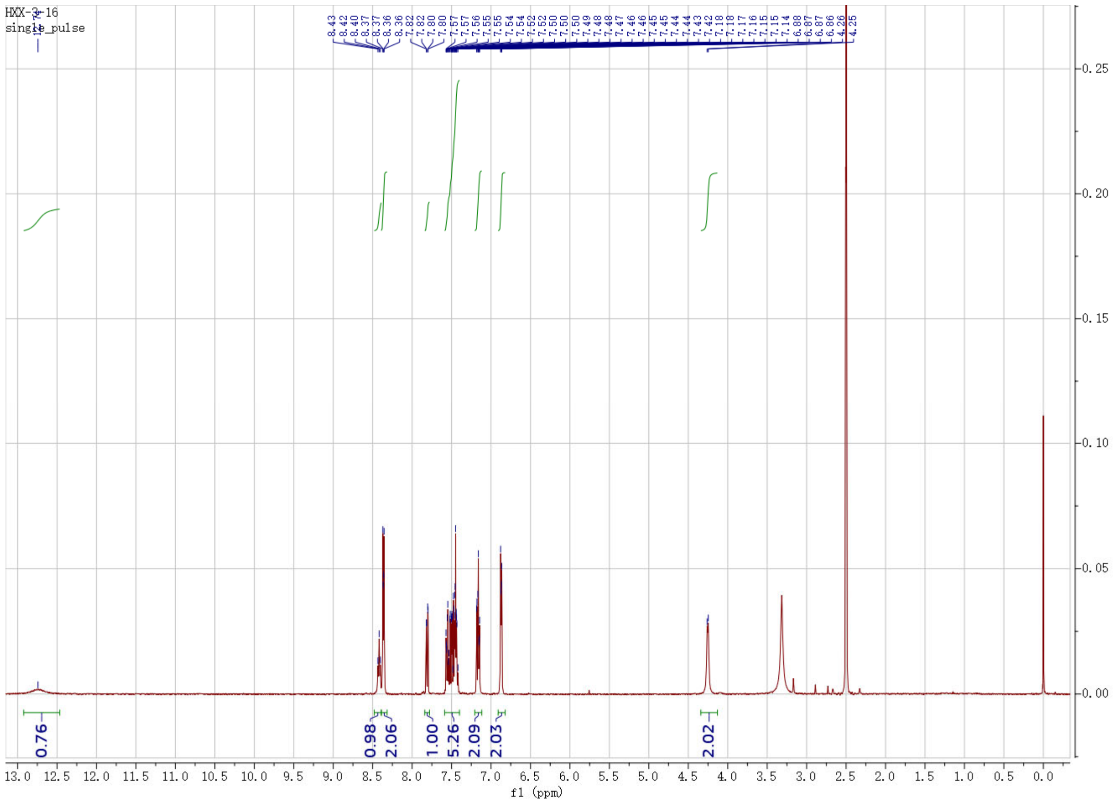

Compound **A18**:

HR-ESI-MS:

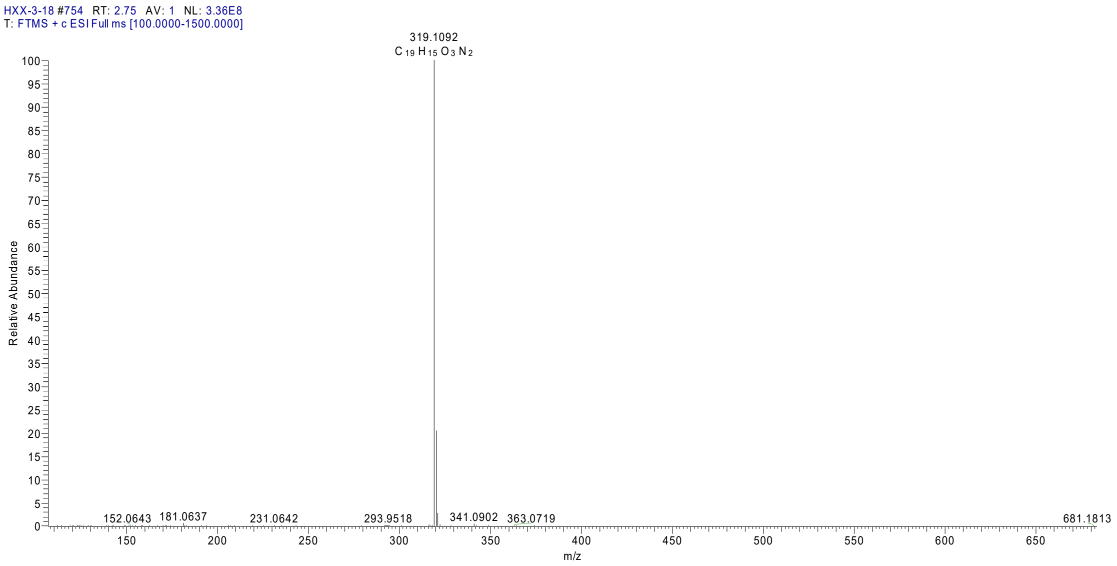

^{1}H-NMR:

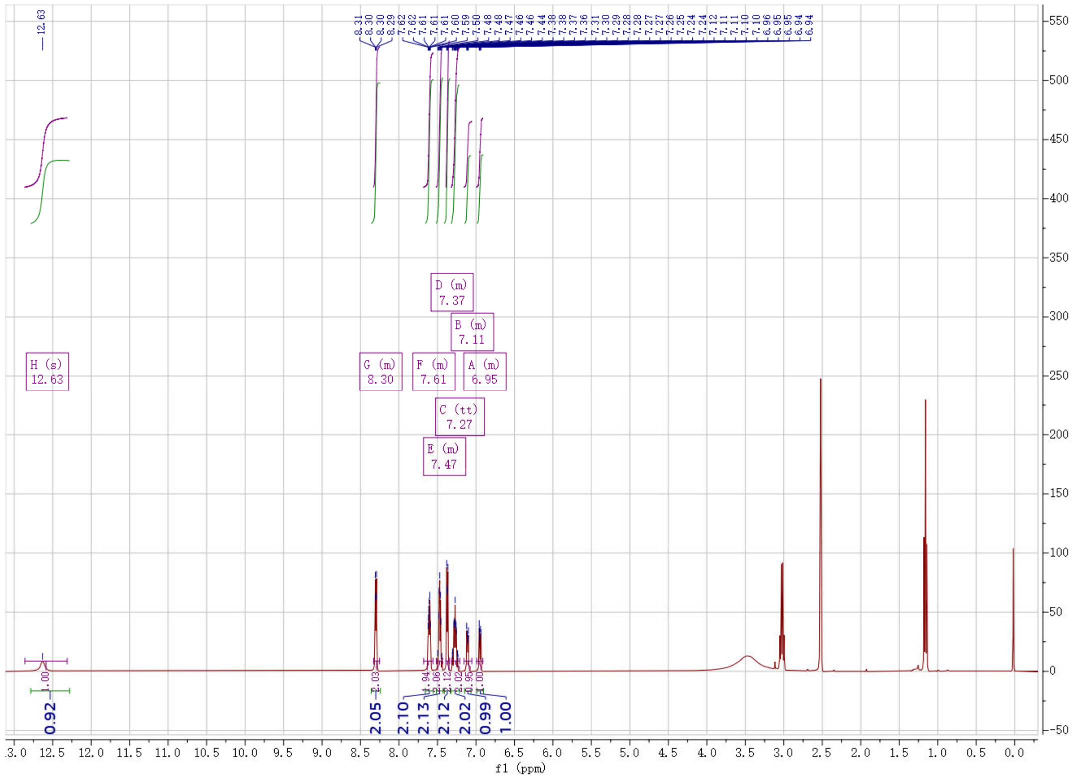

Compound **A19**:

HR-ESI-MS:

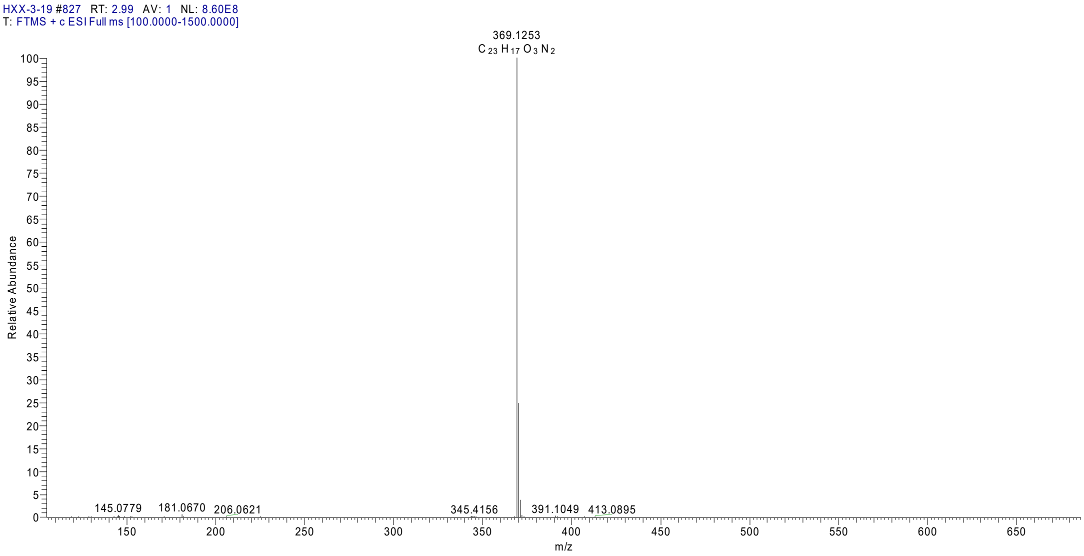

^{1}H-NMR:

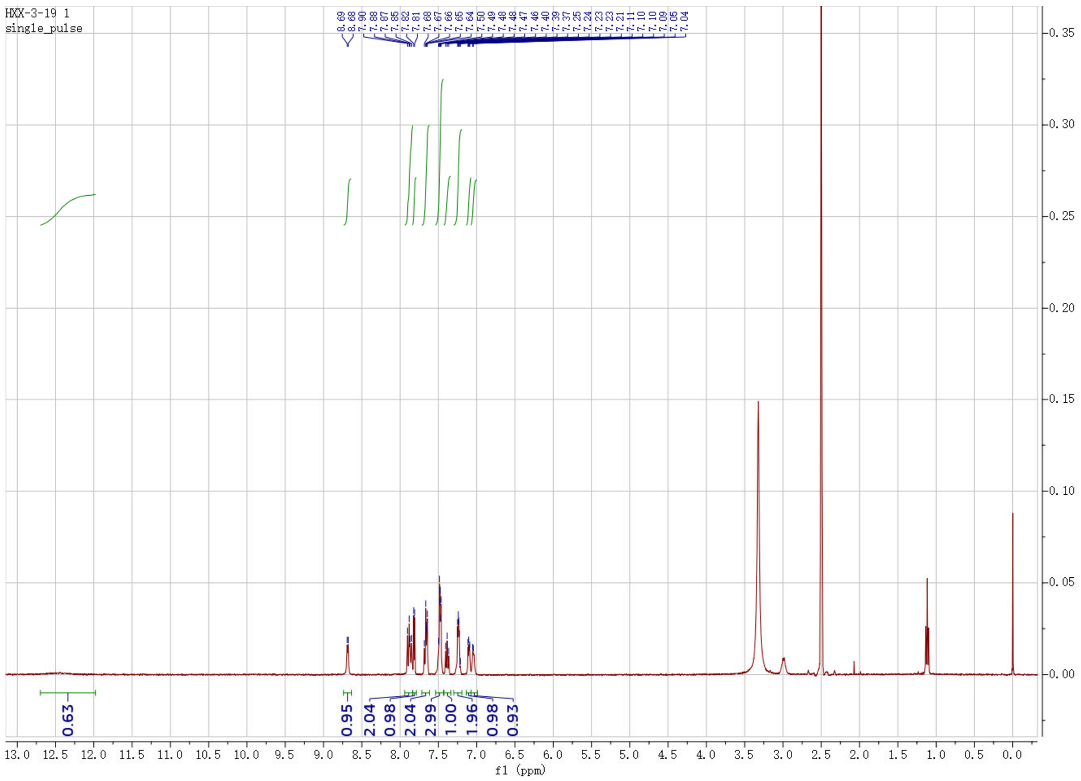

Compound **A20**:

HR-ESI-MS:

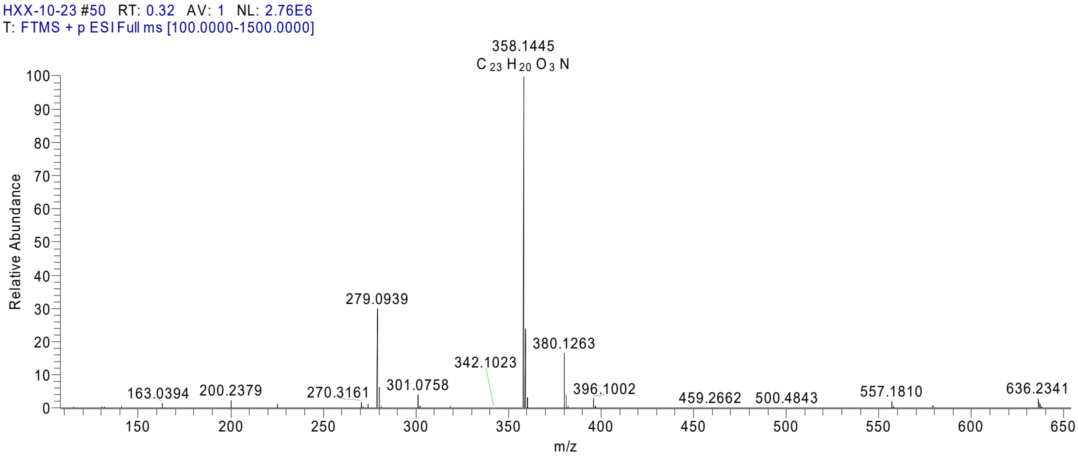

^{1}H-NMR:

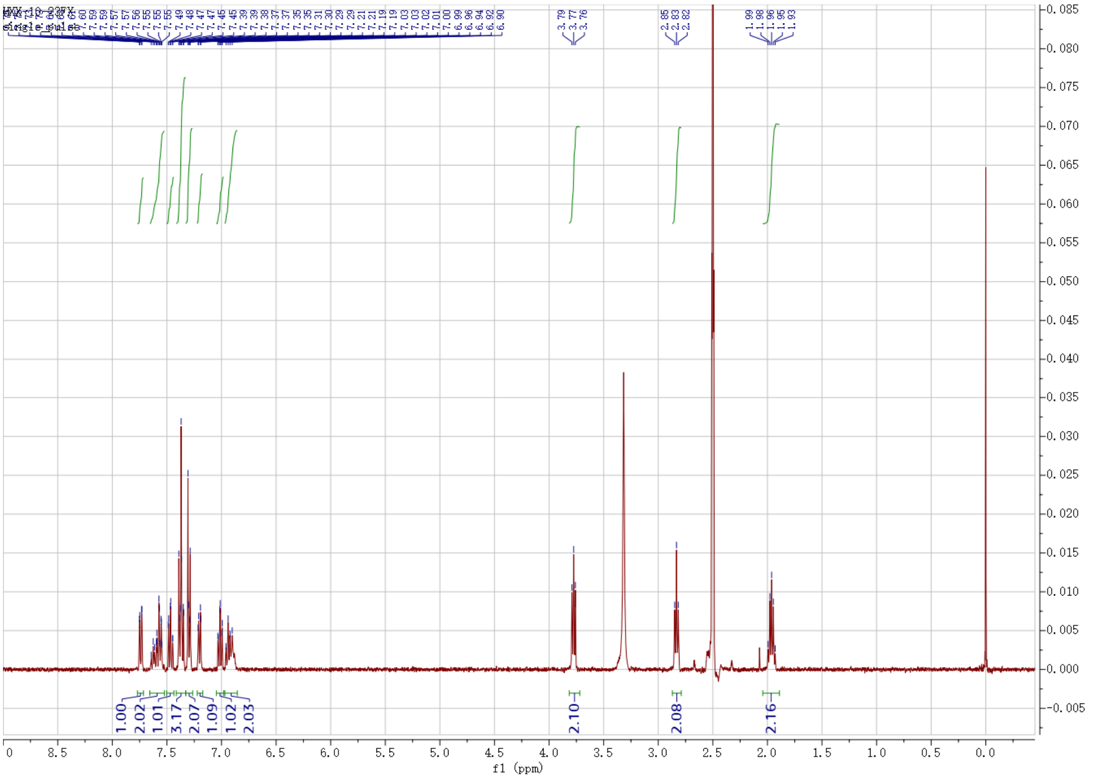

^{13}C-NMR:

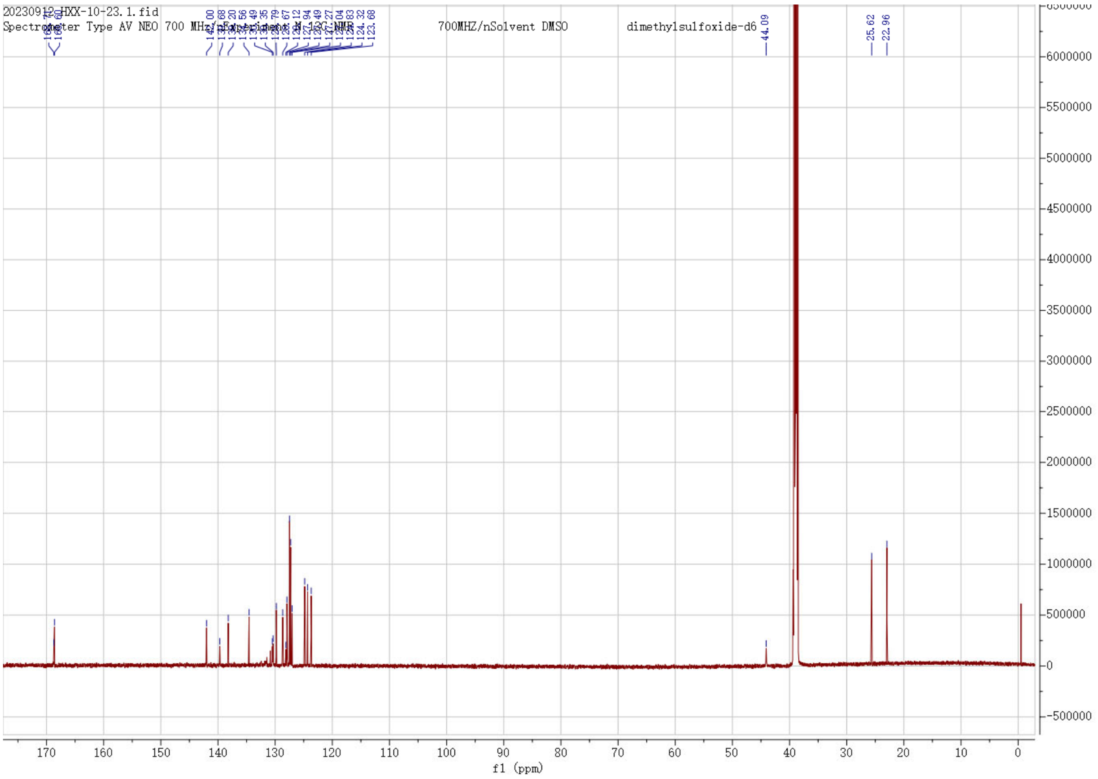

Compound **A21**:

HR-ESI-MS:

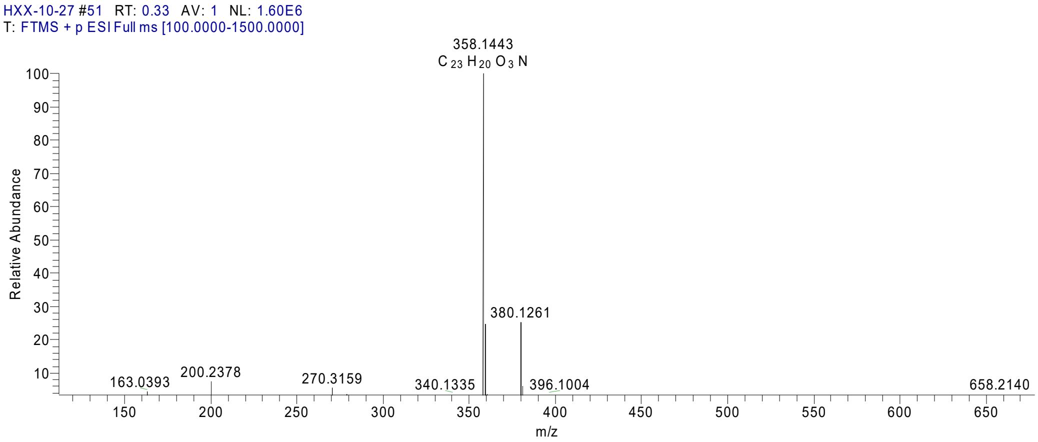

^{1}H-NMR:

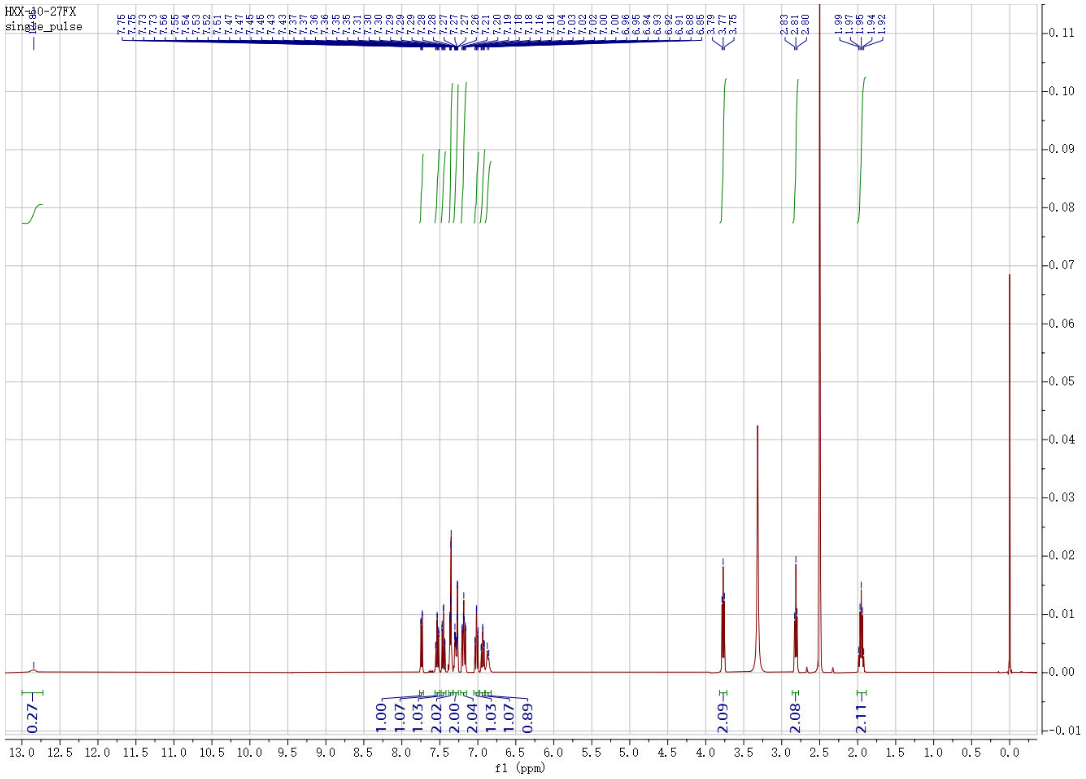

^{13}C-NMR:

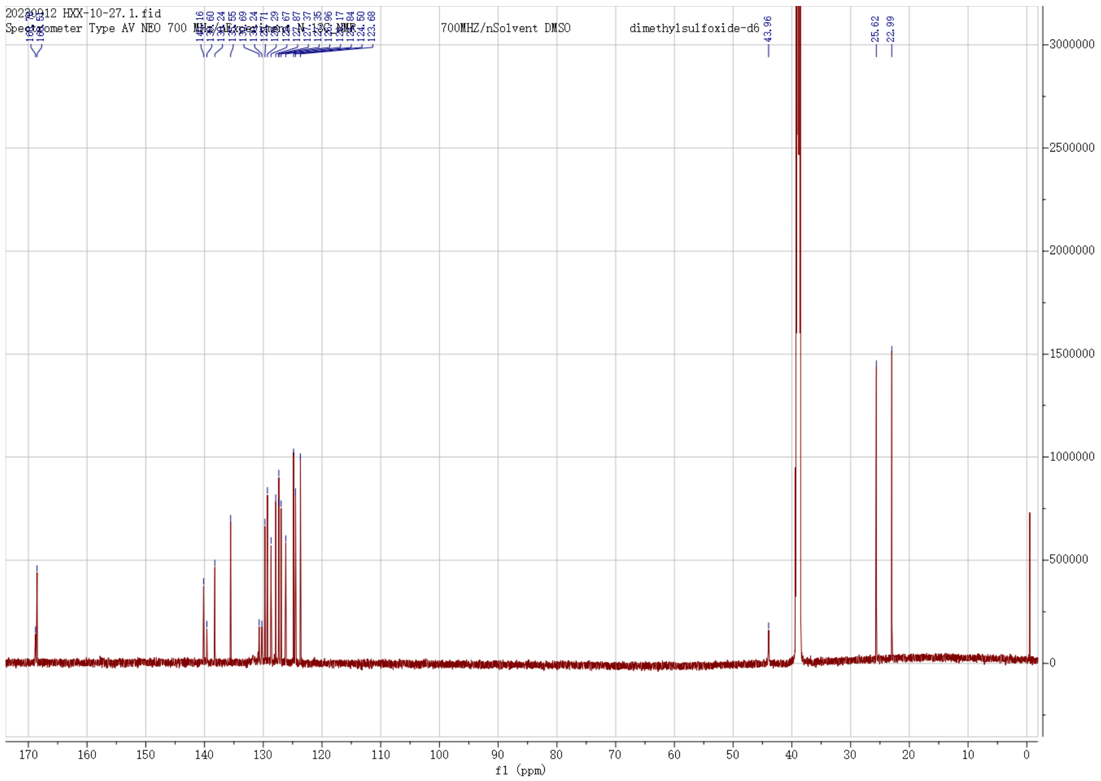

Compound **B1**:

HR-ESI-MS:

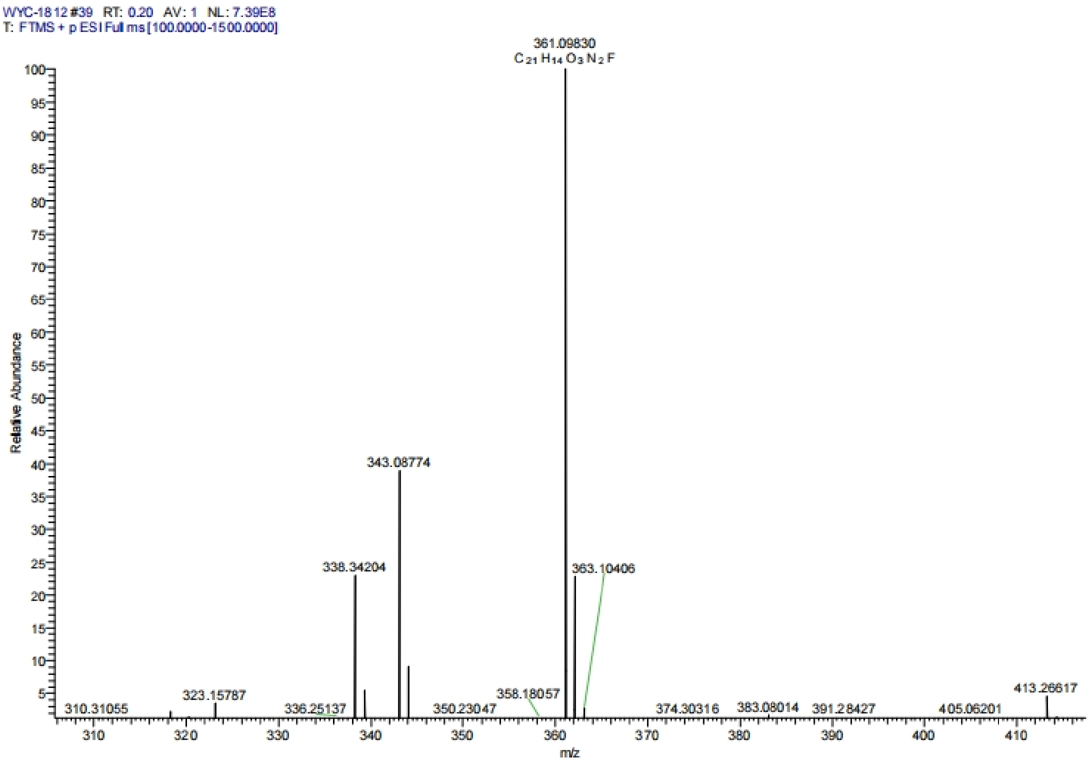

^{1}H-NMR:

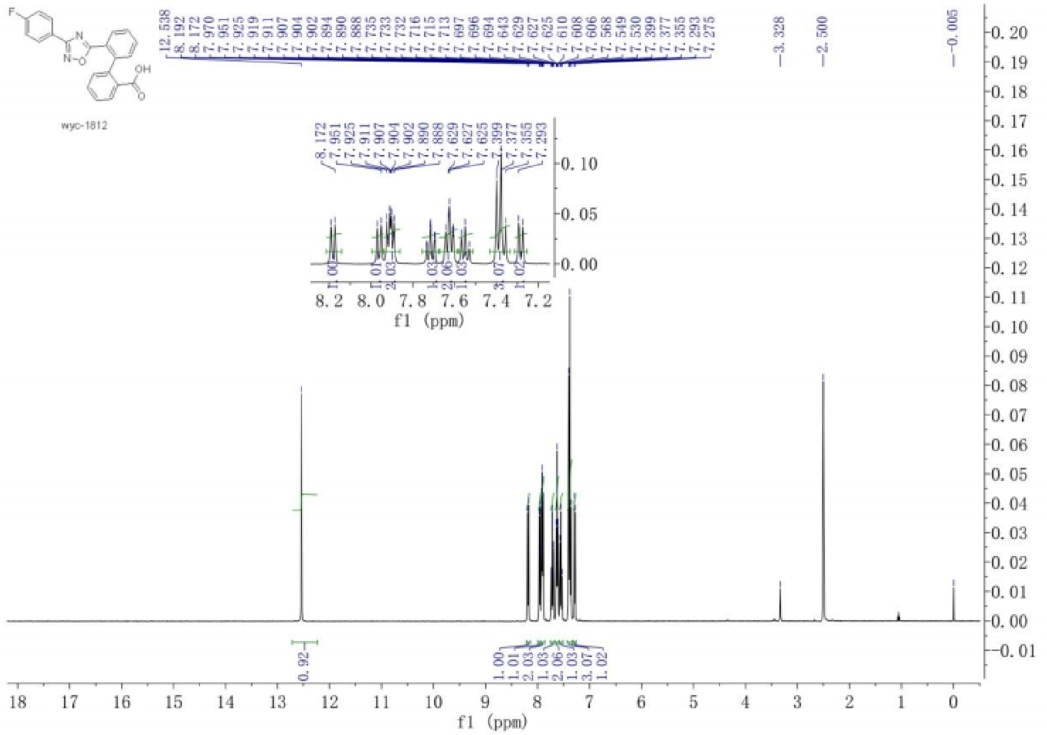

^{13}C-NMR:

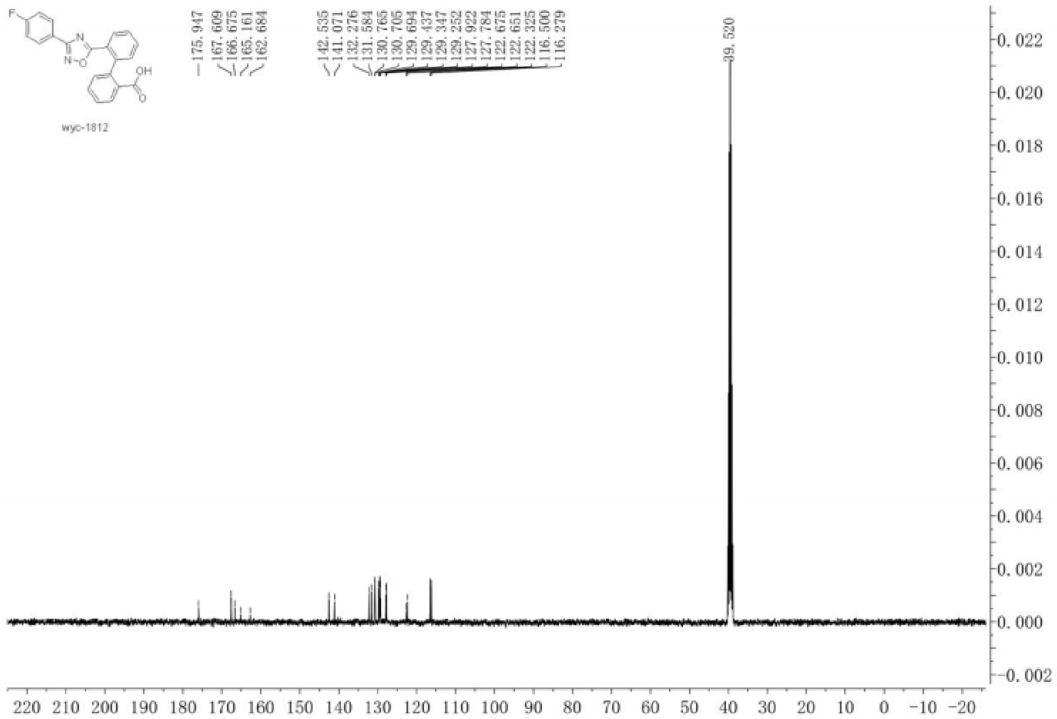

Compound **B2**:

HR-ESI-MS:

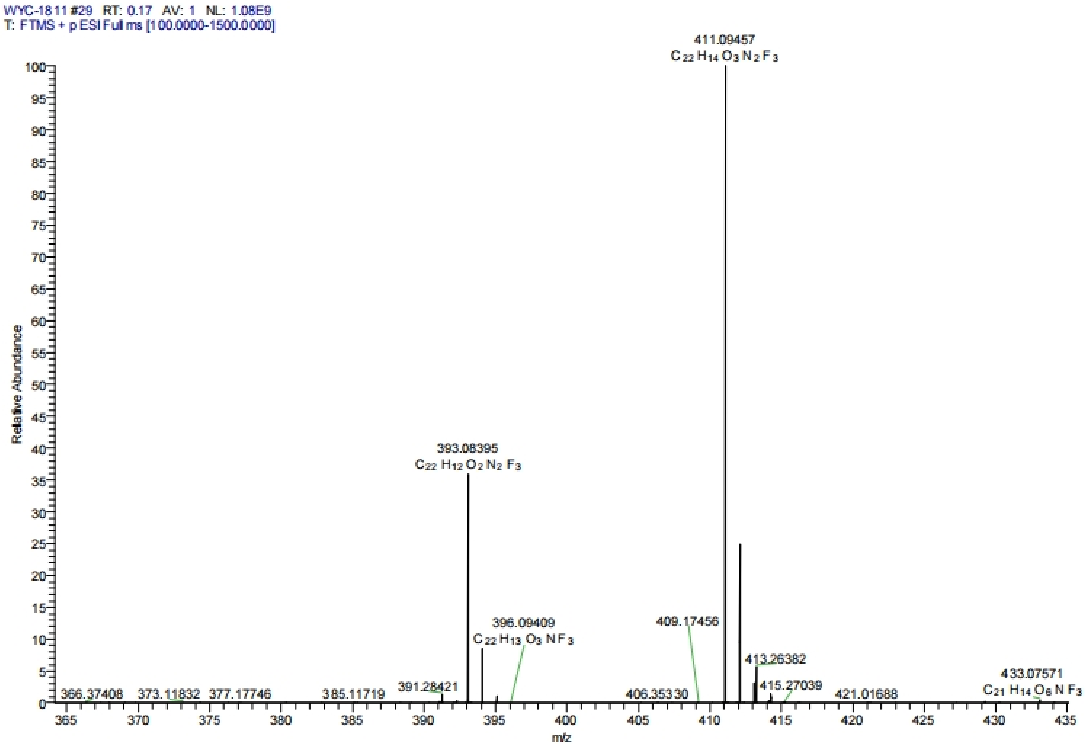

^{1}H-NMR:

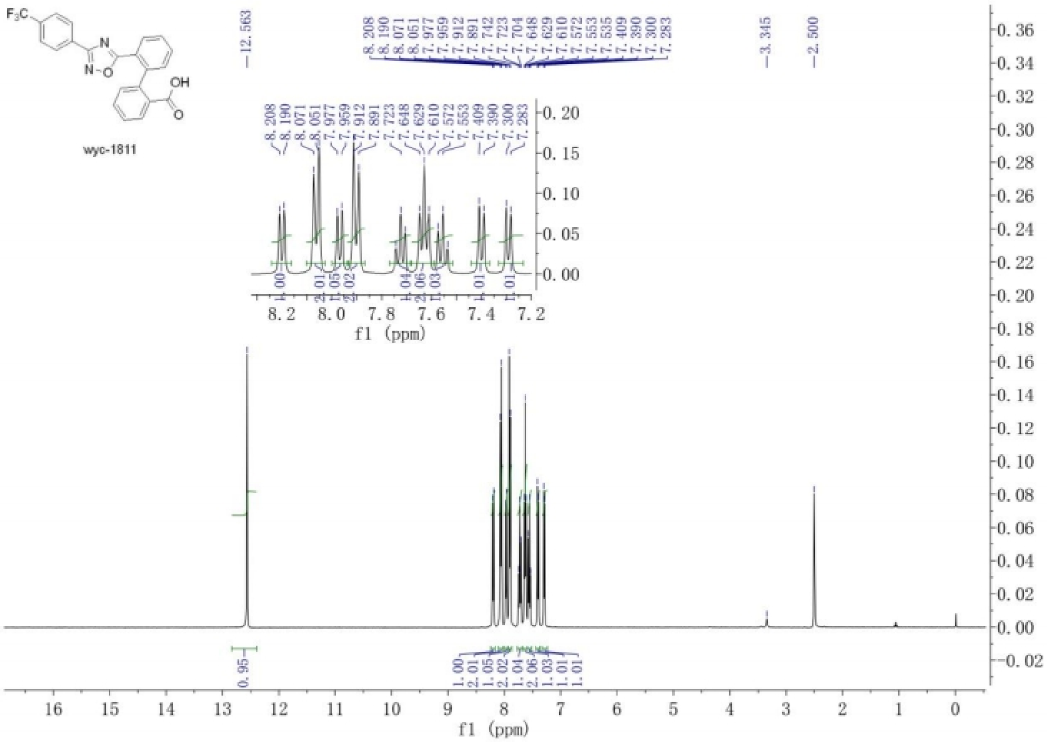

^{13}C-NMR:

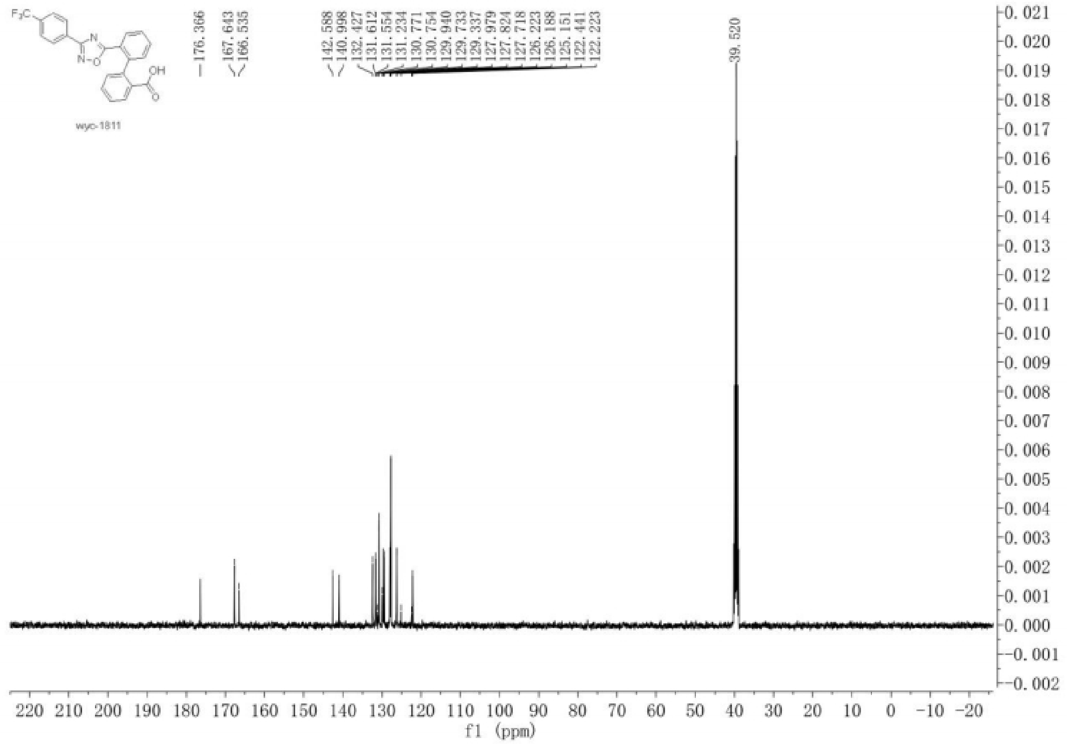

Compound **B3**:

HR-ESI-MS:

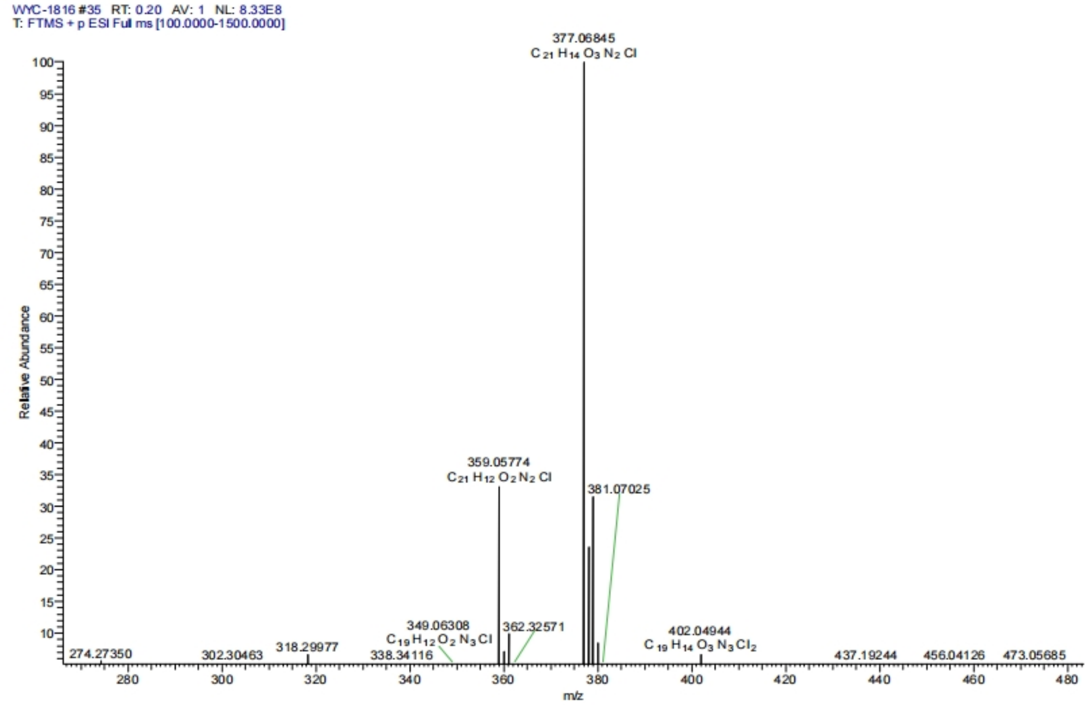

^{1}H-NMR:

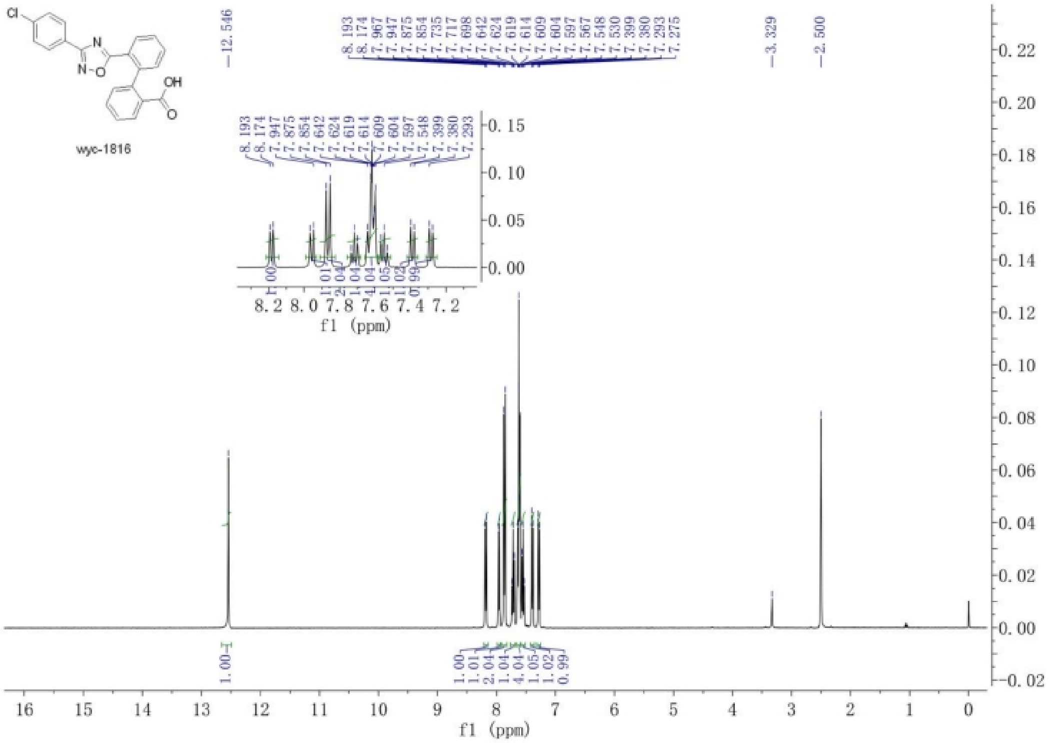

^{13}C-NMR:

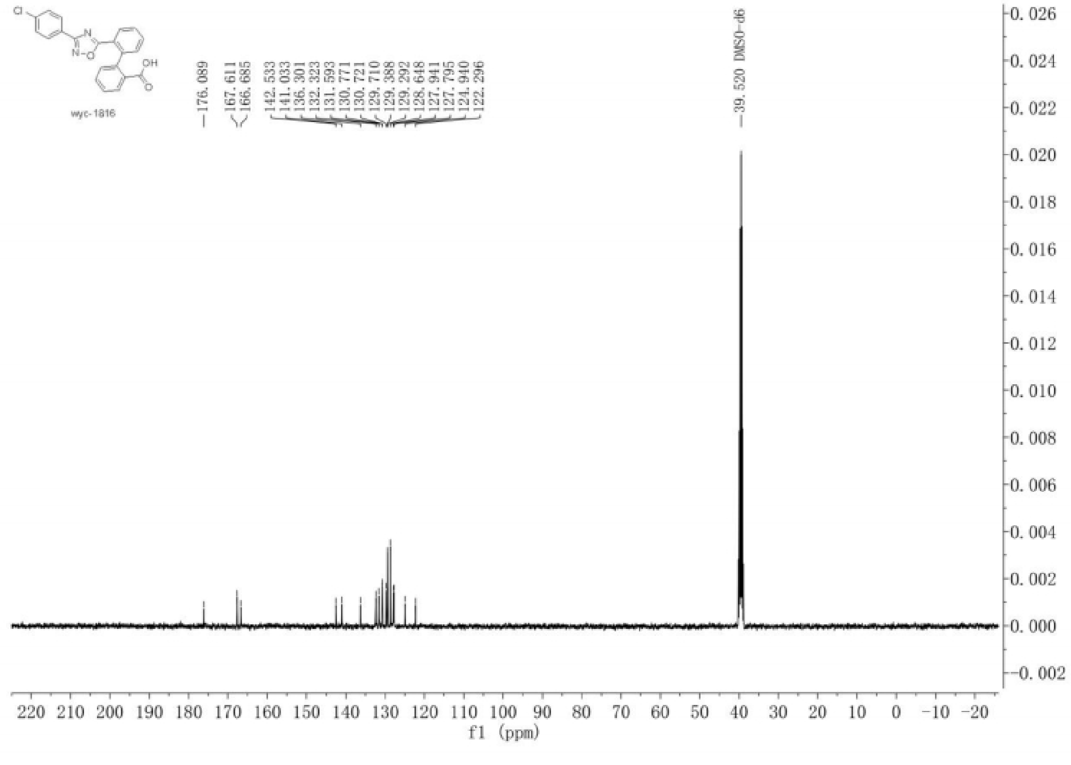

Compound **B4**:

HR-ESI-MS:

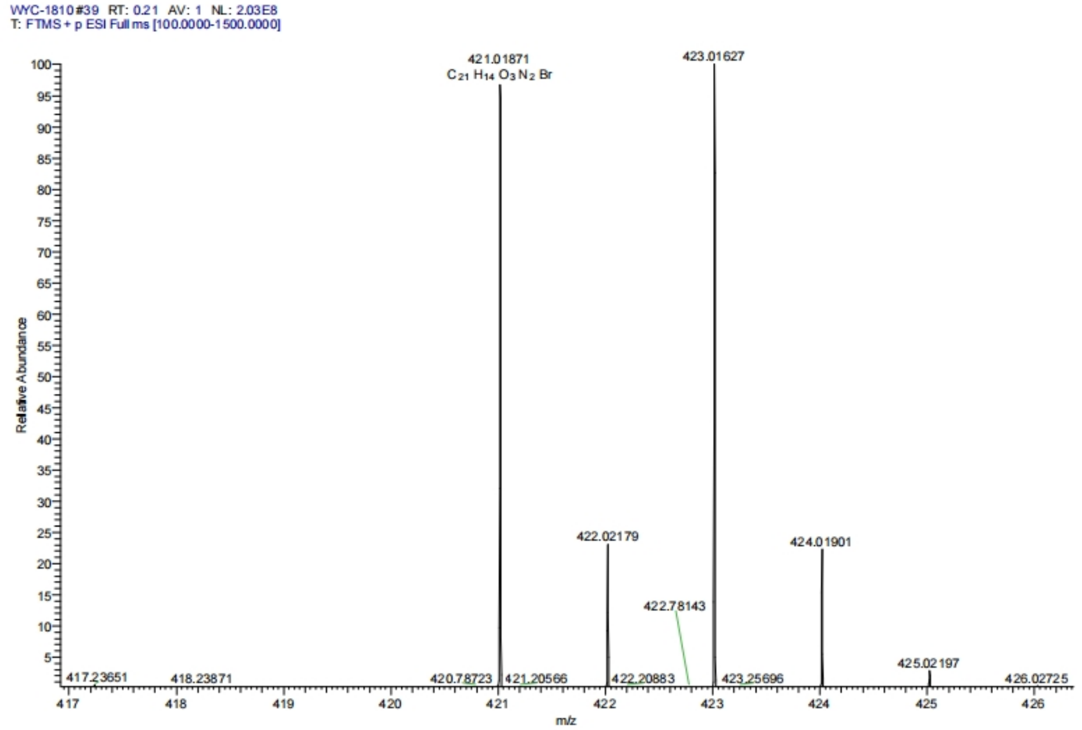

^{1}H-NMR:

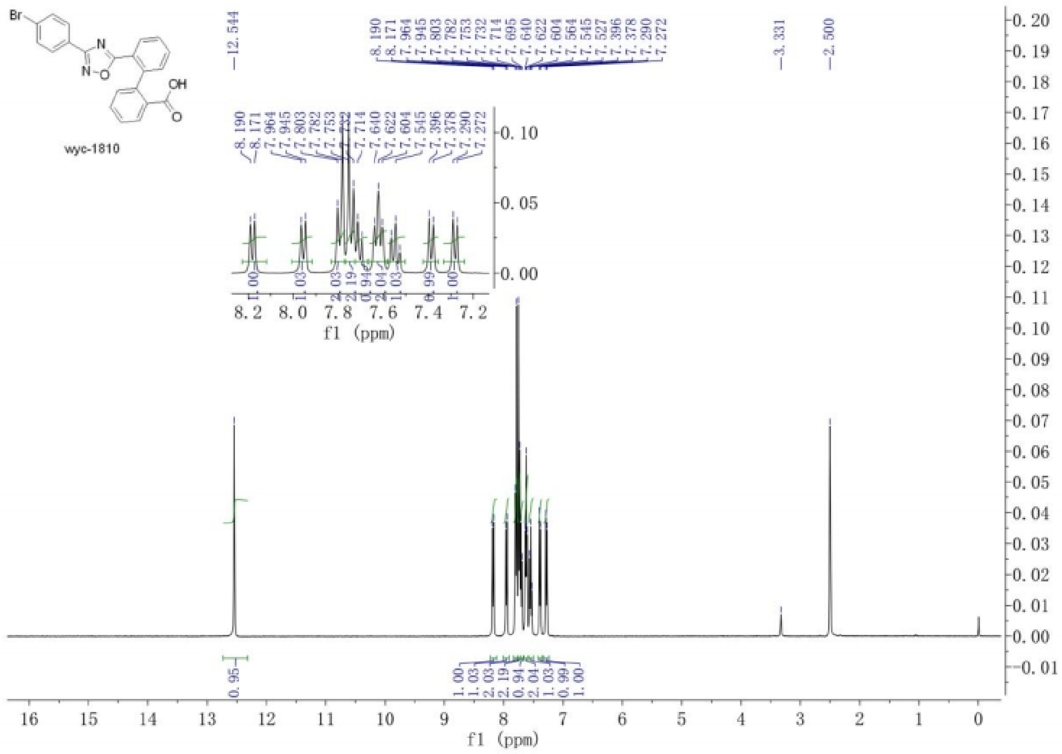

^{13}C-NMR:

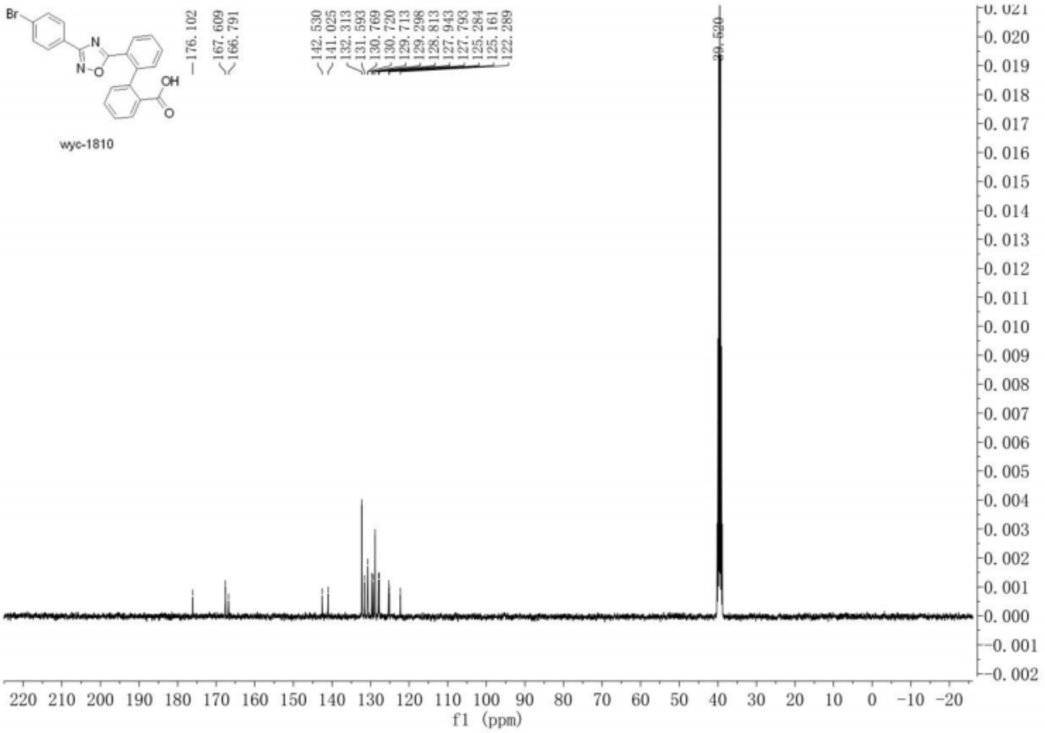

Compound **B5**:

HR-ESI-MS:

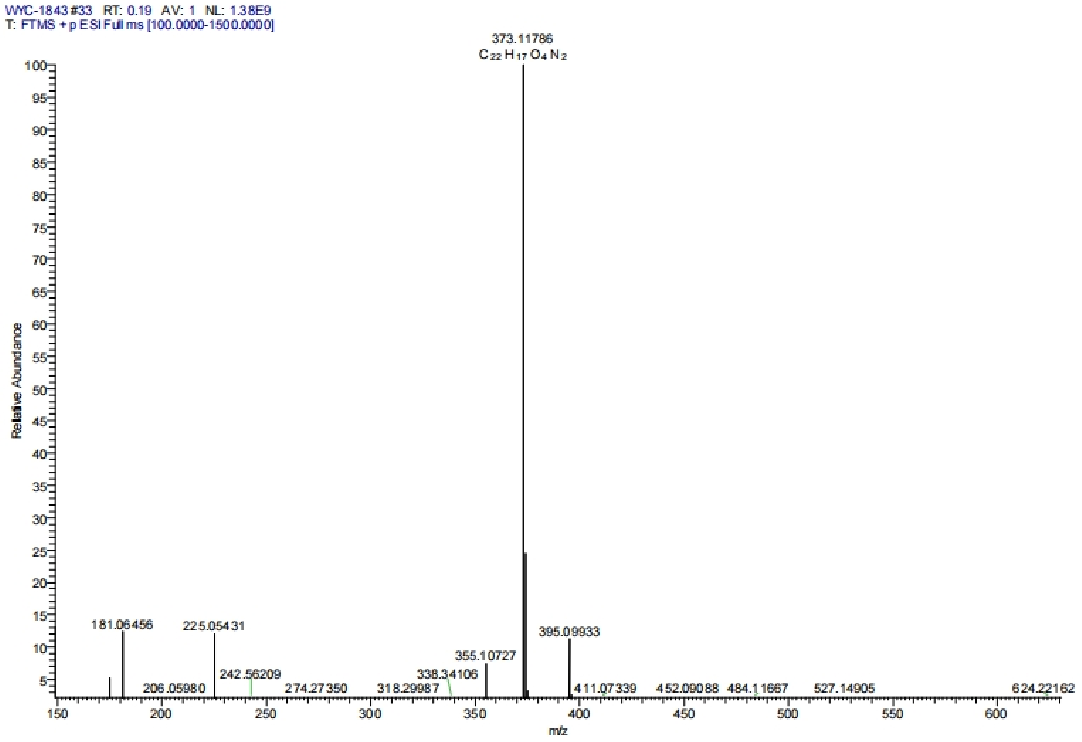

^{1}H-NMR:

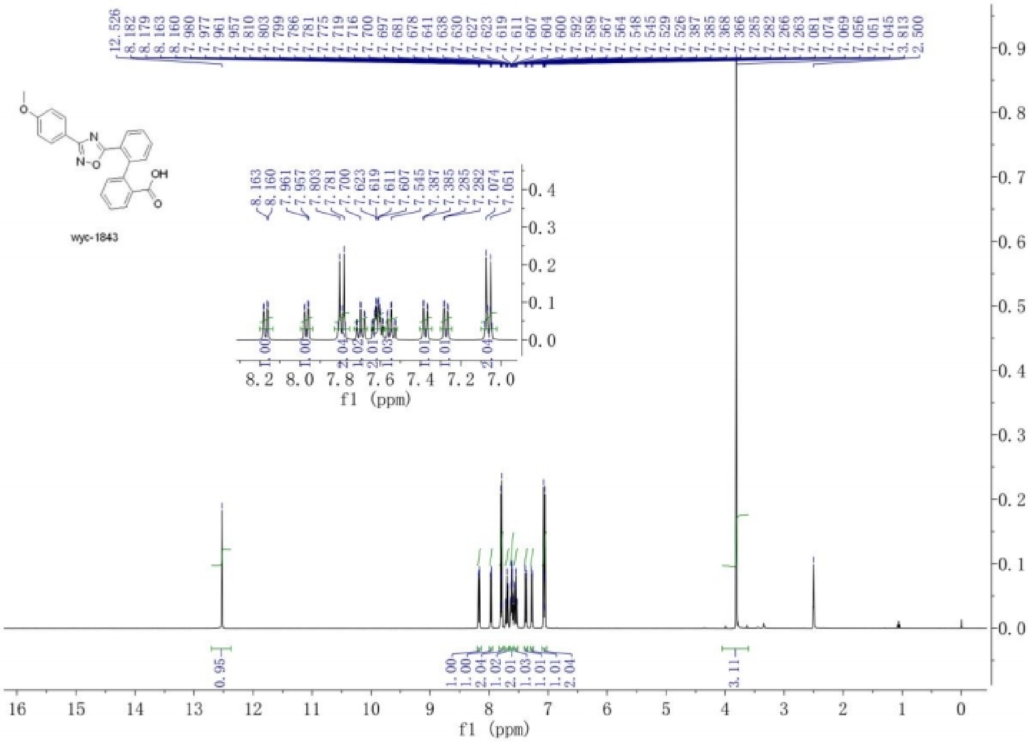

^{13}C-NMR:

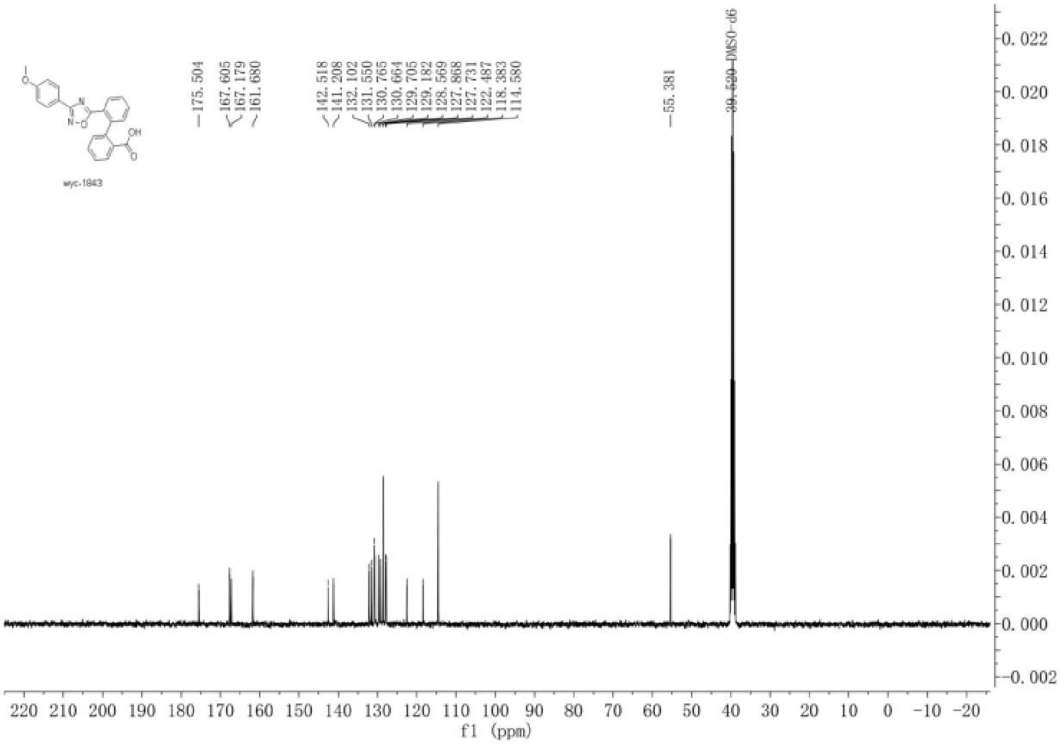

Compound **B6**:

HR-ESI-MS:

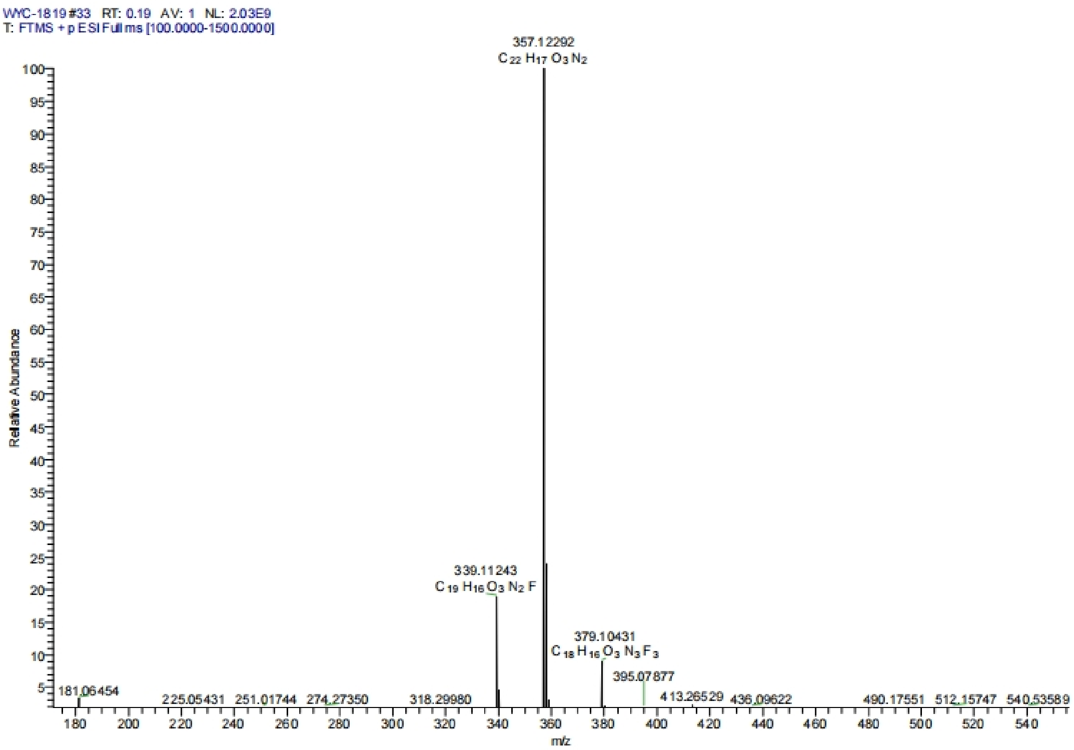

^{1}H-NMR:

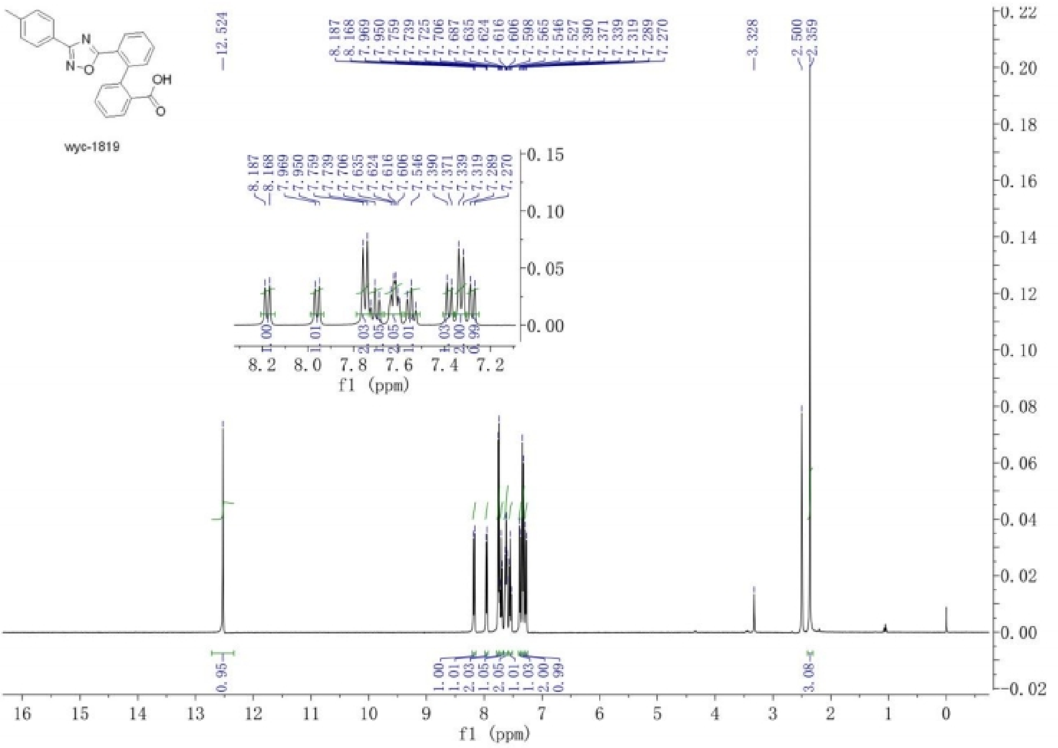

^{13}C-NMR:

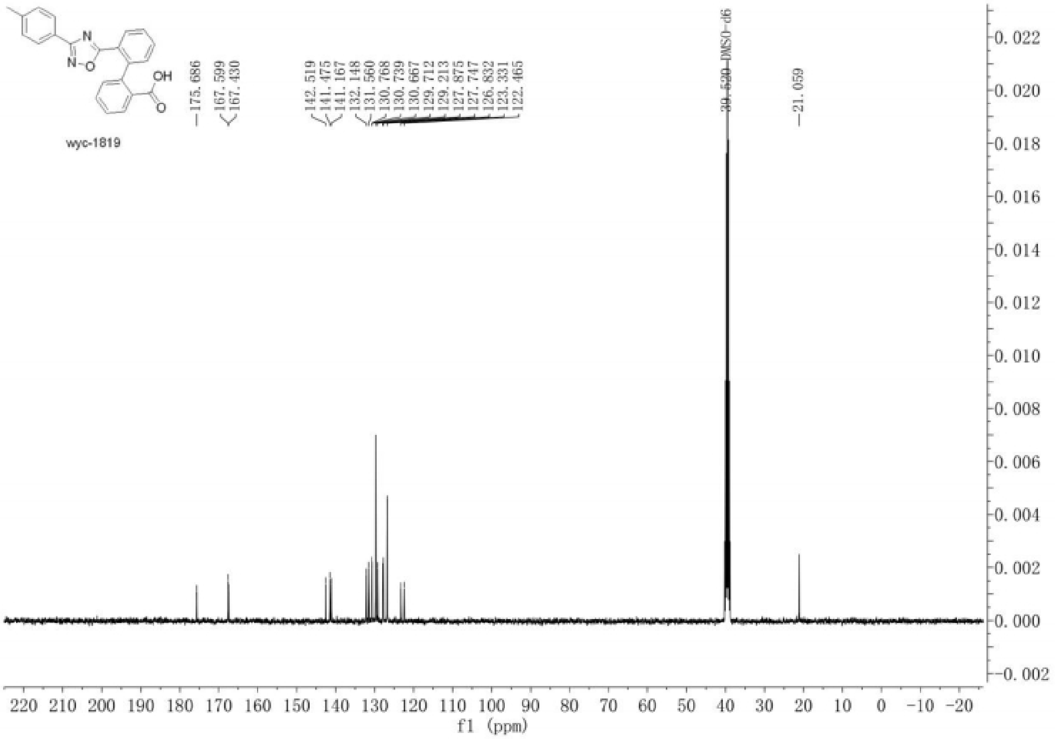

Compound **B7**:

HR-ESI-MS:

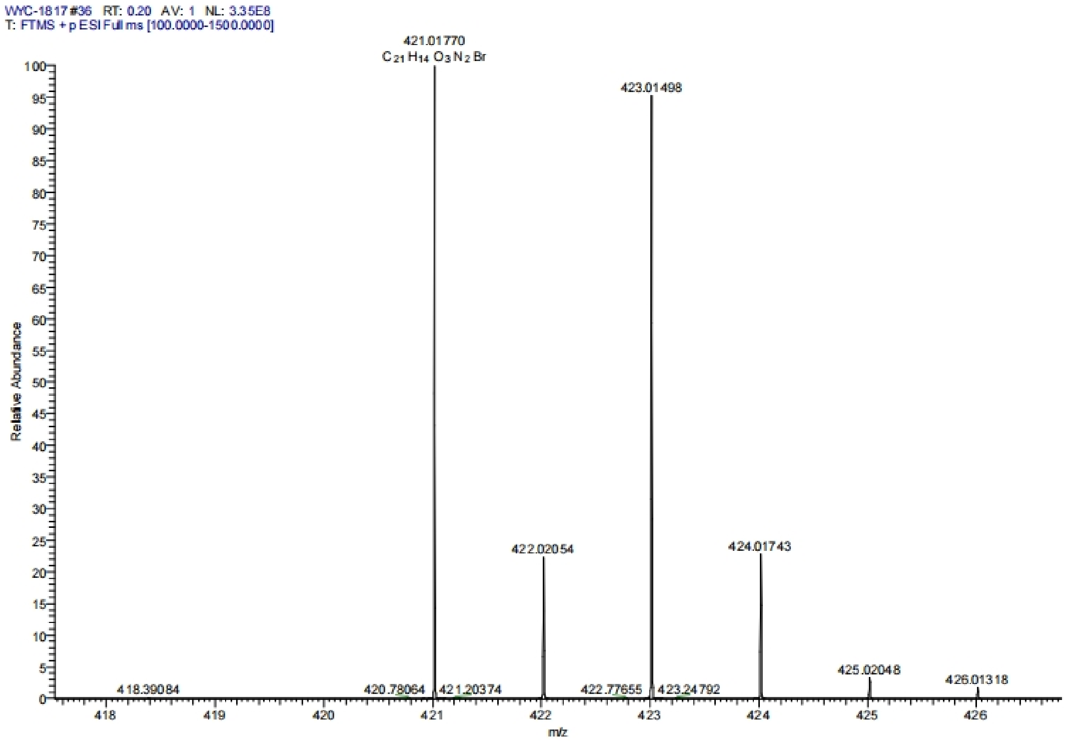

^{1}H-NMR:

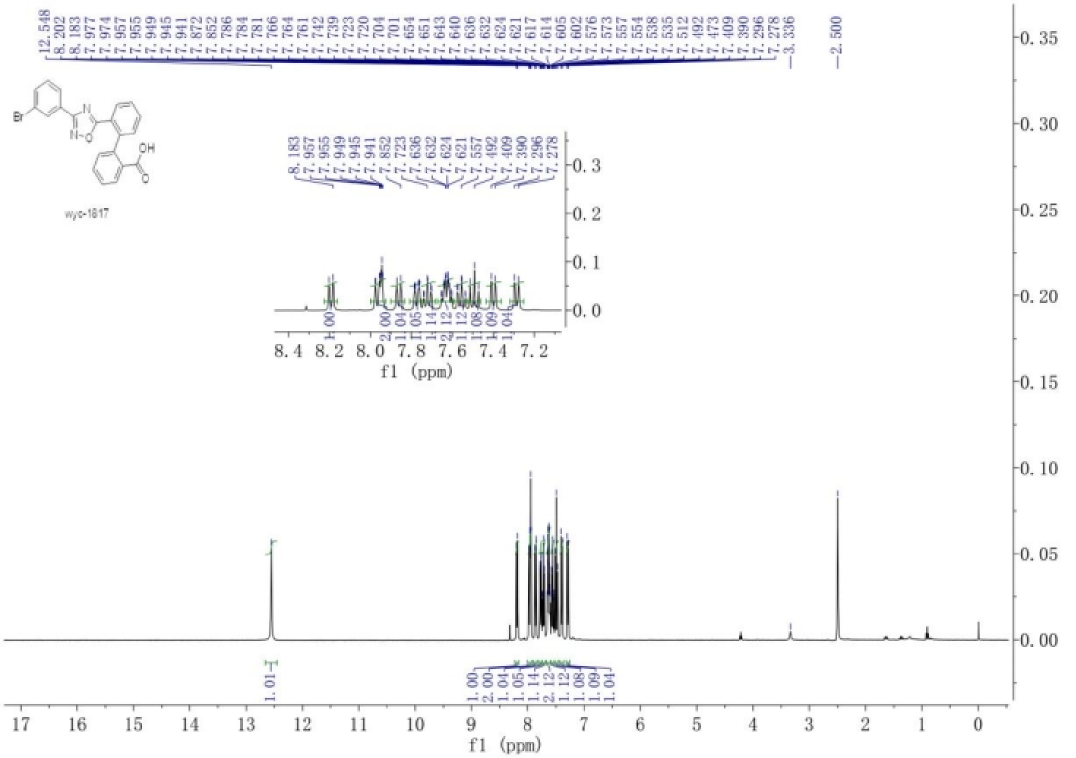

^{13}C-NMR:

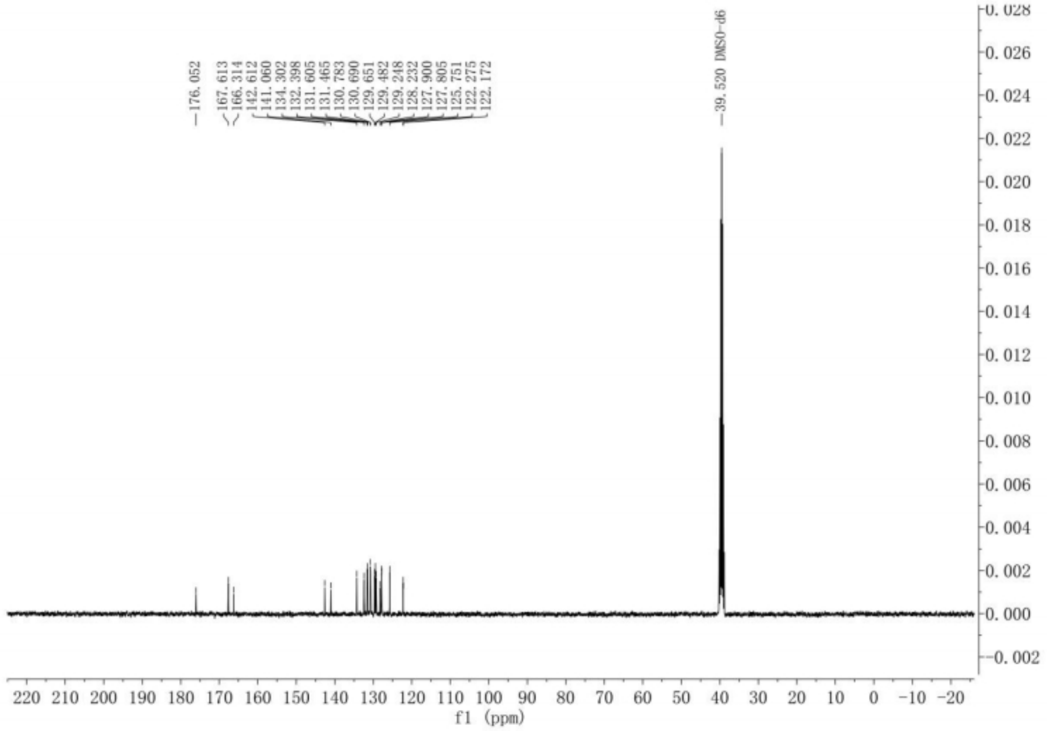

Compound **B8**:

HR-ESI-MS:

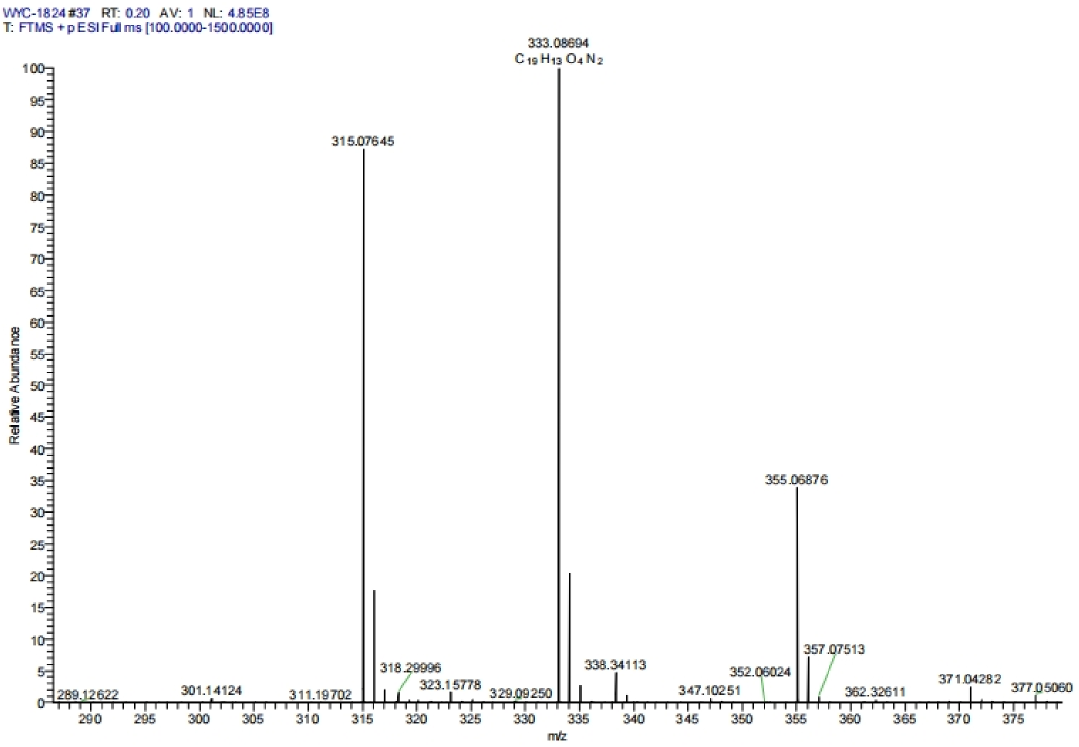

^{1}H-NMR:

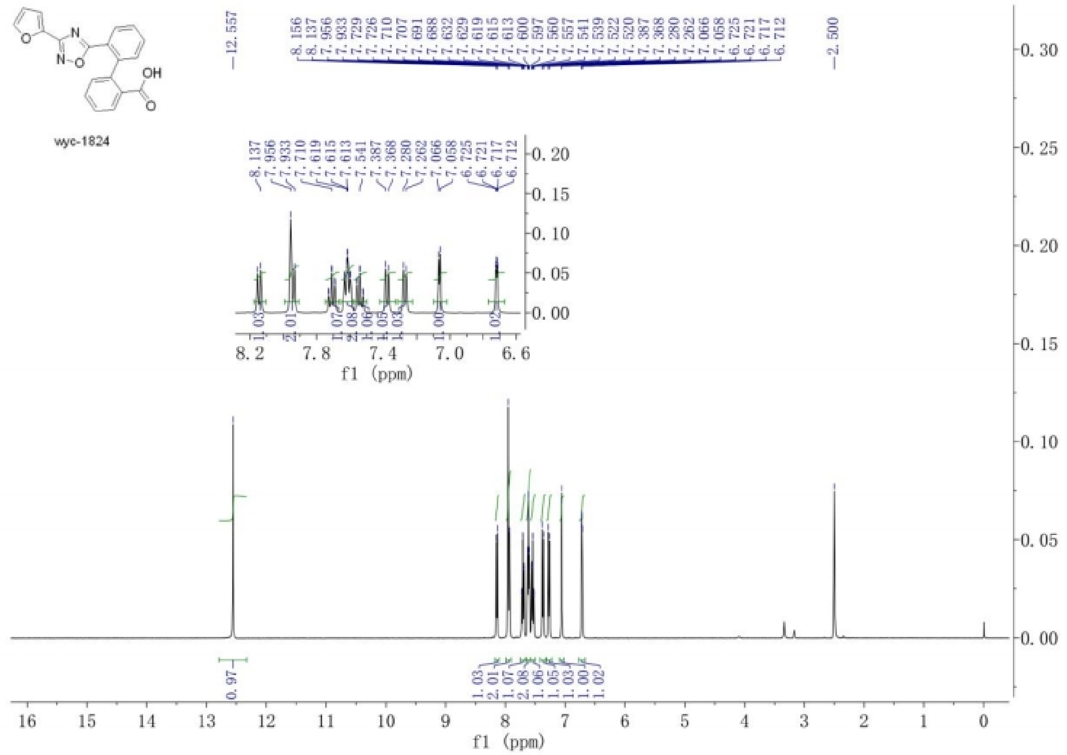

^{13}C-NMR:

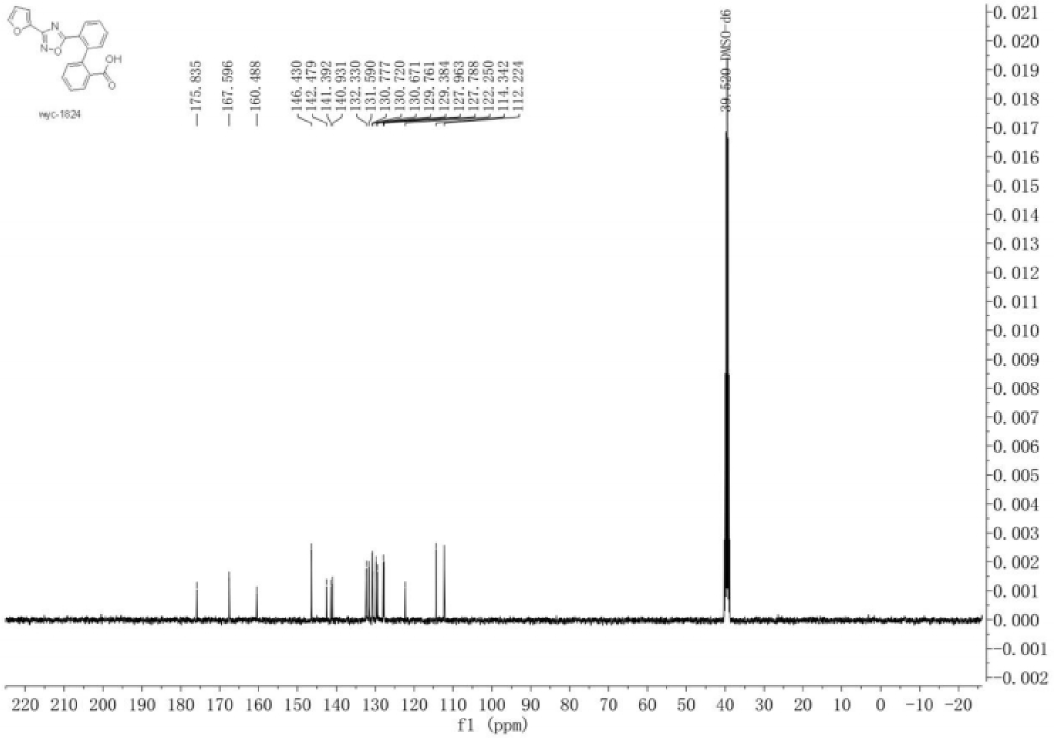

Compound **B9**:

HR-ESI-MS:

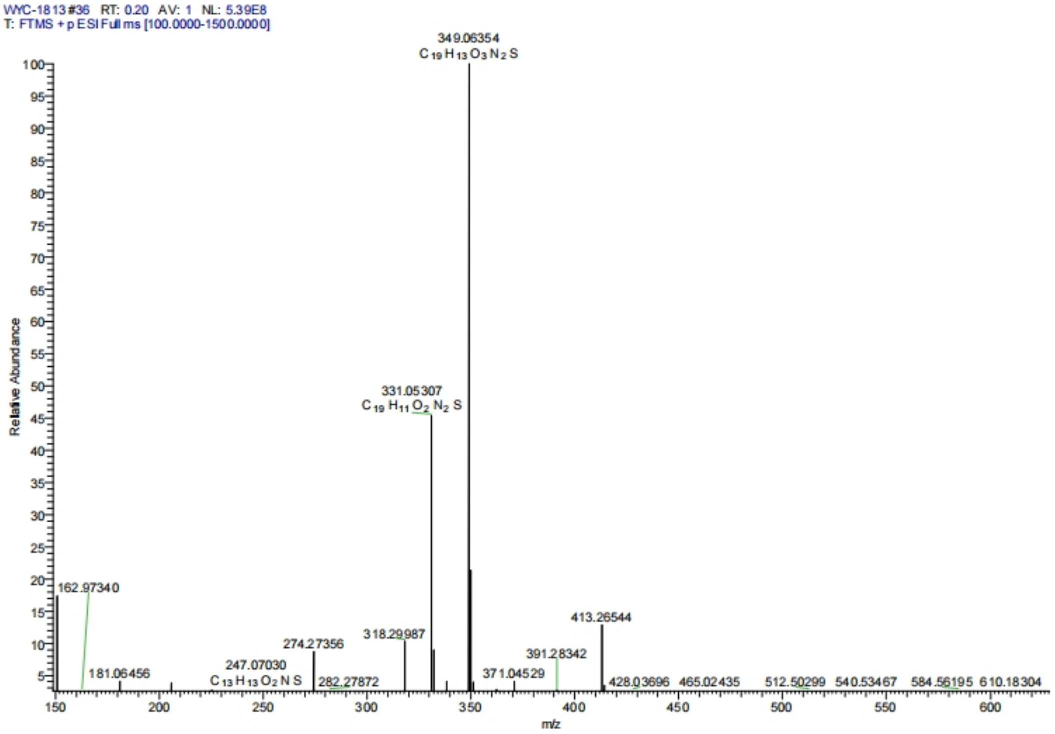

^{1}H-NMR:

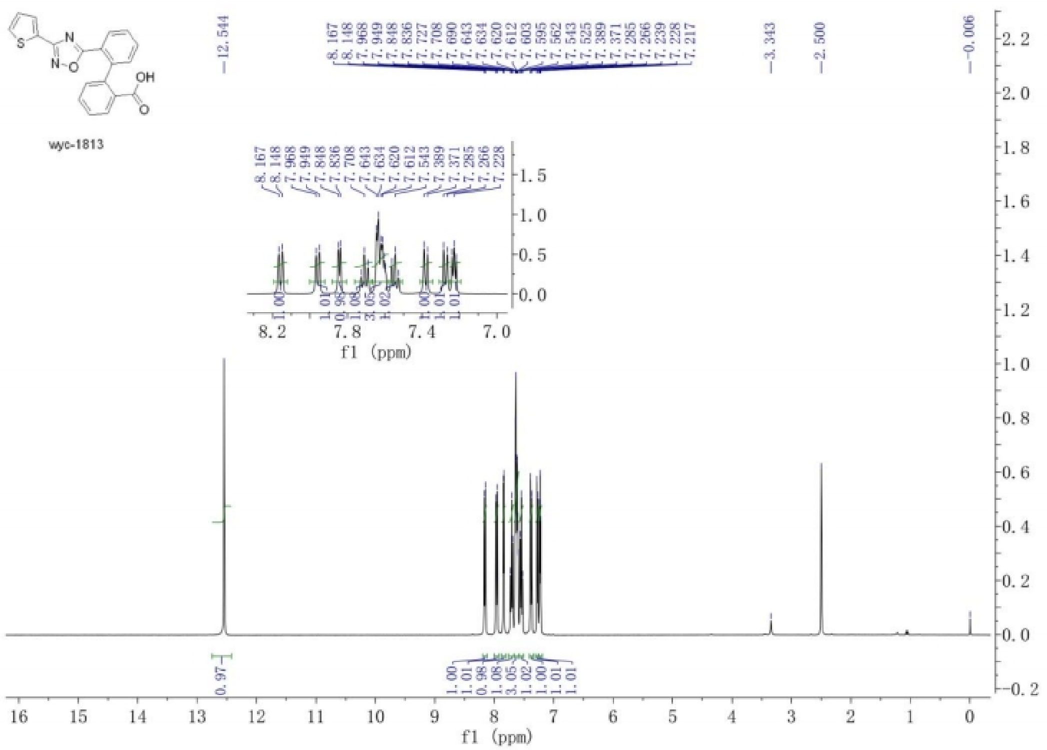

^{13}C-NMR:

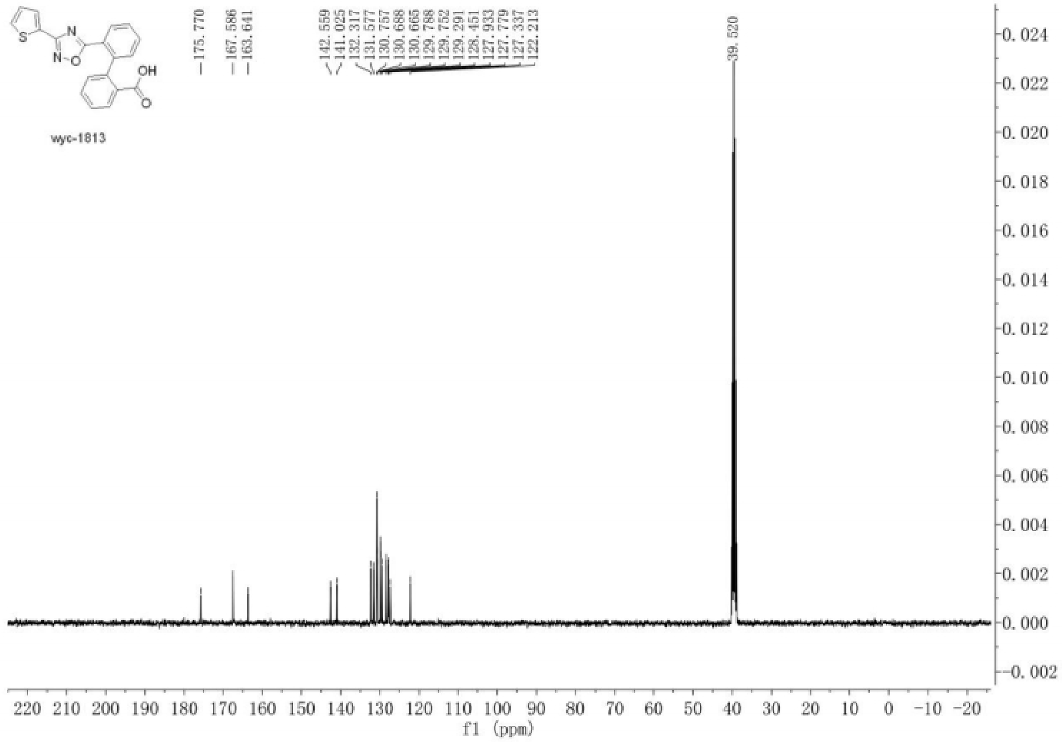

Compound **B10**:

HR-ESI-MS:

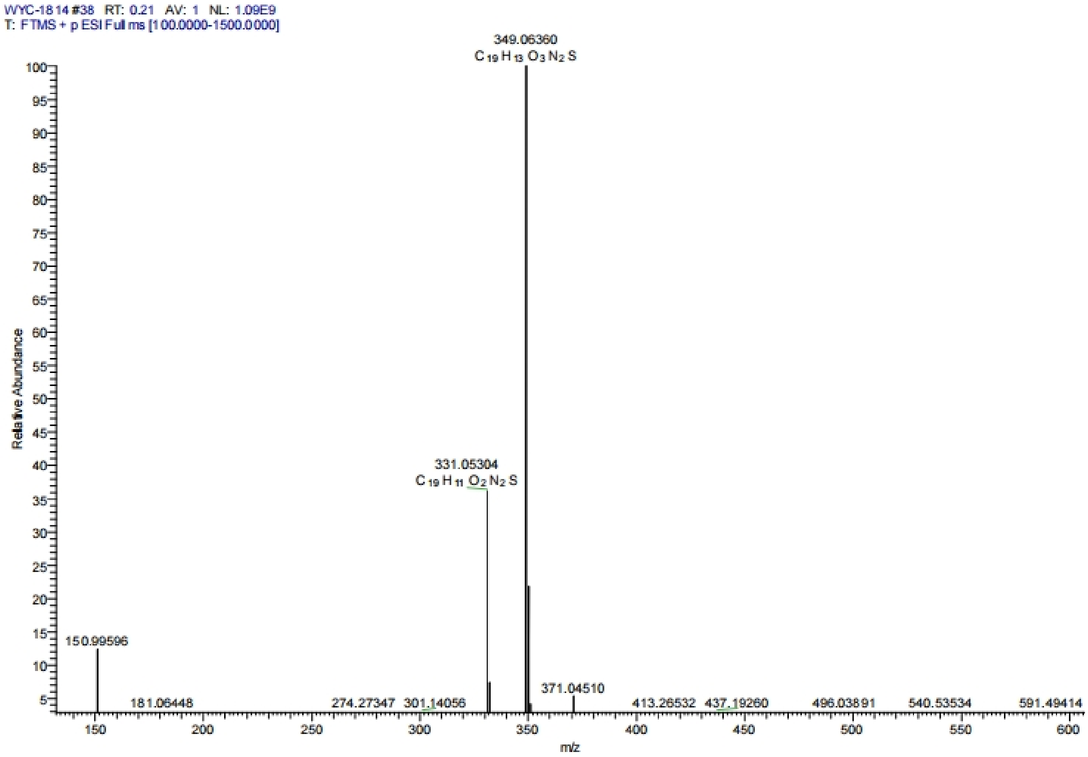

^{1}H-NMR:

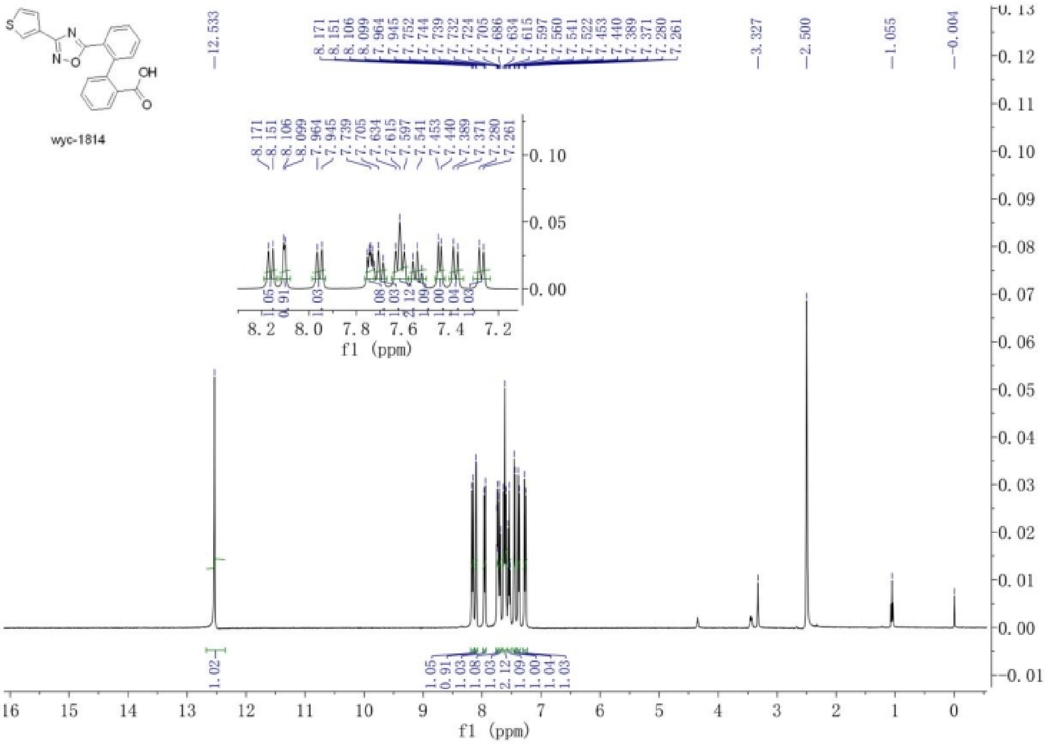

^{13}C-NMR:

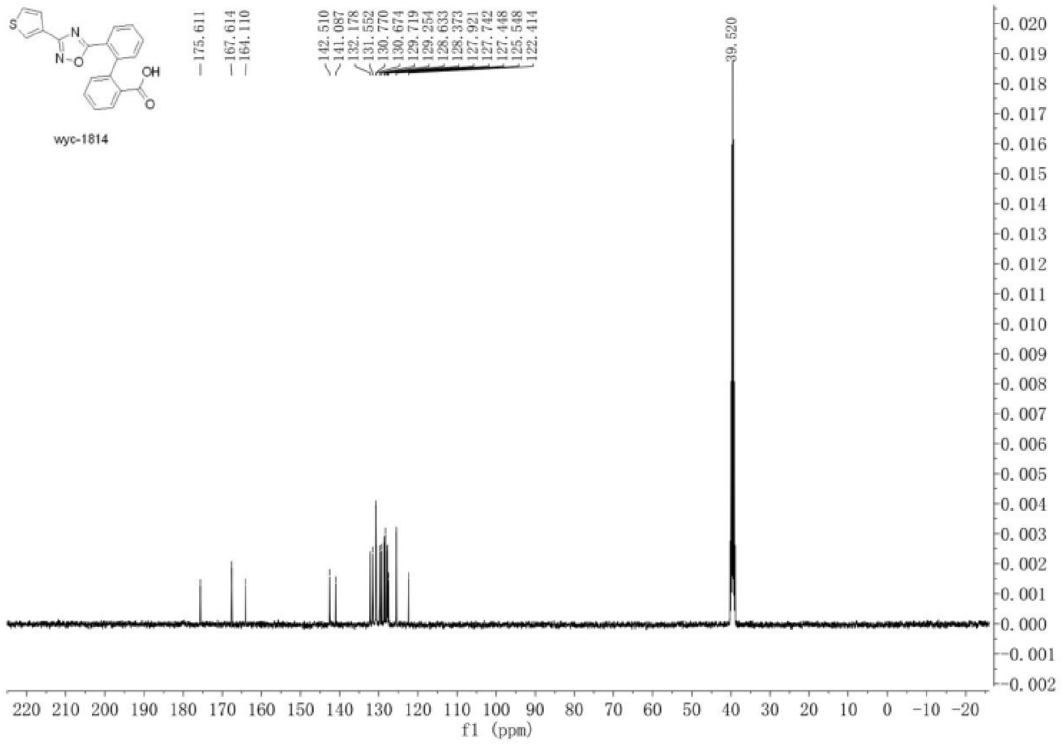

Compound **B11**:

HR-ESI-MS:

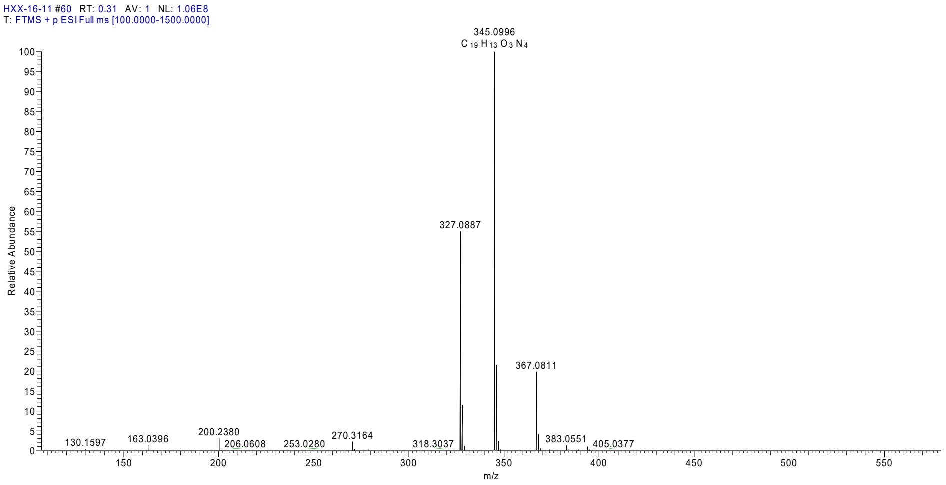

^{1}H-NMR:

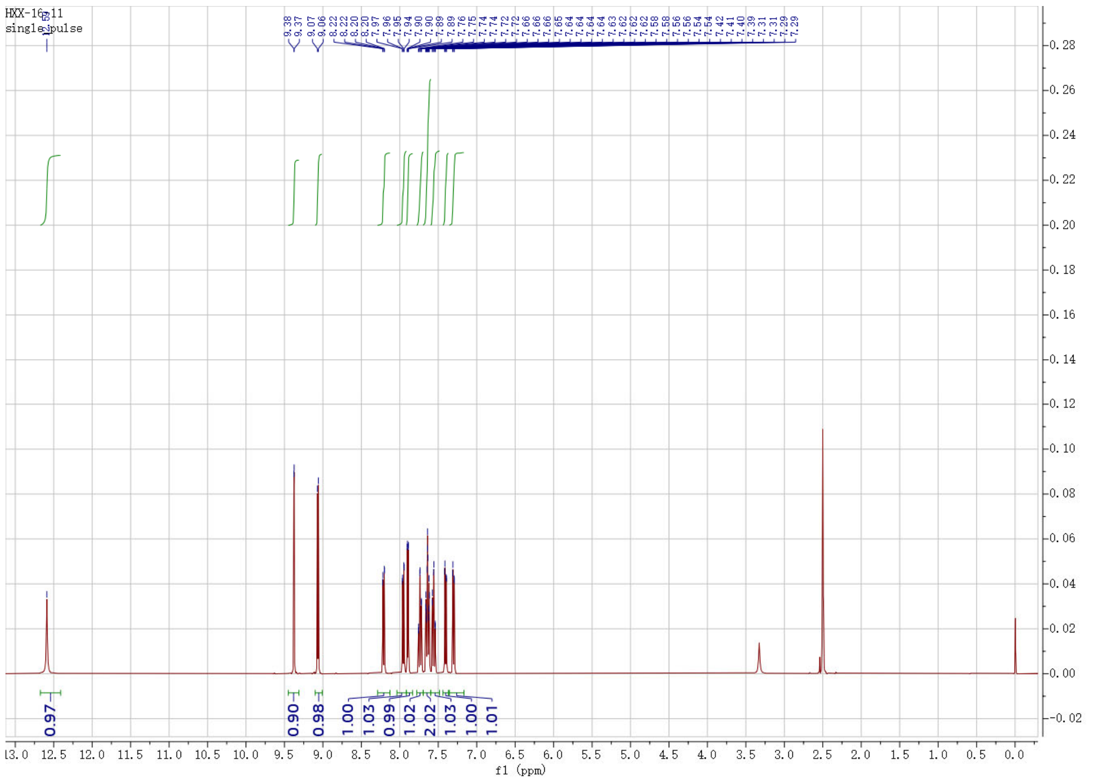

^{13}C-NMR:

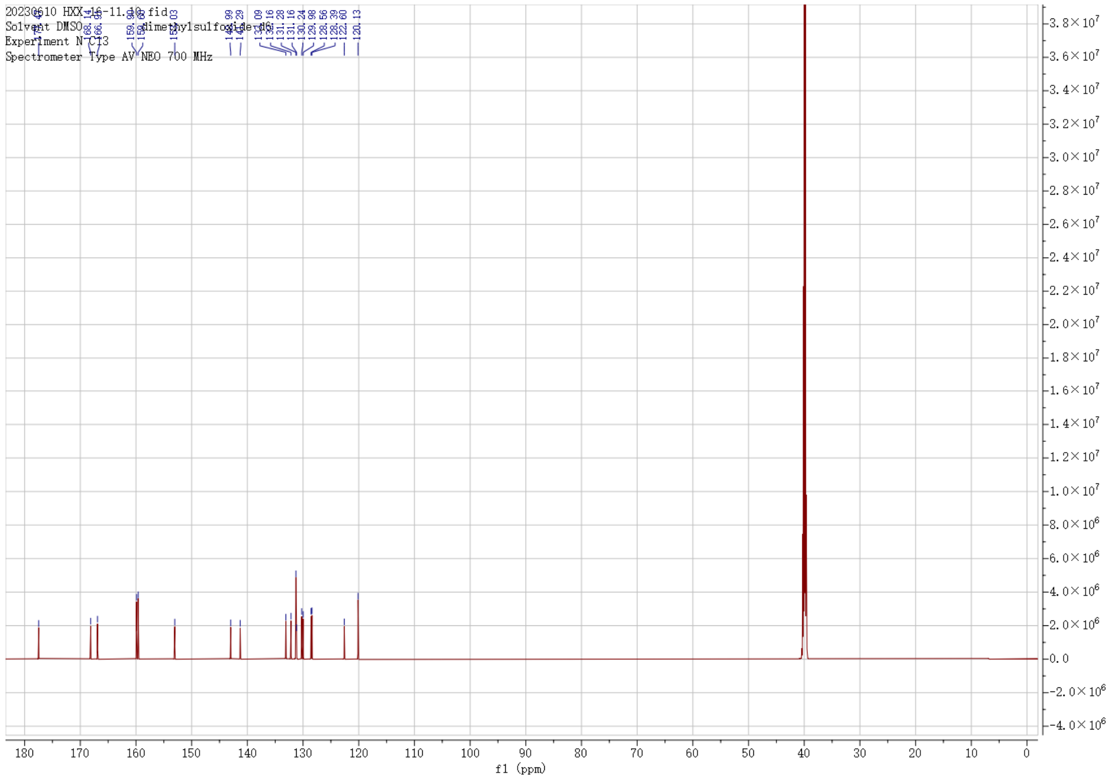

Compound **B12**:

HR-ESI-MS:

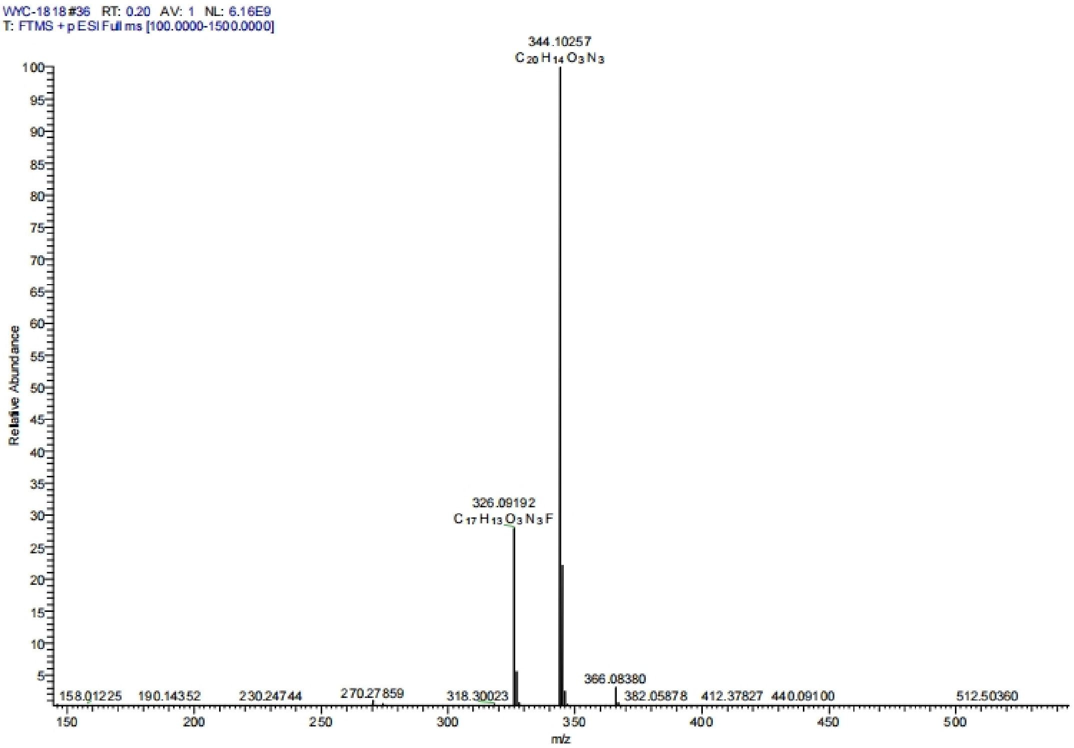

^{1}H-NMR:

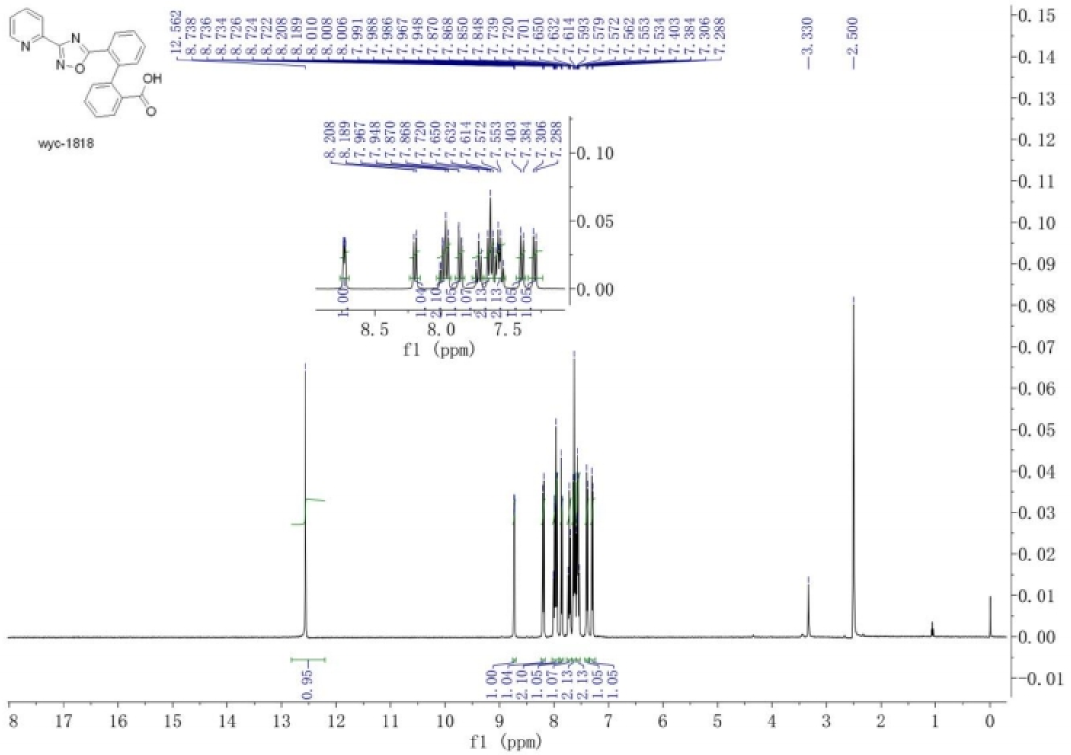

^{13}C-NMR:

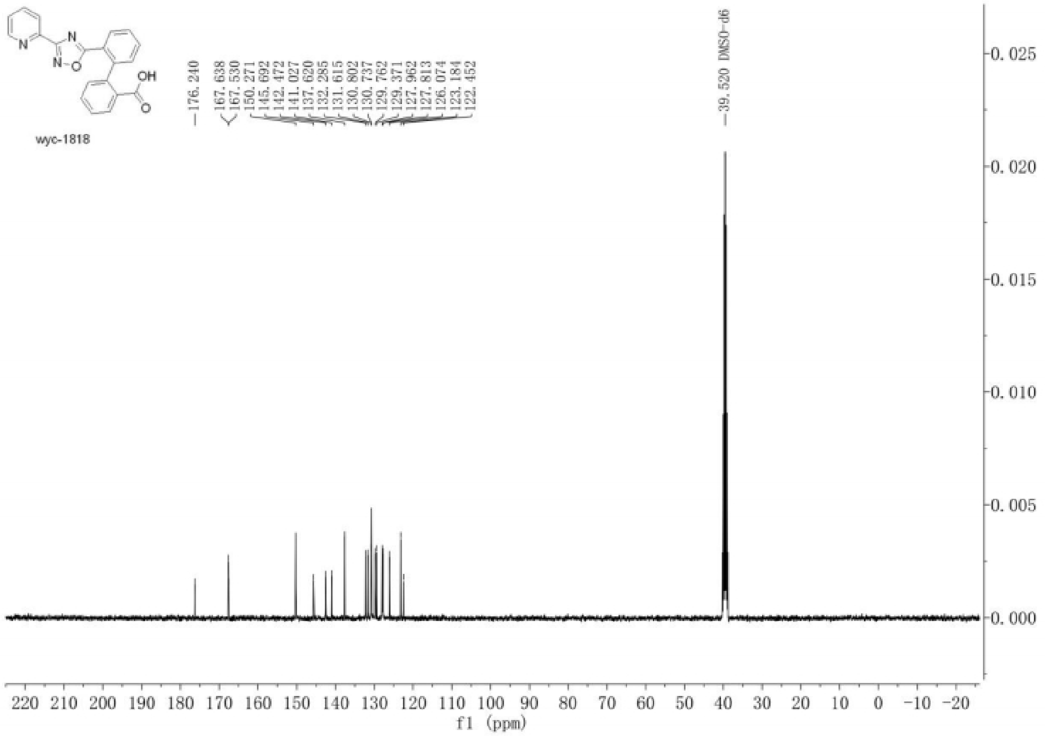

Compound **B13**:

HR-ESI-MS:

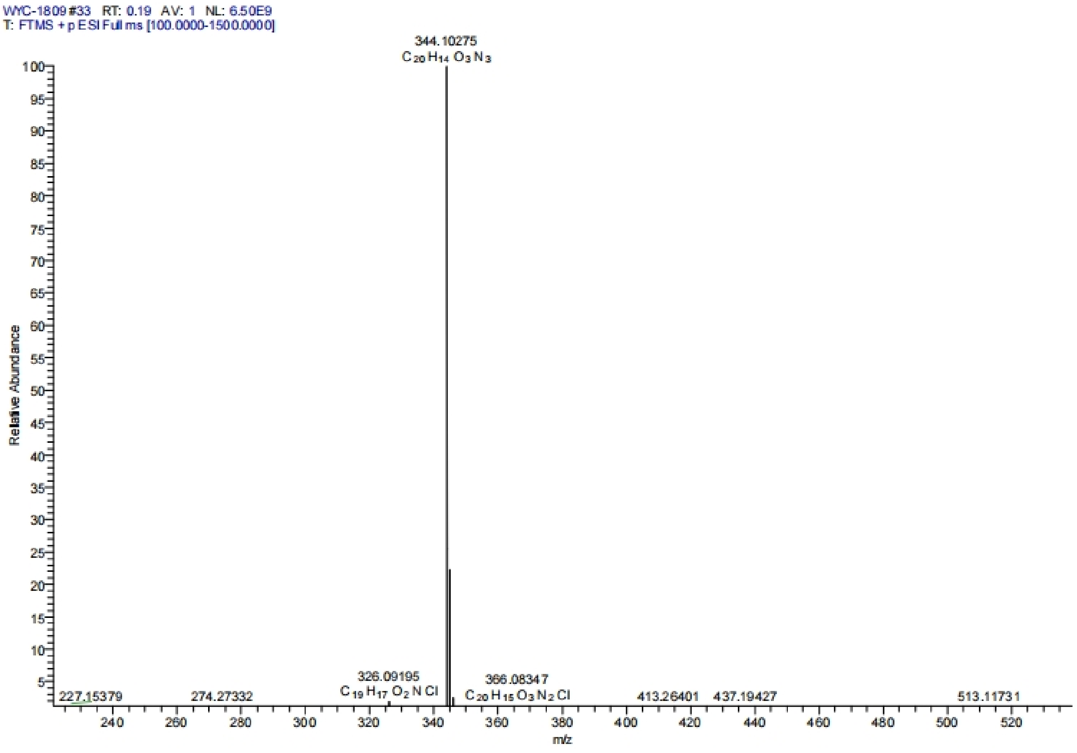

^{1}H-NMR:

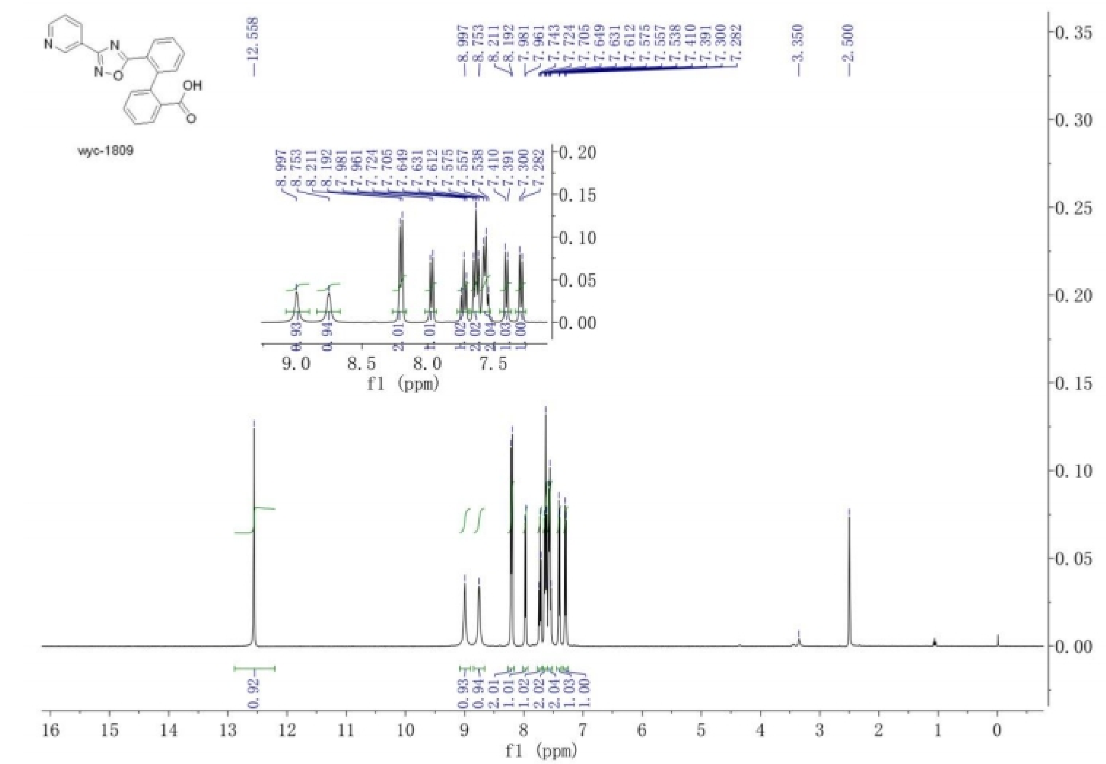

^{13}C-NMR:

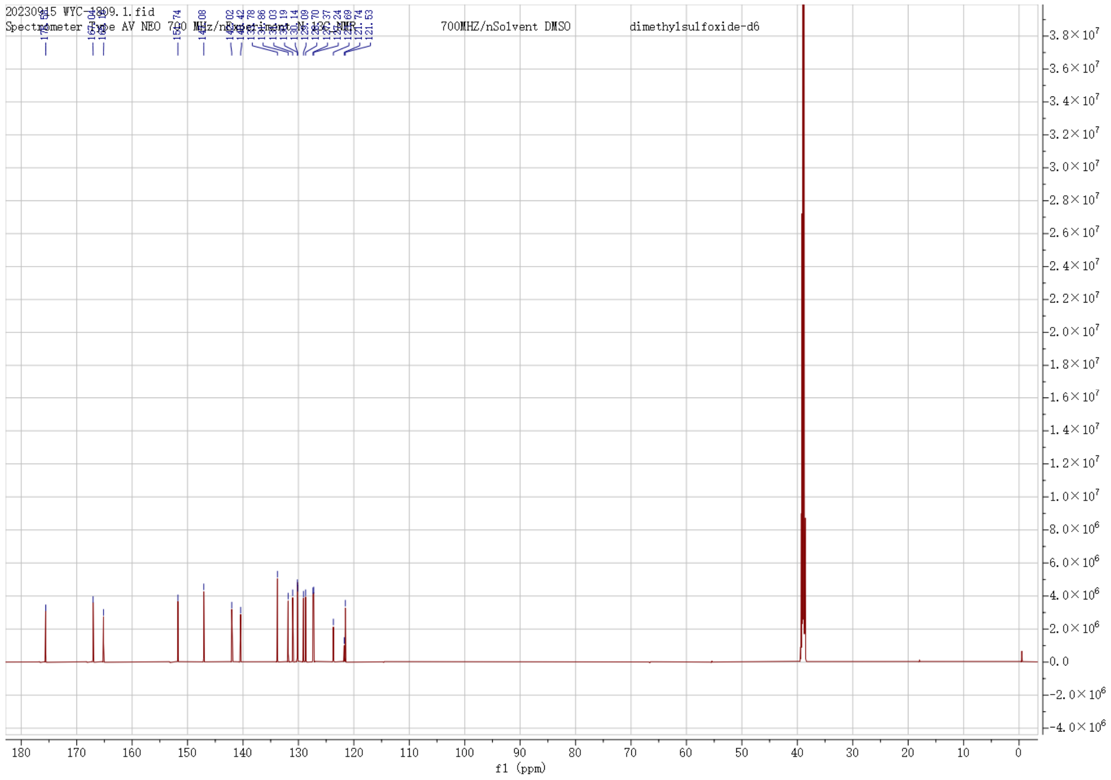

Compound **B14**:

HR-ESI-MS:

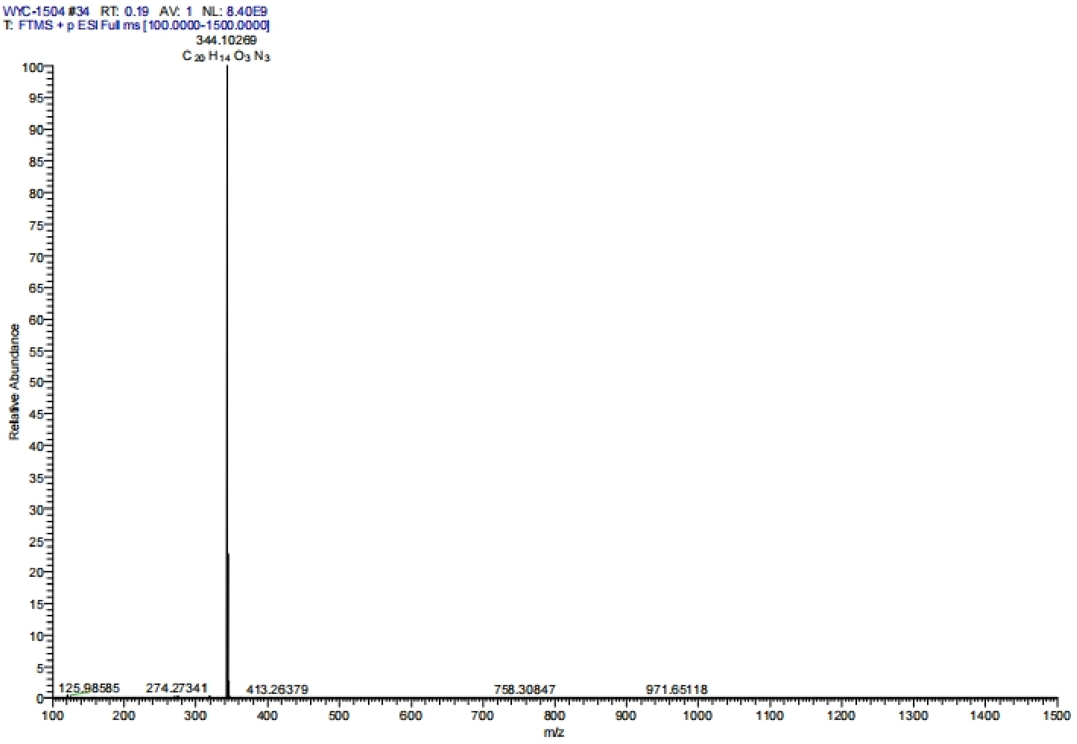

^{1}H-NMR:

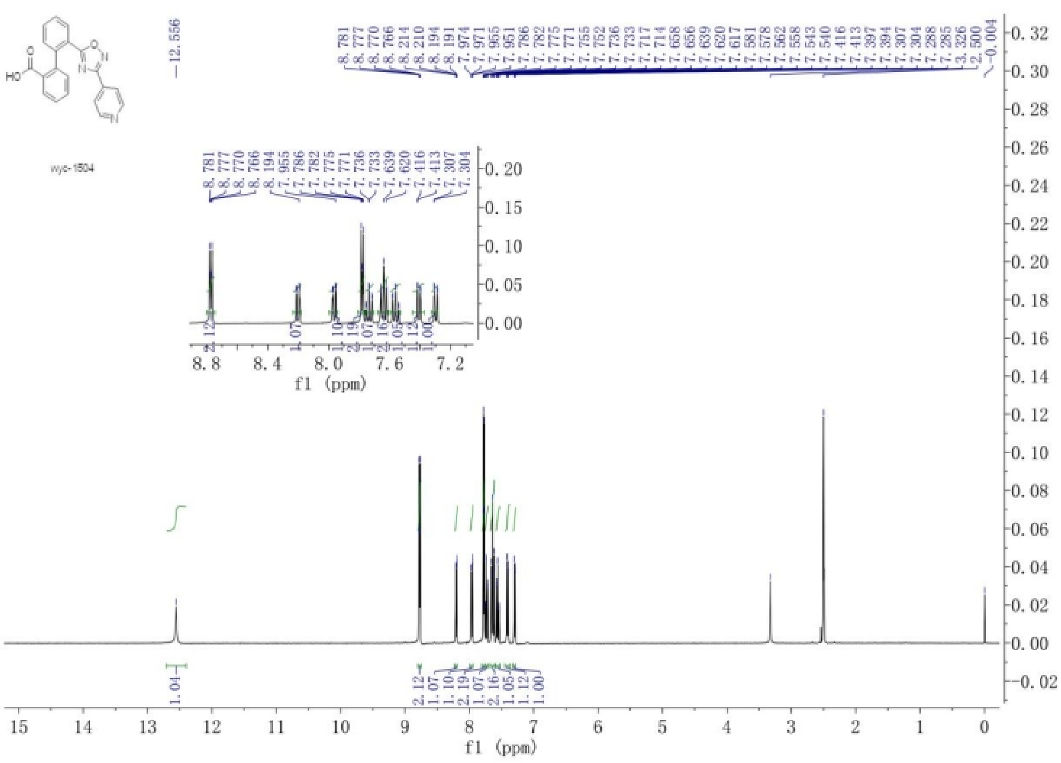

^{13}C-NMR:

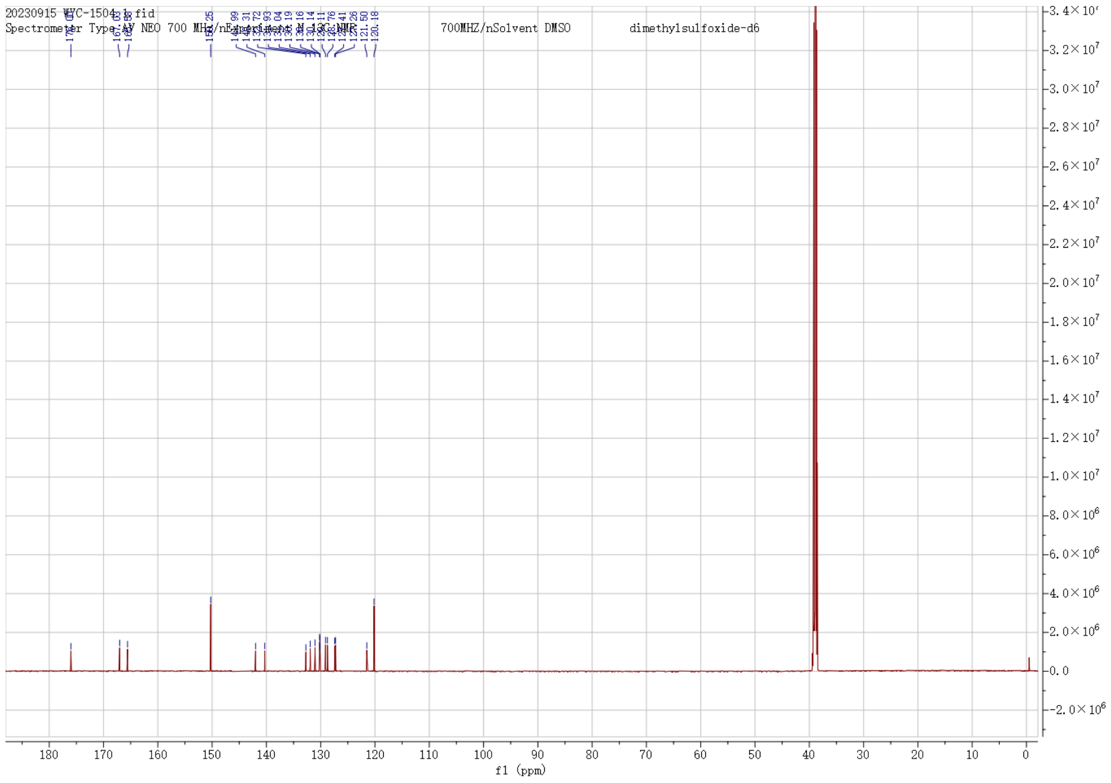

Compound **B15**:

HR-ESI-MS:

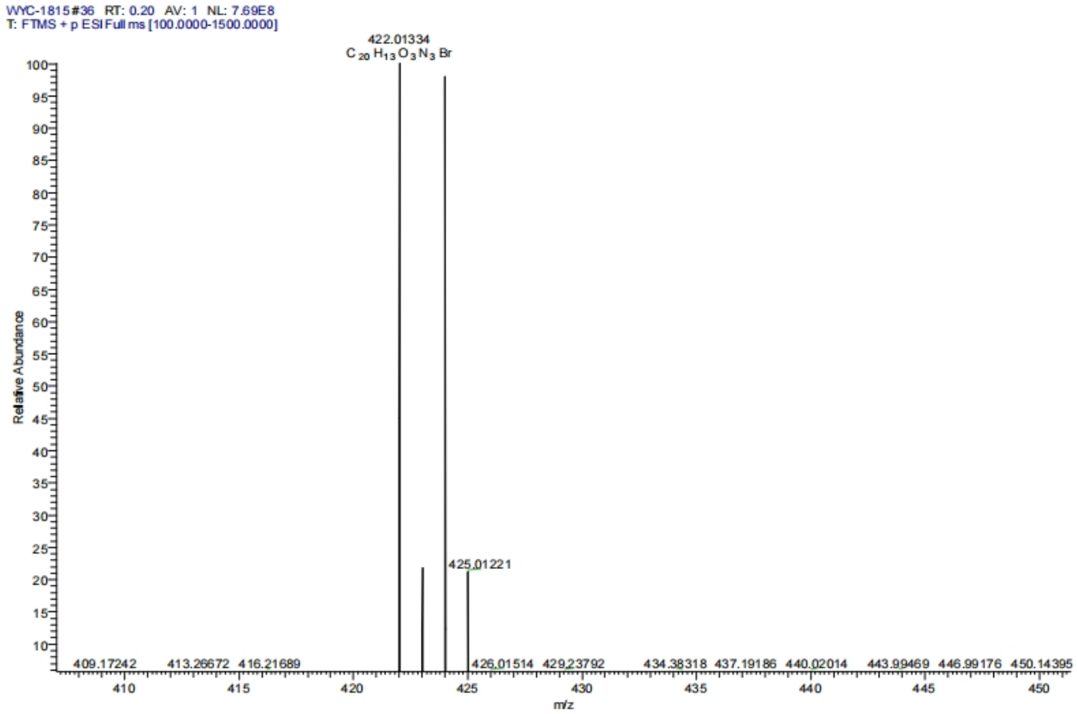

^{1}H-NMR:

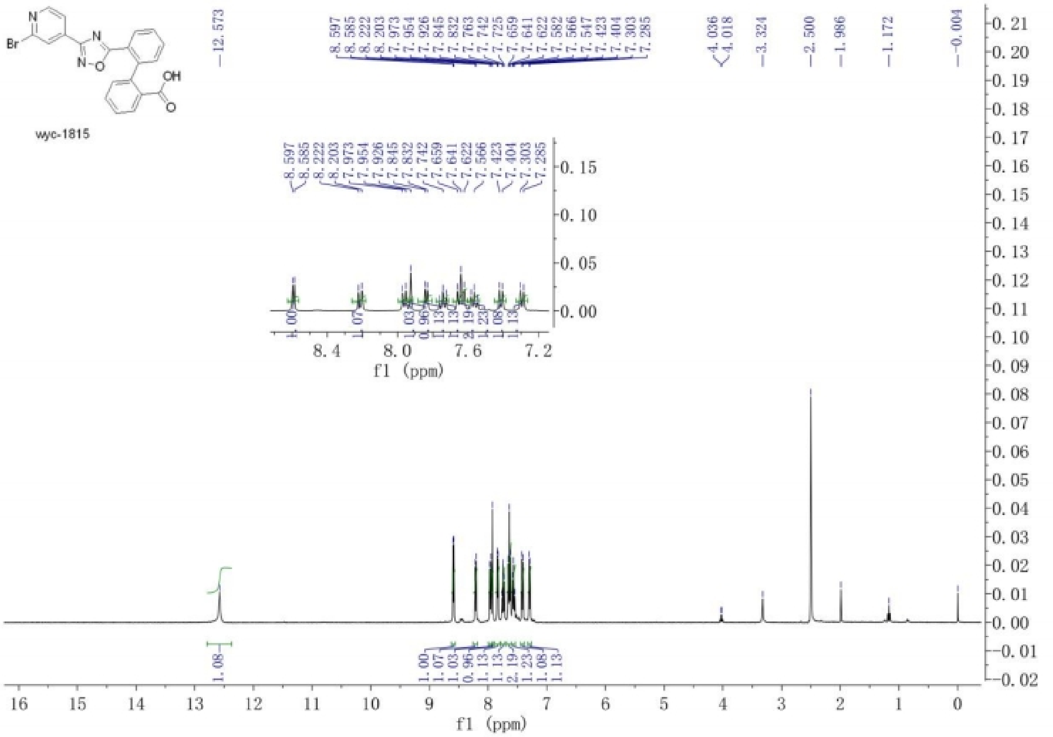

^{13}C-NMR:

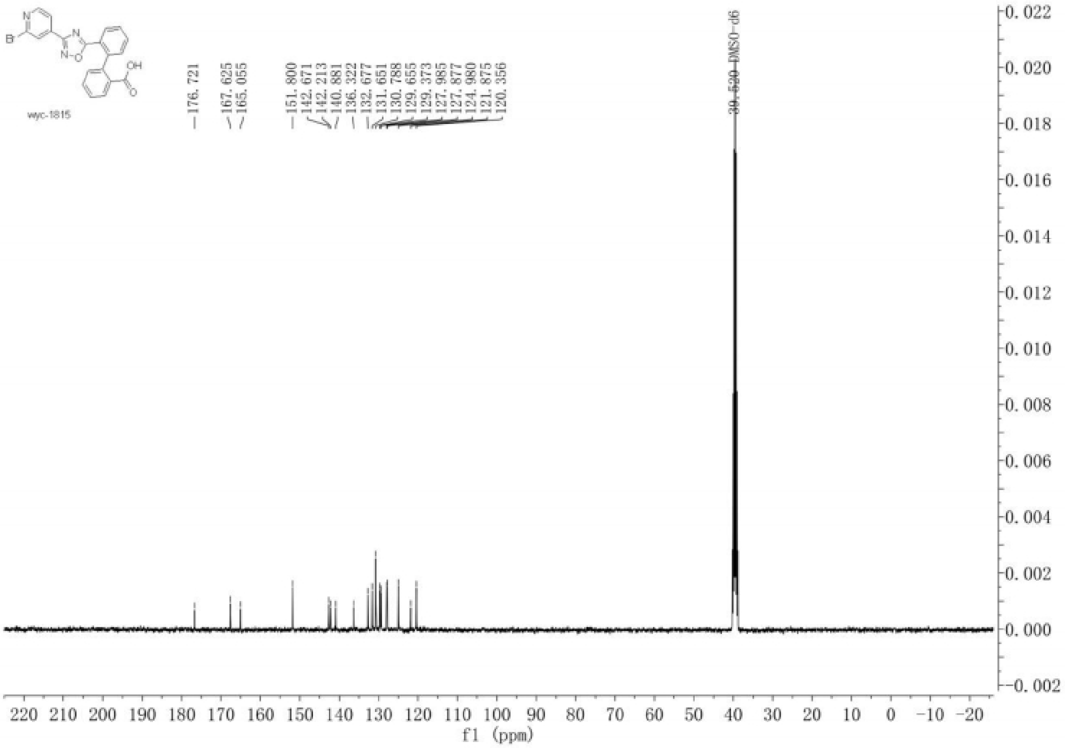

Compound **B16**:

HR-ESI-MS:

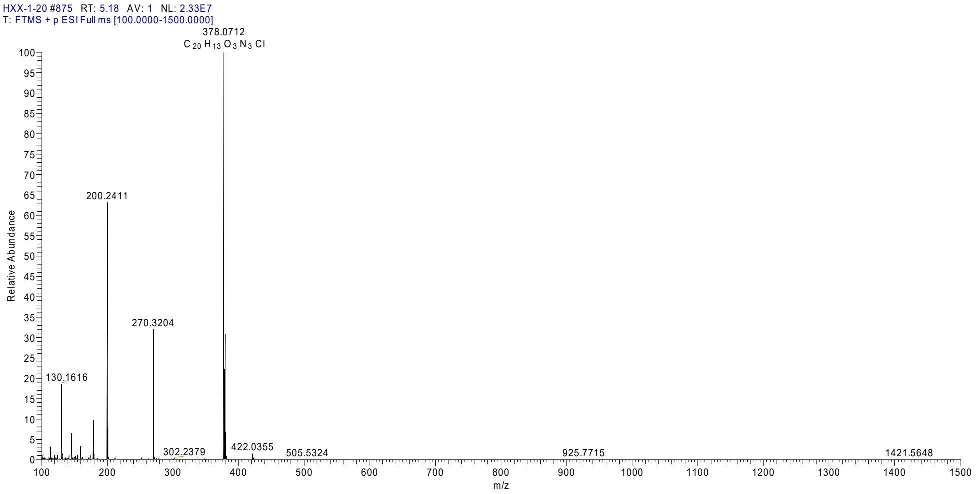

^{1}H-NMR:

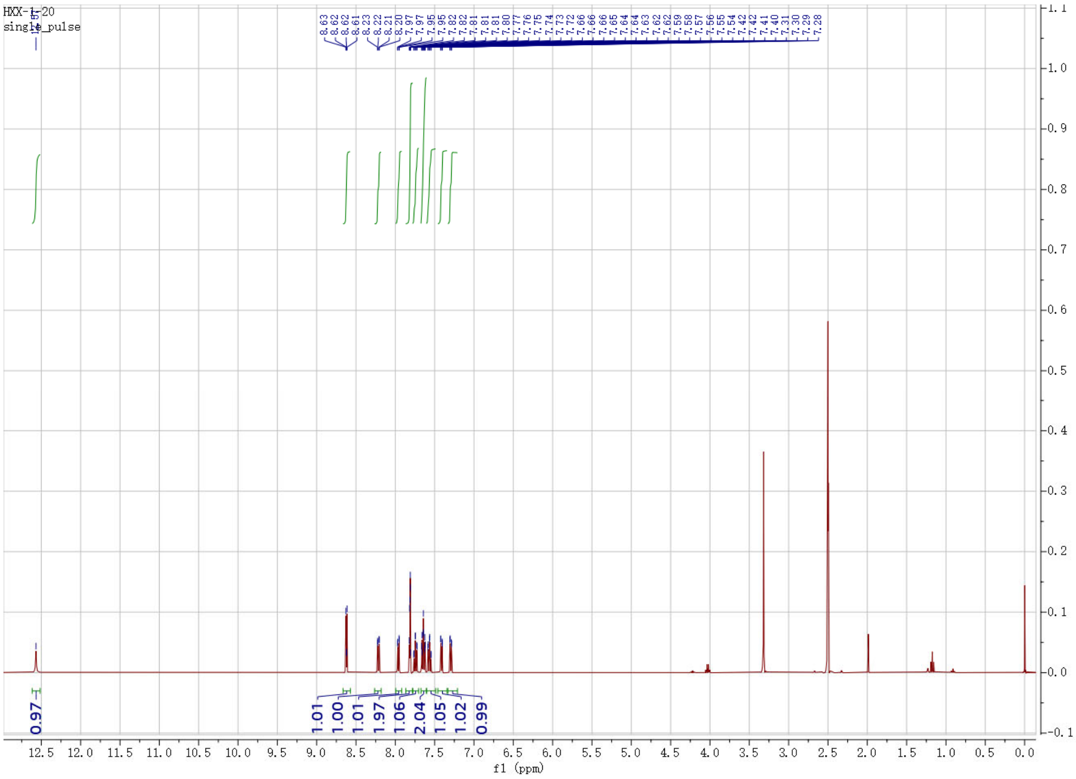

^{13}C-NMR:

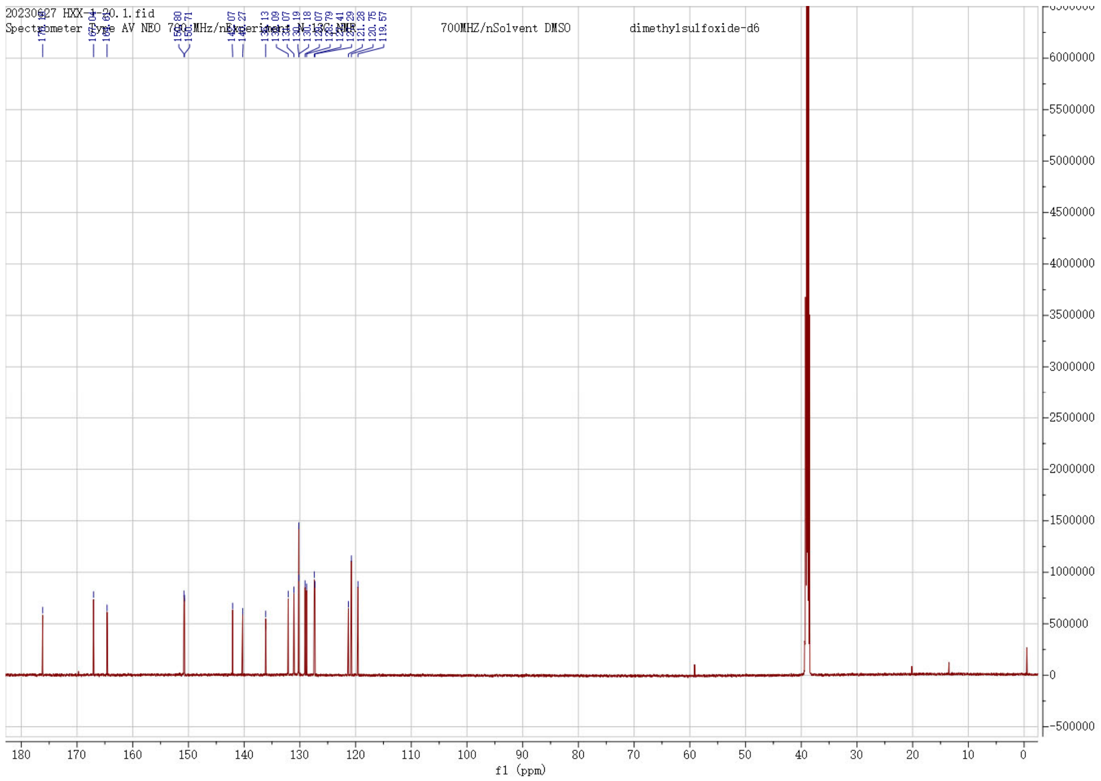

Compound **B17**:

HR-ESI-MS:

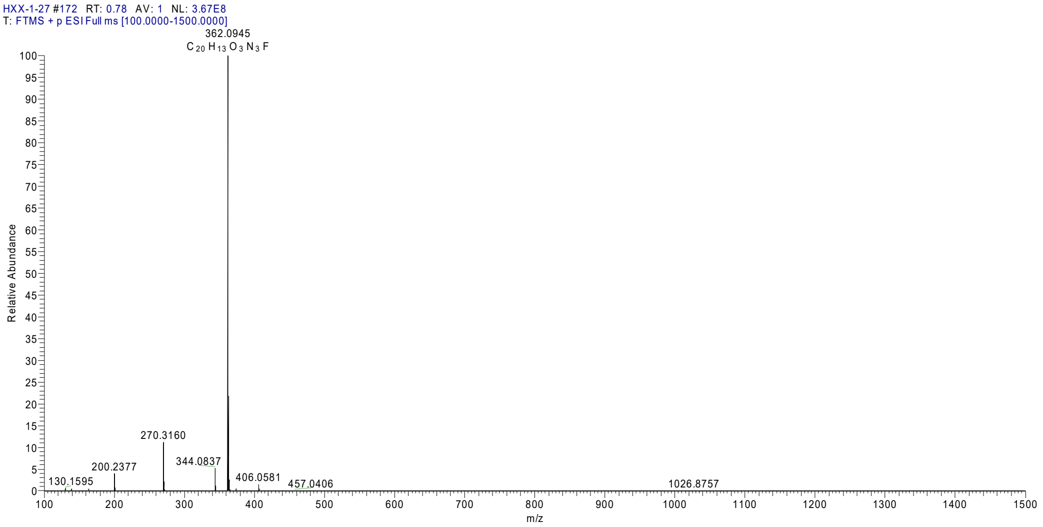

^{1}H-NMR:

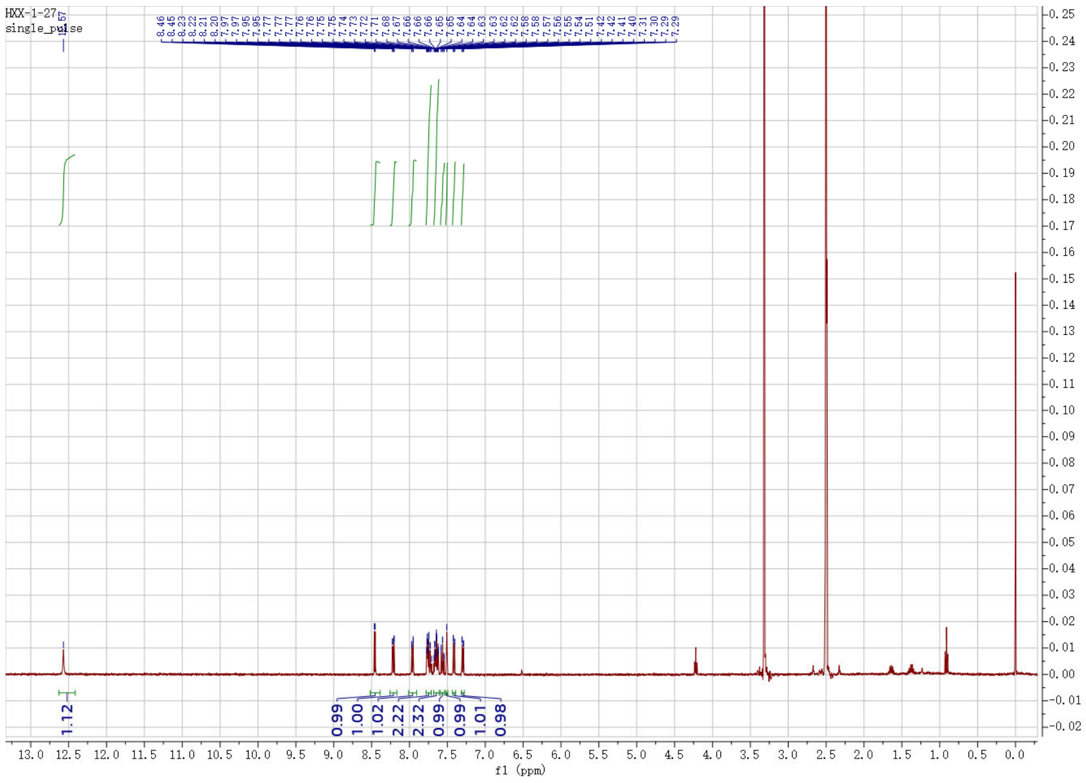

^{13}C-NMR:

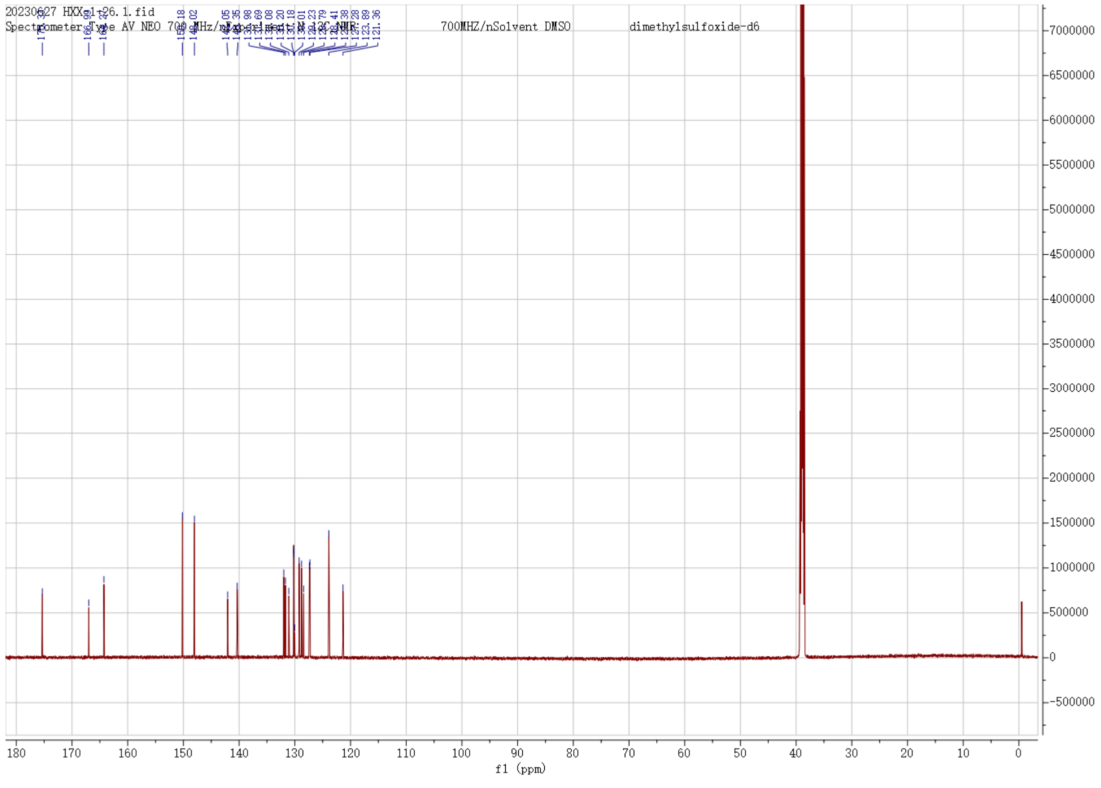

Compound **B18**:

HR-ESI-MS:

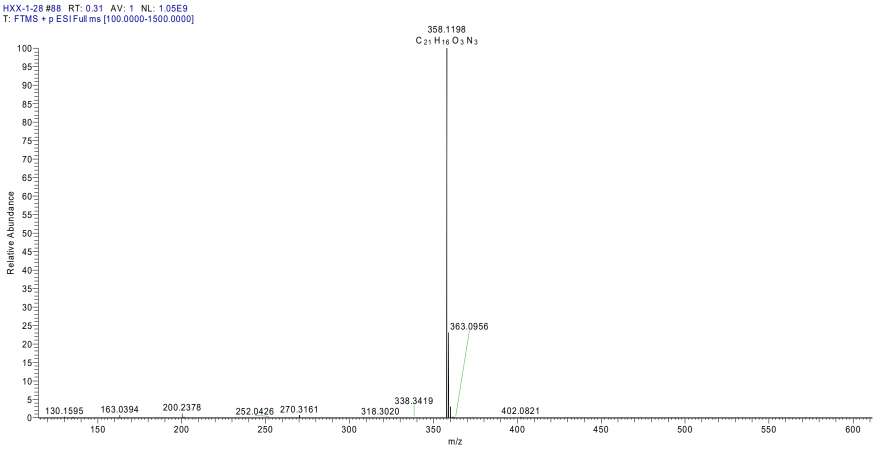

^{1}H-NMR:

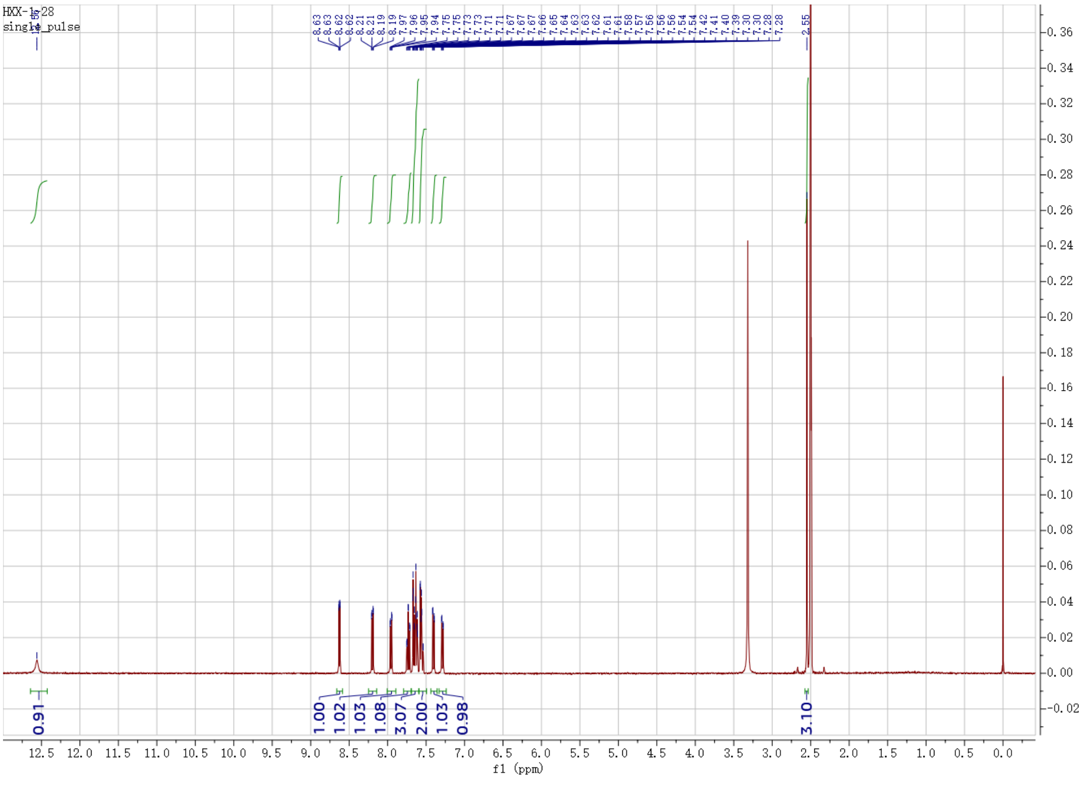

^{13}C-NMR:

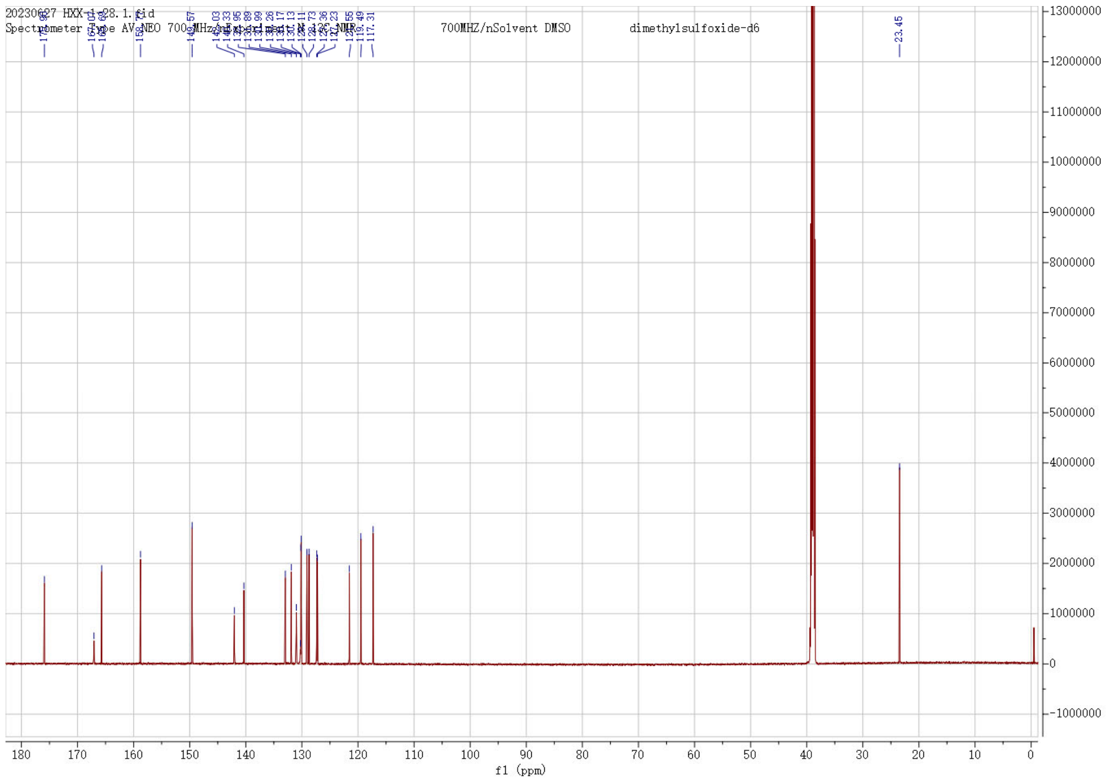

Compound **B19**:

HR-ESI-MS:

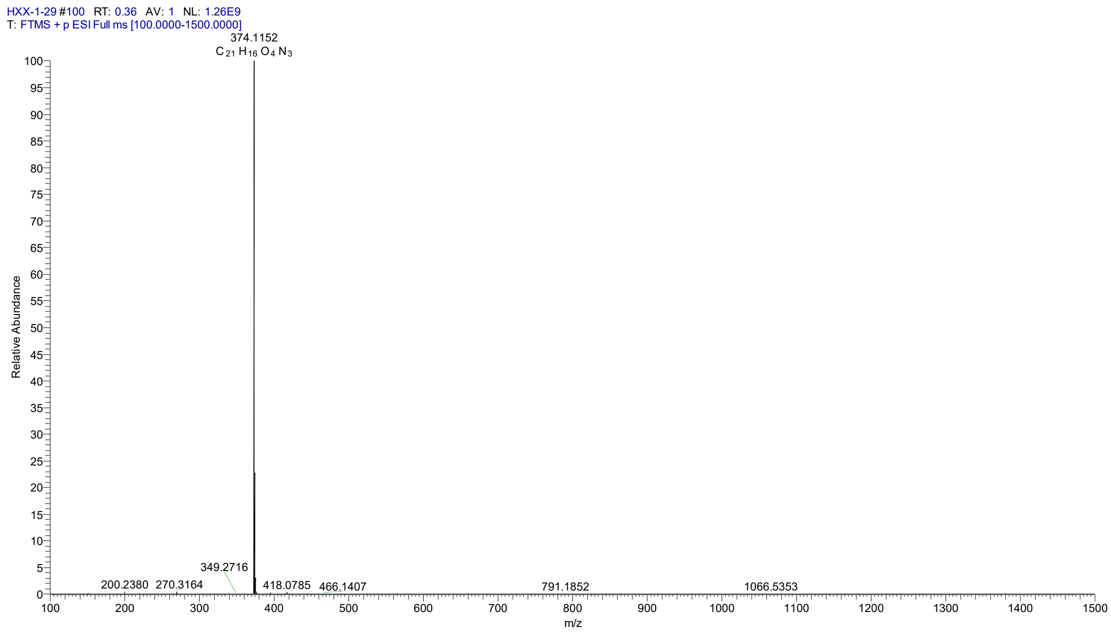

^{1}H-NMR:

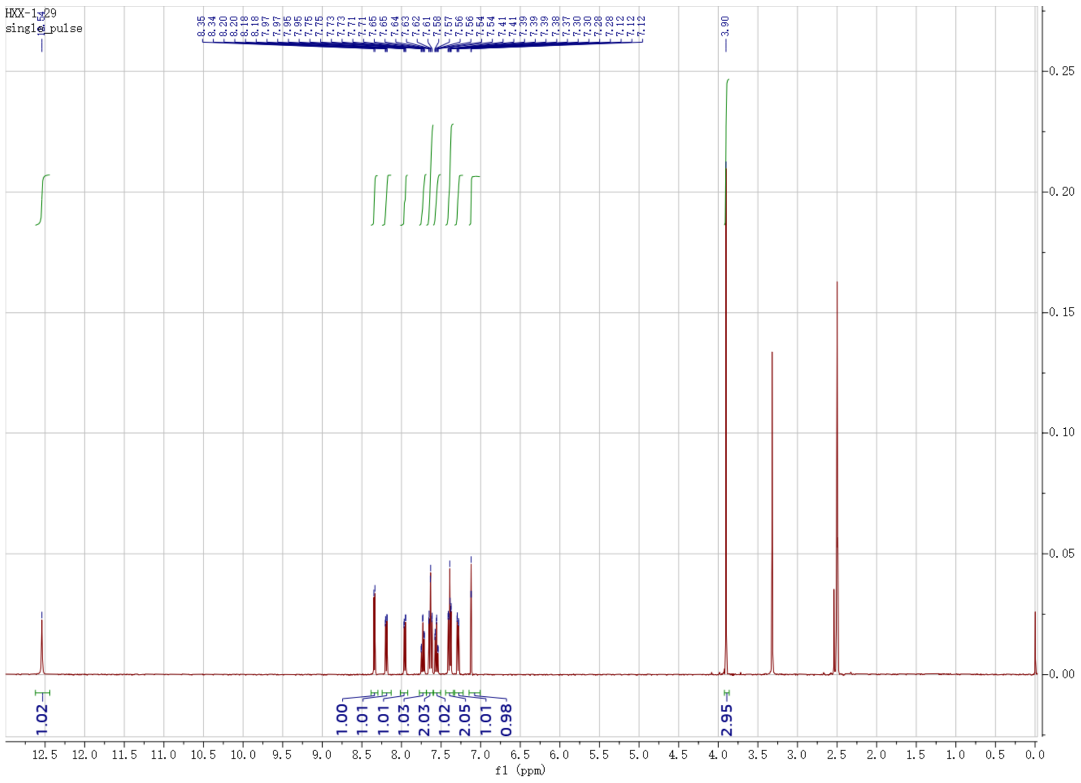

^{13}C-NMR:

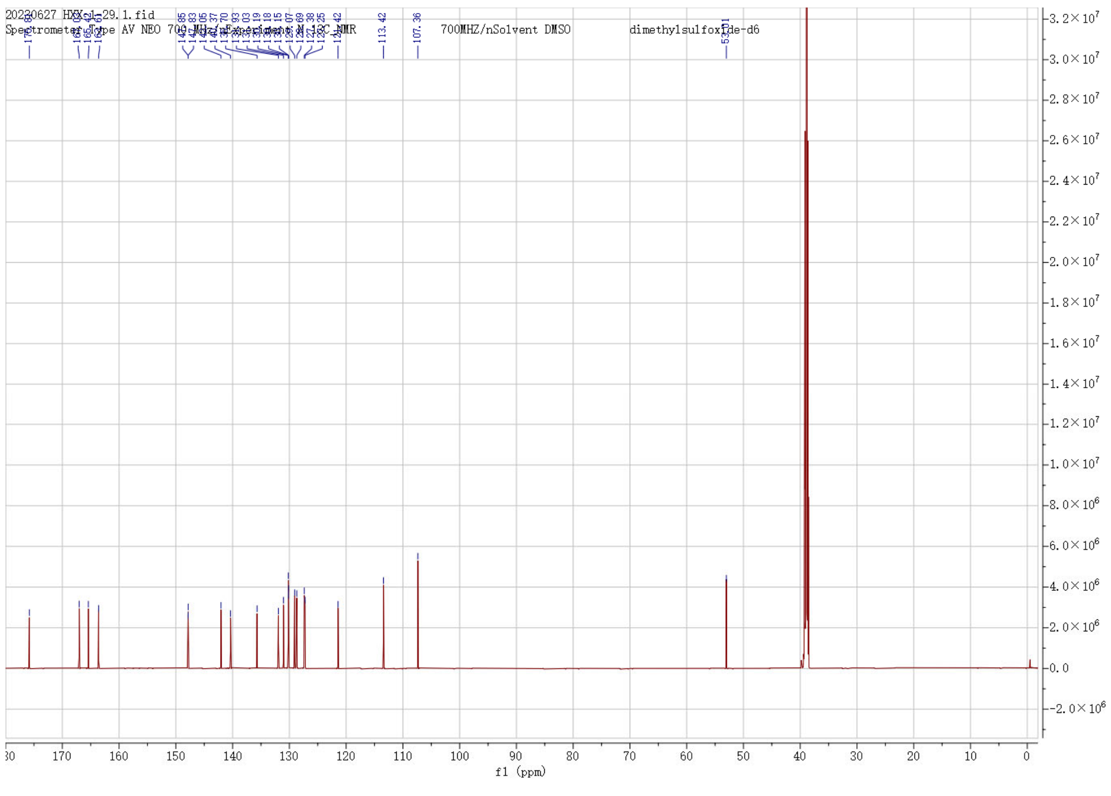

Compound **B20**:

HR-ESI-MS:

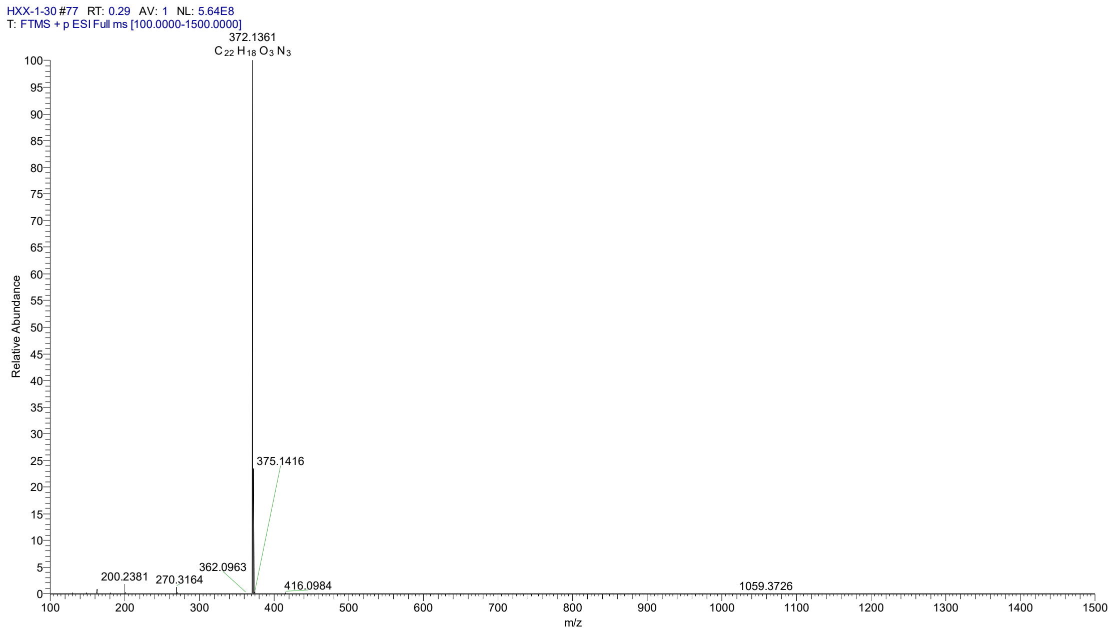

^{1}H-NMR:

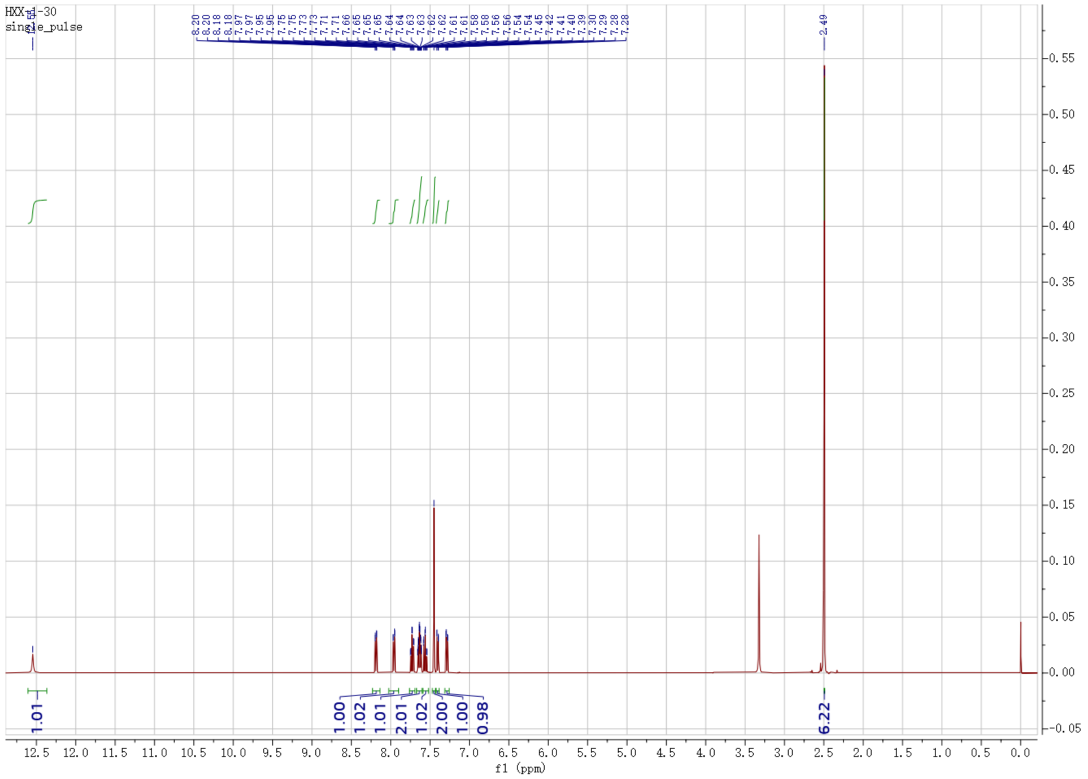

^{13}C-NMR:

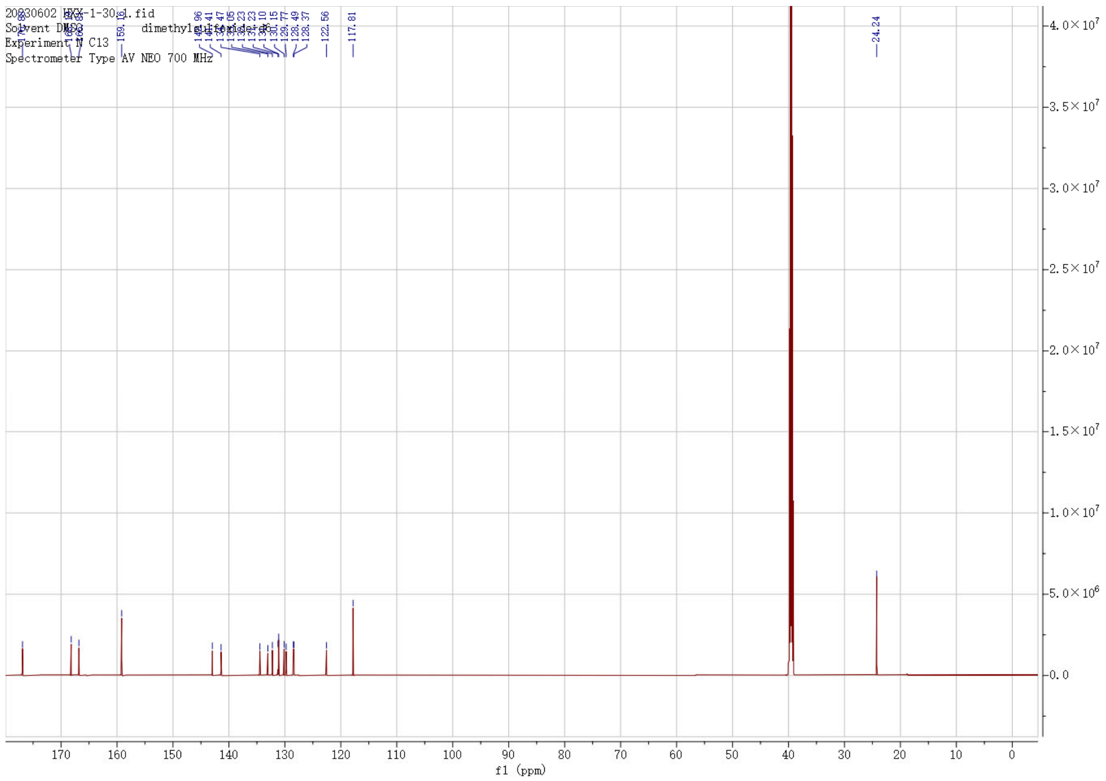

Compound **B21**:

HR-ESI-MS:

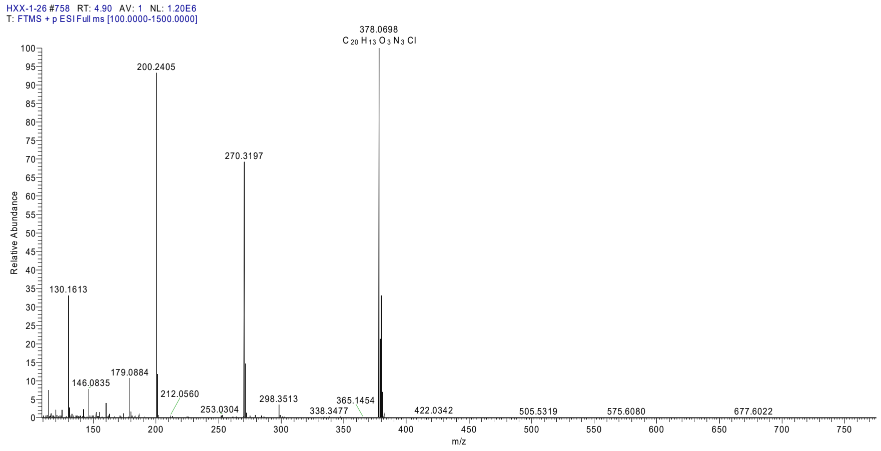

^{1}H-NMR:

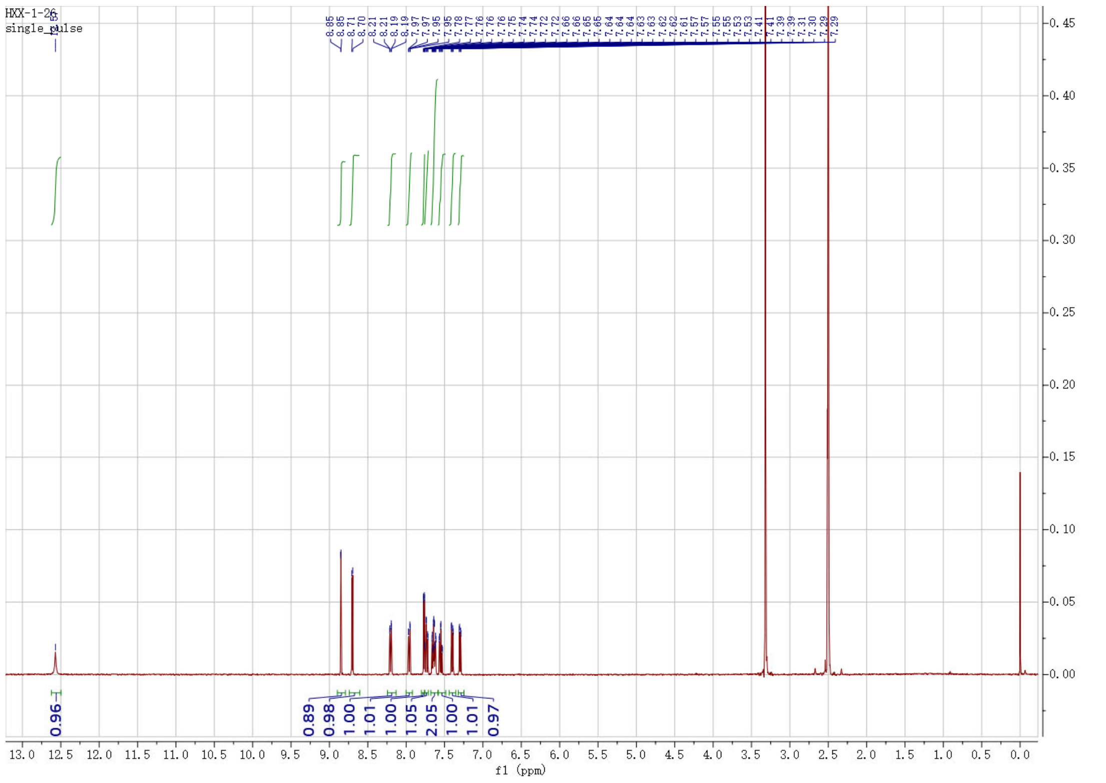

^{13}C-NMR:

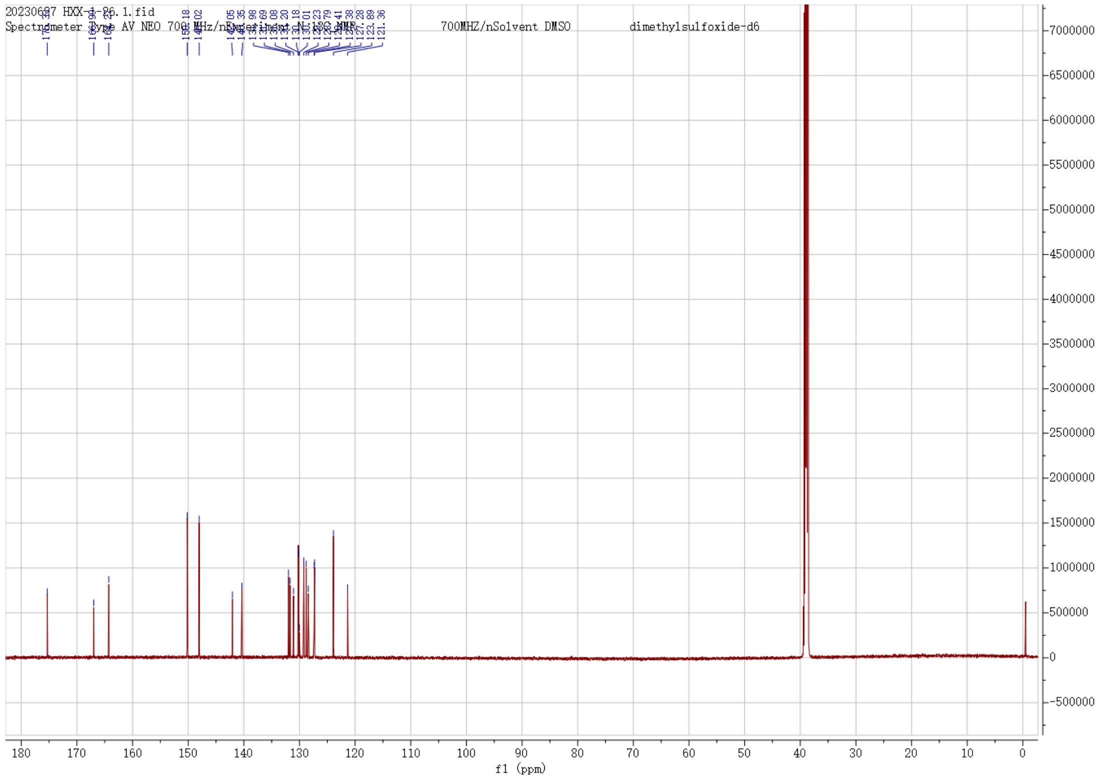

Compound **B22**:

HR-ESI-MS:

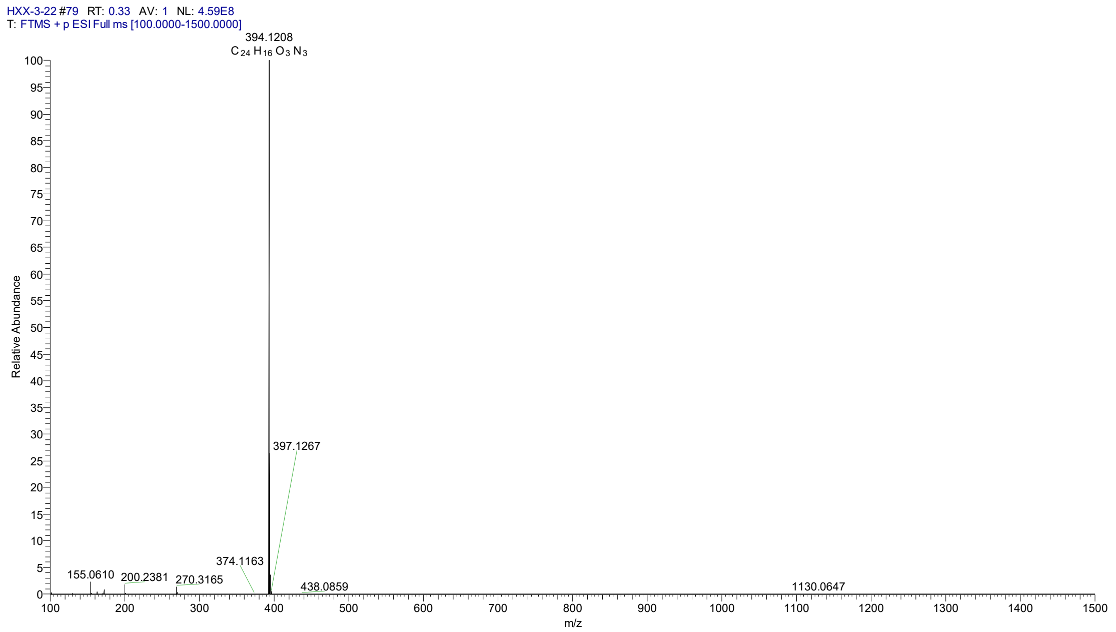

^{1}H-NMR:

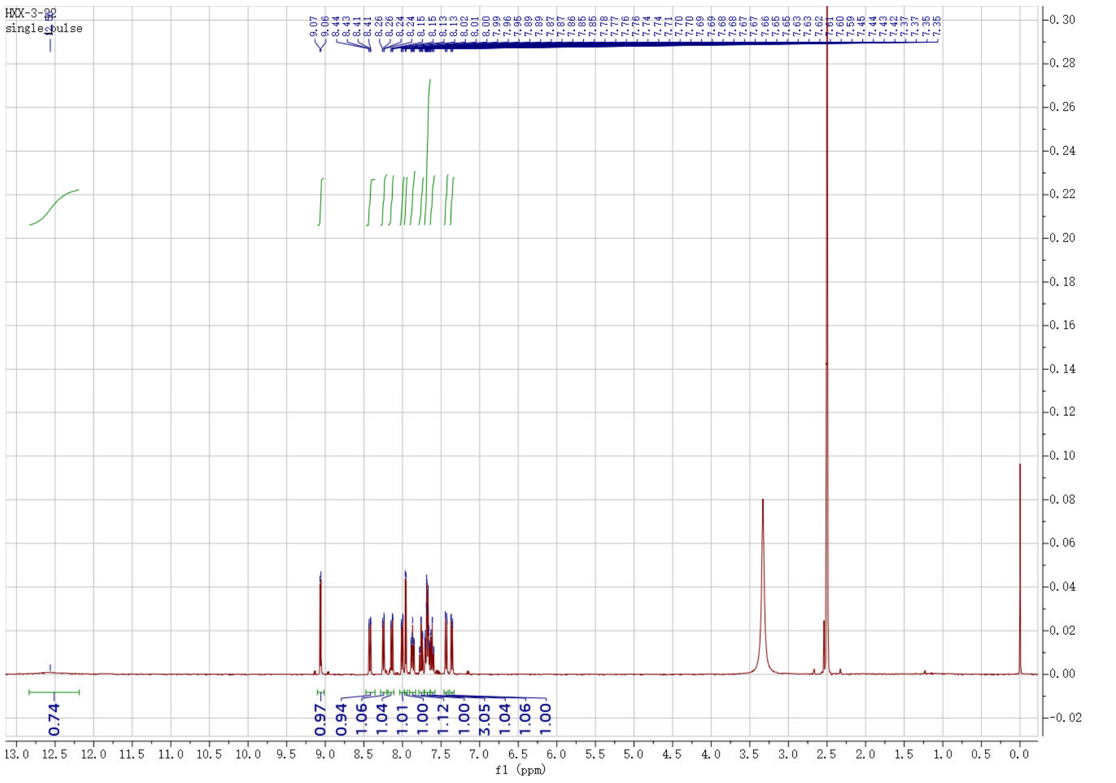

^{13}C-NMR:

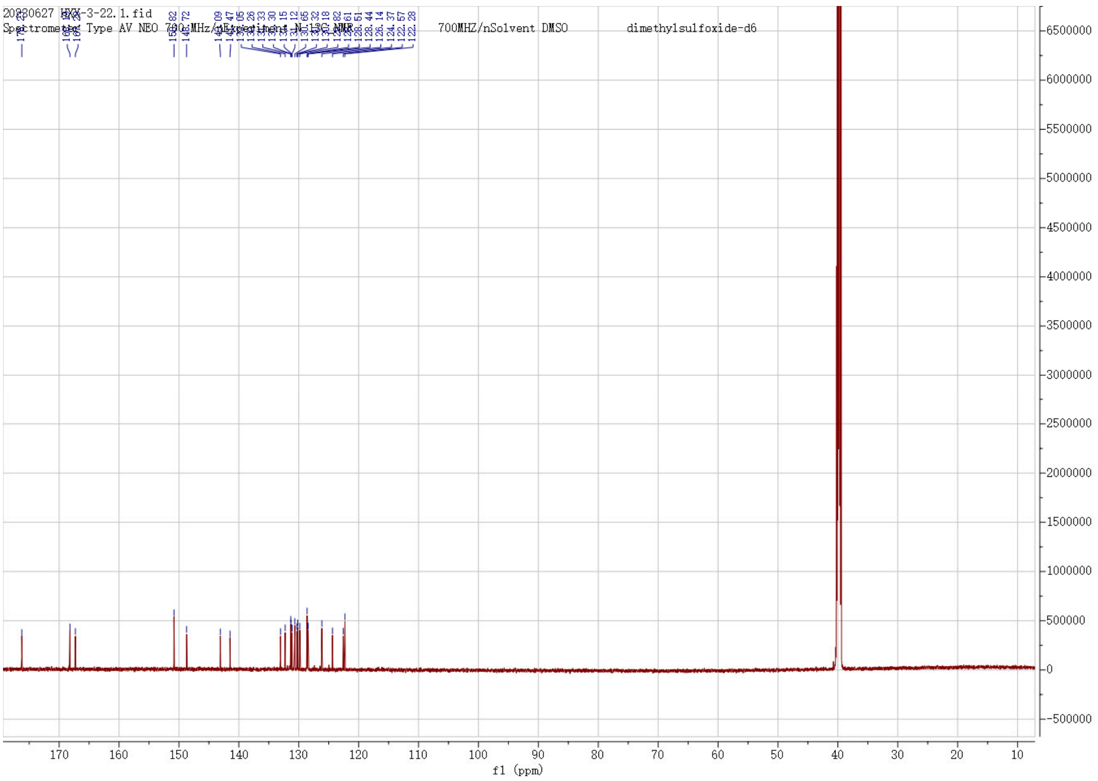

Compound **B23**:

HR-ESI-MS:

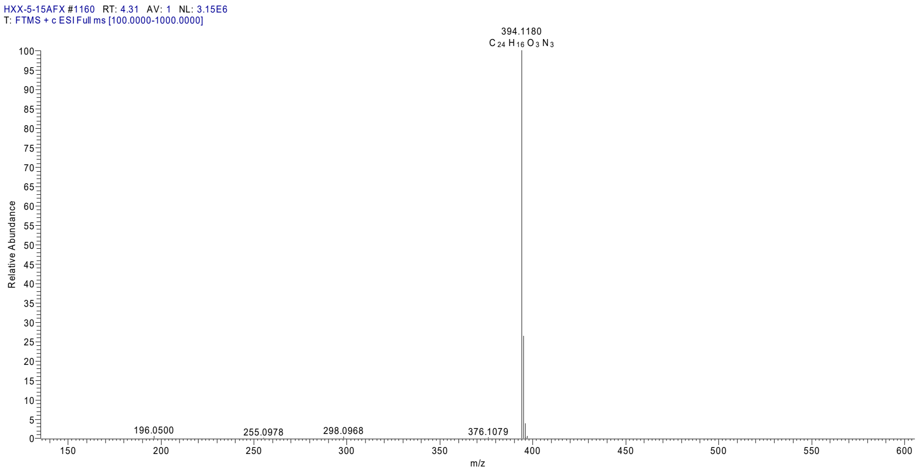

^{1}H-NMR:

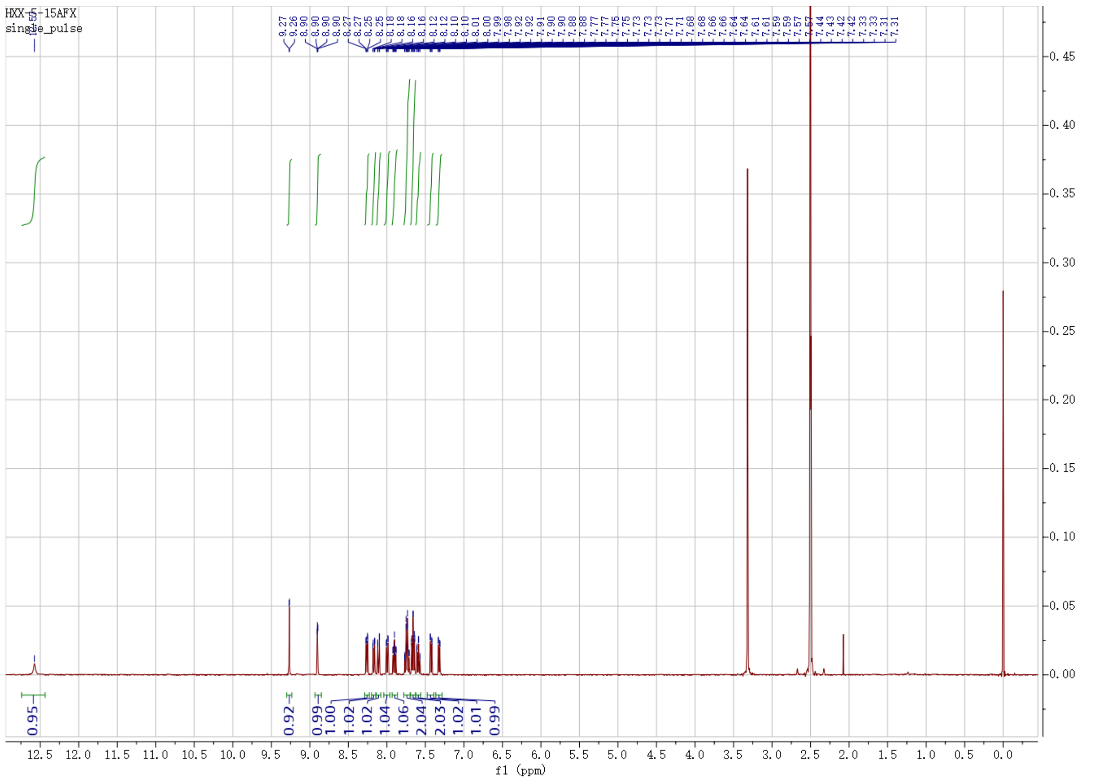

^{13}C-NMR:

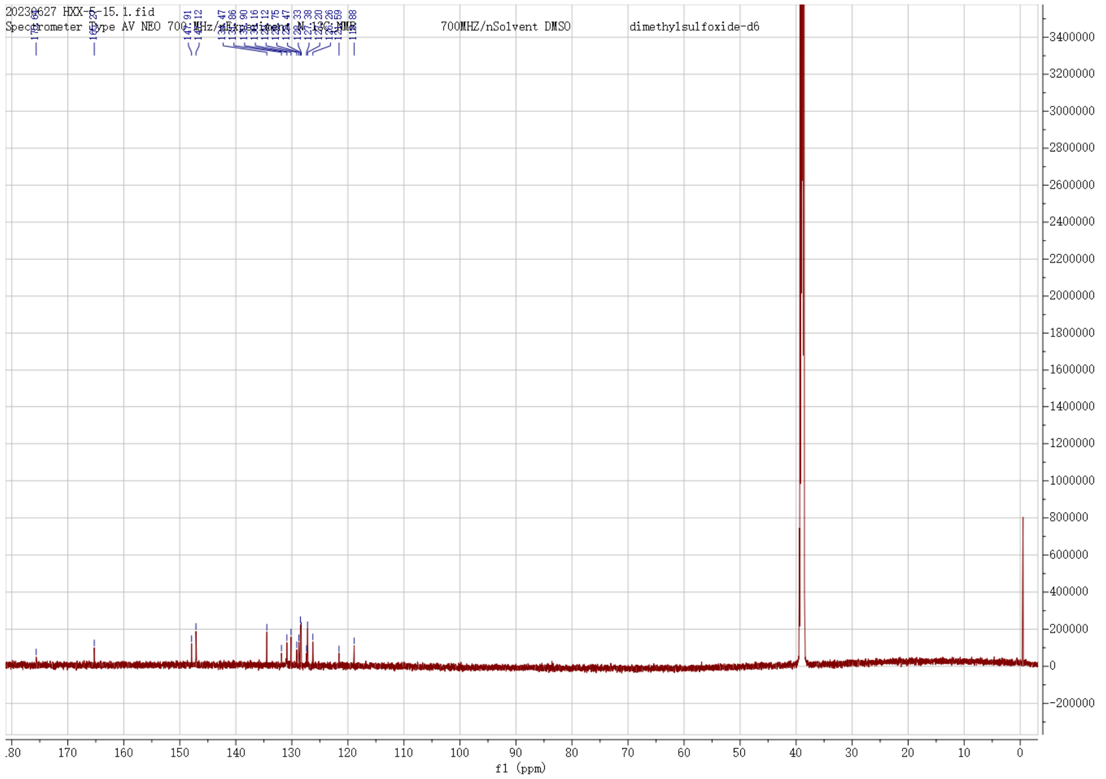

Compound **B24**:

HR-ESI-MS:

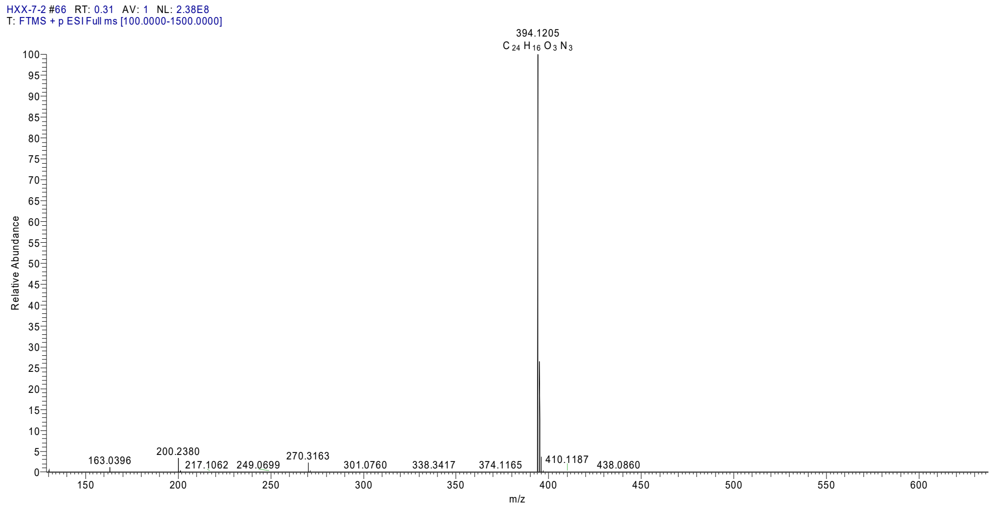

^{1}H-NMR:

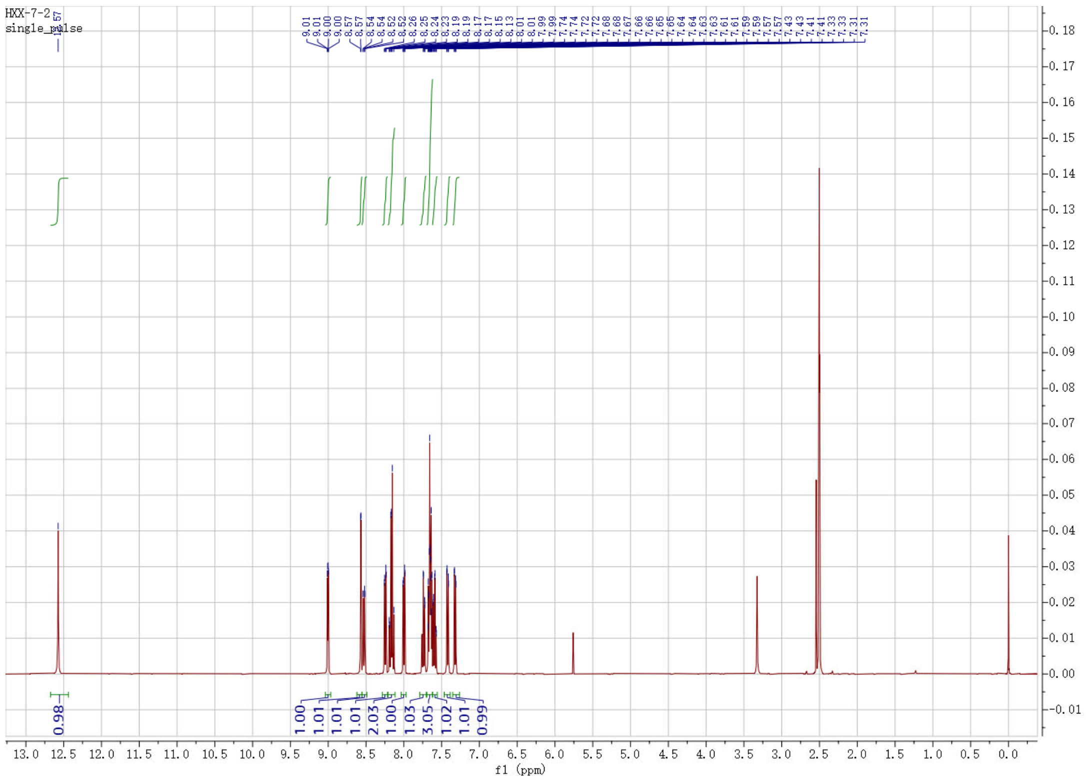

^{13}C-NMR:

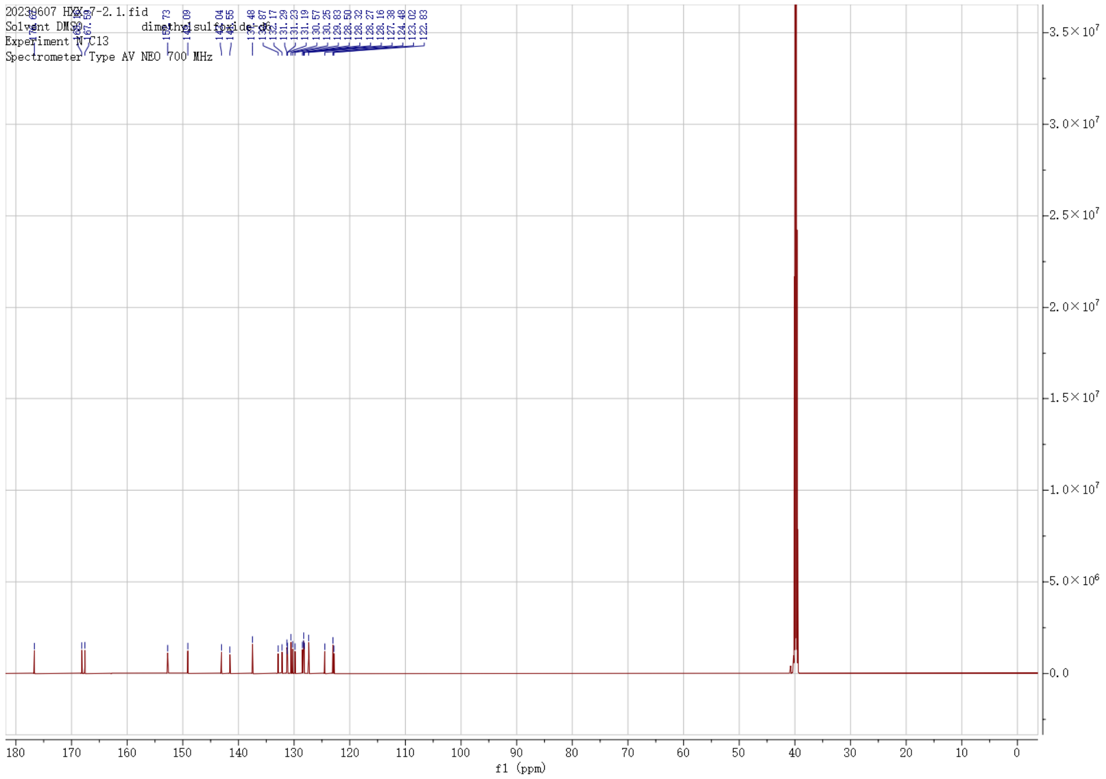

Compound **B25**:

HR-ESI-MS:

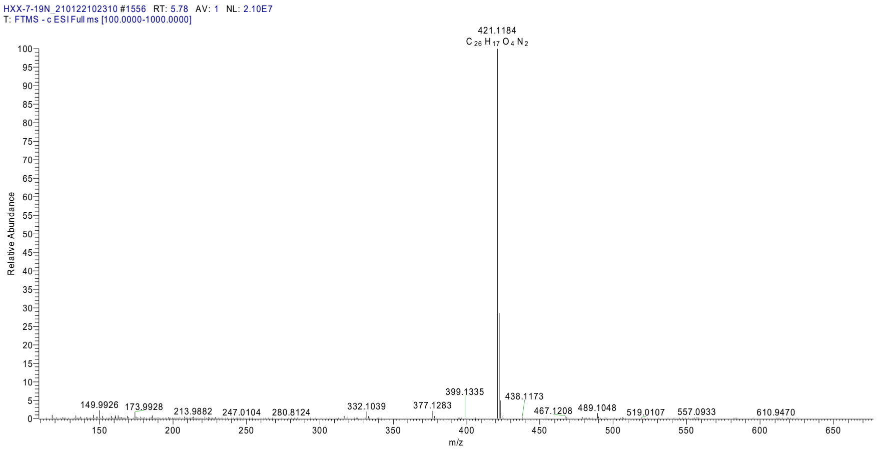

^{1}H-NMR:

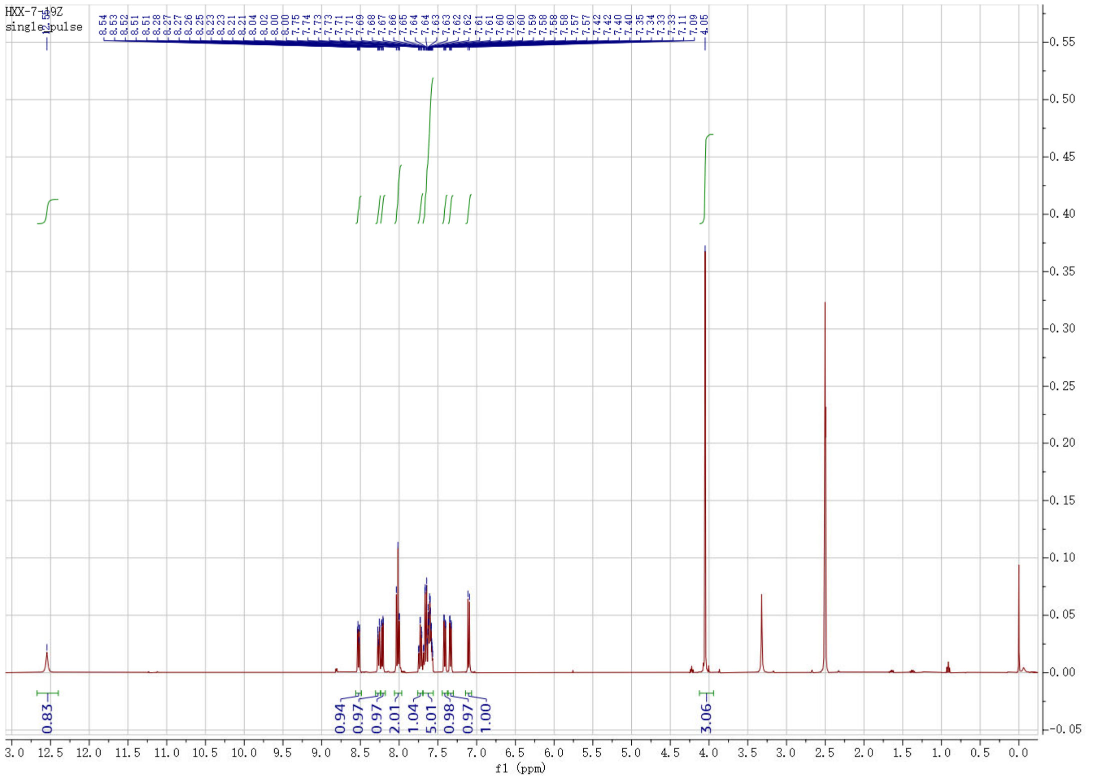

^{13}C-NMR:

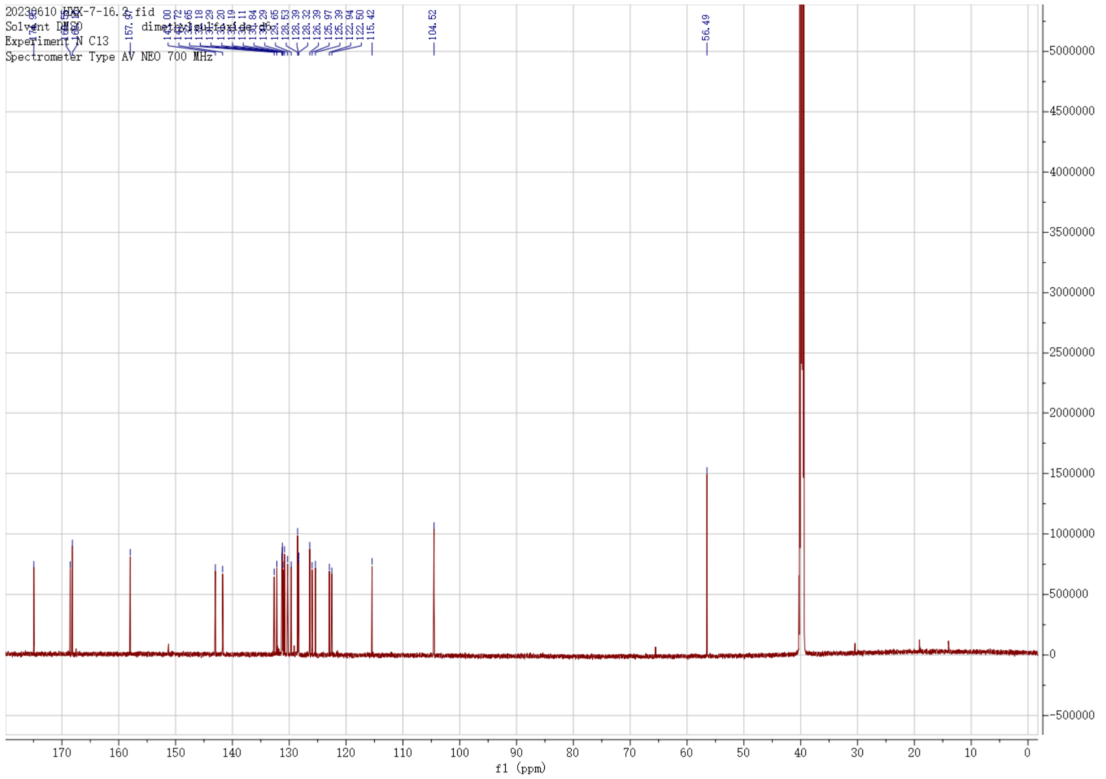

Compound **B26**:

HR-ESI-MS:

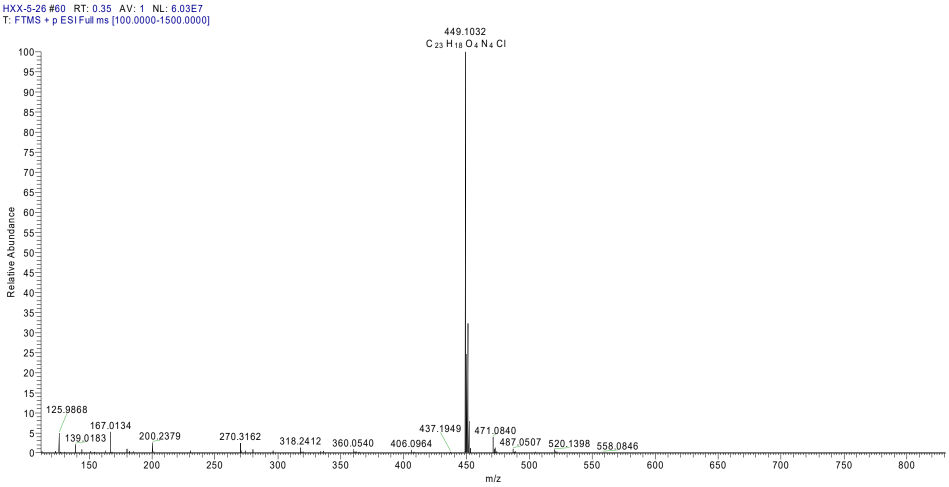

¹H-NMR:

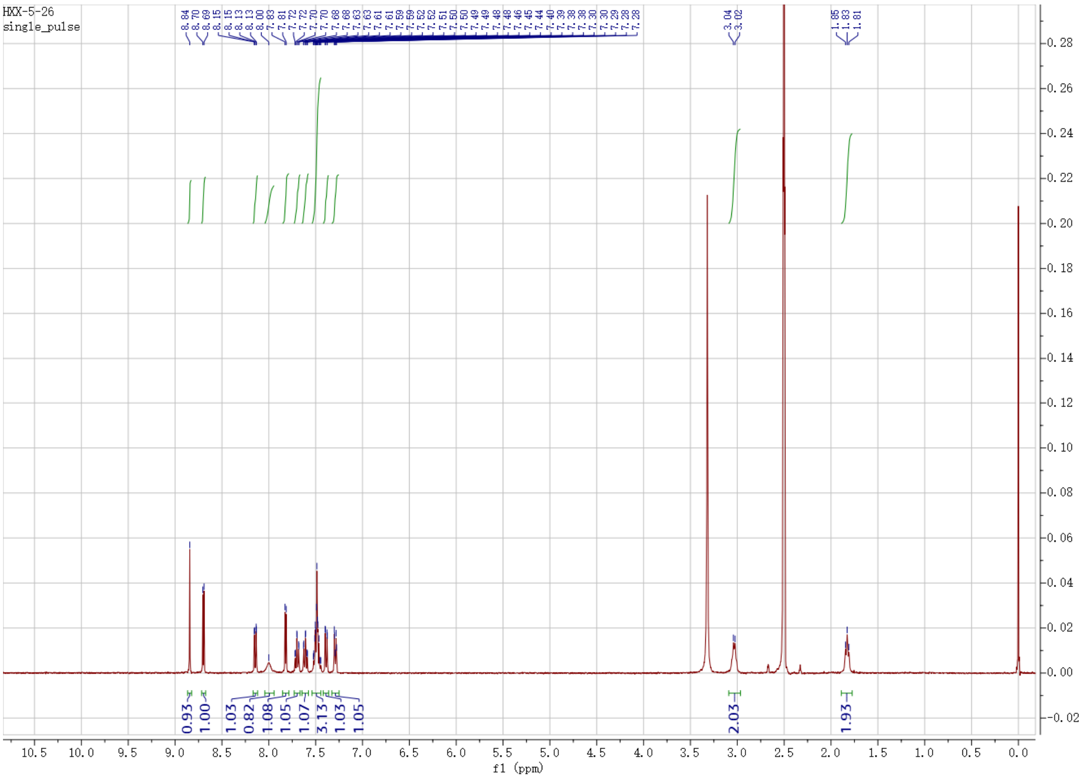

^{13}C-NMR:

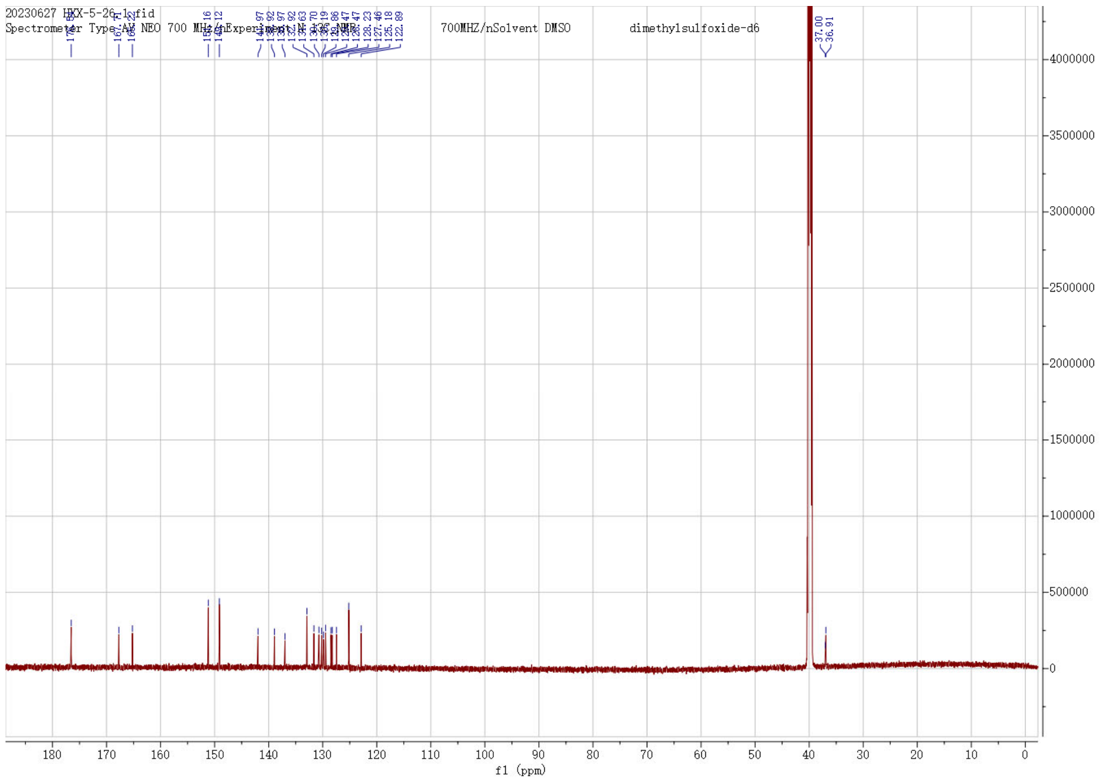

Compound **B27**:

HR-ESI-MS:

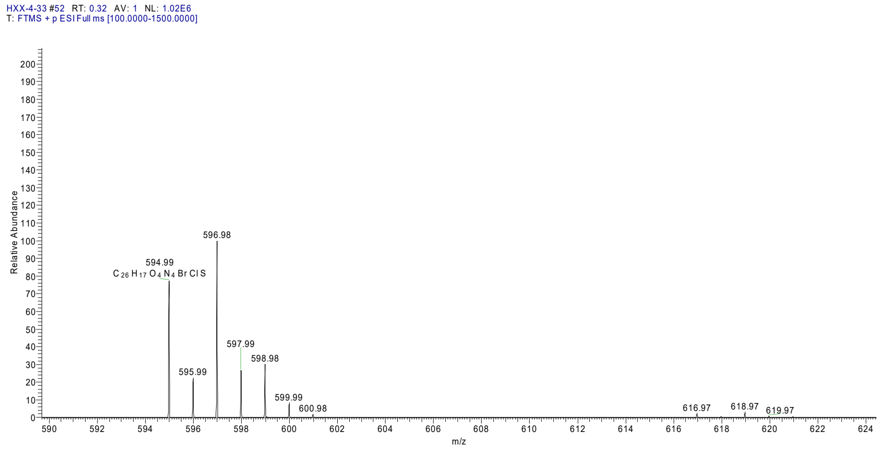

^{1}H-NMR:

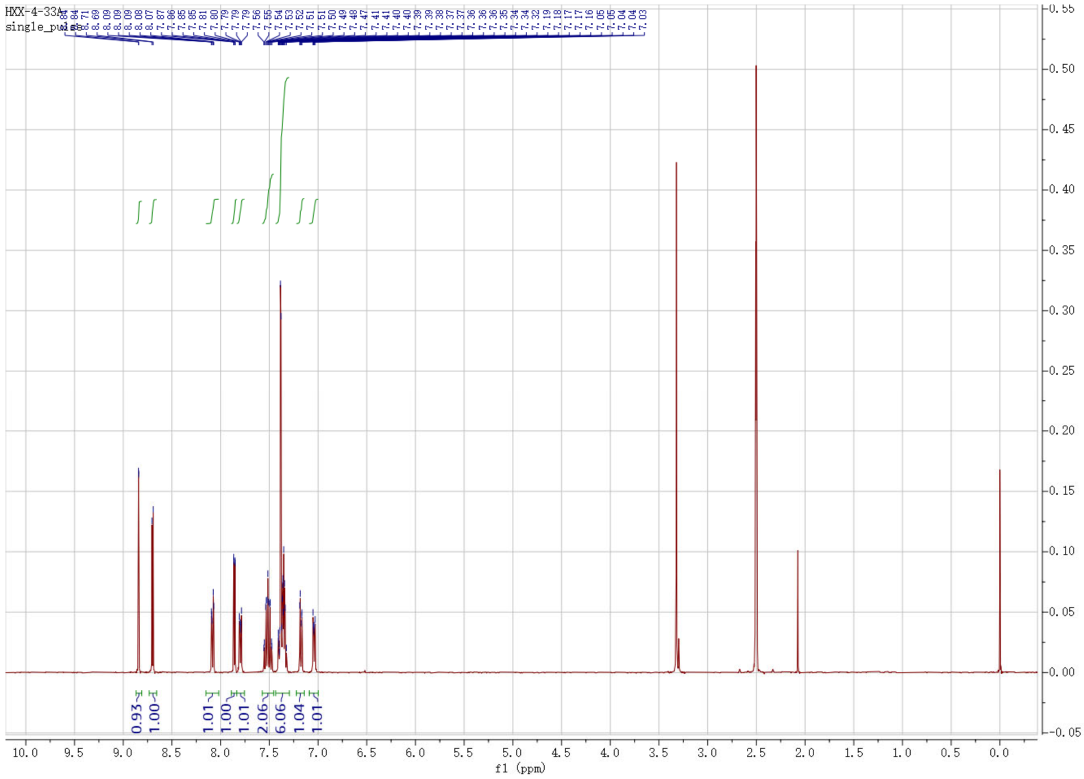

^{13}C-NMR:

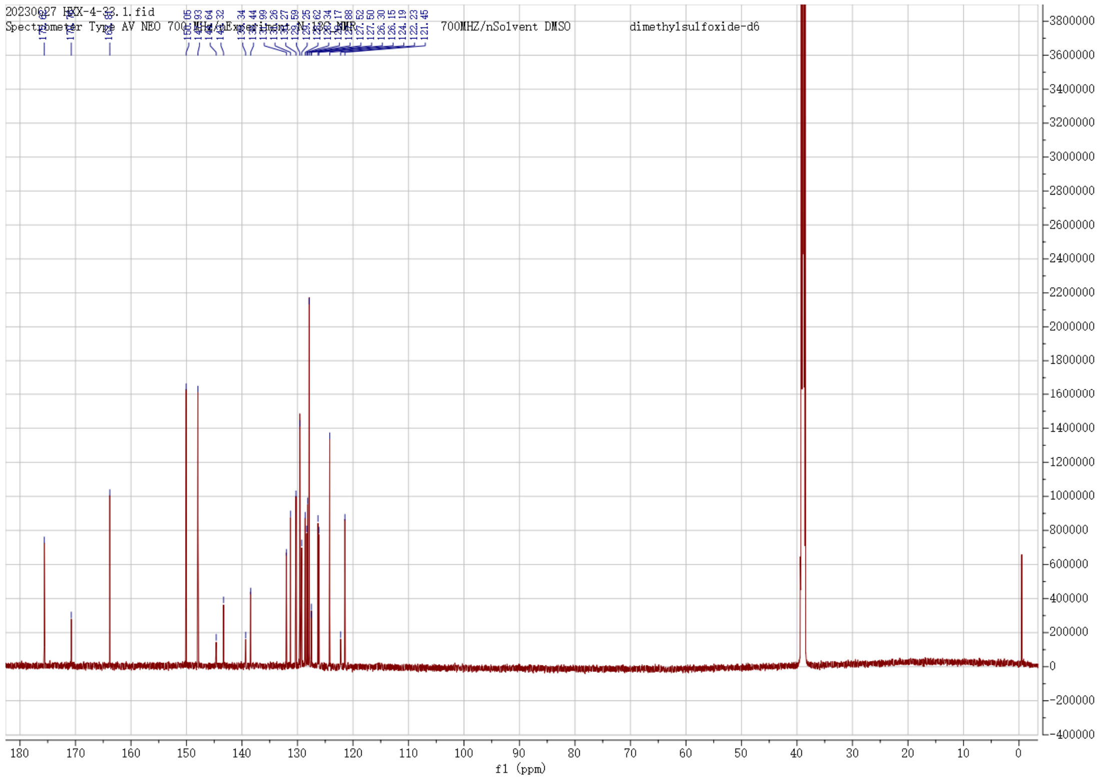

Compound **B28**:

HR-ESI-MS:

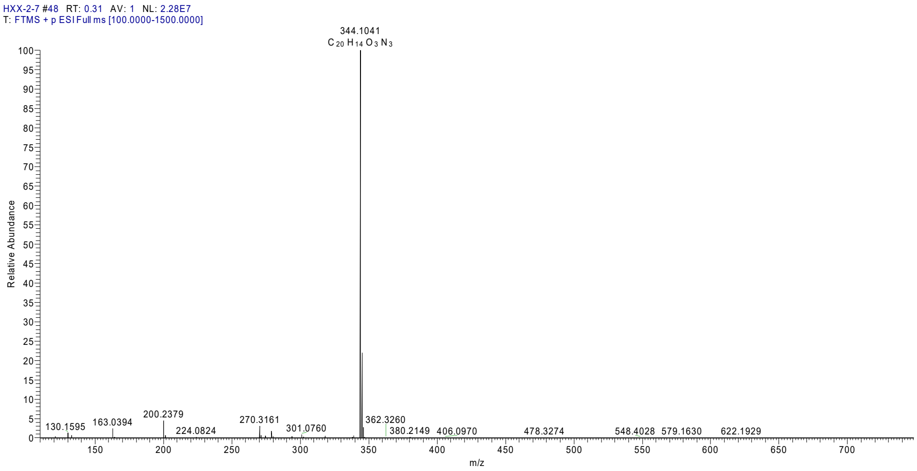

^{1}H-NMR:

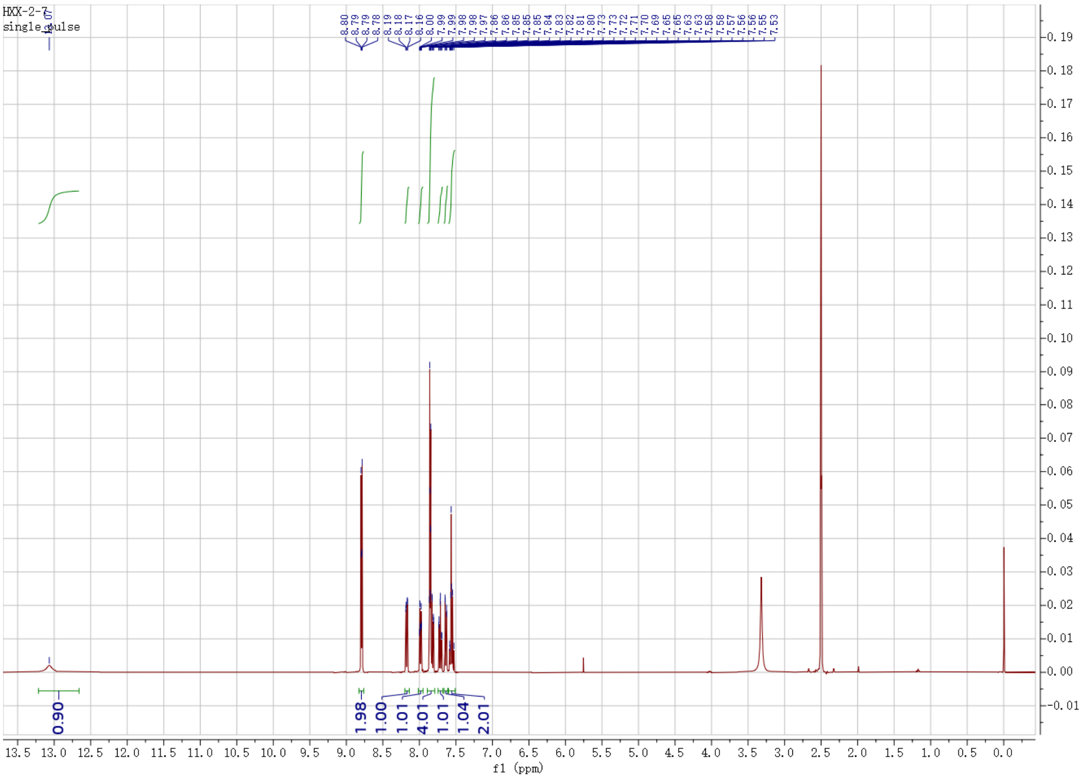

^{13}C-NMR:

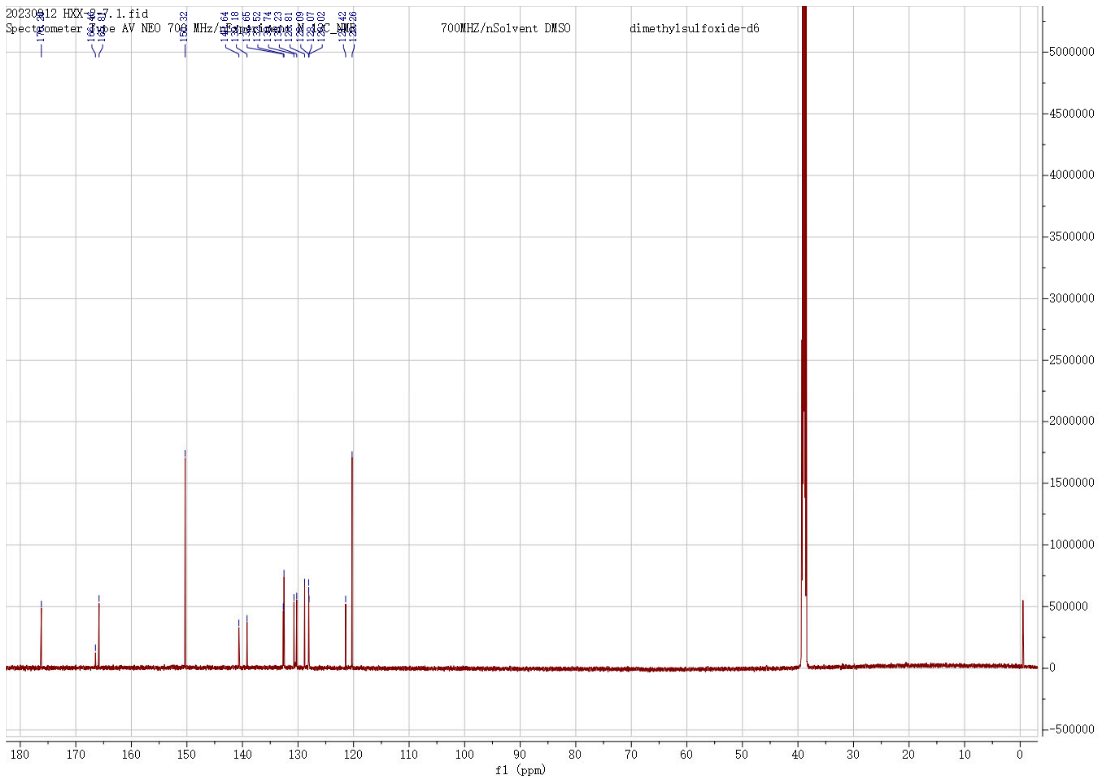

Compound **B29**:

HR-ESI-MS:

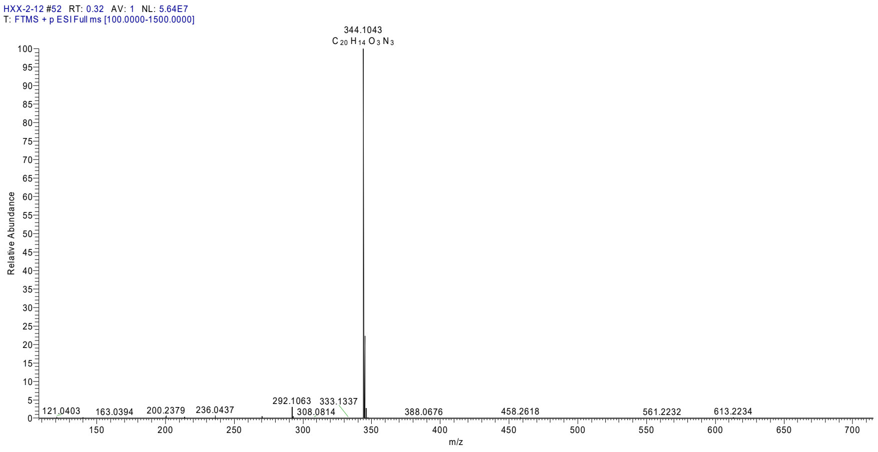

^{1}H-NMR:

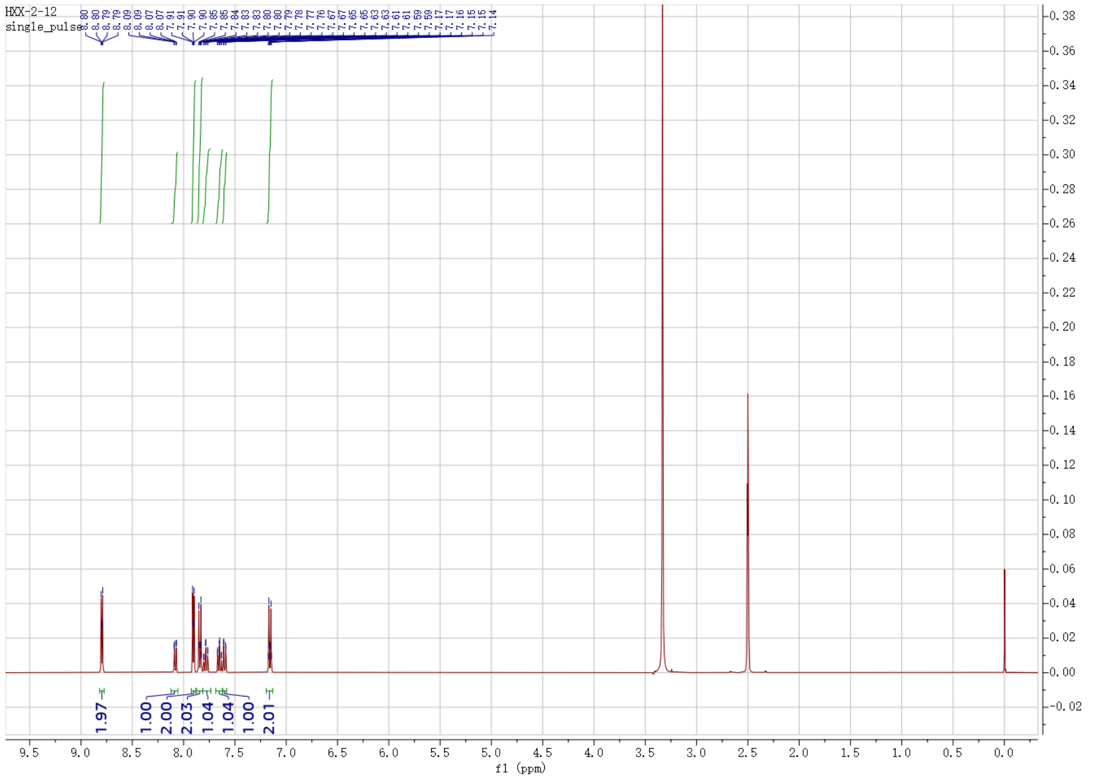

^{13}C-NMR:

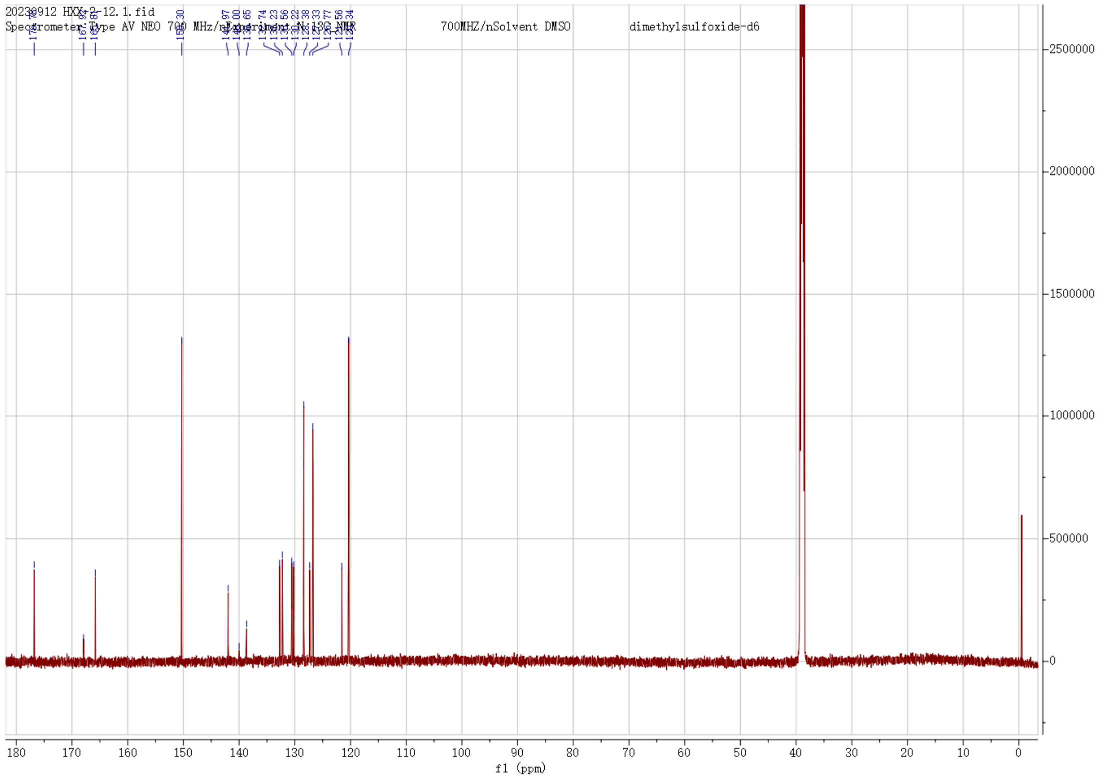

Compound **B30**:

HR-ESI-MS:

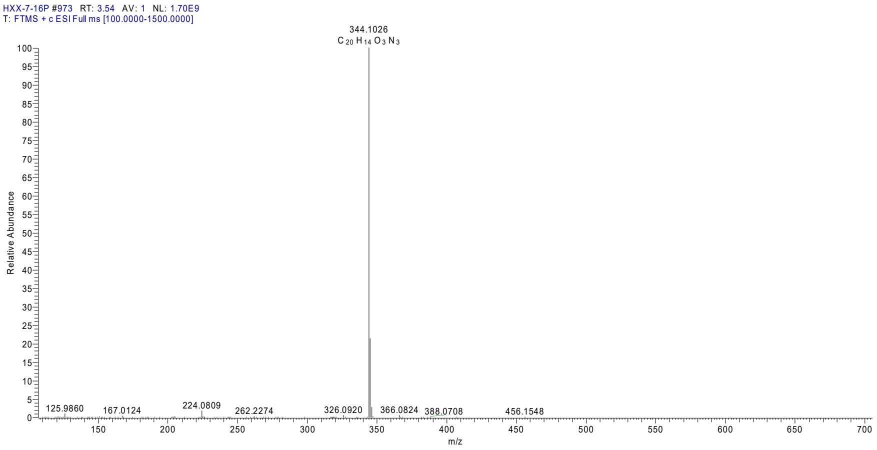

^{1}H-NMR:

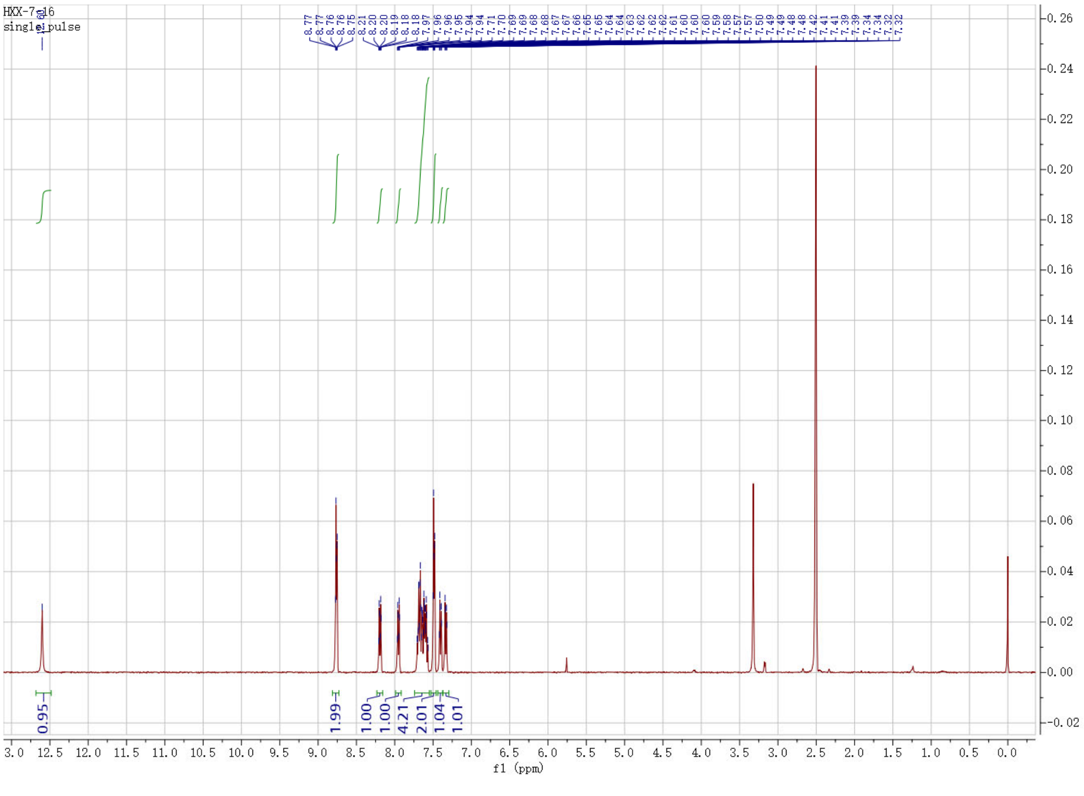

^{13}C-NMR:

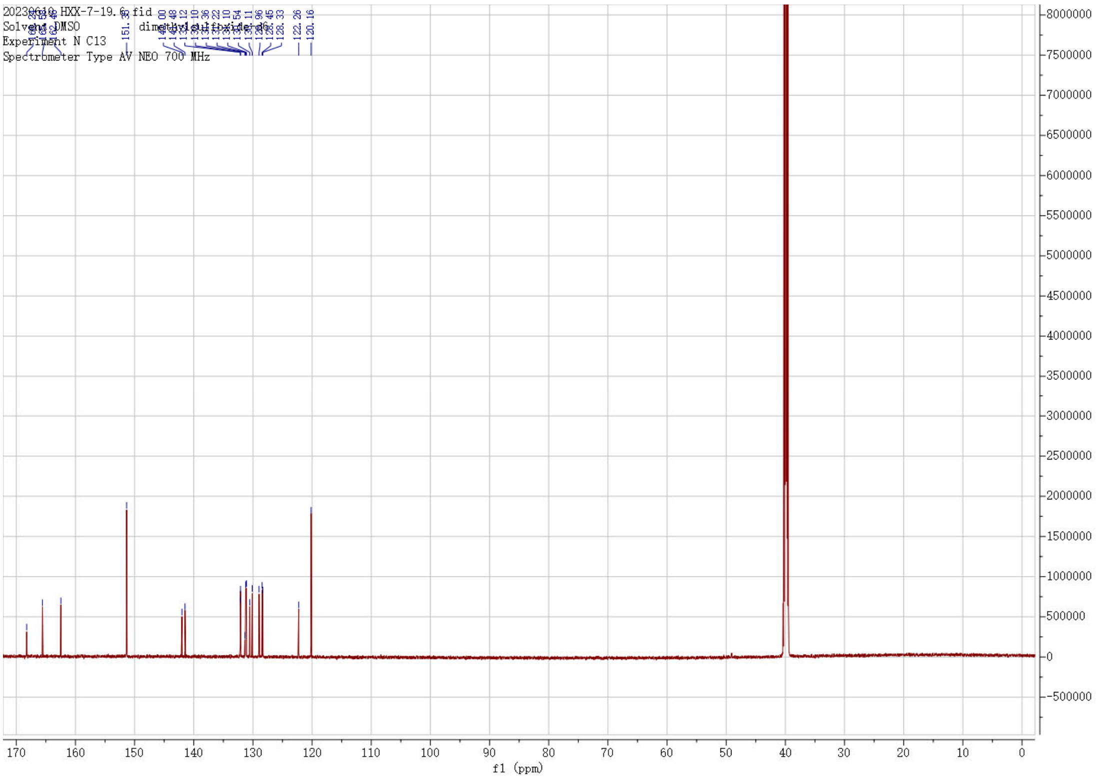

IC50 curves for all the target compounds are provided herein.

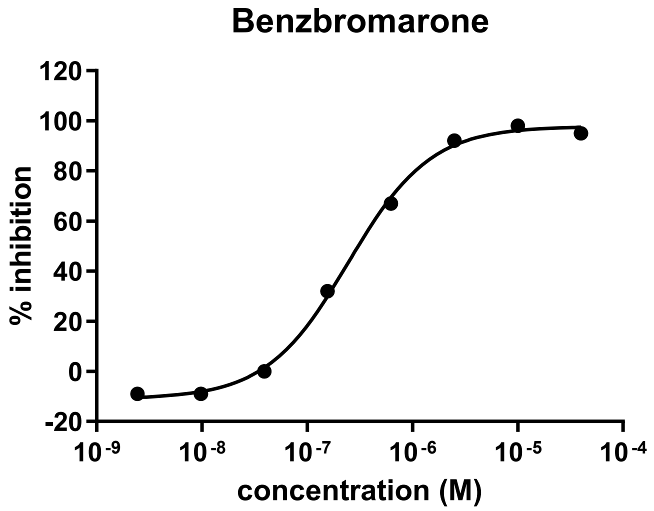

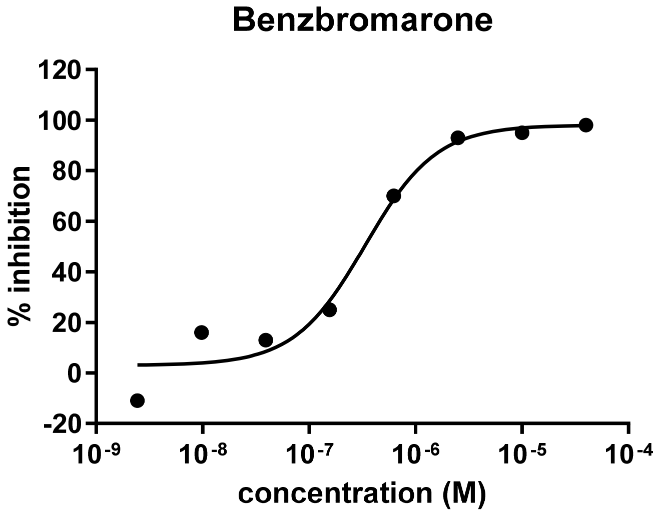

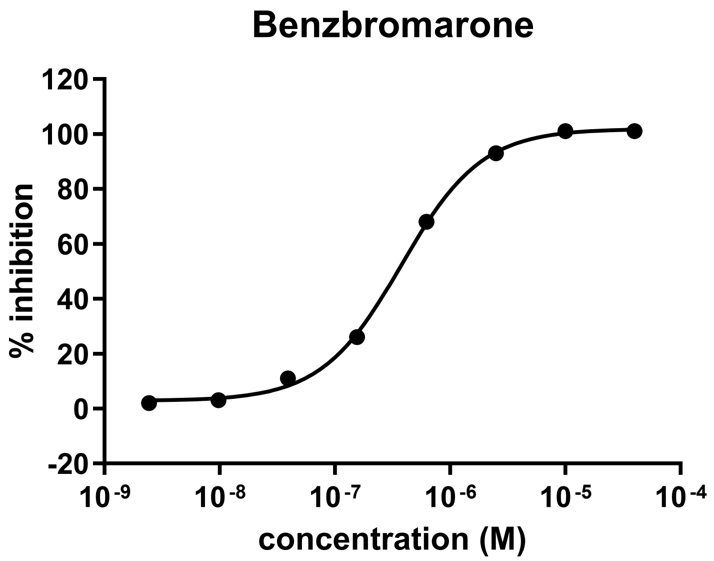
Benzbromarone
120
100
80
60
40
20
0
-20
% inhibition
10 -9
10 -8
10 -7
10 -6
10 -5
10 -4
concentration (M)

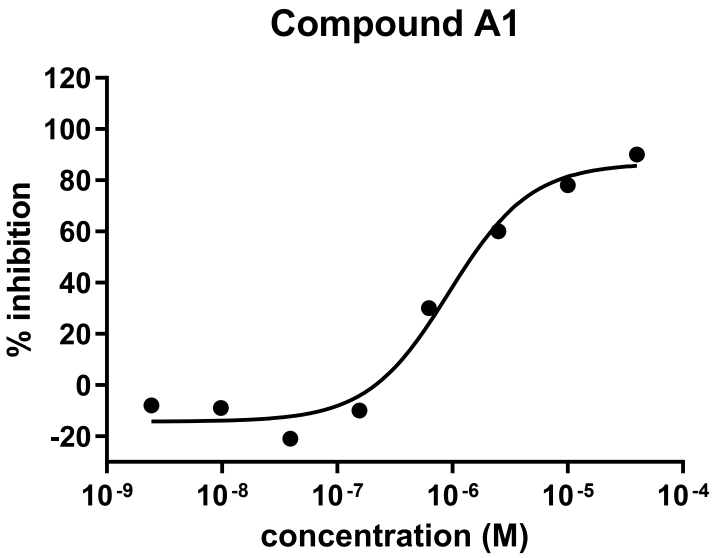
Compound A1
120
100
80
60
40
20
0
-20
% inhibition
10 -9
10 -8
10 -7
10 -6
10 -5
10 -4
concentration (M)

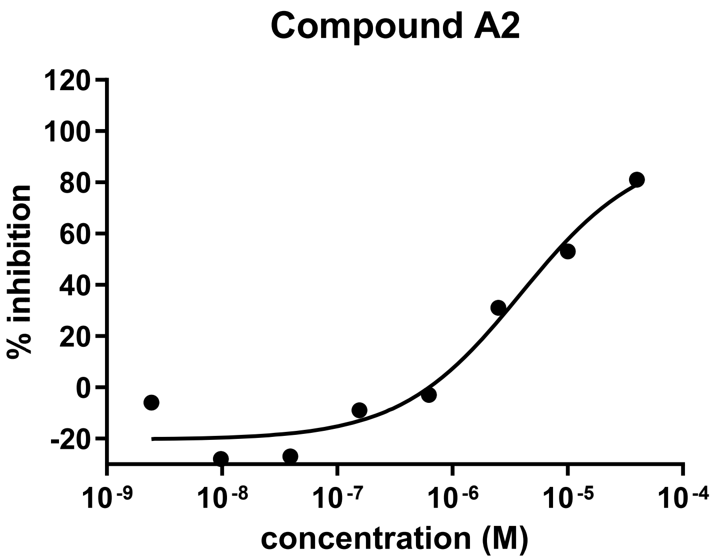
Compound A2
% inhibition
120
100
80
60
40
20
0
-20
10-9
10-8
10-7
10-6
10-5
10-4
concentration (M)

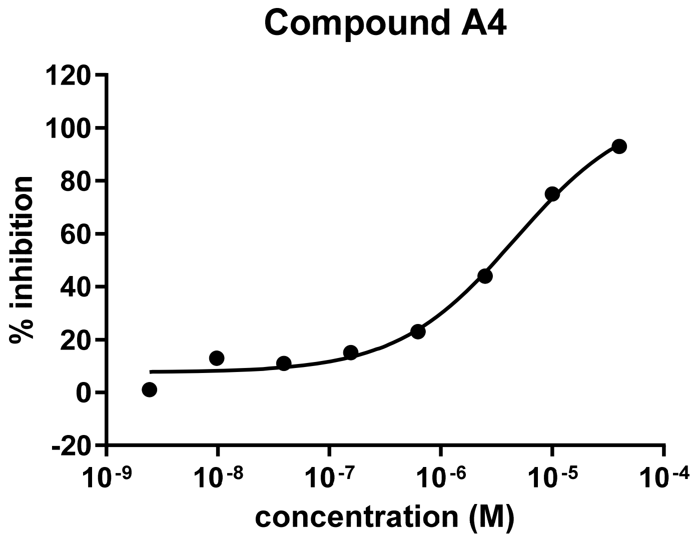
Compound A4
% inhibition
120
100
80
60
40
20
0
-20
10-9
10-8
10-7
10-6
10-5
10-4
concentration (M)

Compound A6

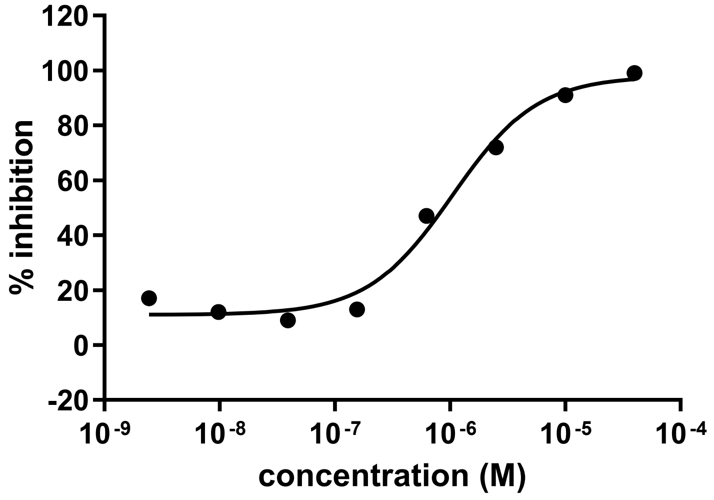

Compound A7

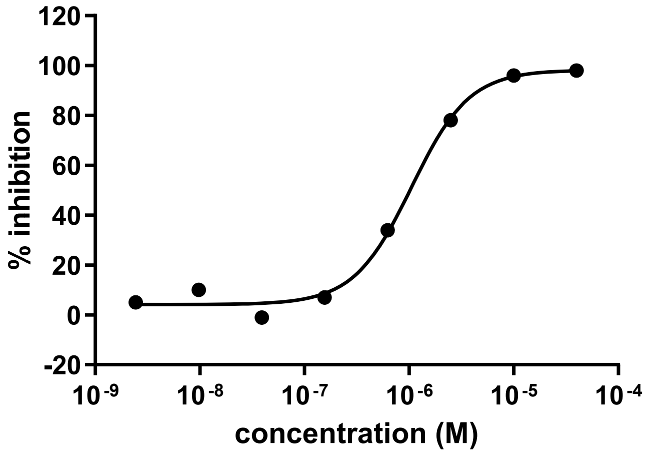

Compound A8

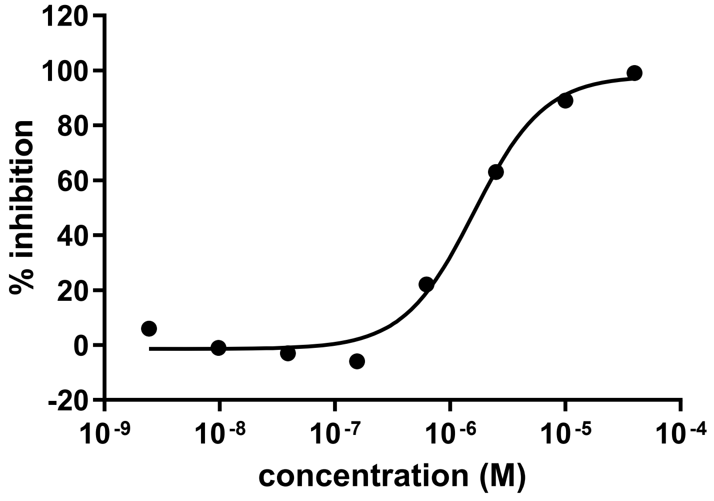

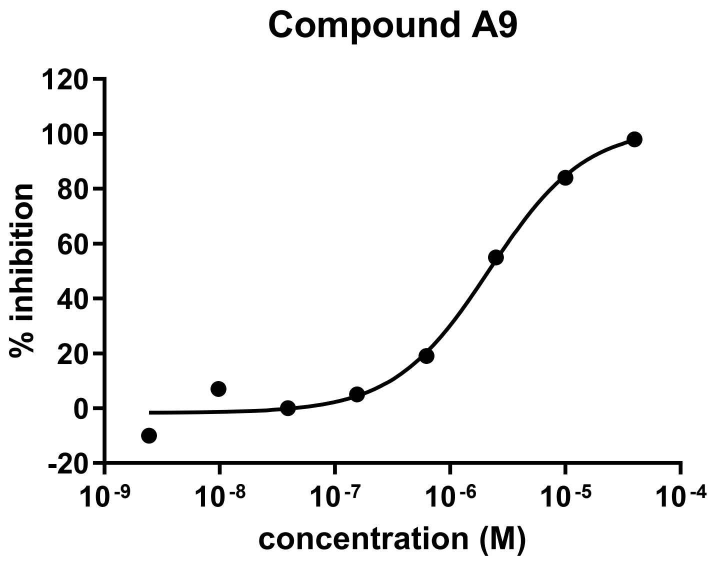
Compound A9
120
100
80
60
40
20
0
-20
% inhibition
10^{-9}
10^{-8}
10^{-7}
10^{-6}
10^{-5}
10^{-4}
concentration (M)

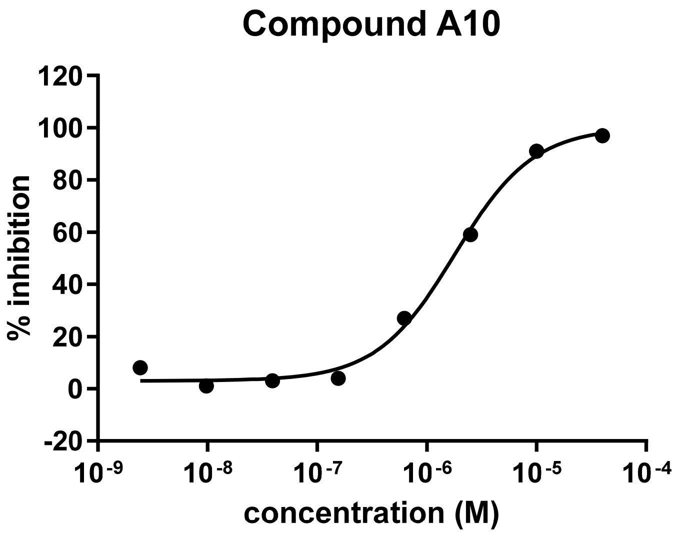
Compound A10
120
100
80
60
40
20
0
-20
% inhibition
10^{-9}
10^{-8}
10^{-7}
10^{-6}
10^{-5}
10^{-4}
concentration (M)

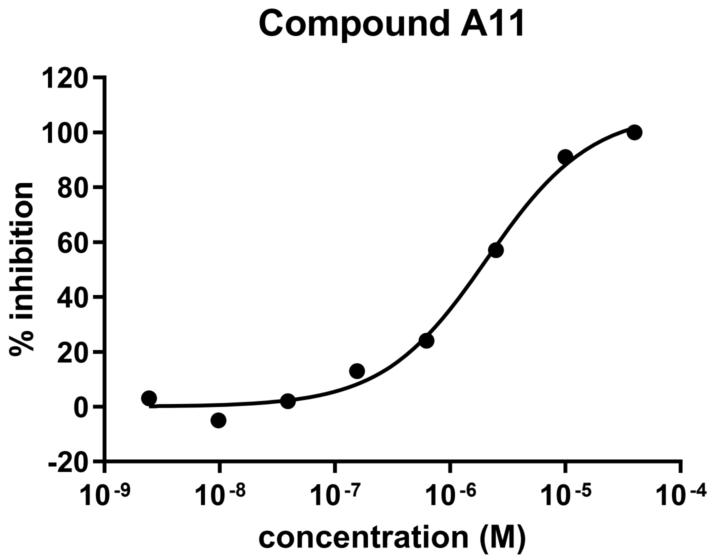
Compound A11
120
100
80
60
40
20
0
-20
% inhibition
10^{-9}
10^{-8}
10^{-7}
10^{-6}
10^{-5}
10^{-4}
concentration (M)

Compound A13

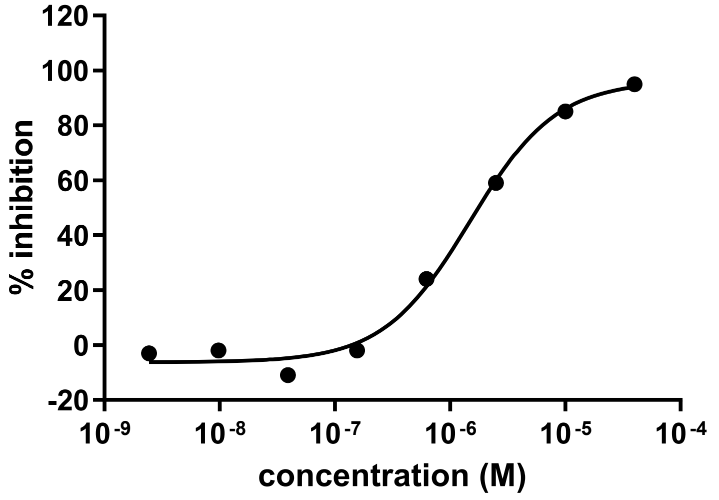

Compound B14

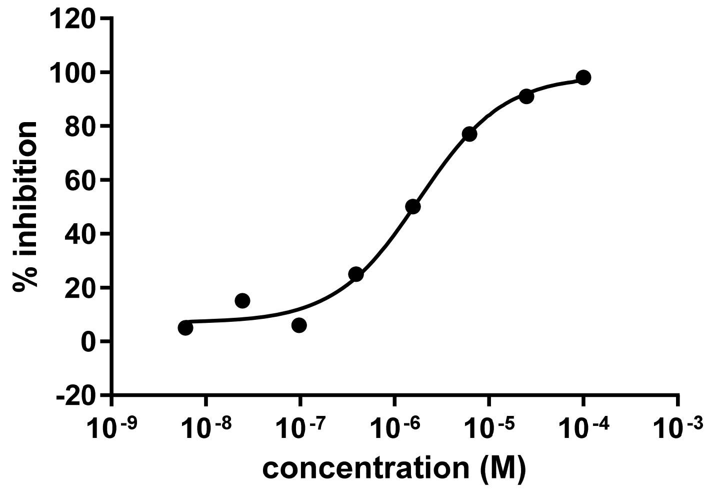

Compound B15

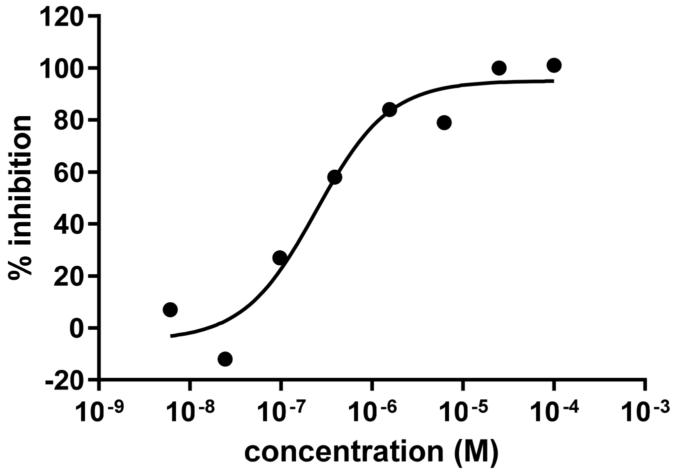

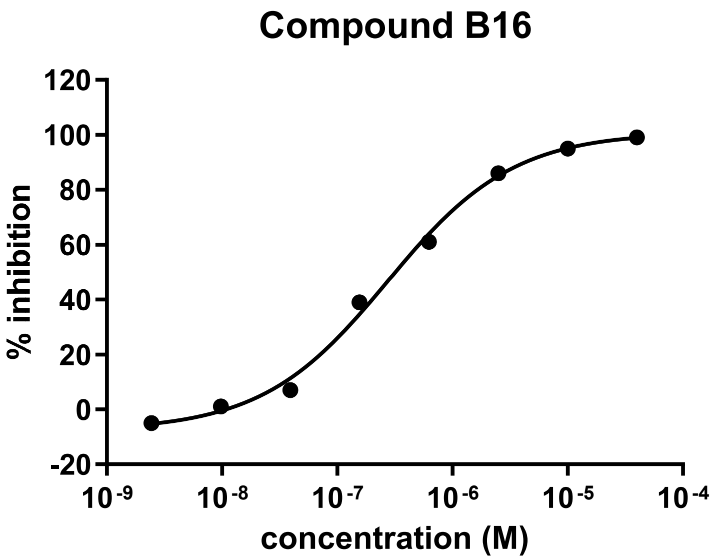

Compound B16
% inhibition
120
100
80
60
40
20
0
-20
10^{-9}
10^{-8}
10^{-7}
10^{-6}
10^{-5}
10^{-4}
concentration (M)

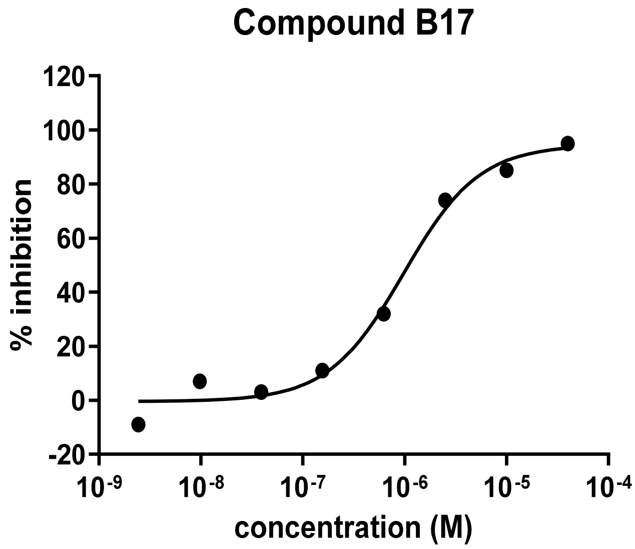

Compound B17
% inhibition
120
100
80
60
40
20
0
-20
10^{-9}
10^{-8}
10^{-7}
10^{-6}
10^{-5}
10^{-4}
concentration (M)

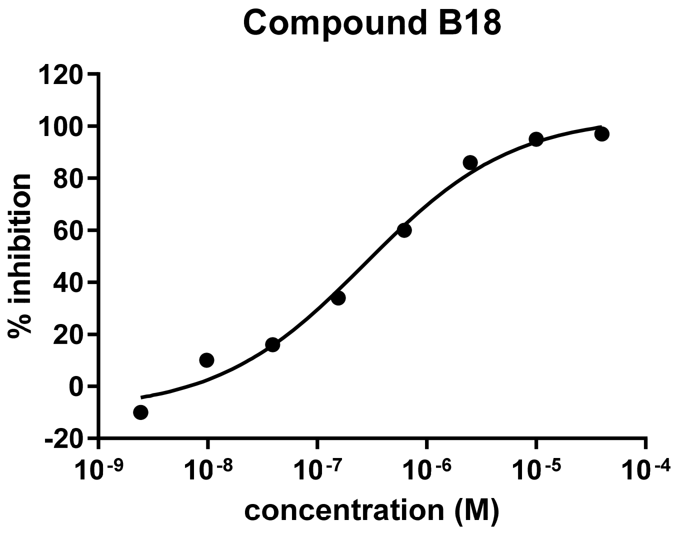

Compound B18
% inhibition
120
100
80
60
40
20
0
-20
10^{-9}
10^{-8}
10^{-7}
10^{-6}
10^{-5}
10^{-4}
concentration (M)

Compound B19

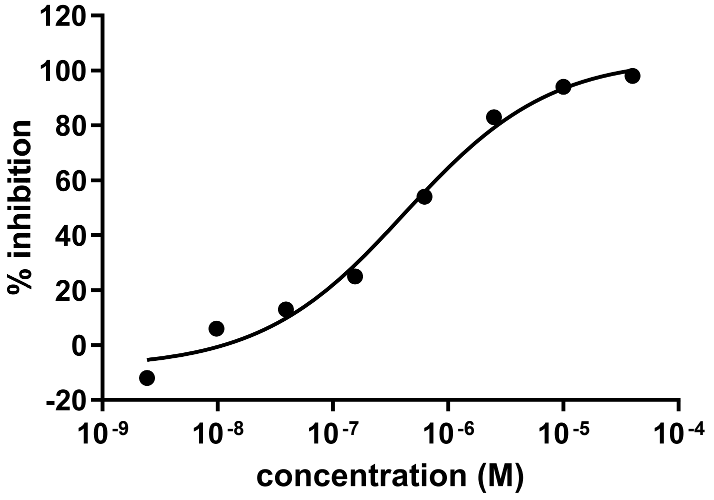

Compound B20

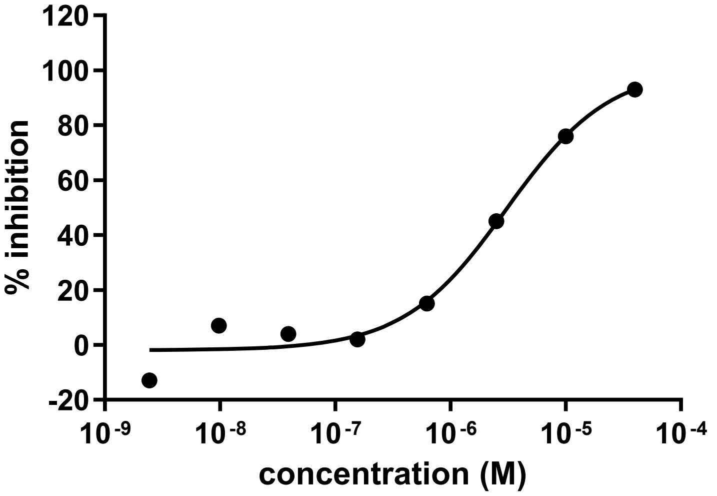

Compound B21

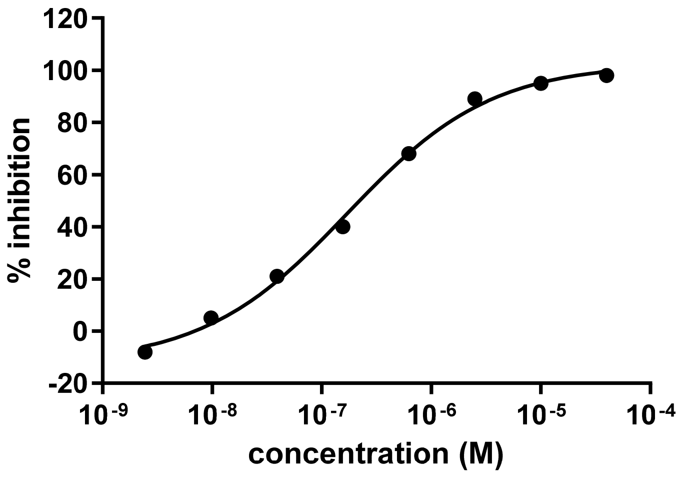

Compound B22

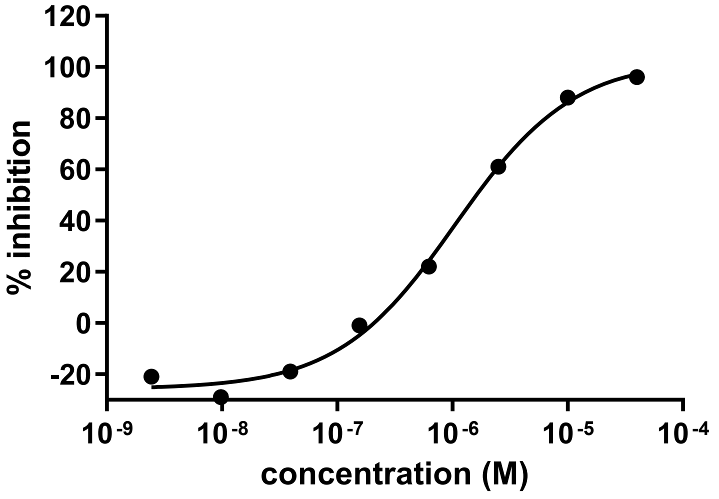

Compound B23

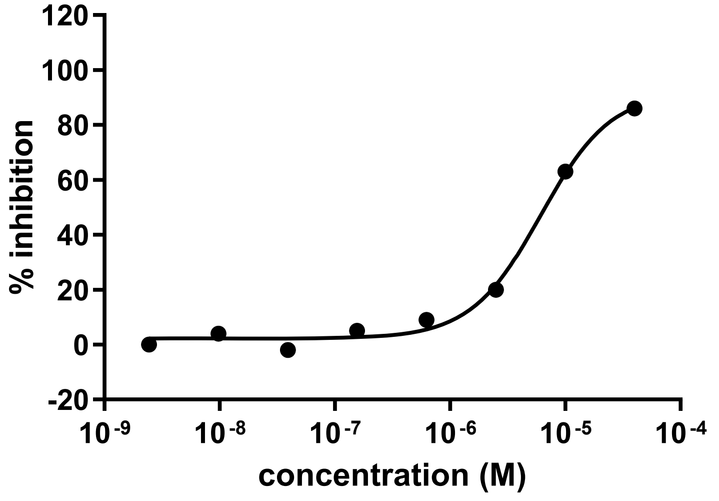

Compound B27

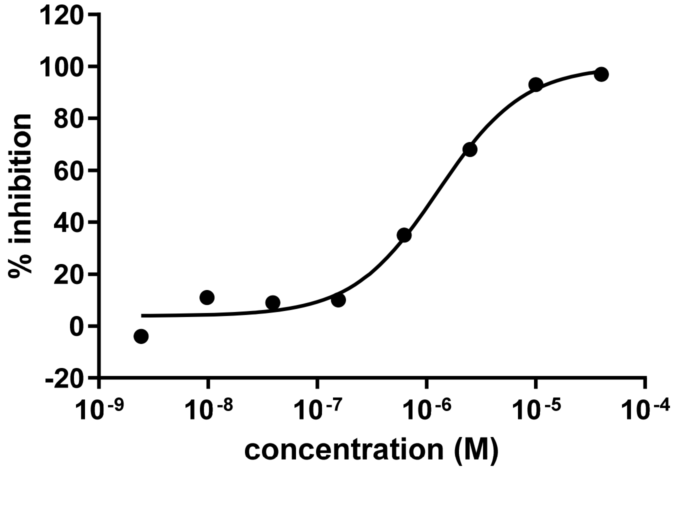

Supplement: Supplementary file 1 [file molecules-28-07415-s001.zip › molecules-2690179-supplementary.pdf]
